# A High Throughput Lipidomics Method Using *Scheduled* Multiple Reaction Monitoring

**DOI:** 10.3390/biom12050709

**Published:** 2022-05-16

**Authors:** Akash Kumar Bhaskar, Salwa Naushin, Arjun Ray, Praveen Singh, Anurag Raj, Shalini Pradhan, Khushboo Adlakha, Towfida Jahan Siddiqua, Dipankar Malakar, Debasis Dash, Shantanu Sengupta

**Affiliations:** 1 CSIR-Institute of Genomics and Integrative Biology, Mathura Road, New Delhi 110025, India; akash@igib.in (A.K.B.); salwa.naushin@igib.in (S.N.); praveen.singh@igib.in (P.S.); anurag.raj@igib.in (A.R.); sp300395@gmail.com (S.P.); khushiadlakha15@gmail.com (K.A.); towfida@gmail.com (T.J.S.); ddash@igib.in (D.D.); 2 Academy of Scientific and Innovative Research (AcSIR), Ghaziabad 201002, India; 3 Department of Computational Biology, Indraprastha Institute of Information Technology, Okhla, New Delhi 110020, India; arjun@iiitd.ac.in; 4 Nutrition and Clinical Services Division, International Centre for Diarrheal Disease Research, Dhaka 1212, Bangladesh; 5 SCIEX, 121, Udyog Vihar, Phase IV, Gurgaon 122015, India; dipankar.malakar@sciex.com

**Keywords:** lipidomics, *scheduled* MRM, plasma lipidome, vitamin B_12_, isomers, mass spectrometry, variable RT window, dwell time

## Abstract

Lipid compositions of cells, tissues, and bio-fluids are complex, with varying concentrations and structural diversity making their identification challenging. Newer methods for comprehensive analysis of lipids are thus necessary. Herein, we propose a targeted-mass spectrometry based lipidomics screening method using a combination of variable retention time window and relative dwell time weightage. Using this method, we identified more than 1000 lipid species within 24-min. The limit of detection varied from the femtomolar to the nanomolar range. About 883 lipid species were detected with a coefficient of variance <30%. We used this method to identify plasma lipids altered due to vitamin B_12_ deficiency and found a total of 18 lipid species to be altered. Some of the lipid species with ω-6 fatty acid chains were found to be significantly increased while ω-3 decreased in vitamin B_12_ deficient samples. This method enables rapid screening of a large number of lipid species in a single experiment and would substantially advance our understanding of the role of lipids in biological processes.

## 1. Introduction

Lipids constitutes highly diverse biomolecules, which play an important role in the normal functioning of the body, maintaining cellular homeostasis, cell signaling, and energy storage [1,2,3,4,5]. Dysregulation of lipid homeostasis is associated with a large number of pathologies such as obesity and diabetes [6,7], cardiovascular disease [8], cancer [9], and other metabolic diseases [10]. Lipid compositions of cells, tissues and bio-fluids are complex, reflecting a wide range of concentrations of different lipid classes and structural diversity within lipid species [11,12]. Although the exact number of distinct lipids present in cells is not exactly known, it is believed that the cellular lipidome consists of more than 1000 different lipid species, each with several structural isomers [4,13,14,15].

Identification of lipids using traditional methods such as thin layer chromatography (TLC), nuclear magnetic resonance (NMR), and soft ionization techniques (field desorption, chemical ionization or fast atom bombardment) are limited by their lower sensitivity and accuracy and hence are not suitable for comprehensive lipidomics studies [16,17]. Recent advances in electrospray ionization-mass spectrometry (ESI-MS) based lipidomics have enabled accurate identification of a large number of lipid species from various biological sources [18,19]. Analysis of lipids in both positive and negative ion modes in a single mass spectrometric scan using untargeted or targeted approaches have been used for greater coverage with increasing sensitivity and specificity [20,21]. The limitations of untargeted lipidomics approach with respect to characterization of the lipid species, processing time and bias towards the detection of lipids with high-abundance [19,22], are greatly reduced using a targeted approach by means of multiple reaction monitoring (MRM) [22,23]. A *scheduled*-MRM, where the MRM transitions are monitored only around the expected retention time of the eluting lipid species, enables monitoring of greater number of MRM transitions in a single MS acquisition [21,24,25,26,27].

A variable retention time (RT) window width, specific for each lipid species could reduce the analysis time of a *schedule*-MRM method, thereby increasing the throughput [28,29,30,31,32,33,34]. Further, the quality of peaks can be improved by varying dwell time weightage for each transition without compromising the cycle time [35]. Assigning a low dwell time weightage to high abundant compounds and high dwell time weightage to less abundant compounds, irrespective of the elution window, helps in accommodating large number of transitions in a single run with improved data quality.

Optimizing a combination of *scheduled*-MRM, variable RT window and dwell time weightage, we report a rapid and sensitive targeted lipidomics method capable of identifying more than 1000 lipid species, in a single MS run-time of 24 min. This method can be used as a first screening method in large-scale lipidomics experiments. We further exploited this method to screen lipid species altered in vitamin B_12_ deficiency in the Indian population.

## 2. Materials and Methods

### 2.1. Chemicals and Reagents

MS-grade acetonitrile, methanol, water, 2-propanol (IPA) and HPLC-grade dichloromethane (DCM), were purchased from Biosolve (Dieuze, France); ammonium acetate and ethanol were obtained from Merck (Merck & Co. Inc., Kenilworth, NJ, USA). Lipid internal standards used in the study: SM (d18:1–18:1(d9)), TAG (15:0–18:1(d7)-15:0), DAG (15:0–18:1(d7)), LPC (18:1(d7)), PC (15:0–18:1(d7)), LPE (18:1(d7)), PE (15:0–18:1(d7)), PG (15:0–18:1(d7)), PI (15:0–18:1(d7)), PS (15:0–18:1(d7), PA (15:0–18:1(d7)) in the form of Splash mix and ceramide (17:0) were purchased from Avanti polar (Alabaster, AL, USA).

### 2.2. Lipid Extraction from Human Plasma

We used a modified *Bligh and Dyer method* using dichloromethane/methanol/water (2:2:1 *v*/*v*). The study was approved by institutional ethical committee of CSIR-IGIB. Human plasma (10 μL) was mixed with 490 μL of water (in glass tube) and incubated on ice for 10 min. Lipid internal standard mixes (5 µL, consisting of splashmix and ceramide) was added to a mixture of methanol (2 mL) and dichloromethane (1 mL); the mixture was vortexed and allowed to incubate for 30 min at room temperature. After incubation, 500 μL water and 1 mL dichloromethane was added to the solution and vortexed for 5 s. The mixture was centrifuged at 300 g for 10 min for a phase separation. The lower organic layer was collected into a fresh glass tube. 2 mL dichloromethane was added to remaining mixture in extraction tube and centrifuged again to collect the lower layer. The previous step was repeated one more time. Solvent was evaporated in vacuum dryer at 25 °C and the lipids were resuspended in 100 μL of ethanol; vortexed for 5 min, sonicated for 10 min and again vortexed for 5 min. The suspension was transferred to polypropylene auto sampler vials and subjected to LC-MS run.

### 2.3. Liquid Chromatography-Mass Spectrometry

We used an Exion LC system with a Waters AQUITY UPLC BEH HILIC XBridge Amide column (3.5 µm, 4.6 × 150 mm) for chromatographic separation. The oven temperature was set at 35 °C and the auto sampler was set at 4 °C. Lipids were separated using buffer A (95% acetonitrile with 10 mM ammonium acetate, pH-8.2) and buffer B (50% acetonitrile with 10 mM ammonium acetate, pH-8.2) with following gradient: with a flow rate of 0.7 mL/minute, buffer B was increased from 0.1% to 6% in 6 min, increased to 25% buffer B in next 4 min. In the next 1 min buffer B was ramped up to 98%, further increased to 100% in the next 2 min, and held at the same concentration and flow rate for 30 s. Flow rate was increased from 0.7 mL/min to 1.5 mL/min and 100% buffer B was maintained for the next 5.1 min. Buffer B was brought to initial 0.1% concentration in 0.1 min and column was equilibrated at the same concentration and flow for 4.3 min before flow rate was brought to initial 0.7 mL/minute in next 30 s and maintained at the same till the end of 24 min gradient. Additionally, the separation system was equilibrated for 3 min for subsequent runs. A single injection of 10 μL was used.

Sciex QTRAP 6500+ LC/MS/MS system in low mass range, Turbo source with Electrospray Ionization (ESI) probe was used with the following parameters; curtain gas (CUR): 35 psi, temperature (TEM): 500-degree, source gas 1(GS1): 50 and source gas 2 (GS2): 60 psi, ionization voltage (IS): 5500 for positive mode and IS: −4500 for negative mode, target scan time: 0.5 s, scan speed: 10 Da/s, settling time: 5.0000 ms and MR pause: 5.0070 ms. Acquisition was carried out using Analyst 1.6.3 software.

### 2.4. Method Development

For identification and relative quantification of all of the lipid species, theoretical MRM library were used [35]. Using internal standards from different lipid classes, the MRM parameters (collision energy, declusturing potential, cell exit potential, and entrance potential) were optimized for 1218 lipid species which belonged to 17 lipid classes. These were sphingomyelin (SM), ceramide (Cer), cholesterol ester (CE), monoacylglycerol (MAG), diacylglycerol (DAG), Triacylglycerol (TAG), phosphotidic acid (PA), lysophosphatidylcholine (LPC), phosphatidylcholine (PC), lysophosphatidylethanolamine (LPE), phosphatidylethanolamine (PE), lysophosphatidylinositol (LPI), phosphatidylinositol (PI), lysophosphatidylglycerol (LPG), phosphatidylglycerol (PG), lysophosphatidylserine (LPS), and phosphatidylserine (PS) (Supplementary Table S1).

The MRM library consisted of 1230 transitions including 12 internal standards, of which 611 species were identified in positive mode (SM, CE, Cer, TAG, DAG, MAG) and 619 identified in negative mode (Phospholipids and lysophospholipids). The current MRM panel covers major lipid classes and categories having fatty acids with 14–22 carbons and 0–6 double bonds per fatty acyl chain [35]. Transitions were distributed into multiple unscheduled MRM method and the relative retention time of each transition was determined with respect to their respective internal standards through Amide-HILIC column. Furthermore, the retention time validation was carried out for SM and PC by performing MS/MS experiment using Information dependent acquisition (IDA) with enhanced product ion scan (EPI) of specific ions in unscheduled MRM for each lipid class. MS/MS analysis in EPI mode was based on the conventional triple quadrupole ion path property of an ion-trap for the third quadrupole. LC gradient used in this experiment is same as of the *scheduled* MRM and the ion source parameters were kept the same as mentioned in MRM experiment. The mass range scanned between 100 and 1000 Da in positive as well as negative polarity for EPI spectra. The acquired MS/MS spectra were compared with fragmentation information from LIPID MAPS (http://www.lipidmaps.org/) (accessed on 1 September 2021) to verify the structures of the putative lipid species and predicting the structure from MS/MS spectra based on specific cleavage rules for lipids.

### 2.5. Variable Retention Time Window (Variable-RTW) and Relative Dwell Time Weightage (Relative DTW)

Using *scheduled* MRM Builder [35], an Excel based tool from Sciex, the variable retention time window and variable dwell time weightage for all transitions were optimized. The principle on which the tool works is based on the variation in width, area, and retention time of the chromatographic peak [35]. With variable retention time window width, each MRM transition can have its own RT window. Wider windows are assigned to analytes that show higher run-to-run variation or have broader peak widths. Variable dwell times were assigned to improve the signal to noise ratio of MRM transitions based on the abundance of the analyte in the sample, higher dwell time weightage assigned for analytes with low abundance. Dwell time for each species were assigned based on this weight, which maintains the cycle time and optimizes the signal to noise ratio for low abundant peaks.

### 2.6. Comparison of MRM, Scheduled MRM and Scheduled MRM with Variable Retention Time Window and Relative Dwell Time Weightage

After optimization of dwell weight and RT window, we compared the area under the curve obtained in XIC (extracted ion chromatogram) for different lipid species in advanced MRM (*scheduled* MRM with variable-RTW and relative-DTW) with *s*MRM and MRM. All of the parameters of LC and MS were same in three types of scan. From the pooled plasma (pooled from five samples), lipids were extracted on three different days and subjected to MS run with five technical replicates on each day. 

### 2.7. Identification of Isomers within Lipid Classes

For identification of isomers within lipid species from human plasma (*n* = 30, biological replicate), we used customized-approaches for various lipid classes. For TAGs, the selection of unique parent ion/daughter ion (Q1/Q3) combination was based on neutral loss of one of the *sn*-position fatty acyl chain (RCOOH) and NH_3_ from parent ion [M + NH_4_]+ while the phospholipids were analyzed in negative ion mode to identify the different isomers within different classes at fatty acid composition level. After prior blank subtraction for the samples, further analysis was performed:

1. There was less than 10% missing values and these were imputed with the median value of the particular lipid species across the samples.
2. Based on the sum of the peak areas of all TAG/PL species, the species abundance and the isomer abundance within that species were calculated (Supplementary Tables S3 and S4).

### 2.8. Limit of Detection and Quantitation

The limits of detection (LoD) and quantitation (LoQ) were derived from peak area of known amounts of lipid internal standards added to lipid extract from human plasma (matrix):

The master mix of lipid internal standards was prepared from splashmix and ceramide (17:0) having following concentrations: SM (41.86 nmol), Cer (24.91 nmol), TAG (70.59 nmol), DAG (15.99 nmol), LPC (48.23 nmol), PC (213.38 μmol), LPE (10.89 nmol), PE (8.02 nmol), PG (38.09 nmol), PI (5.40 nmol), PS (10.74 nmol), PA (10.73 nmol).

Limit of Blank (LoB)- was defined as the average (based on triplicate experiments) signal found only in matrix (without internal standards; blank). LoB was calculated using mean and standard deviation from plasma matrix [36]:

LoB = mean blank + 1.645(SD blank)

The raw analytical signal obtained for standards from plasma lipid extract (spiked with standards) was used to estimate the LoD and LoQ [37], using the following formula:

LoD = mean blank + 3(Standard Deviation blank)

LoQ = mean blank + 10(Standard Deviation blank)

The standard solution was diluted serially with matrix and the lipid standards were run in the following concentration ranges: 319.39 fmol- 41.86 nmol for SM, 190.06 fmol- 24.91 nmol for Cer, 538.53 fmol-70.59 nmol for TAG, 121.97 fmol- 15.99 nmol for DAG, 367.96 fmol- 48.23 nmol for LPC, 1.63 pmol- 213.38 μmol for PC, 83.09 fmol-10.89 nmol for LPE, 61.16 fmol- 8.02 nmol for PE, 290.59 fmol- 38.09 nmol for PG, 41.24 fmol- 5.40 nmol for PI, 81.96 fmol- 10.74 nmol for PS, 81.83 fmol- 10.73 nmol for PA. The lowest concentration which has signal more than the estimated method limits (based on above formula) was considered as LoD and LoQ. The mean and standard deviation was calculated from three replicates. Linearity was represented by R^2^, where LoQ was taken as the lowest calibrator concentration for each lipid standards.

### 2.9. Spike and Recovery and Coefficient of Variance

Extraction recovery for the method was measured by comparing the peak area of matrix extract spiked with standards before and after extraction. For this, 5 uL of lipid internal standard mix (standard mix: lipid extract resuspension volume:: 1:20 *v*/*v*) was used. The percentage recovery and relative standard deviation was calculated from 3 biological replicates [38].

Relative recovery = Mean area of extracted sample with spiked standard before extraction/Mean area of extracted sample with spiked standard after extraction

%Relative Standard Deviation = Standard Deviation/Mean analytical signal × 100

Coefficient of variance (CV) of the method was determined by observing individual lipid species variation within batch. The intra-batch variation was assessed by analyzing five technical replicates of lipids extracted from pooled plasma. CV values were only calculated for those lipid species which had carry over less than 20% and present in all five technical replicates [39]. Inter day variability for each lipid species was determined by analyzing lipids on three different days from a stock of pooled plasma. The CV values were reported for three different days (*n* = 5, technical replicates) after sum-normalization within lipid class.

Percentage CV = standard deviation/average intensity ×100

### 2.10. Alteration of Plasma Lipids Due to Vitamin B_12_ Deficiency

With respect to the study population, the study (which was a part of a larger study), was designed to identify plasma lipids that were altered due to vitamin B_12_ deficiency. Apparently healthy individuals were classified in two groups based on their plasma vitamin B_12_ levels. An informed consent was obtained from the participants. The study was approved by institutional ethical committee of CSIR-IGIB. Individuals with vitamin B_12_ values less than 150 pg/mL, were considered to be vitamin B_12_ deficient and those with levels between 400 and 800 pg/mL were considered be in the normal range [40,41]. Lipids from plasma were extracted as described above. For this study, plasma of 93 individuals (47 with B_12_ deficiency and 46 with normal plasma vitamin B_12_ levels) were used, where 39 were males and 54 were females. In the vitamin B_12_ deficient group, 29 females (60%) while in the control group 25 females (53%) were present. The median age in the vitamin B_12_ deficient group was 40 years, which was not significantly different from the control group 40.5 years (*p* > 0.05). The list of the samples with their vitamin B_12_ values, age and sex is given in Supplementary Table S5.

Data processing: After acquiring the data, lipid class specific normalization was carried out for 849 lipid species (only those lipid species which are consistently identified from pooled plasma were considered) from the MRM library of 1218 lipid species. Furthermore, the lipid species which have missing values in 10 or more samples were filtered out. The data from 93 samples with 759 lipid species having less than 1% missing values were considered for further analysis:

1. Imputation was carried out for less than 1% missing data in the dataset with median value based on lipid species specific value across the two groups.
2. Batch effect correction for 759 lipid species was carried out to reduce technical variance and remove sex bias using the ComBat function from the SVA package [42].

Data analysis:

1. Differential expression (DE) analysis was performed by two-sided Wilcoxon rank-sum test, where 55 lipid species were found to be altered by more than 1.3 folds with *p* < 0.05 between the two groups (Supplementary Table S7).
2. For important feature selection, Boruta algorithm using random forest was applied to identify possible lipid markers and iteratively removes lipid species that were statistically less relevant than a random probe between the two groups [42]. The ‘Boruta’ function from the R library package was applied on 93 samples with 55 lipids as features, where 18 lipids were finally selected as putative markers.
3. A two-sample Wilcoxon test with *p*-value adjustment (BH method) was further applied to show the significant relationship between the categories of identified lipid markers.

### 2.11. Data Analysis

The .wiff files for relative quantitation were processed in MultiQuant 3.0.2 and for the identification of different lipid species; MS/MS spectrum matching with the structure of putative lipid species using .mol file was carried out in Peakview 2.0.1 software. Statistical analysis was carried out using Excel. Figures were drawn using MATLAB (The MathWorks Inc., Natick, MA, USA), Raw graph (https://rawgraphs.io, accessed on 18 January 2022) and GraphPad Prism version 6.0 (GraphPad Software, San Diego, CA, USA).

## 3. Results

We developed a *scheduled*-MRM method that can identify more than 1000 lipid species in a single mass spectrometric acquisition using a combination of variable-RTW and relative-DTW for each lipid species with an optimized LC-gradient [35]. The theoretical MRM library consisted of 1218 lipid species and 12 internal standards, belonging to the 17 lipid classes [35]. The 17 classes of lipids were analyzed in the positive or negative ion modes. In the positive ion mode, the M+H precursor ions were used for SM and Cer while for neutral lipids (CE, TAG, DAG, and MAG), [M+NH_4_]+ precursor ions were considered. Phospholipids (PL’s) were identified in negative ion mode, forming [M-H]- precursor ion except LPC’s and PC’s, for which [M+CH_3_COO]- were considered. The variable-RTW and relative-DTW for different species was determined using *scheduled* MRM Builder tool which is based on the area, width and retention time of the peaks obtained for each lipid species [35]. We were able detect a total of 1083 lipid species from the MRM library of 1218 lipid. A complete list of all parameters for 1083 lipid species is given in Supplementary Table S1 and the representative total ion chromatogram (TIC) is shown in Supplementary Figure S1. 

**Figure 1:**
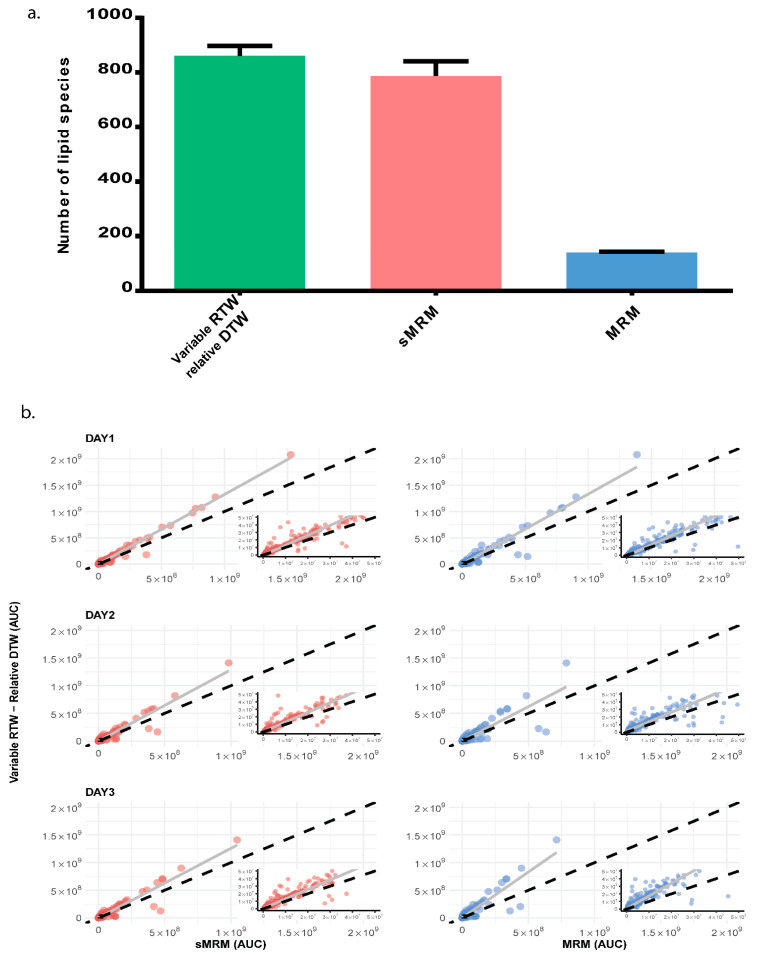
**Comparison of three different MS methods (Variable RTW-Relative DTW, *s*MRM and MRM) on three different days.** (**a**) Total number lipid species obtained through Variable RTW-Relative DTW (829, 901 and 852), sMRM (728, 832, and 802) and MRM (141, 143, and 138) methods. Data shown as mean ± SD of experimental replicates on three different days. (**b**) Comparison of AUC for different lipid species detected through variable RTW- relative DTW method with *s*MRM and MRM method. The *x*-axis is the area under the curve (AUC) of XIC (extracted ion chromatogram) for detected lipids in sMRM and MRM and *y*-axis is AUC for variable RTW- relative DTW. Each dot represents lipid species. Increase in AUC for lipid species is associated with the distance between reference line (diagonal) and the trend line. For equal AUC obtained in different methods, the point should scatter around the reference line while we observed deviation from linearity (reference line) toward *y*-axis (variable-RTW and relative-DTW). Trend line shift toward *y*-axis indicate the improved data quality for variable RTW- relative DTW as compared to *s*MRM and MRM.

We also compared our method (variable RTW-relative DTW) with *scheduled* MRM (*s*MRM) and MRM to check the number of lipid species identified using the three methods. We also compared the area under the curve obtained in XIC for different lipid species. In variable RTW-relative DTW method, we detected 829, 901, and 852 lipid species which were present in all technical replicate with ≥10 data points and <30% CV on day one, day two, and day three, respectively (Figure 1a). While in *s*MRM, the number was 728, 832, and 802 and in MRM, 141, 143, and 138 for day 1, day 2, and day 3, respectively (Figure 1a). A scatter plot for area was created for variable RTW-relative DTW vs. *s*MRM and for variable RTW-relative DTW vs. MRM (Figure 1a) from the mean values. The area of the lipid species obtained in our method was higher than *s*MRM (in 89% cases) and MRM (in 78% cases) clearly indicating that not only the number of lipid species identified were greater in variable RTW-relative DTW but also the area of the species identified were greater (Figure 1b). We also checked the reproducibility of the retention time in our method and found that there was minimal variation of retention time between the technical replicates with 1044, 1058, and 1024 out of 1083 lipid species having CV of less than 5% for day one, day two, and day three, respectively.

Sphingomyelin (SM) and phosphatidyl choline (PC) both have the same head group (phosphocholine- 184.1 Da) and hence it was important to distinguish the two classes. In this method, with the gradient used, the retention time of the two classes were different. Using internal standards for SM, d18:1–18:1(d9) and PC, 15:0–18:1(d7), we found that PC has a lower retention time (10.26 min) than SM (11.81 min) (Supplementary Figure S2a,c). The mapping of MS/MS spectra with respective structures shows the presence of signature peaks for d18:1–18:1(d9) SM (Supplementary Figure S2b) in positive ion mode and for 15:0–18:1(d7) PC in negative ion mode (Supplementary Figure S2d). In this method, all of the PC species eluted between 9.09 and 11.59 min while all of the SM species eluted at 11.74 and 12.38 min (Supplementary Table S2).

### 3.1. Identification of Isomers within Lipid Classes

**Figure 2:**
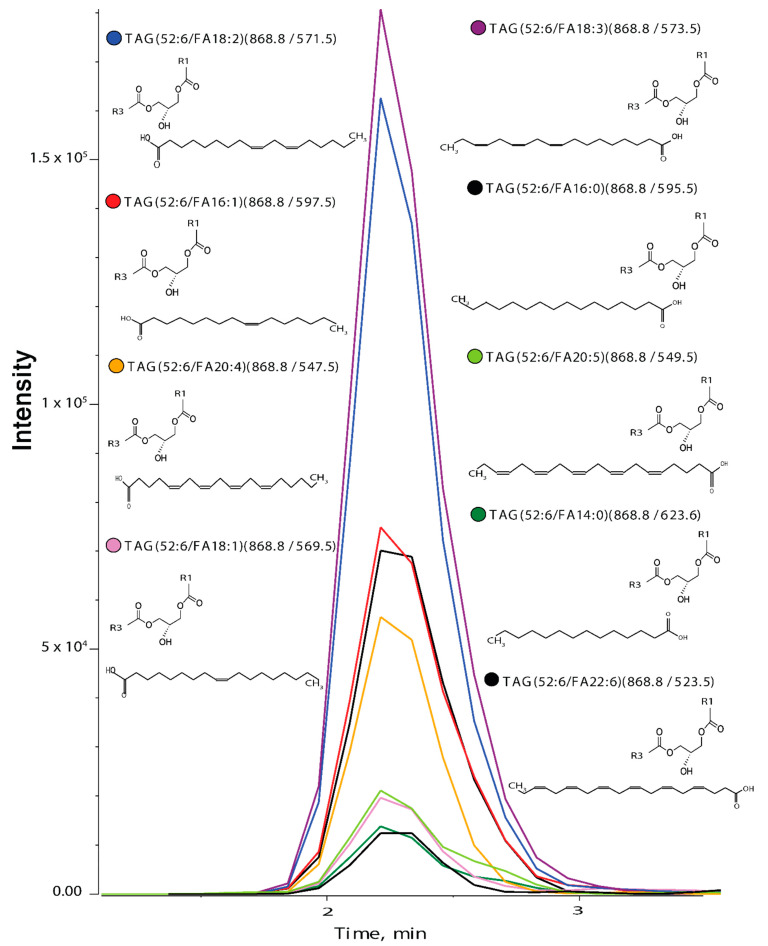
**XIC (extracted ion chromatogram) of nine isomers of TAG (52:6).** Parent *m*/*z* for all was 868.8 while the product *m*/*z* was derived from the remaining mass (R1 + R2 with glycerol backbone) after the loss of fatty acid released from the parent ion. R1 + R2 can be any composition of fatty acid which sum-up to give product ion. Different colored dots represent different isomers.

**Figure 3:**
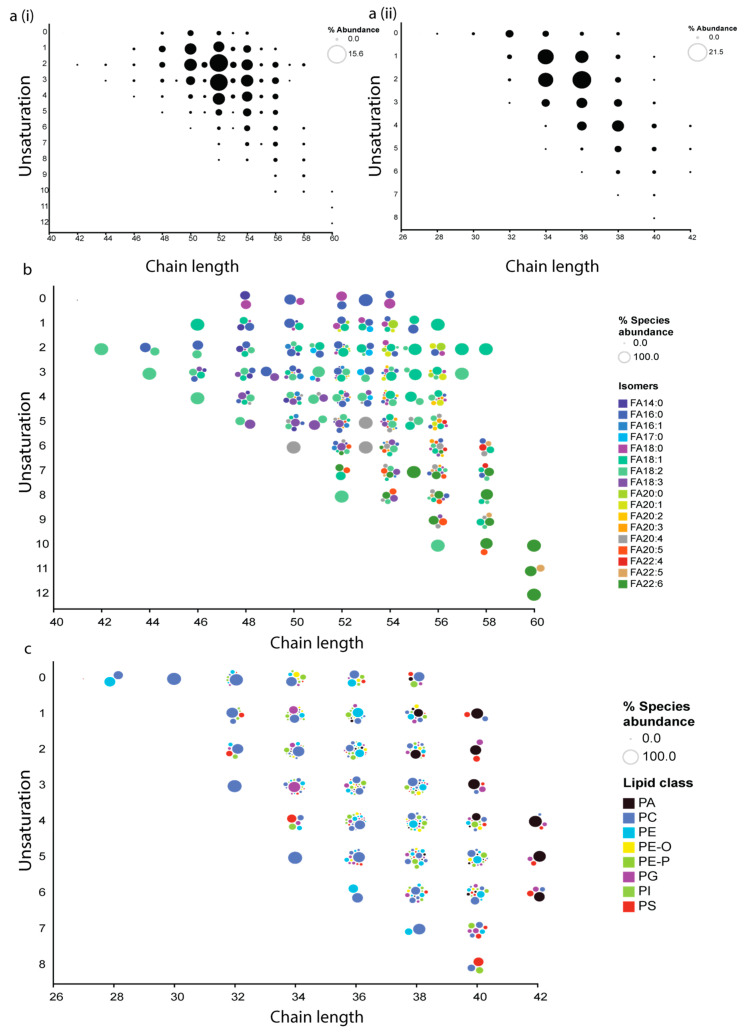
**Abundance of different lipids.** (**a(i)**) Abundance of different TAGs on the basis of total chain length (as a function of main-chain carbon atoms) and unsaturation. (**a(ii)**) Abundance of different phospholipids on the basis of total chain length and unsaturation. (**b**) A total of 339 TAG isomers were detected from 77 different species of TAG. (**c**) Abundance of 365 phospholipids belonging to 6 classes (PC, PE, PG, PI, PS, and PA), different dots of same color represent isomers. The abundance of the difference lipids/isomers is represented by the varying size of the bubble in all of the panels.

In an attempt to identify different lipid isomers, we used customized-approaches for various lipid classes. For TAGs, instead of using pseudo-transitions, we identified different isomers of TAG species on the basis of *sn*-position by selecting a unique parent ion/daughter ion (Q1/Q3) combination, which is based on neutral loss of one of the *sn*-position fatty acyl chain (RCOOH) and NH_3_ from parent ion [M+NH_4_]+. For instance, the parent ion (Q1) for TAG 52:6 is 868.8 while the product ion (Q3) was derived from the remaining mass of TAG after loss of fatty acid present at one of the *sn*-position such as *m/z* 595.5 for TAG (52:6/FA16:0) as shown in Figure 2. Using this approach, we found nine isomers for TAG species (52:6) based on composition of fatty acid present at one of the *sn*-position (Figure 2). From the MRM library which consisted of 445 TAG isomers, from 96 different TAG species, we were able to identify 339 TAG isomers in human plasma (*n* = 30, biological replicates) from 77 different TAG species (Figure 3a(i),b). Among these TAG species, TAG (52:2) was the most abundant (15.6%) form in human plasma (Figure 3a(i)) which had nine isomers, of which TAG (52:2/FA18:1) was the most abundant (52.4%)(Figure 3b). A list of TAG species with their isomers is given in (Supplementary Table S3).

For phospholipids (PC, PE, PG, PS, PI, and PA), instead of the conventional method of using the head group loss in positive ion mode (e.g., PC-38:4, 868.607/184.1), we used a modified approach using negative ion mode via the loss of fatty acid to identify the phospholipids at the fatty acid composition level. Using this approach, we were able to identify isomers of phospholipids within a class, such as PC16:0–22:5, PC 18:0–20:5, PC 18:1–20:4 and PC 18:2–20:3 for PC 38:5 (Supplementary Table S4). From the analysis of 455 phospholipids belonging to 6 phospholipid classes (PC, PE, PG, PI, PS, and PA) in the library, we were able to identify 365 phospholipid species (*n* = 30, biological replicates). Among them, phospholipids (PC, PE, PG, PI, PS, PA) with chain length 36 with 2 unsaturation had the highest abundance (21.5%) (Figure 3a(ii)). Within 36:2 PLs, PE 18:0/18:2 (35.3%) and PC 18:0/18:2 (27.3%) has highest abundance (Figure 3c). A list of PL’s species with their isomers is given in (Supplementary Table S4).

### 3.2. Limit of Blank (LoB), Limit of Detection (LoD), Limit of Quantitation (LoQ), and Linear Range

**Figure 4:**
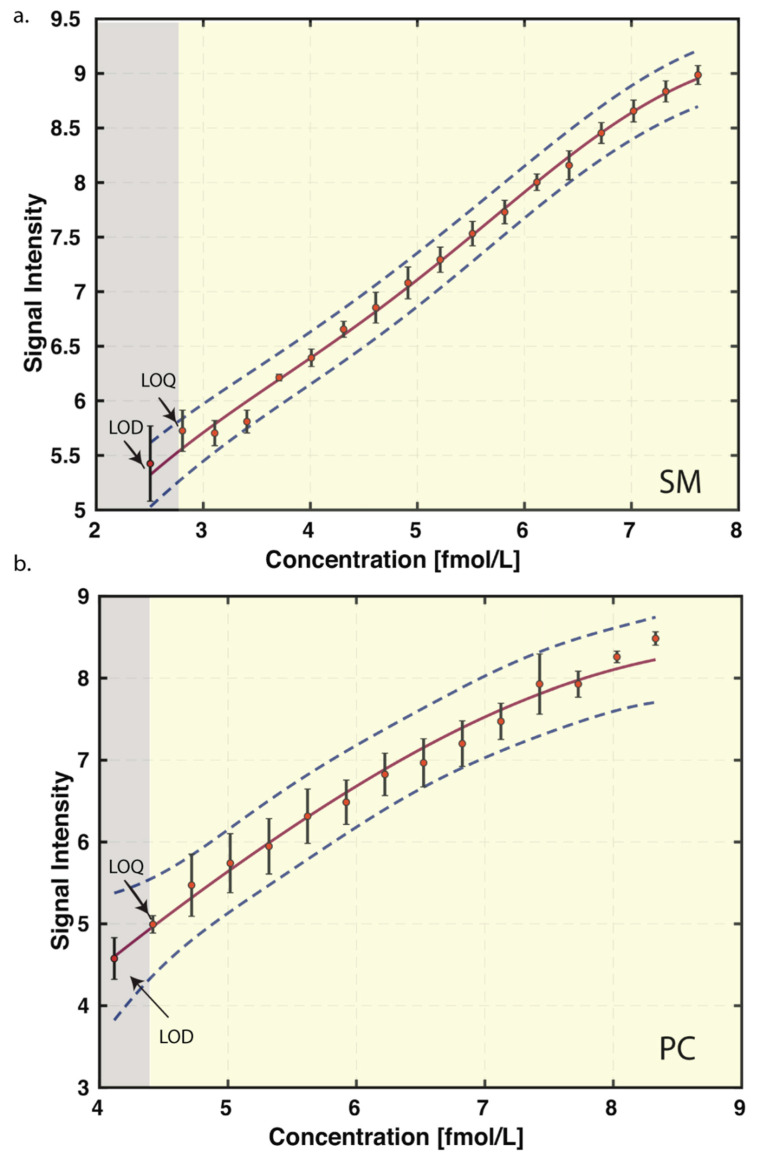
**Representative graphs from positive and negative ion mode showing LoD, LoQ and coefficient of determination, *x* and *y*-axis was log transformed.** (**a**) SM from positive ion mode and (**b**) PC from negative ion mode. The grey area represents the concentration below the linear range while the yellow region is indicative of linear range. The error bar represents the variance/standard deviation obtained in three replicates, reflecting inter day variability.

The raw analytical signal in blank was considered for establishing the LoB, which was determined from the area under the chromatogram for the selected transition of each lipid standards. The LoD and LoQ were obtained from the raw analytical signal (area under the chromatogram) obtained by progressively diluting the lipid standards. The LoD and LoQ were based on the average values obtained in three replicates, reflecting inter day variability as mentioned in the materials and methods section. A representative graph of LoD and LoQ for SM (positive mode) and PC (negative mode) is shown in Figure 4a,b, while the values of LoD and LoQ for other lipid classes are provided in Table 1. The LoDs were in range of 0.245 pmol/L–41.961 pmol/L except for DAG (1 nmol/L). Detection limit for SM, LPC, PE, and PG were found to be in femtomolar range, while the rest were in the picomolar range. The lowest LoQ was detected for PG- 0.291 pmol/L and highest for DAG- 2 nmol/L.

The linearity of the method was checked by defining the relationship between raw values of analytical signal for each lipid standard and its concentration in presence of matrix (plasma). The linear range was determined by checking the performance limit from LoQ to the highest end of the concentration; based on the coefficient of determination (R^2^) value (Table 1).

### 3.3. Spike and Recovery and Coefficient of Variation

**Figure 5:**
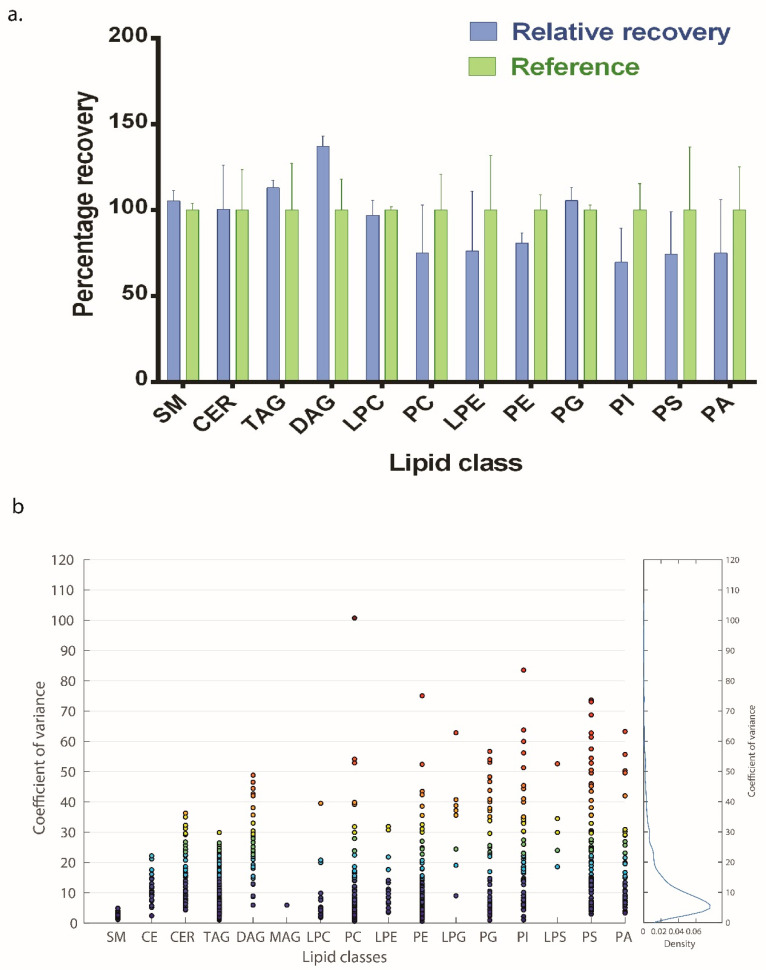
**Validation of the method.** (**a**) Spike and recovery of different lipid classes where blue bars represent the recovery of lipids when known concentration of lipid standards was spiked during extraction and green bars represents the reference (same concentration of lipid standard spiked after extraction). (**b**) Coefficient of variance on day 2 where 1043 lipid species from 16 lipid classes were detected (*n* = 5). The color scale of the bubble is based on the function of coefficient of variance in the increasing order. The right-hand panel represents the density function w.r.t. coefficient of variance.

To determine the percent recovery of all of the lipid species, a known amount of lipid standards was added to plasma (matrix) before or after (spike) extraction of the lipids from the plasma. The experiment was carried out in three replicates. The raw area signals obtained from these two conditions were compared to determine the percentage recovery. These experiments were performed on three different days and the average percent recovery of the lipid standards is provided in Figure 5a and Supplementary Table S6.

To determine the coefficient of variation of all of the lipid species, we extracted lipids from plasma pooled from five individuals. For intra batch variations, the same sample was subjected to mass spectrometric analysis five times. The coefficient of variation was calculated after sum normalization of raw values obtained within each class. To obtain the inter day variability, lipids were extracted from the same sample on three different days. A total of 929, 1043, 1000 lipid species were detected in all five technical replicates on day one, day two, and day three, respectively. The median CV of all of the identified lipids on three different days was 6.98%, 8.28%, and 8.18%, respectively. On day 1 out of 929 lipid species, we observed 879 lipid species with CV below 30%. Of these, 627 had CV < 10% and 812 had CV < 20%(Figure 5b). We observed 947 and 907 lipids species on day two and day three, respectively, with CV < 30%. In total, we identified 890 lipid species consistently on all days with 883 lipids having CV < 30% in either of the three days, out of which 812 lipid species has been consistently detected in all days with CV < 30%. The detailed table with CV and data points for individual lipid species observed on three different days is given in Supplementary Table S1.

### 3.4. Lipidomics Study in Normal and Vitamin B_12_ Deficient Human Plasma

**Figure 6:**
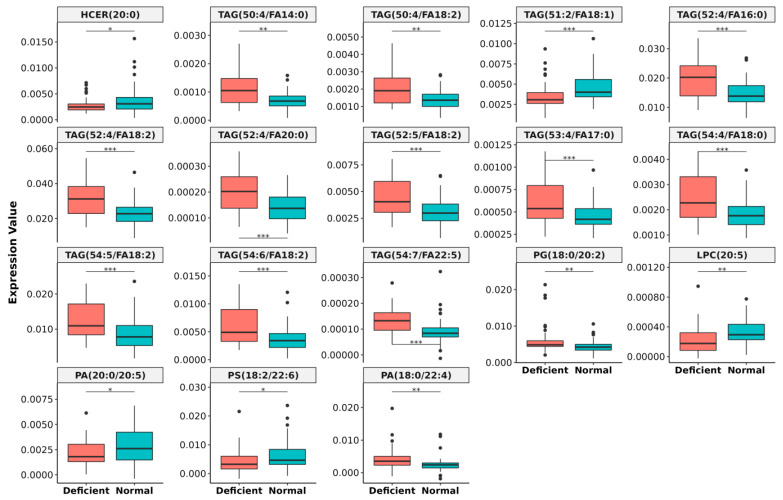
**Significantly altered lipid species in vitamin B_12_ deficiency.** The significance level for different lipid species are represented by p-value ( * = *p* < 0.05, ** = *p* < 0.01 and *** = *p* < 0.001).

As an application of the method, we attempted to identify the plasma lipid species that are altered due to vitamin B_12_ deficiency. Vitamin B_12_ is a micronutrient mainly sourced from animal products, deficiency of which has been reported to result in lipid imbalance [43]. There was no significant alteration in any of the lipid classes when taken as a whole (Supplementary Table S7). However, when individual lipid species within the classes were compared using differential expression analysis, a total of 55 lipid species were found to be altered between the two groups (Supplementary Table S8). After feature selection using random forest through Boruta algorithm and *p*-value correction using Benjamini and Hochberg (BH) method, we found that 18 lipid species were significantly altered (Figure 6). We observed that 11 TAG species composed of poly unsaturated fatty acids having chain length of 50–54 carbon and 4–7 unsaturation were found to be significantly higher in vitamin B_12_ deficiency while one TAG species containing odd carbon number-51 and total of two unsaturation was downregulated in deficient condition. Additionally, we observed that lipid species (LPC 20:5, PA 20:0/20:5) containing one of the types of omega 3 fatty acid (FA 20:5) was significantly low in plasma of vitamin B_12_ deficient individuals and 5 lipid species containing omega 6 fatty acid (FA 18:2) was significantly high in vitamin B_12_ deficient condition (Figure 6). These results hint at the possibility of lower ω-3: ω-6 ratio in vitamin B_12_ deficient individuals. Thus, these results show the utility of identifying species specific lipids for which this method has been developed.

## 4. Discussion

Lipids in general are known to be associated with the pathogenesis of various complex diseases [10]. However, the exact role played by each lipid species has not been studied in detail majorly due to the limitation in identifying individual lipid species in large scale studies. We report a single extraction, targeted mass spectrometric method using Amide-HILIC-chromatography (scheduled MRM with variable-RTW and relative-DTW) which detects more than 1000 lipid species from 17 lipid classes including various isomers in a single MS run-time of just 24 min per sample injection. This method covers most of the lipid species with 14–22 carbons atoms and 0–6 double bonds in fatty acid chain which are the most prevalent in human plasma [44,45,46]. This method enables identification of a considerably larger numbers of lipid species than those reported previously [14,21,47,48,49] and hence could be a method of choice for the initial screening for any large scale lipidomics study.

In this method, the MRM transitions were monitored in a particular time segment, rather than performing scans for all of the lipid species during the entire run. This strategy reduces the time required for identification of the multiple transitions during a run. We improved the coverage by additionally optimizing the assigned dwell time weightage for each lipid species, which is required especially for medium and low abundant lipid species. The dwell time for each lipid species was customized and the dwell weightage was optimized based on lipid species abundance, elution window width, and variation in retention time without affecting the target scan time in each cycle [35]. This improved peak quality with good reproducibility. Our method enabled identification of greater number of lipid species compared to both MRM and *scheduled* MRM. Further, the area under the curve for lipid species in our method was consistently higher than obtained in *s*MRM. 

We took the advantage of an amide linked HILIC column for the elution of both polar and non-polar lipid species [50], which is not possible with an unmodified HILIC column, that are generally used for polar metabolites only [47,51,52]. Using this column, we found that non-polar lipids such as TAGs binds to the column and elutes post void volume. Apart from this, to avoid the misidentification of phosphocholine containing head group (SM and PC), we used positive mode for identification of SM and negative mode for PC [53]. The presence of peak at *m*/*z* 184.1 in MS/MS spectrum indicates the presence of phosphocholine head group, which might belong to either of them [54]. Shingomyelin species were distinguished from PC, using their retention time (and also by the fact that SM precursor ions had an odd number *m*/*z* and PC had an even number (nitrogen rule [54,55])). However, the rules may not hold in case of PC isotopes which have same *m*/*z* precursor ion as of SM (e.g., the 13C isotope/2H isotope of a PC would be present as an odd *m*/*z* ion) and vice-versa [53,56,57]. Fatty acid present at *sn*-1 and *sn*-2 position of glycerol backbone was used to identify PC based on the product ions in negative mode (Supplementary Table S1), which was confirmed from MS/MS spectra through EPI scan (Supplementary Figure S2). 

Current methods for large-scale lipid analysis can only identify the lipid classes and total fatty acyl composition of lipid species but the structure specificity is critical for studying the biological function of lipid species. Finding the composition of fatty acyl chain with respect to *sn*-position is a major limitation in large scale lipidomics studies [21,58]. Using pseudo-transitions for identifying TAG has its own disadvantages [21]. Firstly, it is based on same Q1 and Q3 *m*/*z* value (e.g.: 868.8/868.8), other compound which has same parent mass (Q1) with similar polarity, will also be eluted at same time and MS cannot differentiate between two compounds. So, scanning unique pair of Q1/Q3 transition, where Q1 is parent ion and Q3 is characteristic daughter ion, for that compound is essential. Secondly, isomers cannot be detected as Q3 is same as Q1. Recently using a combination of photochemical reaction (Ozone-induced dissociation and ultraviolet photo-dissociation) with tandem MS, Cao et al. reported the identification of isomers for TAGs and PLs on the basis of *sn*-position and carbon-carbon double bond (C=C) [59]. Their identification also revealed the sequential loss of different fatty acyl chain based on *sn*-position, disclosing identification of different positional isomers [59]. However, a single step identification of TAG isomers in large scale studies remains a challenge due to the three fatty acyl chains with glycerol backbone, bearing no easily ionizable moiety [21,58,60]. We have focused on identification of structural isomers based on *sn*-position using LC-MS platform, without adding extra steps to burden the analysis time and effort. We were able to detect structural isomers with respect to fatty acyl chain at *sn*-position where the neutral loss of one of the *sn*-position fatty acyl chain (RCOOH) and NH_3_ from parent ion [M+NH_4_]+ makes their detection possible. Detection was purely based on assigning a unique combination of Q1/Q3 for structural isomer of TAG species (Figure 3a). However, one of the limitations of this method is the inability to assign fatty acyl group (*sn1*, *sn2*, or *sn3*) to their respective *sn*-position. Hence, the three fatty acyl chains are represented by the adding the number of carbon atoms and unsaturation level (e.g., TAG (52:6) and the identified fatty acid at one of the *sn*-position (e.g., FA-14:0) is represented by TAG (52:6/FA14:0).

The other limitation is the inability to distinguish type-II overlap, i.e., M+2 isotopologue overlapping species which may contribute to misidentification and overestimation of some individual lipid species [57]. However, the consensus within the lipidomic community, when using nominal mass MRM, is that the contribution of the M+2 isotopologues using HILIC based chromatography is very minimal (less than 10% and many times less than 5%, which is within the error of the instrument itself) [61]. There might be an exception in case of TAGs where this error might be higher [61]. However, this method is intended to be a screen for TAGs considering the number that are eluted at similar RT. Thus, this method for quantifying TAGs is more relative quantitation but not accurate/absolute quantitation. 

The LoD for various lipid classes in our method was between 0.245 fmol/L–41.96 pmol/L which was better than or similar to previously reported LoD utilizing different LC-MS platforms [21,27,48,49,60] and similar to a previously reported large scale lipidomics method using supercritical fluid-scheduled MRM (5–1000 fmol/L) [21]. The LoQ in previously reported methods were in between nmol to μmol/L range while we have observed much lower LoQs (0.291 pmol/L to 167.84 pmol/L) [21]. Our calculation of limits was based on mean raw analytical signal and SD, which gives better idea about the method, without any false detection hope (or lower detection limits). In our method, DAG has highest LoD and LoQ of 1 nmol/L and 2 nmol/L, respectively, which was still lower as compared to the previously reported methods for targeted analysis [21]. The linearity of our method was found to be comparable to previously reported lipidomics methods [21,27,48].

The recovery of lipid species in our method was in the range of 69.75–113.19 %, except DAG-137.5%, which were within the generally accepted range for quantification and is comparable with other lipidomics studies [21,27], although, DAG class is not frequently quantified in other published papers [62]. The high recovery of DAG could be due to in-source fragmentation of TAG species [53].

A major challenge in lipidomics experiments have been the high variability in the signals and even the “shared reference materials harmonize lipidomics across MS-based detection platforms and laboratories” have shown that most lipid species showed large variability (CV) between 30% to 80% [61]. However, variability for endogenous lipid species that were normalized to the corresponding stable isotope-labelled analogue were lower than 30% [47,61]. In this method, we used lipid class specific sum normalization and found 890 lipid species consistently on all days with 883 lipids having CV < 30% in either of the three days [47]. Overall, the median CV of our method on three different days (6.98%, 8.28%, and 8.18%), was similar to or better than the previous reports [21,27,60]. In addition, we have also reported species-specific CV. It should be noted that in most of the large scale lipidomics studies previously carried out, reports the median or average CV of the method but not the species-specific CV [14,21,27,60].

### Lipidomics Study in Normal and Vitamin B_12_ Deficient Human Plasma

Using the method developed, we identified lipid species that are altered in individuals with vitamin B_12_ deficiency. Vitamin B_12_ is a cofactor of methyl malonyl CoA mutase and controls the transfer of long-chain fatty acyl-CoA into the mitochondria. Deficiency of vitamin B_12_ results in accumulation of methylmalonyl CoA increasing lipogenesis via inhibition of beta-oxidation. Plasma vitamin B_12_ levels less than 150 pg/mL is considered to be deficient [40,41].

In the last decade, several studies revealed that vitamin B_12_ deficiency causes alteration in the lipid profile through changes in lipid metabolism, either by modulating their synthesis or its transport [63]. In particular, the effects of vitamin B_12_ on omega 3 fatty acid and phospholipid metabolism have received much attention. Khaire A et al., found that vitamin B_12_ deficiency increased cholesterol levels but reduced docosahexaenoic acid (DHA-omega 3) [64]. An imbalance in maternal micronutrients (folic acid, vitamin B_12_) in Wistar rats increased maternal oxidative stress, decreases placental and pup brain DHA levels, and decreases placental global methylation levels [65,66]. Our results corroborate such findings. We also find alteration in 20:5 (eicosapentaenoic acid, ω-3) and 18:2 (linoleic acid, ω-6) chain containing lipid species. The only limitation of our method here is that it does not distinguish the position of double bonds in a given fatty acid chain. The following example illustrates this. Arachidonic acid is an ω-6 fatty acid with fatty acid composition 20:4 and double bonds at carbon position 5,8,11,14, while eicosatetraenoic acid is also 20:4 but an ω-3 fatty acid with double bonds at carbon position 8,11,14,17. Our method cannot differentiate between these two fatty acids. Thus, we cannot comment on other fatty acids containing lipid species besides eicosapentaenoic acid, linoleic acid and docosahexaenoic acid. Additionally, we found one lipid species PS (18:2/22:6) having one chain of docosahexaenoic acid (22:6), an ω-3 fatty acid, to be significantly altered between the two groups. However, the other fatty acid chain present is 18:2 (ω-6), and therefore we cannot conclude the identity of this lipid. Although various studies have shown that B_12_ deficiency results in adverse lipid profile, as well as pathophysiological changes linked to CAD, type2 diabetes mellitus, and atherosclerosis, very few studies have independently investigated the effect of vitamin B_12_ status on changes in human plasma at lipid species level among apparently healthy population [67,68,69]. In previously published reports, long chain of TAG (less than 50 and more than 50 carbons) has been found to be associated with coronary artery disease [60,70]. Importantly, the lipid species that are altered because of the vitamin B_12_ deficiency are still not yet well understood. 

To our knowledge, this is the first study to identify lipids with a significantly decreased ω-3 fatty acid (20:5) chains and increased ω-6 (18:2) chains, which might alter/increased ω-6 to ω-3 fatty acid ratio in human plasma in relation to vitamin B_12_ deficiency and may promote development of various chronic diseases. Most importantly we found that although there was no significant alteration in the lipid classes, individual lipid species varied in vitamin B_12_ deficient individuals clearly demonstrating the utility of identifying lipid species.

The application of *scheduled* MRM with variable-RTW and relative-DTW enabled large-scale relative quantification of lipid species in a single-run as compared to unscheduled/scheduled/dynamic MRM. With this combinatorial approach, we were able to detect more than 1000 lipid species in plasma, including isomers of TAG and PL’s. It should be noted that the MRMs currently used are specific for plasma and may not be ideal for other biological systems. Therefore, developing a separate MRM panel may be required for other type of studies. To the best of our knowledge this is the largest number of lipid species identified till date in a single experiment. A comprehensive identification of structural isomers in large-scale lipid method proves to be critical for studying the important biological functions of lipids.

## Figures and Tables

**Table 1:** Analytical validation of the method with lipid standards.

Lipid Class	Ion Mode	Number of Lipid Species in MRM Library	Internal Standard	Q1	Q3	Retention Time	DP	EP	CE	CxP	LoD Conc. (pmol/L)	LoQ Conc. (pmol/L)	Coefficient of Determination (R^2^)
SM	ESI+	12	SM (d18:1–18:1(d9))	738.7	184.2	11.89	80	10	43	15	0.319	0.639	0.99
CE	ESI+	21	Ceramide (17:0)	552.5	264.3	2.58	80	10	43	15	6.082	12.164	0.99
Cer	ESI+	62											
TAG	ESI+	445	TAG (15:0–18:1(d7)–15:0)	829.4	570.5	2.39	80	10	38	15	17.233	34.466	0.99
DAG	ESI+	50	DAG (15:0–18:1(d7))	605.5	346.3	2.46	80	10	25	15	999.184	1998.367	0.99
MAG	ESI+	17											
LPC	ESI−	16	LPC (18:1(d7))	587.4	288.3	12.63	−80	−10	−50	−15	0.368	5.887	0.99
PC	ESI−	79	PC (15:0–18:1(d7))	811.6	288.3	9.73	−80	−10	−50	−15	13.024	26.048	0.98
LPE	ESI−	16	LPE (18:1(d7))	485.3	288.3	13.09	−80	−10	−50	−15	1.329	5.318	0.99
PE	ESI−	142	PE (15:0–18:1(d7))	709.6	288.3	10.61	−80	−10	−50	−15	0.245	0.979	0.99
LPG	ESI−	16	PG (15:0–18:1(d7))	740.5	288.3	6.59	−80	−10	−50	−15	0.291	0.291	0.99
PG	ESI−	78											
LPI	ESI−	16	PI (15:0–18:1(d7))	828.6	288.3	13.09	−80	−10	−50	−15	2.639	10.557	0.98
PI	ESI−	77											
LPS	ESI−	16	PS (15:0–18:1(d7))	753.5	288.3	9.78	−80	−10	−50	−15	41.961	167.846	0.99
PS	ESI−	78											
PA	ESI−	77	PA (15:0–18:1(d7))	666.5	288.3	11.85	−80	−10	−50	−15	41.897	167.587	0.97

## Data Availability

All data supporting the finding of this study are contained in the manuscript and supplementary. All of the primary raw data are available from the corresponding author.

## Supplementary Materials

The following supporting information can be downloaded at: https://www.mdpi.com/article/10.3390/biom12050709/s1. Figure S1: Total ion chromatograms of the scheduled MRM method using variable-RTW and relative-DTW; Figure S2: Visual inspection of SM-XIC for peak assignment through MRM-EPI; Table S1: Optimized MRM parameters for 1083 transitions with detailed CV(n = 5, technical replicates) table for 3 different days; Table S2: The reproducibility of retention time and % CV for RT of 1083 lipid species for 3 different days. Table S3: TAG’s species with their respective isomers; Table S4: PL’s species with their respective isomers; Table S5: The list of the samples with their vitamin B12 values, age and sex; Table S6: Spike and recovery; Table S7: Fold change and p-value of different lipid classes in vitamin B12 deficient study; Table S8: DE selected lipids.
