# Supplementary material for: A High Throughput Lipidomics Method Using Scheduled Multiple Reaction Monitoring
Source: Biomolecules. 2022 May 16;12(5):709. doi: 10.3390/biom12050709 (PMC9138805; doi:10.3390/biom12050709)
Supplement: Supplementary file 1 [file biomolecules-12-00709-s001.zip › biomolecules-1728266-Supplementary.pdf]

Supplementary Figure and Table:

Supplementary Figure S1. Total ion chromatograms of the *scheduled* MRM method using variable-RTW and relative-DTW.

Supplementary Figure S2. Visual inspection of SM-XIC for peak assignment through MRM-EPI (a) The chromatogram of internal standard d18:1-18:1(d9) SM showing the RT (11.81 min) in positive polarity. **(b)** The spectral mapping of d18:1-18:1(d9) SM fragmented peaks with chemical structure showing the presence of following peaks at : 103.84 (choline), 124.92, 166.28, 184.18 (phosphocholine head group) and 738.80 (precursor ion). **(c)** Chromatogram of 15:0-18:1(d7) PC showing the RT (10.26 min) negative polarity (relative RT was subjected to variation with buffer and column condition). **(d)** The spectral mapping of 15:0-18:1(d7) PC fragmented peaks with chemical structure showing the presence of following peaks at : 153.25 (Glycerol-3-phosphate ion with loss of H2O), 168.21 (Phosphocholine with loss of CH3), 224.29 (Glycerophosphocholine with loss of CH3 and H2O), 241.37 (FA 15:0 RCOO- ion), 288.45 (FA 18:1(d7) RCOO- ion), 448.60 (Neutral loss of 18:1(d7) RCOOH group, loss of CH3 and acetate from precursor ion), 466.60 (Loss of 18:1(d7) acyl chain as ketene (RCH=C=O), CH3 and acetate from precursor ion), 495.73 (Neutral loss of 15:0 RCOOH group, loss of CH3 and acetate from precursor ion), 513.75 (Loss of 15:0 acyl chain as ketene (RCH=C=O), CH3 and acetate from precursor ion), 666.95 (Loss of choline and acetate from precursor ion), 737.86 (Loss of CH3 and acetate from precursor ion) and 811.88 (Precursor ion [M+acetate]-).

Supplementary- Table S1 Optimized MRM parameters for 1083 transitions with detailed CV(n=5, technical replicates) table for 3 different days.

Supplementary- Table S2 The reproducibility of retention time and % CV for RT of 1083lipid species for 3 different days.

Supplementary- Table S3 TAG's species with their respective isomers.

Supplementary- Table S4 PL's species with their respective isomers.

Supplementary-Table S5 The list of the samples with their vitamin B_{12} values, age and sex.

Supplementary Table S6 Spike and recovery.

Supplementary Table S7 Fold change and p-value of different lipid classes in vitamin B_{12} deficient study.

Supplementary Table S8 DE selected lipids.

a. Total ion chromatogram

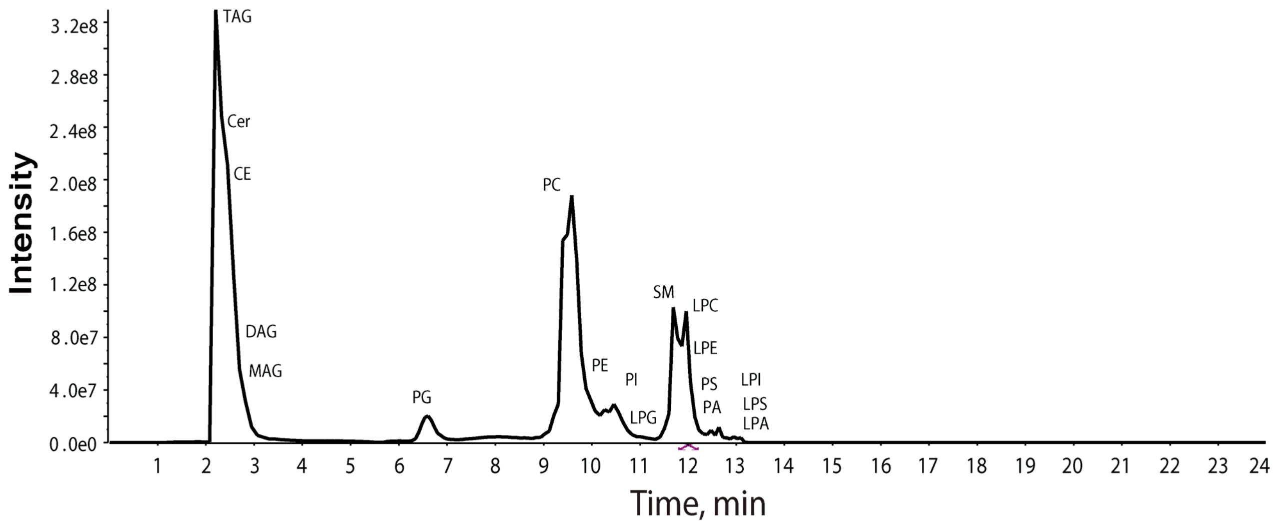

Supplementary Figure S1

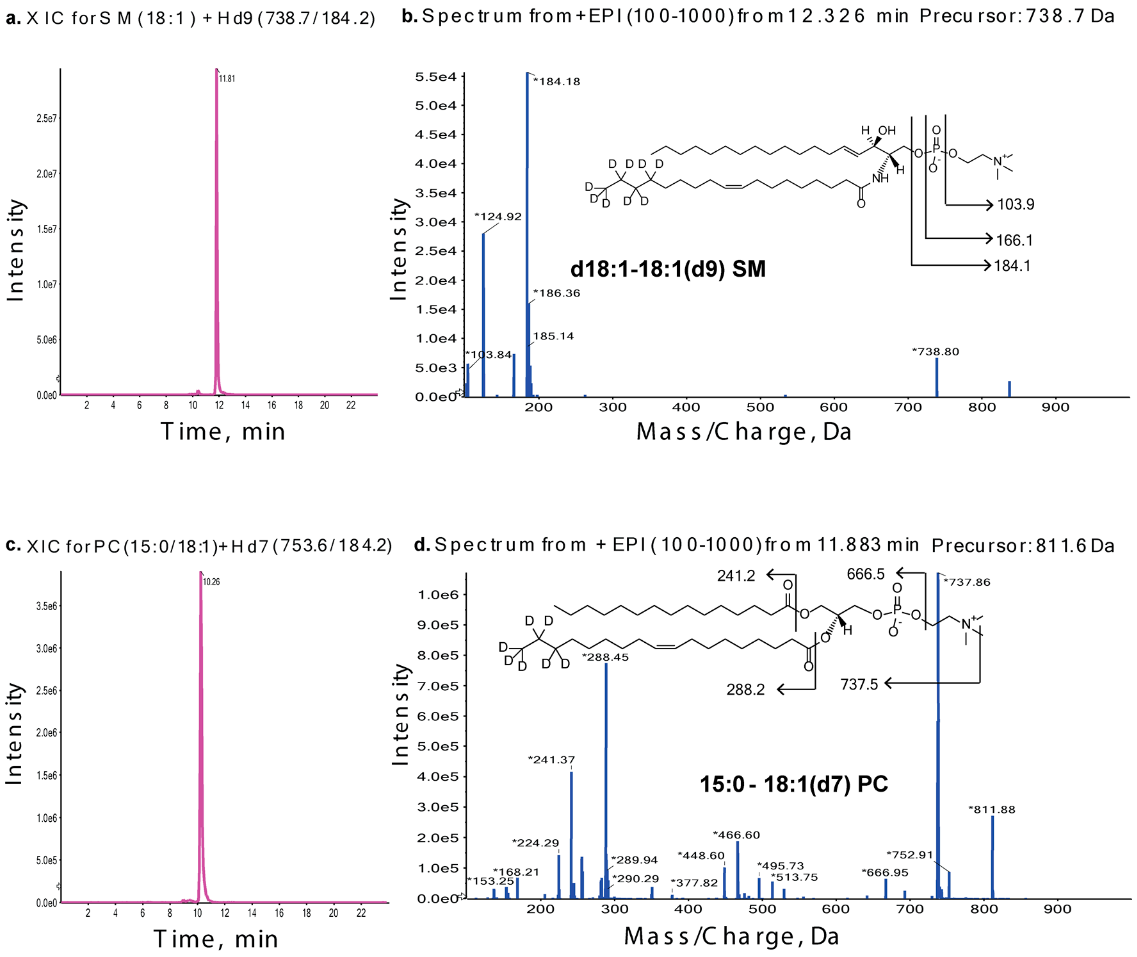

Supplementary Figure S2

Supplementary- Table S1: Optimized MRM parameters for 1083 transitions with detailed CV(n=5, technical replicates) and data points for 3 different days.

S.No.	Q1	Q3	Dwell time weight	ID	DP	EP	CE	CXP	Day1 CV (Data point)	Day2 CV (Data point)	Day3 CV (Data point)
1	675.5	184.1	1	SM(14:0)+H	80	10	43	15	2.53 (19)	3.93 (17)	3.94 (19)
2	703.6	184.1	1	SM(16:0)+H	80	10	43	15	4.9 (21)	2.93 (21)	3.6 (23)
3	731.6	184.1	1	SM(18:0)+H	80	10	43	15	5.01 (20)	1.23 (20)	1.75 (21)
4	729.6	184.1	1	SM(18:1)+H	80	10	43	15	2.95 (20)	1.83 (19)	2.51 (21)
5	759.6	184.1	1	SM(20:0)+H	80	10	43	15	0.85 (19)	4.01 (20)	4.7 (21)
6	757.6	184.1	1	SM(20:1)+H	80	10	43	15	2.85 (18)	2.55 (18)	4.56 (20)
7	787.7	184.1	1	SM(22:0)+H	80	10	43	15	4.39 (22)	3.39 (21)	5.25 (23)
8	785.7	184.1	1	SM(22:1)+H	80	10	43	15	3.12 (22)	2.05 (20)	4.33 (23)
9	815.7	184.1	1	SM(24:0)+H	80	10	43	15	5.69 (26)	2.9 (24)	3.9 (27)
10	813.7	184.1	1	SM(24:1)+H	80	10	43	15	2.19 (28)	2.19 (24)	3.36 (28)
11	843.7	184.1	1	SM(26:0)+H	80	10	43	15	19.09 (19)	2.6 (18)	3.31 (19)
12	841.7	184.1	1	SM(26:1)+H	80	10	25	15	2.2 (29)	4.92 (24)	3.88 (33)
13	754.7	369.4	1	CE(24:0)+NH4	80	10	25	15	8.67 (11)	9.46 (11)	8.7 (12)
14	714.6	369.4	1	CE(22:6)+NH4	80	10	25	15	13.12 (11)	7.41 (12)	9.04 (12)
15	698.7	369.4	1	CE(20:0)+NH4	80	10	25	15	10.94 (12)	10.65 (13)	7.92 (13)
16	696.7	369.4	1	CE(20:1)+NH4	80	10	25	15	8.09 (15)	13.05 (13)	11.46 (13)
17	716.6	369.4	1	CE(22:5)+NH4	80	10	25	15	26.01 (12)	14.85 (13)	12.23 (12)
18	614.6	369.4	1	CE(14:0)+NH4	80	10	25	15	17.25 (11)	5.19 (12)	13.64 (13)
19	642.6	369.4	1	CE(16:0)+NH4	80	10	25	15	25.7 (11)	21.21 (21)	20.99 (15)
20	640.6	369.4	1	CE(16:1)+NH4	80	10	25	15	20.79 (12)	22.23 (15)	15.98 (15)
21	670.6	369.4	1	CE(18:0)+NH4	80	10	25	15	22.52 (10)	11.85 (11)	4.87 (12)
22	668.6	369.4	1	CE(18:1)+NH4	80	10	25	15	13.64 (19)	9.19 (21)	6.35 (23)
23	666.6	369.4	1	CE(18:2)+NH4	80	10	25	15	11.53 (21)	10.12 (15)	13.03 (20)
24	664.6	369.4	1	CE(18:3)+NH4	80	10	25	15	8.45 (13)	7.71 (11)	12.13 (13)
25	694.6	369.4	1	CE(20:2)+NH4	80	10	25	15	13.07 (13)	16.15 (15)	17.04 (14)

26	692.6	369.4	1	CE(20:3)+NH4	80	10	25	15	10.71 (12)	2.45 (13)	4.33 (14)
27	690.6	369.4	1	CE(20:4)+NH4	80	10	25	15	13.24 (17)	9.37 (18)	9.48 (18)
28	688.6	369.4	1	CE(20:5)+NH4	80	10	25	15	11.75 (12)	8.92 (13)	11.98 (13)
29	726.7	369.4	1	CE(22:0)+NH4	80	10	25	15	19.83 (11)	14.56 (13)	10.79 (13)
30	724.7	369.4	1	CE(22:1)+NH4	80	10	25	15	22.1 (13)	17.62 (14)	26.25 (11)
31	722.7	369.4	1	CE(22:2)+NH4	80	10	25	15	17.8 (12)	11.61 (12)	17.03 (12)
32	718.6	369.4	1	CE(22:4)+NH4	80	10	25	15	23.65 (12)	5.74 (11)	5.98 (12)
33	752.7	369.4	1	CE(24:1)+NH4	80	10	43	15	13.76 (21)	12.1 (15)	14.12 (18)
34	510.6	264.4	1.02	CER(14:0)+H	80	10	43	15	45.78 (11)	30.12 (9)	47.06 (18)
35	538.6	264.4	1	CER(16:0)+H	80	10	43	15	16.95 (9)	8.86 (9)	17.91 (9)
36	566.7	264.4	1	CER(18:0)+H	80	10	43	15	15.19 (9)	22.28 (8)	15.55 (9)
37	564.8	264.4	1	CER(18:1)+H	80	10	43	15	9.16 (9)	23.51 (9)	12.56 (9)
38	594.6	264.4	1	CER(20:0)+H	80	10	43	15	14.42 (8)	29.24 (7)	10.69 (8)
39	592.6	264.4	1	CER(20:1)+H	80	10	43	15	17.05 (8)	16.79 (8)	18.31 (7)
40	622.7	264.4	1	CER(22:0)+H	80	10	43	15	19.04 (10)	11.96 (7)	9.41 (10)
41	620.7	264.4	1	CER(22:1)+H	80	10	43	15	7.72 (9)	7.67 (14)	4.17 (11)
42	650.8	264.4	1	CER(24:0)+H	80	10	43	15	12.93 (10)	14.03 (8)	13.83 (8)
43	648.8	264.4	1	CER(24:1)+H	80	10	43	15	5.94 (9)	9.61 (8)	3.18 (10)
44	678.9	264.4	1	CER(26:0)+H	80	10	43	15	10.36 (11)	15.2 (11)	16.4 (10)
45	676.9	264.4	1	CER(26:1)+H	80	10	43	15	4.45 (9)	6.35 (13)	3.02 (9)
46	512.6	266.4	1.04	DCER(14:0)+H	80	10	43	15	26.11 (13)	24.24 (13)	14.05 (12)
47	540.6	266.4	1	DCER(16:0)+H	80	10	43	15	13.69 (9)	22.52 (9)	9.31 (10)
48	568.7	266.4	1	DCER(18:0)+H	80	10	43	15	7.64 (11)	25.7 (11)	6.35 (10)
49	566.8	266.4	1.05	DCER(18:1)+H	80	10	43	15	22.57 (14)	29.4 (14)	22.42 (14)
50	596.7	266.4	1	DCER(20:0)+H	80	10	43	15	22.24 (9)	18.17 (10)	7.02 (9)
51	594.4	266.4	1.04	DCER(20:1)+H	80	10	43	15	26.06 (10)	32.07 (12)	22.17 (12)
52	624.8	266.4	1	DCER(22:0)+H	80	10	43	15	7.4 (11)	8.09 (14)	6.75 (11)
53	620.4	266.4	1	DCER(22:1)+H	80	10	43	15	5.91 (11)	8.17 (11)	7.45 (10)
54	652.9	266.4	1	DCER(24:0)+H	80	10	43	15	2.61 (10)	5.24 (11)	4.81 (12)

55	650.9	266.4	1	DCER(24:1)+H	80	10	43	15	6.87 (10)	4.34 (11)	6.71 (10)
56	680.5	266.4	1.01	DCER(26:0)+H	80	10	43	15	11.09 (11)	9.36 (11)	14.02 (10)
57	672.5	264.4	1	HCER(14:0)+H	80	10	43	15	13.36 (30)	13.26 (26)	5.78 (43)
58	700.7	264.4	1	HCER(16:0)+H	80	10	43	15	5.17 (36)	7.96 (38)	37.74 (22)
59	728.8	264.4	1	HCER(18:0)+H	80	10	43	15	2.78 (33)	7.41 (37)	12.51 (33)
60	726.7	264.4	1	HCER(18:1)+H	80	10	43	15	18.08 (25)	16.43 (22)	4.59 (25)
61	756.7	264.4	1	HCER(20:0)+H	80	10	43	15	7.4 (35)	10.92 (38)	5.21 (36)
62	754.7	264.4	1	HCER(20:1)+H	80	10	43	15	6.57 (39)	9.19 (38)	7.48 (31)
63	784.9	264.4	1	HCER(22:0)+H	80	10	43	15	4.65 (38)	9.58 (39)	6.2 (35)
64	782.8	264.4	1	HCER(22:1)+H	80	10	43	15	3.66 (49)	6.71 (48)	6.28 (37)
65	812.9	264.4	1	HCER(24:0)+H	80	10	43	15	7.05 (40)	7.23 (45)	2.74 (36)
66	810.9	264.4	1	HCER(24:1)+H	80	10	43	15	17.03 (37)	5.66 (41)	5.07 (45)
67	840.9	264.4	1	HCER(26:0)+H	80	10	43	15	#DIV/0!	#DIV/0!	8.41 (34)
68	838.9	264.4	1	HCER(26:1)+H	80	10	43	15	14.79 (30)	15.33 (31)	3.77 (40)
69	730.8	266.4	1.06	HCER(d18:0/18:0)+H	80	10	43	15	26.18 (34)	31.25 (25)	10.04 (49)
70	758.7	266.4	1.06	HCER(d18:0/20:0)+H	80	10	43	15	25.76 (32)	23.54 (25)	10.6 (38)
71	786.9	266.4	1.02	HCER(d18:0/22:0)+H	80	10	43	15	10.1 (38)	8.76 (42)	6.83 (40)
72	814.9	266.4	1.01	HCER(d18:0/24:0)+H	80	10	43	15	7 (34)	9.54 (36)	11.1 (28)
73	812.9	266.4	1.01	HCER(d18:0/24:1)+H	80	10	43	15	3.71 (47)	9.65 (56)	5.63 (54)
74	842.9	266.4	1.06	HCER(d18:0/26:0)+H	80	10	43	15	#DIV/0!	#DIV/0!	9.58 (40)
75	840.9	266.4	1.1	HCER(d18:0/26:1)+H	80	10	43	15	#DIV/0!	#DIV/0!	7.85 (36)
76	834.9	264.4	1.02	LCER(14:0)+H	80	10	43	15	19.94 (14)	13.93 (18)	29.11 (10)
77	862.9	264.4	1.02	LCER(16:0)+H	80	10	43	15	14.43 (16)	4.53 (17)	6 (15)
78	890.2	264.4	1.01	LCER(18:0)+H	80	10	43	15	13.08 (13)	10.3 (11)	9.96 (11)
79	888.2	264.4	1	LCER(18:1)+H	80	10	43	15	8.2 (12)	15.82 (12)	10.2 (15)
80	918.2	264.4	1.02	LCER(20:0)+H	80	10	43	15	6.59 (14)	6.29 (11)	8.76 (14)
81	916.2	264.4	1.01	LCER(20:1)+H	80	10	43	15	11.45 (19)	12.92 (13)	11.92 (13)
82	946.2	264.4	1.02	LCER(22:0)+H	80	10	43	15	13.39 (13)	15.67 (15)	12.95 (14)
83	944.2	264.4	1.01	LCER(22:1)+H	80	10	43	15	16.15 (14)	15.92 (32)	20.9 (14)

84	974.8	264.4	1.01	LCER(24:0)+H	80	10	43	15	32.9 (18)	26.81 (17)	14.95 (17)
85	972.9	264.4	1.01	LCER(24:1)+H	80	10	38	15	26.41 (12)	10.61 (17)	29.55 (18)
86	1002.4	264.4	1.03	LCER(26:0)+H	80	10	43	15	15.35 (10)	29.61 (19)	31.31 (16)
87	1000.4	264.4	1.05	LCER(26:1)+H	80	10	43	15	18.04 (12)	24.58 (17)	32.18 (12)
88	892.7	266.4	1.02	LCER(d18:0/18:0)+H	80	10	43	15	8.15 (16)	18.69 (16)	19.8 (16)
89	920.7	266.4	1.02	LCER(d18:0/20:0)+H	80	10	43	15	30.77 (19)	20.78 (18)	30.91 (13)
90	948.7	266.4	1.03	LCER(d18:0/22:0)+H	80	10	43	15	31.74 (12)	35.05 (18)	10.18 (17)
91	976.8	266.4	1.03	LCER(d18:0/24:0)+H	80	10	43	15	47.98 (15)	23.53 (19)	32.36 (16)
92	974.7	266.4	1.04	LCER(d18:0/24:1)+H	80	10	43	15	32.83 (14)	17.52 (18)	27.9 (15)
93	1004.9	266.4	1.04	LCER(d18:0/26:0)+H	80	10	43	15	4.21 (12)	36.33 (18)	47.14 (18)
94	1002.9	266.4	1.05	LCER(d18:0/26:1)+H	80	10	43	15	25.35 (15)	32.33 (19)	8.88 (19)
95	712.645	467.409	1	TAG(40:0/FA14:0)+NH4	80	10	38	15	6.7 (9)	25.74 (12)	15.89 (12)
96	712.645	439.378	1	TAG(40:0/FA16:0)+NH4	80	10	38	15	#DIV/0!	18.79 (14)	13 (20)
97	740.676	495.441	1	TAG(42:0/FA14:0)+NH4	80	10	38	15	#DIV/0!	16.9 (11)	28.24 (11)
98	740.676	467.409	1	TAG(42:0/FA16:0)+NH4	80	10	38	15	#DIV/0!	21.33 (12)	9.29 (11)
99	738.661	493.425	1	TAG(42:1/FA14:0)+NH4	80	10	38	15	28.02 (9)	22.79 (13)	11.65 (13)
100	738.661	465.394	1	TAG(42:1/FA16:0)+NH4	80	10	38	15	5.78 (12)	8.34 (13)	15.15 (15)
101	738.661	439.378	1	TAG(42:1/FA18:1)+NH4	80	10	38	15	27.22 (13)	8.22 (13)	8.99 (12)
102	736.645	439.378	1	TAG(42:2/FA18:2)+NH4	80	10	38	15	14.88 (10)	24.35 (12)	17.46 (11)
103	768.708	523.472	1	TAG(44:0/FA14:0)+NH4	80	10	38	15	13.87 (11)	6.14 (16)	7.69 (14)
104	768.708	495.441	1	TAG(44:0/FA16:0)+NH4	80	10	38	15	8.39 (11)	7.54 (12)	10.05 (12)
105	768.708	467.409	1	TAG(44:0/FA18:0)+NH4	80	10	38	15	#DIV/0!	15.27 (12)	12.32 (10)
106	766.692	521.456	1	TAG(44:1/FA14:0)+NH4	80	10	38	15	6.67 (11)	10.85 (12)	14.37 (11)
107	766.692	493.425	1	TAG(44:1/FA16:0)+NH4	80	10	38	15	9.72 (11)	5.78 (11)	10.33 (12)
108	766.692	495.441	1	TAG(44:1/FA16:1)+NH4	80	10	38	15	5.19 (12)	15.98 (13)	18.92 (14)
109	766.692	467.409	1	TAG(44:1/FA18:1)+NH4	80	10	38	15	6.79 (11)	4.88 (13)	15.2 (11)
110	764.676	519.441	1	TAG(44:2/FA14:0)+NH4	80	10	38	15	11.58 (12)	19.16 (12)	18.03 (11)
111	764.676	491.409	1	TAG(44:2/FA16:0)+NH4	80	10	38	15	7.56 (12)	8.7 (11)	13.52 (11)
112	764.676	493.425	1	TAG(44:2/FA16:1)+NH4	80	10	38	15	13.19 (13)	15.16 (13)	27.64 (14)

113	764.676	465.394	1	TAG(44:2/FA18:1)+NH4	80	10	38	15	17.95 (12)	6.52 (13)	3.46 (12)
114	764.676	467.409	1	TAG(44:2/FA18:2)+NH4	80	10	38	15	17.12 (15)	14.16 (13)	4.52 (13)
115	762.661	465.394	1	TAG(44:3/FA18:2)+NH4	80	10	38	15	17.3 (11)	24.71 (12)	25.02 (11)
116	782.723	537.488	1	TAG(45:0/FA14:0)+NH4	80	10	38	15	10.02 (11)	11.3 (13)	13.01 (12)
117	782.723	509.456	1	TAG(45:0/FA16:0)+NH4	80	10	38	15	10.12 (11)	10.62 (12)	6.05 (11)
118	780.708	507.4	1	TAG(45:1/FA16:0)+NH4	80	10	38	15	15.11 (13)	6.19 (13)	8.5 (14)
119	780.708	481.4	1	TAG(45:1/FA18:1)+NH4	80	10	38	15	9.92 (12)	11.47 (13)	8.46 (11)
120	796.7	551.503	1	TAG(46:0/FA14:0)+NH4	80	10	38	15	6.92 (17)	8.38 (19)	4.39 (17)
121	796.7	523.472	1	TAG(46:0/FA16:0)+NH4	80	10	38	15	5.58 (15)	2.37 (16)	4.05 (20)
122	796.7	495.441	1	TAG(46:0/FA18:0)+NH4	80	10	38	15	17.11 (12)	11.29 (14)	23.74 (14)
123	794.7	549.5	1	TAG(46:1/FA14:0)+NH4	80	10	38	15	6.73 (12)	5.06 (13)	7.3 (12)
124	794.7	521.4	1	TAG(46:1/FA16:0)+NH4	80	10	38	15	2.99 (14)	5.77 (14)	2.65 (14)
125	794.7	523.472	1	TAG(46:1/FA16:1)+NH4	80	10	38	15	6.83 (12)	5.26 (13)	7.78 (12)
126	794.7	493.425	1	TAG(46:1/FA18:0)+NH4	80	10	38	15	18.63 (11)	7.32 (11)	11.87 (12)
127	794.7	495.441	1	TAG(46:1/FA18:1)+NH4	80	10	38	15	6 (13)	8.94 (13)	6.8 (15)
128	792.7	547.5	1	TAG(46:2/FA14:0)+NH4	80	10	38	15	7.43 (12)	9 (13)	11.62 (13)
129	792.7	519.441	1	TAG(46:2/FA16:0)+NH4	80	10	38	15	2.4 (11)	9.99 (11)	4.04 (11)
130	792.7	521.4	1	TAG(46:2/FA16:1)+NH4	80	10	38	15	3.14 (12)	6.7 (13)	4.23 (12)
131	792.7	493.425	1	TAG(46:2/FA18:1)+NH4	80	10	38	15	2.39 (10)	5.32 (12)	5.3 (11)
132	792.7	495.441	1	TAG(46:2/FA18:2)+NH4	80	10	38	15	3.86 (13)	4.34 (12)	9.83 (13)
133	790.7	545.5	1.01	TAG(46:3/FA14:0)+NH4	80	10	38	15	12.26 (15)	20.25 (17)	13.77 (17)
134	790.7	517.4	1	TAG(46:3/FA16:0)+NH4	80	10	38	15	14.95 (12)	9.36 (11)	8.31 (14)
135	790.7	519.441	1	TAG(46:3/FA16:1)+NH4	80	10	38	15	4.82 (11)	12.42 (12)	19.47 (12)
136	790.7	491.409	1	TAG(46:3/FA18:1)+NH4	80	10	38	15	17.28 (12)	6.83 (14)	8.23 (13)
137	790.7	493.425	1	TAG(46:3/FA18:2)+NH4	80	10	38	15	6.26 (11)	9.23 (11)	19.95 (12)
138	790.7	495.441	1	TAG(46:3/FA18:3)+NH4	80	10	38	15	6.84 (9)	19.32 (11)	19.92 (12)
139	788.7	491.409	1	TAG(46:4/FA18:2)+NH4	80	10	38	15	25.19 (10)	10.11 (14)	20.52 (12)
140	810.7	565.5	1	TAG(47:0/FA14:0)+NH4	80	10	38	15	7.93 (13)	7.15 (16)	13.81 (16)
141	810.7	537.4	1	TAG(47:0/FA16:0)+NH4	80	10	38	15	3.32 (13)	4.66 (14)	10.49 (18)

142	810.7	523.472	1	TAG(47:0/FA17:0)+NH4	80	10	38	15	14.57 (11)	6.35 (15)	11.51 (13)
143	808.7	563.5	1	TAG(47:1/FA14:0)+NH4	80	10	38	15	6.69 (12)	5.44 (16)	3.46 (21)
144	808.7	535.4	1	TAG(47:1/FA16:0)+NH4	80	10	38	15	8.91 (11)	3.43 (13)	9.05 (12)
145	808.7	537.4	1	TAG(47:1/FA16:1)+NH4	80	10	38	15	6.02 (12)	8.08 (11)	11.54 (11)
146	808.7	521.4	1	TAG(47:1/FA17:0)+NH4	80	10	38	15	9.72 (12)	15.19 (13)	14.52 (11)
147	808.7	509.4	1	TAG(47:1/FA18:1)+NH4	80	10	38	15	10 (11)	6.93 (12)	7.89 (11)
148	806.7	561.5	1	TAG(47:2/FA14:0)+NH4	80	10	38	15	5.12 (12)	14.3 (14)	12.64 (12)
149	806.7	535.4	1	TAG(47:2/FA16:1)+NH4	80	10	38	15	16.9 (12)	5.4 (12)	39.4 (10)
150	806.7	507.4	1	TAG(47:2/FA18:1)+NH4	80	10	38	15	5.79 (13)	7.3 (11)	9.24 (11)
151	806.7	509.4	1	TAG(47:2/FA18:2)+NH4	80	10	38	15	12.27 (13)	7.13 (12)	9 (13)
152	824.7	579.5	1	TAG(48:0/FA14:0)+NH4	80	10	38	15	7.25 (18)	6.88 (19)	8.96 (18)
153	824.7	551.4	1	TAG(48:0/FA16:0)+NH4	80	10	38	15	2.23 (25)	6.58 (26)	3.04 (24)
154	824.7	523.472	1	TAG(48:0/FA18:0)+NH4	80	10	38	15	4.06 (18)	4.62 (24)	4.03 (23)
155	822.7	577.5	1	TAG(48:1/FA14:0)+NH4	80	10	38	15	2.11 (17)	4.58 (18)	3.6 (18)
156	822.7	549.4	1	TAG(48:1/FA16:0)+NH4	80	10	38	15	1.16 (14)	5.53 (20)	4.16 (18)
157	822.7	551.4	1	TAG(48:1/FA16:1)+NH4	80	10	38	15	4.73 (21)	7.66 (21)	3.21 (18)
158	822.7	521.4	1	TAG(48:1/FA18:0)+NH4	80	10	38	15	4.67 (17)	7.45 (16)	6.85 (16)
159	822.7	523.472	1	TAG(48:1/FA18:1)+NH4	80	10	38	15	4.62 (24)	2.2 (23)	2.77 (23)
160	820.7	575.5	1	TAG(48:2/FA14:0)+NH4	80	10	38	15	3.82 (13)	2.96 (13)	2.96 (14)
161	820.7	547.4	1	TAG(48:2/FA16:0)+NH4	80	10	38	15	2.6 (13)	4.83 (14)	4.01 (14)
162	820.7	549.4	1	TAG(48:2/FA16:1)+NH4	80	10	38	15	3.66 (13)	4.36 (14)	3.82 (13)
163	820.7	519.441	1	TAG(48:2/FA18:0)+NH4	80	10	38	15	8.44 (13)	16.67 (15)	14.07 (15)
164	820.7	521.4	1	TAG(48:2/FA18:1)+NH4	80	10	38	15	3.17 (13)	4.16 (13)	3.12 (14)
165	820.7	523.472	1	TAG(48:2/FA18:2)+NH4	80	10	38	15	6.18 (14)	7.01 (14)	6.27 (13)
166	818.7	573.5	1	TAG(48:3/FA14:0)+NH4	80	10	38	15	3.94 (13)	3.29 (13)	4.9 (13)
167	818.7	545.4	1	TAG(48:3/FA16:0)+NH4	80	10	38	15	6.03 (12)	7.87 (14)	7.36 (13)
168	818.7	547.4	1	TAG(48:3/FA16:1)+NH4	80	10	38	15	8.38 (12)	5.21 (12)	7.84 (14)
169	818.7	519.441	1	TAG(48:3/FA18:1)+NH4	80	10	38	15	7.44 (13)	4.02 (13)	4.74 (12)
170	818.7	521.4	1	TAG(48:3/FA18:2)+NH4	80	10	38	15	4.46 (12)	12.49 (13)	6.56 (13)

171	818.7	523.472	1	TAG(48:3/FA18:3)+NH4	80	10	38	15	4.93 (13)	4.35 (12)	7.64 (13)
172	816.7	571.5	1.02	TAG(48:4/FA14:0)+NH4	80	10	38	15	14.01 (14)	15.5 (18)	18.19 (19)
173	816.7	543.4	1.01	TAG(48:4/FA16:0)+NH4	80	10	38	15	10.06 (14)	8.52 (13)	10.01 (15)
174	816.7	545.4	1.01	TAG(48:4/FA16:1)+NH4	80	10	38	15	12.71 (12)	3.07 (13)	9.66 (14)
175	816.7	517.4	1	TAG(48:4/FA18:1)+NH4	80	10	38	15	7.21 (12)	10.09 (12)	10.29 (11)
176	816.7	519.441	1	TAG(48:4/FA18:2)+NH4	80	10	38	15	5.72 (14)	16.54 (14)	7.48 (14)
177	816.7	521.4	1	TAG(48:4/FA18:3)+NH4	80	10	38	15	11.45 (14)	12.23 (13)	14.24 (13)
178	816.7	495.441	1.02	TAG(48:4/FA20:4)+NH4	80	10	38	15	8.87 (14)	10.07 (16)	29.47 (16)
179	814.7	517.4	1.01	TAG(48:5/FA18:2)+NH4	80	10	38	15	4.55 (11)	16.31 (12)	21.09 (12)
180	814.7	519.441	1	TAG(48:5/FA18:3)+NH4	80	10	38	15	12.34 (18)	15.18 (15)	31.68 (19)
181	838.8	565.5	1	TAG(49:0/FA16:0)+NH4	80	10	38	15	8.73 (29)	6.25 (19)	3.14 (27)
182	838.8	551.503	1	TAG(49:0/FA17:0)+NH4	80	10	38	15	5.71 (21)	4.37 (25)	5.93 (22)
183	838.8	537.5	1	TAG(49:0/FA18:0)+NH4	80	10	38	15	16.25 (19)	10.77 (17)	6.24 (18)
184	836.8	591.6	1	TAG(49:1/FA14:0)+NH4	80	10	38	15	11.6 (16)	7.65 (15)	12.16 (17)
185	836.8	563.5	1	TAG(49:1/FA16:0)+NH4	80	10	38	15	2.86 (19)	2.8 (15)	4.58 (27)
186	836.8	565.5	1	TAG(49:1/FA16:1)+NH4	80	10	38	15	5.93 (15)	7.2 (17)	7.28 (16)
187	836.8	549.5	1	TAG(49:1/FA17:0)+NH4	80	10	38	15	6.93 (20)	4.21 (18)	6.49 (16)
188	836.8	537.5	1	TAG(49:1/FA18:1)+NH4	80	10	38	15	8.66 (21)	3.92 (15)	5.85 (26)
189	834.8	589.6	1	TAG(49:2/FA14:0)+NH4	80	10	38	15	10.22 (12)	7.66 (13)	10.13 (13)
190	834.8	561.5	1	TAG(49:2/FA16:0)+NH4	80	10	38	15	9.94 (13)	3.36 (14)	4.46 (13)
191	834.8	563.5	1	TAG(49:2/FA16:1)+NH4	80	10	38	15	4.77 (12)	3.29 (13)	6.66 (14)
192	834.8	547.5	1	TAG(49:2/FA17:0)+NH4	80	10	38	15	7.46 (14)	8.1 (14)	8.07 (13)
193	834.8	535.5	1	TAG(49:2/FA18:1)+NH4	80	10	38	15	6.2 (13)	5.1 (13)	6.01 (13)
194	834.8	537.5	1	TAG(49:2/FA18:2)+NH4	80	10	38	15	8.65 (14)	6.4 (15)	7.6 (14)
195	832.8	559.5	1	TAG(49:3/FA16:0)+NH4	80	10	38	15	6.72 (12)	5.85 (14)	3.61 (14)
196	832.8	561.5	1	TAG(49:3/FA16:1)+NH4	80	10	38	15	8.9 (13)	7.19 (12)	7.87 (13)
197	832.8	535.5	1	TAG(49:3/FA18:2)+NH4	80	10	38	15	8.56 (12)	6.88 (13)	10.33 (13)
198	832.8	537.5	1	TAG(49:3/FA18:3)+NH4	80	10	38	15	7.3 (14)	11.13 (13)	13.13 (14)
199	852.8	607.6	1	TAG(50:0/FA14:0)+NH4	80	10	38	15	9.38 (21)	6.17 (23)	7.42 (23)

200	852.8	579.5	1	TAG(50:0/FA16:0)+NH4	80	10	38	15	3.18 (12)	3.77 (31)	22.64 (11)
201	852.8	551.503	1	TAG(50:0/FA18:0)+NH4	80	10	38	15	5.22 (12)	7.6 (28)	19.11 (26)
202	850.8	605.6	1	TAG(50:1/FA14:0)+NH4	80	10	38	15	7.66 (29)	5.71 (30)	7.38 (25)
203	850.8	577.5	1	TAG(50:1/FA16:0)+NH4	80	10	38	15	6.89 (25)	2.22 (25)	21.82 (21)
204	850.8	579.5	1	TAG(50:1/FA16:1)+NH4	80	10	38	15	3.74 (24)	5.57 (21)	25.13 (17)
205	850.8	549.5	1	TAG(50:1/FA18:0)+NH4	80	10	38	15	4.76 (21)	5.02 (25)	23.85 (13)
206	850.8	551.503	1	TAG(50:1/FA18:1)+NH4	80	10	38	15	3.89 (24)	5.04 (24)	4.2 (23)
207	850.8	523.472	1	TAG(50:1/FA20:1)+NH4	80	10	38	15	10.68 (12)	5.62 (12)	8.39 (12)
208	848.8	603.6	1	TAG(50:2/FA14:0)+NH4	80	10	38	15	6.25 (14)	6.65 (17)	25.83 (12)
209	848.8	575.5	1	TAG(50:2/FA16:0)+NH4	80	10	38	15	3.44 (20)	4.94 (23)	1.35 (22)
210	848.8	577.5	1	TAG(50:2/FA16:1)+NH4	80	10	38	15	5.66 (19)	3.49 (17)	5.29 (18)
211	848.8	547.5	1	TAG(50:2/FA18:0)+NH4	80	10	38	15	3.63 (15)	3.5 (18)	21.47 (15)
212	848.8	549.5	1	TAG(50:2/FA18:1)+NH4	80	10	38	15	4.28 (21)	3.34 (17)	3.37 (18)
213	848.8	551.503	1	TAG(50:2/FA18:2)+NH4	80	10	38	15	4.19 (28)	4.55 (23)	4.32 (27)
214	848.8	523.472	1	TAG(50:2/FA20:2)+NH4	80	10	38	15	6.07 (18)	11.19 (17)	6.42 (18)
215	846.8	601.6	1	TAG(50:3/FA14:0)+NH4	80	10	38	15	4.48 (13)	5.79 (13)	4.68 (14)
216	846.8	573.5	1	TAG(50:3/FA16:0)+NH4	80	10	38	15	1.88 (14)	3.9 (13)	7.22 (14)
217	846.8	575.5	1	TAG(50:3/FA16:1)+NH4	80	10	38	15	2.94 (14)	5.94 (13)	6.06 (13)
218	846.8	545.5	1	TAG(50:3/FA18:0)+NH4	80	10	38	15	4.63 (13)	10.95 (15)	7.65 (15)
219	846.8	547.5	1	TAG(50:3/FA18:1)+NH4	80	10	38	15	3.46 (13)	1.08 (13)	6.76 (14)
220	846.8	549.5	1	TAG(50:3/FA18:2)+NH4	80	10	38	15	0.6 (14)	2.34 (13)	3.54 (14)
221	846.8	551.503	1	TAG(50:3/FA18:3)+NH4	80	10	38	15	3.18 (13)	6.71 (14)	5.51 (13)
222	846.8	523.472	1	TAG(50:3/FA20:3)+NH4	80	10	38	15	5.34 (14)	3.61 (16)	7.84 (17)
223	844.6	599.4	1	TAG(50:4/FA14:0)+NH4	80	10	38	15	8.77 (13)	7.24 (13)	8.4 (13)
224	844.6	571.3	1	TAG(50:4/FA16:0)+NH4	80	10	38	15	9.21 (14)	3.8 (13)	5.67 (14)
225	844.6	573.3	1	TAG(50:4/FA16:1)+NH4	80	10	38	15	4.88 (12)	4.47 (13)	5.04 (13)
226	844.6	545.3	1	TAG(50:4/FA18:1)+NH4	80	10	38	15	7.65 (13)	4.6 (13)	9.62 (13)
227	844.6	547.3	1	TAG(50:4/FA18:2)+NH4	80	10	38	15	8.13 (13)	6.33 (12)	7.03 (14)
228	844.6	549.3	1	TAG(50:4/FA18:3)+NH4	80	10	38	15	6.51 (13)	3.7 (13)	5 (13)

229	844.6	521.3	1.01	TAG(50:4/FA20:3)+NH4	80	10	38	15	16.07 (17)	16.35 (16)	18.05 (18)
230	844.6	523.3	1	TAG(50:4/FA20:4)+NH4	80	10	38	15	5.52 (13)	10.68 (13)	7.95 (13)
231	842.6	597.4	1.02	TAG(50:5/FA14:0)+NH4	80	10	38	15	11.27 (18)	5.98 (15)	21.96 (18)
232	842.6	569.3	1	TAG(50:5/FA16:0)+NH4	80	10	38	15	9.12 (12)	9.55 (14)	10.68 (17)
233	842.6	571.3	1.01	TAG(50:5/FA16:1)+NH4	80	10	38	15	13.14 (12)	29.93 (12)	20.22 (12)
234	842.6	543.3	1	TAG(50:5/FA18:1)+NH4	80	10	38	15	12.38 (12)	17.87 (14)	17.46 (15)
235	842.6	545.3	1.01	TAG(50:5/FA18:2)+NH4	80	10	38	15	9.02 (13)	6.54 (14)	3.26 (14)
236	842.6	547.3	1	TAG(50:5/FA18:3)+NH4	80	10	38	15	4.34 (13)	5.26 (12)	4.56 (13)
237	842.6	521.3	1.01	TAG(50:5/FA20:4)+NH4	80	10	38	15	3.95 (14)	20.99 (18)	9.72 (17)
238	842.6	523.3	1	TAG(50:5/FA20:5)+NH4	80	10	38	15	16.27 (13)	12.78 (13)	20.68 (12)
239	840.7	519.441	1.02	TAG(50:6/FA20:4)+NH4	80	10	38	15	7.66 (13)	13.7 (16)	15.09 (18)
240	866.8	593.5	1	TAG(51:0/FA16:0)+NH4	80	10	38	15	6.24 (11)	9.24 (12)	7.01 (11)
241	866.8	579.5	1	TAG(51:0/FA17:0)+NH4	80	10	38	15	9.29 (12)	19.49 (12)	14.75 (26)
242	866.8	565.5	1	TAG(51:0/FA18:0)+NH4	80	10	38	15	9.45 (12)	15.96 (12)	29.32 (23)
243	864.8	591.5	1	TAG(51:1/FA16:0)+NH4	80	10	38	15	4.8 (22)	2.86 (24)	7.06 (22)
244	864.8	577.5	1	TAG(51:1/FA17:0)+NH4	80	10	38	15	3.07 (26)	2.11 (32)	3.51 (31)
245	864.8	563.5	1	TAG(51:1/FA18:0)+NH4	80	10	38	15	1.15 (29)	6.15 (18)	12.3 (28)
246	864.8	565.5	1	TAG(51:1/FA18:1)+NH4	80	10	38	15	6.37 (27)	4.78 (23)	26.85 (13)
247	862.8	589.5	1	TAG(51:2/FA16:0)+NH4	80	10	38	15	7.15 (13)	1.83 (13)	3.44 (13)
248	862.8	591.5	1	TAG(51:2/FA16:1)+NH4	80	10	38	15	10.87 (16)	7.62 (17)	4.38 (16)
249	862.8	575.5	1	TAG(51:2/FA17:0)+NH4	80	10	38	15	4.2 (19)	3.87 (17)	3.54 (18)
250	862.8	563.5	1	TAG(51:2/FA18:1)+NH4	80	10	38	15	1.37 (19)	3.8 (16)	21.66 (15)
251	862.8	565.5	1	TAG(51:2/FA18:2)+NH4	80	10	38	15	3.11 (24)	2.54 (26)	4.56 (25)
252	860.8	589.5	1	TAG(51:3/FA16:1)+NH4	80	10	38	15	5.79 (13)	7.3 (13)	6.26 (13)
253	860.8	573.5	1	TAG(51:3/FA17:0)+NH4	80	10	38	15	5.98 (14)	7.63 (17)	7.83 (17)
254	860.8	563.5	1	TAG(51:3/FA18:2)+NH4	80	10	38	15	2.22 (15)	4.96 (15)	5.64 (15)
255	860.8	565.5	1	TAG(51:3/FA18:3)+NH4	80	10	38	15	2.59 (12)	3.18 (13)	9.25 (13)
256	858.8	587.5	1	TAG(51:4/FA16:1)+NH4	80	10	38	15	7.87 (15)	3.31 (13)	10.77 (15)
257	858.8	561.5	1	TAG(51:4/FA18:2)+NH4	80	10	38	15	3.04 (14)	9.52 (13)	9.15 (13)

258	858.8	563.5	1	TAG(51:4/FA18:3)+NH4	80	10	38	15	6.28 (13)	7.91 (13)	2.11 (14)
259	858.8	537.5	1.02	TAG(51:4/FA20:4)+NH4	80	10	38	15	8.76 (17)	20.21 (15)	9.39 (16)
260	856.8	559.5	1.01	TAG(51:5/FA18:2)+NH4	80	10	38	15	9.7 (14)	12.87 (15)	6.11 (14)
261	856.8	561.5	1	TAG(51:5/FA18:3)+NH4	80	10	38	15	9.02 (13)	14.28 (13)	10.28 (13)
262	880.8	607.5	1	TAG(52:0/FA16:0)+NH4	80	10	38	15	6.16 (12)	23.7 (12)	16.7 (12)
263	880.8	579.5	1	TAG(52:0/FA18:0)+NH4	80	10	38	15	16.34 (11)	11.96 (11)	28.87 (11)
264	880.8	551.503	1	TAG(52:0/FA20:0)+NH4	80	10	38	15	8.32 (10)	10.06 (12)	25.23 (9)
265	878.8	605.5	1	TAG(52:1/FA16:0)+NH4	80	10	38	15	5.43 (13)	10.84 (11)	4.76 (35)
266	878.8	607.5	1	TAG(52:1/FA16:1)+NH4	80	10	38	15	5.07 (26)	6.23 (25)	15.58 (16)
267	878.8	577.5	1	TAG(52:1/FA18:0)+NH4	80	10	38	15	3.77 (31)	4.58 (31)	1.37 (30)
268	878.8	579.5	1	TAG(52:1/FA18:1)+NH4	80	10	38	15	2.22 (33)	4.12 (32)	2.45 (33)
269	878.8	549.5	1	TAG(52:1/FA20:0)+NH4	80	10	38	15	10.97 (11)	9.52 (11)	6.09 (11)
270	878.8	551.503	1	TAG(52:1/FA20:1)+NH4	80	10	38	15	6.5 (10)	16.22 (10)	38.37 (10)
271	876.8	631.6	1	TAG(52:2/FA14:0)+NH4	80	10	38	15	8.96 (11)	5.31 (12)	14.78 (12)
272	876.8	603.5	1	TAG(52:2/FA16:0)+NH4	80	10	38	15	4.48 (26)	5.36 (31)	1.39 (25)
273	876.8	605.5	1	TAG(52:2/FA16:1)+NH4	80	10	38	15	4.26 (24)	4.35 (24)	18.76 (17)
274	876.8	575.5	1	TAG(52:2/FA18:0)+NH4	80	10	38	15	3.18 (31)	2.58 (29)	3.18 (30)
275	876.8	577.5	1	TAG(52:2/FA18:1)+NH4	80	10	38	15	1.27 (31)	4.8 (30)	5.19 (20)
276	876.8	579.5	1	TAG(52:2/FA18:2)+NH4	80	10	38	15	2.39 (27)	1.96 (27)	3.15 (20)
277	876.8	547.5	1	TAG(52:2/FA20:0)+NH4	80	10	38	15	9.83 (15)	10.55 (12)	14.81 (15)
278	876.8	549.5	1	TAG(52:2/FA20:1)+NH4	80	10	38	15	2.87 (11)	7.56 (13)	3.29 (12)
279	876.8	551.503	1	TAG(52:2/FA20:2)+NH4	80	10	38	15	3.35 (22)	4.3 (23)	4.47 (24)
280	874.8	629.6	1	TAG(52:3/FA14:0)+NH4	80	10	38	15	9.01 (13)	10.89 (12)	8.67 (13)
281	874.8	601.5	1	TAG(52:3/FA16:0)+NH4	80	10	38	15	4.05 (21)	1.32 (22)	5.4 (21)
282	874.8	603.5	1	TAG(52:3/FA16:1)+NH4	80	10	38	15	4.34 (18)	3.59 (18)	3.64 (18)
283	874.8	573.5	1	TAG(52:3/FA18:0)+NH4	80	10	38	15	6.05 (19)	3.37 (19)	3.31 (17)
284	874.8	575.5	1	TAG(52:3/FA18:1)+NH4	80	10	38	15	2.95 (20)	2.48 (19)	2.66 (21)
285	874.8	577.5	1	TAG(52:3/FA18:2)+NH4	80	10	38	15	0.58 (20)	2.52 (22)	2.69 (20)
286	874.8	579.5	1	TAG(52:3/FA18:3)+NH4	80	10	38	15	5.16 (13)	7.24 (13)	3.19 (14)

287	874.8	545.5	1	TAG(52:3/FA20:0)+NH4	80	10	38	15	4.93 (22)	8.27 (19)	5.2 (18)
288	874.8	547.5	1	TAG(52:3/FA20:1)+NH4	80	10	38	15	1 (12)	9.59 (13)	5.63 (14)
289	874.8	549.5	1	TAG(52:3/FA20:2)+NH4	80	10	38	15	4.43 (18)	6.63 (16)	8.3 (18)
290	874.8	551.503	1	TAG(52:3/FA20:3)+NH4	80	10	38	15	5.1 (15)	3.77 (18)	5.57 (16)
291	872.8	627.6	1.01	TAG(52:4/FA14:0)+NH4	80	10	38	15	3.27 (14)	11.21 (13)	8.18 (14)
292	872.8	599.5	1	TAG(52:4/FA16:0)+NH4	80	10	38	15	3.79 (14)	3.66 (14)	4.05 (14)
293	872.8	601.5	1	TAG(52:4/FA16:1)+NH4	80	10	38	15	3.47 (14)	3.26 (13)	3.74 (15)
294	872.8	571.5	1	TAG(52:4/FA18:0)+NH4	80	10	38	15	6.84 (13)	10.3 (14)	10.88 (13)
295	872.8	573.5	1	TAG(52:4/FA18:1)+NH4	80	10	38	15	4.47 (13)	4.23 (14)	3.53 (15)
296	872.8	575.5	1	TAG(52:4/FA18:2)+NH4	80	10	38	15	5.56 (14)	3.43 (14)	4.68 (14)
297	872.8	577.5	1	TAG(52:4/FA18:3)+NH4	80	10	38	15	0.81 (13)	4.1 (13)	1.88 (14)
298	872.8	543.5	1.01	TAG(52:4/FA20:0)+NH4	80	10	38	15	4.93 (15)	13.3 (12)	26.17 (8)
299	872.8	547.5	1	TAG(52:4/FA20:2)+NH4	80	10	38	15	5.9 (17)	5.15 (15)	7.64 (15)
300	872.8	549.5	1	TAG(52:4/FA20:3)+NH4	80	10	38	15	4.93 (18)	5.99 (17)	5.8 (17)
301	872.8	551.503	1	TAG(52:4/FA20:4)+NH4	80	10	38	15	4.98 (18)	4.03 (17)	2.79 (19)
302	872.8	523.472	1	TAG(52:4/FA22:4)+NH4	80	10	38	15	9.11 (12)	6.11 (12)	5.86 (11)
303	870.8	625.6	1.02	TAG(52:5/FA14:0)+NH4	80	10	38	15	10.03 (13)	7.06 (13)	10.45 (12)
304	870.8	597.5	1	TAG(52:5/FA16:0)+NH4	80	10	38	15	5.69 (14)	5.79 (13)	4.34 (13)
305	870.8	599.5	1	TAG(52:5/FA16:1)+NH4	80	10	38	15	4.93 (12)	7 (12)	3.91 (13)
306	870.8	571.5	1	TAG(52:5/FA18:1)+NH4	80	10	38	15	3.08 (14)	4.65 (14)	5.4 (14)
307	870.8	573.5	1	TAG(52:5/FA18:2)+NH4	80	10	38	15	3.84 (13)	6.51 (13)	5.91 (14)
308	870.8	575.5	1	TAG(52:5/FA18:3)+NH4	80	10	38	15	3.3 (13)	5.02 (12)	3.81 (13)
309	870.8	547.5	1.01	TAG(52:5/FA20:3)+NH4	80	10	38	15	4.77 (16)	17 (16)	14.06 (17)
310	870.8	549.5	1	TAG(52:5/FA20:4)+NH4	80	10	38	15	2.49 (14)	4.4 (14)	4.69 (14)
311	870.8	551.503	1	TAG(52:5/FA20:5)+NH4	80	10	38	15	3.94 (14)	10.17 (15)	3.43 (13)
312	870.8	523.472	1	TAG(52:5/FA22:5)+NH4	80	10	38	15	6.72 (13)	4.76 (15)	26.23 (12)
313	868.8	623.6	1.02	TAG(52:6/FA14:0)+NH4	80	10	38	15	6.09 (14)	3.15 (15)	12.44 (17)
314	868.8	595.5	1	TAG(52:6/FA16:0)+NH4	80	10	38	15	6.41 (12)	8.89 (15)	17.08 (13)
315	868.8	597.5	1.01	TAG(52:6/FA16:1)+NH4	80	10	38	15	6.06 (12)	11.29 (12)	5.25 (13)

316	868.8	569.5	1	TAG(52:6/FA18:1)+NH4	80	10	38	15	5.19 (12)	5.99 (12)	12.37 (12)
317	868.8	571.5	1.01	TAG(52:6/FA18:2)+NH4	80	10	38	15	5.3 (11)	8.48 (11)	4.95 (12)
318	868.8	573.5	1	TAG(52:6/FA18:3)+NH4	80	10	38	15	7.17 (12)	7.1 (12)	5.99 (12)
319	868.8	547.5	1.01	TAG(52:6/FA20:4)+NH4	80	10	38	15	6 (12)	11.8 (10)	13.66 (11)
320	868.8	549.5	1	TAG(52:6/FA20:5)+NH4	80	10	38	15	4.8 (13)	9.15 (12)	4.09 (11)
321	868.8	523.472	1	TAG(52:6/FA22:6)+NH4	80	10	38	15	5.94 (13)	9.53 (11)	8.4 (14)
322	866.7	593.4	1	TAG(52:7/FA16:0)+NH4	80	10	38	15	5.1 (11)	4.14 (11)	6.05 (12)
323	866.7	567.4	1	TAG(52:7/FA18:1)+NH4	80	10	38	15	9.97 (25)	11.5 (16)	6.12 (28)
324	866.7	547.4	1	TAG(52:7/FA20:5)+NH4	80	10	38	15	10.39 (13)	6.72 (15)	13.2 (14)
325	866.7	521.4	1	TAG(52:7/FA22:6)+NH4	80	10	38	15	17.21 (14)	7.06 (14)	5.79 (14)
326	864.8	593.5	1	TAG(52:8/FA16:1)+NH4	80	10	38	15	15.7 (12)	9.77 (11)	21.78 (10)
327	864.8	567.5	1.01	TAG(52:8/FA18:2)+NH4	80	10	38	15	7.27 (14)	10.77 (12)	5.54 (13)
328	894.8	621.5	1	TAG(53:0/FA16:0)+NH4	80	10	38	15	5.7 (11)	3.35 (11)	7.01 (11)
329	892.8	619.5	1	TAG(53:1/FA16:0)+NH4	80	10	38	15	7.62 (11)	5.43 (11)	2.57 (12)
330	892.8	605.5	1	TAG(53:1/FA17:0)+NH4	80	10	38	15	5.01 (13)	6.33 (11)	14.56 (30)
331	892.8	591.5	1	TAG(53:1/FA18:0)+NH4	80	10	38	15	10.07 (13)	3.25 (13)	21.42 (23)
332	892.8	593.5	1	TAG(53:1/FA18:1)+NH4	80	10	38	15	7.96 (10)	10.09 (11)	6.43 (11)
333	890.8	617.5	1	TAG(53:2/FA16:0)+NH4	80	10	38	15	7.5 (11)	1.67 (11)	7.11 (11)
334	890.8	603.5	1	TAG(53:2/FA17:0)+NH4	80	10	38	15	2.54 (27)	5.7 (28)	4.18 (26)
335	890.8	591.5	1	TAG(53:2/FA18:1)+NH4	80	10	38	15	9.67 (10)	6.82 (11)	33.39 (11)
336	890.8	593.5	1	TAG(53:2/FA18:2)+NH4	80	10	38	15	7.4 (11)	2.66 (11)	4.65 (11)
337	888.8	615.5	1	TAG(53:3/FA16:0)+NH4	80	10	38	15	6.8 (12)	5.41 (12)	4.25 (11)
338	888.8	601.5	1	TAG(53:3/FA17:0)+NH4	80	10	38	15	3.33 (21)	5.1 (21)	4.39 (20)
339	888.8	591.5	1	TAG(53:3/FA18:2)+NH4	80	10	38	15	7.01 (12)	7.14 (12)	5.72 (11)
340	886.8	613.5	1	TAG(53:4/FA16:0)+NH4	80	10	38	15	3.08 (12)	5.48 (11)	12.13 (12)
341	886.8	599.5	1	TAG(53:4/FA17:0)+NH4	80	10	38	15	5.09 (13)	6.4 (13)	6.3 (15)
342	886.8	589.5	1	TAG(53:4/FA18:2)+NH4	80	10	38	15	5.61 (13)	8.01 (13)	4.36 (14)
343	886.8	591.5	1	TAG(53:4/FA18:3)+NH4	80	10	38	15	5.19 (13)	2.4 (13)	9.99 (13)
344	886.8	565.5	1.01	TAG(53:4/FA20:4)+NH4	80	10	38	15	5.99 (16)	5.57 (18)	11.96 (17)

345	884.8	563.5	1.01	TAG(53:5/FA20:4)+NH4	80	10	38	15	5.96 (15)	8.14 (18)	10.5 (20)
346	882.8	561.5	1.01	TAG(53:6/FA20:4)+NH4	80	10	38	15	3.21 (11)	13.25 (11)	13.7 (11)
347	908.8	635.5	1	TAG(54:0/FA16:0)+NH4	80	10	38	15	6.19 (10)	3.23 (34)	59.59 (20)
348	908.8	607.5	1	TAG(54:0/FA18:0)+NH4	80	10	38	15	10.52 (10)	3.09 (35)	70.37 (20)
349	906.8	633.5	1	TAG(54:1/FA16:0)+NH4	80	10	38	15	8.19 (10)	5.92 (20)	23.17 (9)
350	906.8	605.5	1	TAG(54:1/FA18:0)+NH4	80	10	38	15	5.3 (10)	3.01 (10)	16.77 (10)
351	906.8	607.5	1	TAG(54:1/FA18:1)+NH4	80	10	38	15	8.5 (10)	8.13 (11)	44.77 (16)
352	906.8	577.5	1	TAG(54:1/FA20:0)+NH4	80	10	38	15	14.83 (10)	7.07 (11)	7.76 (11)
353	906.8	579.5	1	TAG(54:1/FA20:1)+NH4	80	10	38	15	22.43 (10)	13.29 (10)	27.62 (10)
354	904.8	631.5	1	TAG(54:2/FA16:0)+NH4	80	10	38	15	11.56 (10)	11.19 (11)	21.23 (10)
355	904.8	603.5	1	TAG(54:2/FA18:0)+NH4	80	10	38	15	2.5 (31)	4.78 (29)	2.11 (29)
356	904.8	605.5	1	TAG(54:2/FA18:1)+NH4	80	10	38	15	2.91 (30)	3.82 (30)	23.04 (11)
357	904.8	607.5	1	TAG(54:2/FA18:2)+NH4	80	10	38	15	2.55 (30)	1.29 (26)	3.29 (25)
358	904.8	575.5	1	TAG(54:2/FA20:0)+NH4	80	10	38	15	6.88 (12)	11.52 (11)	10.67 (12)
359	904.8	577.5	1	TAG(54:2/FA20:1)+NH4	80	10	38	15	7.12 (10)	8.04 (11)	6.71 (12)
360	904.8	579.5	1	TAG(54:2/FA20:2)+NH4	80	10	38	15	5.35 (23)	4.4 (23)	20.83 (19)
361	902.8	629.5	1	TAG(54:3/FA16:0)+NH4	80	10	38	15	8.84 (13)	7.57 (12)	5.81 (13)
362	902.8	631.5	1	TAG(54:3/FA16:1)+NH4	80	10	38	15	6.4 (12)	10.32 (12)	4.58 (13)
363	902.8	601.5	1	TAG(54:3/FA18:0)+NH4	80	10	38	15	3.27 (30)	3.25 (25)	3.06 (25)
364	902.8	603.5	1	TAG(54:3/FA18:1)+NH4	80	10	38	15	3.82 (31)	3.2 (29)	2.44 (28)
365	902.8	605.5	1	TAG(54:3/FA18:2)+NH4	80	10	38	15	3.39 (22)	3.01 (22)	4.69 (21)
366	902.8	607.5	1	TAG(54:3/FA18:3)+NH4	80	10	38	15	5.38 (15)	4.73 (14)	11.99 (14)
367	902.8	575.5	1	TAG(54:3/FA20:1)+NH4	80	10	38	15	5.68 (12)	8.35 (12)	5.24 (12)
368	902.8	577.5	1	TAG(54:3/FA20:2)+NH4	80	10	38	15	6.4 (16)	5.1 (16)	2.03 (17)
369	902.8	579.5	1	TAG(54:3/FA20:3)+NH4	80	10	38	15	1.47 (21)	2.17 (21)	23.52 (16)
370	900.8	627.5	1	TAG(54:4/FA16:0)+NH4	80	10	38	15	3.85 (15)	4.21 (16)	5.78 (16)
371	900.8	629.5	1	TAG(54:4/FA16:1)+NH4	80	10	38	15	5.8 (15)	8.37 (13)	6.56 (14)
372	900.8	599.5	1	TAG(54:4/FA18:0)+NH4	80	10	38	15	2.98 (17)	2.07 (18)	29.97 (12)
373	900.8	601.5	1	TAG(54:4/FA18:1)+NH4	80	10	38	15	4.87 (19)	5.7 (18)	5.42 (19)

374	900.8	603.5	1	TAG(54:4/FA18:2)+NH4	80	10	38	15	2.82 (13)	3.89 (12)	4.61 (13)
375	900.8	605.5	1	TAG(54:4/FA18:3)+NH4	80	10	38	15	1.18 (12)	6.66 (12)	4.03 (14)
376	900.8	573.5	1	TAG(54:4/FA20:1)+NH4	80	10	38	15	3.7 (14)	5.27 (14)	6.34 (14)
377	900.8	575.5	1	TAG(54:4/FA20:2)+NH4	80	10	38	15	3.46 (15)	9.74 (14)	4.12 (15)
378	900.8	577.5	1	TAG(54:4/FA20:3)+NH4	80	10	38	15	4.63 (22)	6.39 (21)	2.11 (21)
379	900.8	579.5	1	TAG(54:4/FA20:4)+NH4	80	10	38	15	4.43 (17)	4.64 (21)	3.56 (17)
380	900.8	551.503	1	TAG(54:4/FA22:4)+NH4	80	10	38	15	4.01 (13)	6.77 (16)	6.2 (19)
381	898.8	625.5	1	TAG(54:5/FA16:0)+NH4	80	10	38	15	4.02 (14)	4.12 (13)	5.57 (15)
382	898.8	627.5	1	TAG(54:5/FA16:1)+NH4	80	10	38	15	8.54 (13)	9.5 (13)	3.73 (13)
383	898.8	597.5	1	TAG(54:5/FA18:0)+NH4	80	10	38	15	3.5 (12)	17.23 (12)	3.7 (12)
384	898.8	599.5	1	TAG(54:5/FA18:1)+NH4	80	10	38	15	4.92 (12)	3.74 (13)	6.37 (13)
385	898.8	601.5	1	TAG(54:5/FA18:2)+NH4	80	10	38	15	3.79 (13)	7.6 (12)	5.75 (13)
386	898.8	603.5	1	TAG(54:5/FA18:3)+NH4	80	10	38	15	2.67 (13)	4.3 (12)	7.45 (13)
387	898.8	573.5	1.01	TAG(54:5/FA20:2)+NH4	80	10	38	15	6.78 (12)	10.97 (13)	9.5 (13)
388	898.8	575.5	1	TAG(54:5/FA20:3)+NH4	80	10	38	15	1.66 (12)	3.44 (14)	6.39 (13)
389	898.8	577.5	1	TAG(54:5/FA20:4)+NH4	80	10	38	15	1.25 (15)	5.92 (16)	3.18 (17)
390	898.8	579.5	1	TAG(54:5/FA20:5)+NH4	80	10	38	15	5.9 (15)	8.31 (14)	6.35 (15)
391	898.8	549.5	1	TAG(54:5/FA22:4)+NH4	80	10	38	15	3.01 (14)	9.42 (14)	3.04 (16)
392	898.8	551.503	1	TAG(54:5/FA22:5)+NH4	80	10	38	15	5.97 (12)	3.8 (15)	5.91 (13)
393	896.8	623.5	1	TAG(54:6/FA16:0)+NH4	80	10	38	15	4.63 (13)	4.12 (13)	5.4 (13)
394	896.8	625.5	1.01	TAG(54:6/FA16:1)+NH4	80	10	38	15	5.39 (13)	5.89 (14)	6.97 (13)
395	896.8	597.5	1	TAG(54:6/FA18:1)+NH4	80	10	38	15	3.41 (13)	5.32 (13)	7.12 (14)
396	896.8	599.5	1	TAG(54:6/FA18:2)+NH4	80	10	38	15	3.09 (12)	8.06 (12)	6.75 (18)
397	896.8	601.5	1	TAG(54:6/FA18:3)+NH4	80	10	38	15	5.05 (13)	3.09 (13)	5.3 (13)
398	896.8	573.5	1.01	TAG(54:6/FA20:3)+NH4	80	10	38	15	8.57 (14)	9.48 (12)	11.03 (15)
399	896.8	575.5	1	TAG(54:6/FA20:4)+NH4	80	10	38	15	1.8 (13)	3.07 (14)	1.64 (14)
400	896.8	577.5	1	TAG(54:6/FA20:5)+NH4	80	10	38	15	4.6 (14)	2.77 (15)	5.02 (16)
401	896.8	549.5	1	TAG(54:6/FA22:5)+NH4	80	10	38	15	3.16 (12)	8.71 (15)	23.87 (9)
402	896.8	551.503	1	TAG(54:6/FA22:6)+NH4	80	10	38	15	2.87 (12)	7.69 (13)	4.43 (14)

403	894.8	623.5	1.01	TAG(54:7/FA16:1)+NH4	80	10	38	15	6.03 (15)	7.29 (16)	8.41 (23)
404	894.8	595.5	1	TAG(54:7/FA18:1)+NH4	80	10	38	15	6.37 (14)	12.48 (14)	6.04 (15)
405	894.8	597.5	1	TAG(54:7/FA18:2)+NH4	80	10	38	15	2.03 (13)	7.41 (16)	3.49 (30)
406	894.8	599.5	1	TAG(54:7/FA18:3)+NH4	80	10	38	15	1.21 (12)	13.49 (11)	3.97 (11)
407	894.8	573.5	1.01	TAG(54:7/FA20:4)+NH4	80	10	38	15	3.04 (12)	9.3 (13)	6.37 (13)
408	894.8	575.5	1	TAG(54:7/FA20:5)+NH4	80	10	38	15	4.44 (13)	3.26 (13)	4.21 (13)
409	894.8	547.5	1	TAG(54:7/FA22:5)+NH4	80	10	38	15	6.96 (13)	9.94 (13)	6.02 (16)
410	894.8	549.5	1	TAG(54:7/FA22:6)+NH4	80	10	38	15	5.98 (12)	4.45 (12)	7.64 (13)
411	892.8	595.5	1.02	TAG(54:8/FA18:2)+NH4	80	10	38	15	5.23 (13)	9.56 (14)	11.88 (15)
412	892.8	597.5	1.02	TAG(54:8/FA18:3)+NH4	80	10	38	15	3.23 (13)	6 (11)	9.97 (13)
413	892.8	571.5	1.04	TAG(54:8/FA20:4)+NH4	80	10	38	15	4.4 (19)	8.28 (19)	13.86 (22)
414	892.8	573.5	1	TAG(54:8/FA20:5)+NH4	80	10	38	15	4.73 (14)	17.47 (14)	9.55 (13)
415	892.8	547.5	1	TAG(54:8/FA22:6)+NH4	80	10	38	15	6.7 (15)	10.59 (13)	9.08 (15)
416	920.9	647.6	1	TAG(55:1/FA16:0)+NH4	80	10	38	15	7.97 (11)	5.53 (11)	5.26 (12)
417	920.9	621.6	1	TAG(55:1/FA18:1)+NH4	80	10	38	15	4.62 (12)	6.98 (11)	8.98 (12)
418	918.8	619.5	1	TAG(55:2/FA18:1)+NH4	80	10	38	15	7.63 (11)	8.13 (12)	6.02 (12)
419	918.8	621.5	1	TAG(55:2/FA18:2)+NH4	80	10	38	15	6.65 (17)	6.09 (14)	10.14 (12)
420	916.8	617.5	1	TAG(55:3/FA18:1)+NH4	80	10	38	15	5.46 (12)	5.33 (11)	5.51 (13)
421	916.8	619.5	1	TAG(55:3/FA18:2)+NH4	80	10	38	15	4.36 (12)	7.69 (12)	8.98 (12)
422	914.8	615.5	1	TAG(55:4/FA18:1)+NH4	80	10	38	15	6.9 (13)	6.74 (12)	6.15 (13)
423	914.8	617.5	1	TAG(55:4/FA18:2)+NH4	80	10	38	15	7.82 (12)	10.93 (13)	5.74 (13)
424	912.8	613.5	1	TAG(55:5/FA18:1)+NH4	80	10	38	15	10.51 (14)	7.68 (12)	8.02 (14)
425	912.8	615.5	1	TAG(55:5/FA18:2)+NH4	80	10	38	15	6.18 (12)	5.78 (12)	8.95 (13)
426	912.8	591.5	1	TAG(55:5/FA20:4)+NH4	80	10	38	15	4.8 (16)	12.51 (16)	5.95 (17)
427	908.8	563.5	1	TAG(55:7/FA22:6)+NH4	80	10	38	15	9.71 (13)	11.01 (13)	12.87 (14)
428	916.7	619.4	1	TAG(56:10/FA18:2)+NH4	80	10	38	15	7.89 (12)	14.7 (11)	11.03 (12)
429	934.9	661.6	1	TAG(56:1/FA16:0)+NH4	80	10	38	15	6.23 (10)	7.48 (10)	1.99 (55)
430	934.9	635.6	1	TAG(56:1/FA18:1)+NH4	80	10	38	15	7.48 (11)	12.35 (11)	17.53 (33)
431	932.9	659.6	1	TAG(56:2/FA16:0)+NH4	80	10	38	15	5.97 (12)	10.6 (12)	20.43 (32)

432	932.9	631.6	1	TAG(56:2/FA18:0)+NH4	80	10	38	15	11.31 (10)	13.06 (10)	74.81 (7)
433	932.9	603.6	1	TAG(56:2/FA20:0)+NH4	80	10	38	15	8.97 (11)	12.18 (11)	10.67 (11)
434	932.9	605.6	1	TAG(56:2/FA20:1)+NH4	80	10	38	15	9 (10)	8.52 (11)	8.96 (11)
435	930.8	657.5	1	TAG(56:3/FA16:0)+NH4	80	10	38	15	7.09 (13)	9.27 (13)	4.17 (30)
436	930.8	629.5	1	TAG(56:3/FA18:0)+NH4	80	10	38	15	5.4 (25)	4.38 (24)	4.26 (26)
437	930.8	631.5	1	TAG(56:3/FA18:1)+NH4	80	10	38	15	7.75 (10)	10.77 (10)	3.76 (11)
438	930.8	633.5	1	TAG(56:3/FA18:2)+NH4	80	10	38	15	11.72 (12)	8.18 (13)	2.75 (29)
439	930.8	601.5	1	TAG(56:3/FA20:0)+NH4	80	10	38	15	6.32 (12)	10.39 (12)	7.09 (12)
440	930.8	603.5	1	TAG(56:3/FA20:1)+NH4	80	10	38	15	11.56 (11)	9.81 (11)	41.78 (10)
441	930.8	605.5	1	TAG(56:3/FA20:2)+NH4	80	10	38	15	12.52 (11)	12.1 (11)	45 (9)
442	928.8	655.5	1	TAG(56:4/FA16:0)+NH4	80	10	38	15	6.63 (23)	5.09 (25)	5.03 (26)
443	928.8	627.5	1	TAG(56:4/FA18:0)+NH4	80	10	38	15	7 (24)	2.77 (23)	7.59 (25)
444	928.8	629.5	1	TAG(56:4/FA18:1)+NH4	80	10	38	15	10.68 (12)	11.51 (13)	11.15 (14)
445	928.8	631.5	1	TAG(56:4/FA18:2)+NH4	80	10	38	15	6.98 (13)	4.64 (13)	11.74 (12)
446	928.8	601.5	1	TAG(56:4/FA20:1)+NH4	80	10	38	15	9.71 (13)	7.11 (15)	10.74 (14)
447	928.8	603.5	1	TAG(56:4/FA20:2)+NH4	80	10	38	15	6.06 (23)	1.5 (22)	5.38 (22)
448	928.8	605.5	1	TAG(56:4/FA20:3)+NH4	80	10	38	15	4.25 (25)	5.26 (22)	5.77 (25)
449	928.8	607.5	1.02	TAG(56:4/FA20:4)+NH4	80	10	38	15	8.4 (29)	6.64 (28)	5.59 (22)
450	928.8	579.5	1	TAG(56:4/FA22:4)+NH4	80	10	38	15	2.3 (21)	8.09 (21)	10.5 (23)
451	926.8	653.5	1	TAG(56:5/FA16:0)+NH4	80	10	38	15	4.84 (21)	2.52 (22)	6.68 (21)
452	926.8	625.5	1	TAG(56:5/FA18:0)+NH4	80	10	38	15	3.59 (25)	7 (27)	6.08 (26)
453	926.8	627.5	1	TAG(56:5/FA18:1)+NH4	80	10	38	15	5.28 (14)	9.75 (15)	3.92 (15)
454	926.8	629.5	1	TAG(56:5/FA18:2)+NH4	80	10	38	15	4.44 (15)	4.83 (14)	7.12 (15)
455	926.8	599.5	1.01	TAG(56:5/FA20:1)+NH4	80	10	38	15	6.45 (15)	3.37 (15)	7.44 (16)
456	926.8	601.5	1	TAG(56:5/FA20:2)+NH4	80	10	38	15	4.82 (19)	6.55 (16)	4.4 (17)
457	926.8	603.5	1	TAG(56:5/FA20:3)+NH4	80	10	38	15	2.42 (20)	1.66 (18)	3.24 (19)
458	926.8	605.5	1	TAG(56:5/FA20:4)+NH4	80	10	38	15	6.59 (26)	3.77 (26)	5.43 (28)
459	926.8	577.5	1	TAG(56:5/FA22:4)+NH4	80	10	38	15	3.14 (20)	3.69 (22)	5.05 (21)
460	926.8	579.5	1	TAG(56:5/FA22:5)+NH4	80	10	38	15	10.06 (13)	5.25 (19)	1.89 (20)

461	924.8	651.5	1	TAG(56:6/FA16:0)+NH4	80	10	38	15	4.33 (20)	3.66 (21)	2.85 (21)
462	924.8	623.5	1	TAG(56:6/FA18:0)+NH4	80	10	38	15	3.79 (21)	9.57 (37)	8.04 (25)
463	924.8	625.5	1	TAG(56:6/FA18:1)+NH4	80	10	38	15	4.22 (21)	3.56 (21)	5.92 (21)
464	924.8	627.5	1	TAG(56:6/FA18:2)+NH4	80	10	38	15	4.09 (15)	3.1 (16)	2.6 (16)
465	924.8	629.5	1	TAG(56:6/FA18:3)+NH4	80	10	38	15	8.04 (15)	2.42 (15)	5.89 (14)
466	924.8	599.5	1	TAG(56:6/FA20:2)+NH4	80	10	38	15	5.41 (15)	12.95 (16)	13.83 (16)
467	924.8	601.5	1	TAG(56:6/FA20:3)+NH4	80	10	38	15	5.22 (18)	3.36 (15)	2.96 (16)
468	924.8	603.5	1	TAG(56:6/FA20:4)+NH4	80	10	38	15	3.06 (19)	4.05 (17)	6.19 (18)
469	924.8	605.5	1	TAG(56:6/FA20:5)+NH4	80	10	38	15	3.94 (15)	3.18 (15)	6.25 (16)
470	924.8	575.5	1	TAG(56:6/FA22:4)+NH4	80	10	38	15	9.17 (12)	8.68 (13)	6.6 (13)
471	924.8	577.5	1	TAG(56:6/FA22:5)+NH4	80	10	38	15	2.68 (19)	2.99 (19)	3.64 (21)
472	924.8	579.5	1	TAG(56:6/FA22:6)+NH4	80	10	38	15	5.66 (12)	7.7 (13)	8.54 (13)
473	922.8	649.5	1	TAG(56:7/FA16:0)+NH4	80	10	38	15	5.38 (12)	10.18 (12)	5.77 (12)
474	922.8	651.5	1	TAG(56:7/FA16:1)+NH4	80	10	38	15	5.86 (11)	6.5 (11)	7.78 (12)
475	922.8	621.5	1	TAG(56:7/FA18:0)+NH4	80	10	38	15	8.61 (12)	8.7 (14)	14.8 (11)
476	922.8	623.5	1	TAG(56:7/FA18:1)+NH4	80	10	38	15	5.17 (23)	3.12 (19)	4.06 (18)
477	922.8	625.5	1.01	TAG(56:7/FA18:2)+NH4	80	10	38	15	3.3 (13)	6.55 (14)	6.74 (14)
478	922.8	627.5	1	TAG(56:7/FA18:3)+NH4	80	10	38	15	7.84 (15)	5.05 (16)	4.68 (15)
479	922.8	599.5	1.02	TAG(56:7/FA20:3)+NH4	80	10	38	15	4.2 (15)	10.02 (16)	4.54 (16)
480	922.8	601.5	1	TAG(56:7/FA20:4)+NH4	80	10	38	15	6.1 (13)	3.15 (13)	6.31 (13)
481	922.8	603.5	1	TAG(56:7/FA20:5)+NH4	80	10	38	15	5.83 (12)	5.58 (14)	6.84 (14)
482	922.8	573.5	1	TAG(56:7/FA22:4)+NH4	80	10	38	15	7.66 (11)	12.79 (12)	7.05 (12)
483	922.8	575.5	1	TAG(56:7/FA22:5)+NH4	80	10	38	15	5.82 (13)	2.4 (13)	3.79 (14)
484	922.8	577.5	1	TAG(56:7/FA22:6)+NH4	80	10	38	15	6.67 (14)	4.54 (13)	2.98 (13)
485	920.8	647.5	1	TAG(56:8/FA16:0)+NH4	80	10	38	15	4.53 (12)	9.8 (11)	8.44 (12)
486	920.8	649.5	1	TAG(56:8/FA16:1)+NH4	80	10	38	15	4.31 (11)	15.82 (12)	25.9 (22)
487	920.8	621.5	1	TAG(56:8/FA18:1)+NH4	80	10	38	15	9.94 (11)	11.47 (12)	8.2 (12)
488	920.8	623.5	1.02	TAG(56:8/FA18:2)+NH4	80	10	38	15	6.1 (15)	6.57 (17)	9.87 (14)
489	920.8	625.5	1.03	TAG(56:8/FA18:3)+NH4	80	10	38	15	6.47 (17)	9.77 (18)	9.99 (18)

490	920.8	599.5	1.01	TAG(56:8/FA20:4)+NH4	80	10	38	15	3.03 (14)	12.29 (14)	5.29 (14)
491	920.8	601.5	1	TAG(56:8/FA20:5)+NH4	80	10	38	15	3.07 (13)	2.31 (13)	4.57 (13)
492	920.8	573.5	1	TAG(56:8/FA22:5)+NH4	80	10	38	15	7.53 (12)	5.53 (13)	10.14 (12)
493	920.8	575.5	1	TAG(56:8/FA22:6)+NH4	80	10	38	15	3.72 (12)	5.8 (12)	5.85 (13)
494	918.8	623.5	1.04	TAG(56:9/FA18:3)+NH4	80	10	38	15	5.1 (16)	19.77 (19)	6.97 (23)
495	918.8	597.5	1.08	TAG(56:9/FA20:4)+NH4	80	10	38	15	1.53 (15)	7.88 (14)	7.78 (28)
496	918.8	599.5	1.01	TAG(56:9/FA20:5)+NH4	80	10	38	15	4.12 (13)	12.96 (12)	8.39 (12)
497	918.8	573.5	1	TAG(56:9/FA22:6)+NH4	80	10	38	15	5.69 (12)	1.95 (12)	5.56 (12)
498	928.7	583.4	1.02	TAG(57:10/FA22:6)+NH4	80	10	38	15	46.68 (7)	23.95 (12)	26.99 (12)
499	946.9	647.6	1	TAG(57:2/FA18:1)+NH4	80	10	38	15	6.39 (11)	12.03 (11)	35.32 (10)
500	944.9	647.6	1.01	TAG(57:3/FA18:2)+NH4	80	10	38	15	9.1 (12)	7.37 (12)	23.02 (11)
501	944.8	647.5	1	TAG(58:10/FA18:2)+NH4	80	10	38	15	12.78 (11)	9.95 (12)	20.4 (11)
502	944.8	623.5	1.08	TAG(58:10/FA20:4)+NH4	80	10	38	15	1.59 (26)	4.03 (16)	4.6 (22)
503	944.8	625.5	1.03	TAG(58:10/FA20:5)+NH4	80	10	38	15	7.92 (17)	14.38 (15)	10.43 (17)
504	944.8	597.5	1.04	TAG(58:10/FA22:5)+NH4	80	10	38	15	7.9 (14)	14.5 (15)	17.41 (16)
505	944.8	599.5	1	TAG(58:10/FA22:6)+NH4	80	10	38	15	7.48 (12)	6.54 (12)	7.27 (13)
506	960.9	661.6	1	TAG(58:2/FA18:1)+NH4	80	10	38	15	3.57 (36)	20.46 (11)	2.87 (37)
507	958.9	659.6	1	TAG(58:3/FA18:1)+NH4	80	10	38	15	4.43 (11)	8.67 (12)	21.36 (31)
508	954.9	655.6	1	TAG(58:5/FA18:1)+NH4	80	10	38	15	5.21 (23)	5.55 (19)	8.81 (21)
509	952.8	679.5	1	TAG(58:6/FA16:0)+NH4	80	10	38	15	13.52 (15)	9.4 (15)	6.69 (15)
510	952.8	651.5	1	TAG(58:6/FA18:0)+NH4	80	10	38	15	8.33 (24)	10.72 (29)	5.17 (22)
511	952.8	653.5	1	TAG(58:6/FA18:1)+NH4	80	10	38	15	3.99 (28)	5.12 (36)	4.39 (29)
512	952.8	631.5	1.01	TAG(58:6/FA20:4)+NH4	80	10	38	15	7.95 (21)	5.02 (20)	8.84 (19)
513	952.8	603.5	1	TAG(58:6/FA22:4)+NH4	80	10	38	15	8.47 (15)	9.66 (17)	10.85 (17)
514	952.8	605.5	1	TAG(58:6/FA22:5)+NH4	80	10	38	15	6.38 (18)	6.38 (17)	11.33 (17)
515	950.8	677.5	1	TAG(58:7/FA16:0)+NH4	80	10	38	15	5.15 (12)	13.09 (15)	8.48 (14)
516	950.8	649.5	1	TAG(58:7/FA18:0)+NH4	80	10	38	15	4.17 (12)	19.5 (12)	10.53 (13)
517	950.8	651.5	1	TAG(58:7/FA18:1)+NH4	80	10	38	15	4.37 (14)	6.22 (13)	9.27 (14)
518	950.8	653.5	1.02	TAG(58:7/FA18:2)+NH4	80	10	38	15	8.02 (14)	11.64 (13)	6.42 (13)

519	950.8	629.5	1	TAG(58:7/FA20:4)+NH4	80	10	38	15	7.79 (24)	11.81 (18)	12.99 (19)
520	950.8	601.5	1	TAG(58:7/FA22:4)+NH4	80	10	38	15	8.16 (12)	7.96 (13)	7.07 (13)
521	950.8	603.5	1	TAG(58:7/FA22:5)+NH4	80	10	38	15	8.27 (13)	9.28 (15)	4.98 (16)
522	950.8	605.5	1	TAG(58:7/FA22:6)+NH4	80	10	38	15	6.97 (13)	4.3 (13)	3.01 (14)
523	948.8	649.5	1	TAG(58:8/FA18:1)+NH4	80	10	38	15	2.75 (11)	9.57 (12)	2.21 (12)
524	948.8	651.5	1.01	TAG(58:8/FA18:2)+NH4	80	10	38	15	4.47 (24)	7.98 (21)	3.6 (21)
525	948.8	625.5	1.06	TAG(58:8/FA20:3)+NH4	80	10	38	15	5.24 (15)	6.98 (15)	15.82 (19)
526	948.8	627.5	1.02	TAG(58:8/FA20:4)+NH4	80	10	38	15	5.98 (11)	10.74 (12)	2.57 (12)
527	948.8	601.5	1	TAG(58:8/FA22:5)+NH4	80	10	38	15	1.91 (12)	4.12 (12)	8.79 (13)
528	948.8	603.5	1	TAG(58:8/FA22:6)+NH4	80	10	38	15	7.39 (13)	5.8 (13)	6.47 (14)
529	946.8	647.5	1	TAG(58:9/FA18:1)+NH4	80	10	38	15	8.46 (10)	3.11 (12)	8.93 (12)
530	946.8	649.5	1	TAG(58:9/FA18:2)+NH4	80	10	38	15	6.75 (12)	12.78 (11)	15.69 (26)
531	946.8	625.5	1.05	TAG(58:9/FA20:4)+NH4	80	10	38	15	7.11 (17)	7.76 (23)	5.2 (19)
532	946.8	599.5	1.01	TAG(58:9/FA22:5)+NH4	80	10	38	15	7 (13)	5.32 (14)	11.66 (13)
533	946.8	601.5	1	TAG(58:9/FA22:6)+NH4	80	10	38	15	7.49 (12)	7.28 (12)	5.36 (13)
534	972.8	625.5	1.05	TAG(60:10/FA22:5)+NH4	80	10	38	15	7.08 (15)	22.24 (14)	18.92 (8)
535	972.8	627.5	1.01	TAG(60:10/FA22:6)+NH4	80	10	38	15	5.31 (16)	13.59 (15)	17.44 (15)
536	970.8	623.5	1.02	TAG(60:11/FA22:5)+NH4	80	10	38	15	13.44 (13)	26.53 (18)	19.99 (19)
537	970.8	625.5	1.02	TAG(60:11/FA22:6)+NH4	80	10	38	15	8.19 (15)	17.48 (16)	22.63 (21)
538	968.8	623.5	1.01	TAG(60:12/FA22:6)+NH4	80	10	38	15	6.04 (14)	6.57 (14)	10.61 (14)
539	530.4	285.2	1	DAG(14:0/14:0)+NH4	80	10	26	15	#DIV/0!	23.2 (10)	42.91 (10)
540	556.5	285.2	1.01	DAG(14:0/16:1)+NH4	80	10	26	15	38.21 (13)	46.54 (15)	33.17 (17)
541	586.5	313.3	1	DAG(16:0/16:0)+NH4	80	10	26	15	28.01 (12)	#DIV/0!	26.94 (16)
542	584.4	285.2	1	DAG(14:0/18:1)+NH4	80	10	26	15	34.84 (12)	18.36 (30)	30.77 (27)
543	582.4	311.2	1	DAG(16:1/16:1)+NH4	80	10	26	15	5.59 (9)	31.2 (12)	27.46 (9)
544	580.4	285.2	1.01	DAG(14:0/18:3)+NH4	80	10	26	15	19.27 (15)	17.13 (21)	42.63 (21)
545	614.6	285.2	1.03	DAG(14:0/20:0)+NH4	80	10	26	15	#DIV/0!	28.94 (15)	16.67 (17)
546	612.6	311.3	1	DAG(16:1/18:0)+NH4	80	10	26	15	#DIV/0!	30.41 (17)	36.5 (10)
547	612.6	313.2	1	DAG(16:0/18:1)+NH4	80	10	26	15	22.87 (12)	14.81 (13)	14.43 (12)

548	610.4	311.2	1	DAG(16:1/18:1)+NH4	80	10	26	15	12.97 (8)	29.68 (10)	17.52 (10)
549	610.4	313.2	1	DAG(16:0/18:2)+NH4	80	10	26	15	36.51 (10)	12.83 (11)	12.28 (10)
550	608.5	313.2	1	DAG(16:0/18:3)+NH4	80	10	26	15	24.6 (15)	14.86 (14)	16.22 (14)
551	606.4	311.2	1	DAG(16:1/18:3)+NH4	80	10	26	15	29.19 (12)	38.13 (17)	24.5 (17)
552	606.4	285.2	1.02	DAG(14:0/20:4)+NH4	80	10	26	15	35.86 (17)	28.34 (19)	37.24 (16)
553	640.4	341.3	1	DAG(18:0/18:1)+NH4	80	10	26	15	19.53 (7)	42.69 (8)	45.54 (8)
554	638.4	339.3	1	DAG(18:1/18:1)+NH4	80	10	26	15	11.4 (13)	8.76 (14)	15.44 (12)
555	638.4	341.3	1.01	DAG(18:0/18:2)+NH4	80	10	26	15	#DIV/0!	31.7 (5)	93.53 (5)
556	636.5	339.3	1	DAG(18:1/18:2)+NH4	80	10	26	15	22.57 (12)	18.1 (15)	30.43 (13)
557	636.5	341.3	1.01	DAG(18:0/18:3)+NH4	80	10	26	15	37.35 (15)	21.36 (16)	21.97 (19)
558	636.6	311.3	1.01	DAG(16:1/20:2)+NH4	80	10	26	15	32.42 (16)	43.43 (7)	30.72 (29)
559	636.5	313.3	1.01	DAG(16:0/20:3)+NH4	80	10	26	15	21.37 (12)	12.96 (19)	37.8 (17)
560	634.5	313.3	1.01	DAG(16:0/20:4)+NH4	80	10	26	15	27.13 (18)	33.03 (19)	26.51 (25)
561	632.4	337.3	1.02	DAG(18:2/18:3)+NH4	80	10	26	15	17.84 (20)	27.88 (17)	30.12 (14)
562	632.4	311.3	1	DAG(16:1/20:4)+NH4	80	10	26	15	#DIV/0!	42.07 (14)	33.3 (14)
563	632.4	313.3	1.01	DAG(16:0/20:5)+NH4	80	10	26	15	43.05 (15)	22.13 (19)	33.45 (18)
564	630.5	285.3	1.01	DAG(14:0/22:6)+NH4	80	10	26	15	#DIV/0!	44.51 (24)	30.28 (16)
565	666.6	339.3	1	DAG(18:1/20:1)+NH4	80	10	26	15	33.48 (14)	21.03 (13)	17.98 (15)
566	664.6	339.3	1.01	DAG(18:1/20:2)+NH4	80	10	26	15	55.6 (16)	30.44 (23)	30.54 (23)
567	662.6	339.3	1.01	DAG(18:1/20:3)+NH4	80	10	26	15	21.69 (19)	25.16 (16)	31.1 (18)
568	660.5	337.3	1.04	DAG(18:2/20:3)+NH4	80	10	26	15	#DIV/0!	47.81 (15)	33.68 (15)
569	660.5	339.3	1.01	DAG(18:1/20:4)+NH4	80	10	26	15	20.79 (15)	15.5 (19)	13.54 (19)
570	660.5	313.3	1.01	DAG(16:0/22:5)+NH4	80	10	26	15	#DIV/0!	14.95 (11)	27.34 (18)
571	658.5	337.3	1.04	DAG(18:2/20:4)+NH4	80	10	26	15	39.34 (18)	29.4 (20)	19.94 (18)
572	658.5	339.3	1.01	DAG(18:1/20:5)+NH4	80	10	26	15	8.3 (20)	24.12 (27)	23.61 (20)
573	658.5	313.3	1	DAG(16:0/22:6)+NH4	80	10	26	15	13 (11)	21.97 (13)	14.58 (12)
574	656.5	337.3	1.07	DAG(18:2/20:5)+NH4	80	10	26	15	32.59 (17)	35.72 (20)	39.34 (17)
575	656.5	311.3	1.01	DAG(16:1/22:6)+NH4	80	10	26	15	58.19 (15)	19.29 (22)	25.96 (24)
576	698.6	369.3	1	DAG(20:0/20:0)+NH4	80	10	26	15	6.28 (14)	5.96 (15)	8.56 (14)

577	688.6	339.3	1	DAG(18:1/22:4)+NH4	80	10	26	15	14.91 (14)	8.97 (15)	26.61 (13)
578	686.6	337.3	1.03	DAG(18:2/22:4)+NH4	80	10	26	15	#DIV/0!	48.86 (16)	50.19 (18)
579	686.6	339.3	1.01	DAG(18:1/22:5)+NH4	80	10	26	15	43.83 (12)	25.2 (14)	38.15 (14)
580	684.6	337.3	1.04	DAG(18:2/22:5)+NH4	80	10	26	15	51.35 (16)	26.63 (18)	9.58 (17)
581	684.6	339.3	1	DAG(18:1/22:6)+NH4	80	10	26	15	39.35 (11)	18.57 (14)	12.56 (14)
582	682.5	337.3	1.04	DAG(18:2/22:6)+NH4	80	10	26	15	34.08 (16)	22.64 (15)	55.75 (15)
583	367.2	293.2	1.01	MAG(20:1)+NH4	80	10	25	15	7.69 (15)	5.94 (21)	12.29 (22)
584	363.2	289.2	1.06	MAG(20:3)+NH4	80	10	25	15	23.79 (15)	20.51 (16)	31.04 (14)
585	395.2	321.2	1.04	MAG(22:1)+NH4	80	10	25	15	25.34 (15)	15.5 (16)	24.51 (17)
586	391.2	317.2	1.04	MAG(22:3)+NH4	80	10	25	15	16.58 (13)	1.91 (18)	23.09 (13)
587	387.2	313.2	1.11	MAG(22:5)+NH4	80	10	25	15	11.51 (13)	#DIV/0!	33.87 (15)
588	526.317	227.202	1	LPC(14:0)+AcO	-80	-10	-50	-15	9.77 (9)	5.28 (11)	5.89 (16)
589	554.346	255.233	1	LPC(16:0)+AcO	-80	-10	-50	-15	2.59 (11)	1.89 (13)	1.18 (20)
590	552.331	253.217	1	LPC(16:1)+AcO	-80	-10	-50	-15	4.88 (11)	2.96 (12)	3.33 (18)
591	582.378	283.264	1	LPC(18:0)+AcO	-80	-10	-50	-15	2.51 (13)	2.35 (13)	2.79 (21)
592	580.362	281.249	1	LPC(18:1)+AcO	-80	-10	-50	-15	3.54 (12)	2.28 (13)	2.11 (21)
593	578.346	279.233	1	LPC(18:2)+AcO	-80	-10	-50	-15	5.11 (12)	3.11 (12)	2.14 (19)
594	576.331	277.217	1.09	LPC(18:3)+AcO	-80	-10	-50	-15	11.53 (12)	4.88 (15)	5.71 (22)
595	610.409	311.3	1	LPC(20:0)+AcO	-80	-10	-50	-15	9.68 (12)	9.89 (13)	4.98 (17)
596	608.393	309.28	1	LPC(20:1)+AcO	-80	-10	-50	-15	7.08 (13)	3.06 (12)	1.83 (19)
597	606.378	307.264	1.01	LPC(20:2)+AcO	-80	-10	-50	-15	6.61 (12)	8.37 (14)	4.88 (18)
598	604.362	305.249	1	LPC(20:3)+AcO	-80	-10	-50	-15	8.57 (14)	7.58 (14)	4.24 (20)

599	602.346	303.233	1.03	LPC(20:4)+AcO	-80	-10	-50	-15	3.34 (13)	3.98 (13)	6.43 (20)
600	600.331	301.217	3.25	LPC(20:5)+AcO	-80	-10	-50	-15	97.45 (7)	39.58 (8)	12.27 (13)
601	630.364	331.264	1.22	LPC(22:4)+AcO	-80	-10	-50	-15	11.35 (12)	20 (10)	12.13 (16)
602	628.362	329.249	1.17	LPC(22:5)+AcO	-80	-10	-50	-15	3.32 (34)	4.23 (29)	7.08 (23)
603	626.346	327.233	1.22	LPC(22:6)+AcO	-80	-10	-50	-15	6.13 (20)	20.82 (14)	11.47 (22)
604	736.513	227.202	1	PC(14:0/14:0)+AcO	-80	-10	-50	-15	39.71 (15)	22.47 (17)	18.61 (23)
605	790.56	281.249	1	PC(14:0/18:1)+AcO	-80	-10	-50	-15	5.94 (18)	7.89 (20)	8.59 (22)
606	788.545	279.233	1	PC(14:0/18:2)+AcO	-80	-10	-50	-15	8.27 (17)	5.3 (21)	7.03 (20)
607	786.529	277.217	1	PC(14:0/18:3)+AcO	-80	-10	-50	-15	37.8 (11)	29.94 (18)	32.08 (15)
608	818.592	309.28	1	PC(14:0/20:1)+AcO	-80	-10	-50	-15	37.33 (11)	23.94 (25)	16.39 (21)
609	816.576	307.264	1	PC(14:0/20:2)+AcO	-80	-10	-50	-15	20.85 (15)	7.75 (28)	10.66 (27)
610	814.56	305.249	1	PC(14:0/20:3)+AcO	-80	-10	-50	-15	24.94 (11)	11.87 (22)	14.67 (19)
611	812.545	303.233	1	PC(14:0/20:4)+AcO	-80	-10	-50	-15	5.12 (30)	13.97 (33)	6.15 (33)
612	810.529	301.217	1.03	PC(14:0/20:5)+AcO	-80	-10	-50	-15	18.96 (19)	27.93 (18)	42.94 (17)
613	838.56	329.249	1.02	PC(14:0/22:5)+AcO	-80	-10	-50	-15	27.41 (10)	39.21 (22)	38.19 (11)
614	732.5	225.2	1.02	PC(14:1/14:1)+AcO	-80	-10	-50	-15	40.68 (15)	31.86 (17)	28.24 (22)
615	764.545	227.202	1	PC(16:0/14:0)+AcO	-80	-10	-50	-15	5.27 (16)	4.16 (18)	1.95 (21)
616	792.576	255.233	1	PC(16:0/16:0)+AcO	-80	-10	-50	-15	4.1 (21)	2.94 (28)	1.54 (30)

617	790.56	253.217	1	PC(16:0/16:1)+AcO	-80	-10	-50	-15	13.01 (19)	3.92 (23)	2.35 (24)
618	820.607	283.264	1	PC(16:0/18:0)+AcO	-80	-10	-50	-15	2.36 (28)	3.72 (31)	3.73 (32)
619	818.592	281.249	1	PC(16:0/18:1)+AcO	-80	-10	-50	-15	3.36 (26)	1.82 (32)	2.18 (32)
620	816.576	279.233	1	PC(16:0/18:2)+AcO	-80	-10	-50	-15	3.05 (25)	0.65 (31)	0.86 (28)
621	814.56	277.217	1	PC(16:0/18:3)+AcO	-80	-10	-50	-15	5.87 (21)	5.53 (25)	2.41 (26)
622	846.623	309.28	1	PC(16:0/20:1)+AcO	-80	-10	-50	-15	6.67 (28)	5.93 (31)	6.12 (32)
623	844.607	307.264	1	PC(16:0/20:2)+AcO	-80	-10	-50	-15	4.39 (23)	7.16 (26)	4.25 (26)
624	842.592	305.249	1	PC(16:0/20:3)+AcO	-80	-10	-50	-15	2.55 (25)	1.58 (29)	2.15 (30)
625	840.576	303.233	1	PC(16:0/20:4)+AcO	-80	-10	-50	-15	3.62 (26)	3.3 (29)	2.67 (30)
626	838.56	301.217	1	PC(16:0/20:5)+AcO	-80	-10	-50	-15	5.11 (22)	4.55 (22)	5.59 (22)
627	868.607	331.264	1	PC(16:0/22:4)+AcO	-80	-10	-50	-15	5.78 (22)	4.41 (26)	3.56 (30)
628	866.592	329.249	1	PC(16:0/22:5)+AcO	-80	-10	-50	-15	3.5 (24)	1.43 (26)	2.7 (29)
629	816.576	281.249	1	PC(16:1/18:1)+AcO	-80	-10	-50	-15	4.99 (23)	9.19 (28)	5.28 (31)
630	814.56	253.217	1	PC(16:1/18:2)+AcO	-80	-10	-50	-15	7.51 (21)	2.65 (19)	4.4 (23)
631	864.576	327.233	1	PC(16:0/22:6)+AcO	-80	-10	-50	-15	5.27 (22)	3.62 (22)	2.61 (27)
632	792.576	227.202	1	PC(18:0/14:0)+AcO	-80	-10	-50	-15	22.34 (18)	11.09 (18)	11.09 (21)
633	818.592	253.217	1	PC(18:0/16:1)+AcO	-80	-10	-50	-15	7.05 (21)	5.8 (20)	9.79 (25)
634	848.639	283.264	1	PC(18:0/18:0)+AcO	-80	-10	-50	-15	8.09 (27)	2.96 (30)	2.14 (30)

635	846.623	281.249	1	PC(18:0/18:1)+AcO	-80	-10	-50	-15	1.8 (26)	1.26 (31)	0.96 (28)
636	844.607	279.233	1	PC(18:0/18:2)+AcO	-80	-10	-50	-15	1.46 (27)	2.5 (31)	1.38 (31)
637	842.592	277.217	1	PC(18:0/18:3)+AcO	-80	-10	-50	-15	9.1 (25)	7.91 (30)	4.87 (30)
638	876.67	283.264	1	PC(18:0/20:0)+AcO	-80	-10	-50	-15	#DIV/0!	7.14 (22)	3.44 (23)
639	874.654	309.28	1	PC(18:0/20:1)+AcO	-80	-10	-50	-15	6.33 (31)	8.4 (34)	7.6 (36)
640	872.639	307.264	1	PC(18:0/20:2)+AcO	-80	-10	-50	-15	5.39 (24)	4.04 (27)	4.6 (26)
641	870.623	305.249	1	PC(18:0/20:3)+AcO	-80	-10	-50	-15	2.96 (25)	1.87 (28)	2.37 (30)
642	868.607	303.233	1	PC(18:0/20:4)+AcO	-80	-10	-50	-15	5.95 (26)	2.05 (27)	4.07 (27)
643	866.592	301.217	1	PC(18:0/20:5)+AcO	-80	-10	-50	-15	8.21 (21)	5.86 (20)	3.24 (27)
644	896.639	331.264	1	PC(18:0/22:4)+AcO	-80	-10	-50	-15	6.64 (21)	4.52 (22)	3.1 (23)
645	894.623	329.249	1	PC(18:0/22:5)+AcO	-80	-10	-50	-15	3.67 (30)	3.23 (25)	3.37 (30)
646	892.607	327.233	1	PC(18:0/22:6)+AcO	-80	-10	-50	-15	5.94 (23)	2.97 (26)	4.74 (25)
647	816.576	281.249	1	PC(18:1/16:1)+AcO	-80	-10	-50	-15	9.6 (23)	5.46 (29)	5.2 (31)
648	844.607	281.249	1	PC(18:1/18:1)+AcO	-80	-10	-50	-15	2.57 (24)	4.22 (26)	2.34 (29)
649	842.592	279.233	1	PC(18:1/18:2)+AcO	-80	-10	-50	-15	1.08 (22)	2.92 (26)	3.12 (26)
650	840.576	277.217	1.01	PC(18:1/18:3)+AcO	-80	-10	-50	-15	15.29 (22)	7.73 (21)	8.64 (23)
651	872.639	309.28	1	PC(18:1/20:1)+AcO	-80	-10	-50	-15	8.26 (23)	5.88 (25)	7.58 (23)
652	870.623	307.264	1	PC(18:1/20:2)+AcO	-80	-10	-50	-15	11.03 (25)	6.31 (24)	6.91 (25)

653	868.607	305.249	1	PC(18:1/20:3)+AcO	-80	-10	-50	-15	7.86 (22)	3.54 (23)	5.13 (25)
654	866.592	303.233	1	PC(18:1/20:4)+AcO	-80	-10	-50	-15	3.85 (22)	3.25 (25)	5.36 (22)
655	864.576	301.217	1.12	PC(18:1/20:5)+AcO	-80	-10	-50	-15	21.85 (23)	14.13 (23)	14.94 (22)
656	894.623	331.264	1.04	PC(18:1/22:4)+AcO	-80	-10	-50	-15	16.57 (22)	14.96 (21)	7.18 (21)
657	892.607	329.249	1.04	PC(18:1/22:5)+AcO	-80	-10	-50	-15	9.98 (22)	6.86 (23)	10.69 (24)
658	890.592	327.233	1.11	PC(18:1/22:6)+AcO	-80	-10	-50	-15	14.37 (17)	15.4 (20)	12.4 (21)
659	814.56	279.233	1	PC(18:2/16:1)+AcO	-80	-10	-50	-15	5.99 (22)	3.52 (27)	5.99 (28)
660	840.576	279.233	1	PC(18:2/18:2)+AcO	-80	-10	-50	-15	2.05 (20)	2.61 (24)	4.05 (26)
661	838.56	277.217	1.01	PC(18:2/18:3)+AcO	-80	-10	-50	-15	10.7 (22)	18.64 (18)	15.78 (22)
662	870.623	309.28	1	PC(18:2/20:1)+AcO	-80	-10	-50	-15	9.56 (24)	10.44 (20)	4.32 (22)
663	868.607	307.264	1	PC(18:2/20:2)+AcO	-80	-10	-50	-15	13.03 (30)	12.17 (29)	10.28 (30)
664	866.592	305.249	1	PC(18:2/20:3)+AcO	-80	-10	-50	-15	8.23 (20)	9.51 (22)	4.17 (25)
665	864.576	303.233	1	PC(18:2/20:4)+AcO	-80	-10	-50	-15	9.98 (29)	4.4 (23)	6.83 (25)
666	862.56	301.217	1.1	PC(18:2/20:5)+AcO	-80	-10	-50	-15	28.12 (25)	16.46 (30)	20.24 (32)
667	890.592	329.249	1.13	PC(18:2/22:5)+AcO	-80	-10	-50	-15	32.41 (23)	23.86 (29)	15.28 (35)
668	888.576	327.233	1.1	PC(18:2/22:6)+AcO	-80	-10	-50	-15	56.49 (19)	41.11 (18)	34.17 (30)
669	846.623	253.217	1	PC(20:0/16:1)+AcO	-80	-10	-50	-15	#DIV/0!	17.08 (19)	#DIV/0!
670	874.654	281.249	1	PC(20:0/18:1)+AcO	-80	-10	-50	-15	61.78 (7)	6.71 (22)	55.51 (5)

671	870.623	277.217	1.06	PC(20:0/18:3)+AcO	-80	-10	-50	-15	#DIV/0!	54.17 (15)	2.56 (7)
672	902.685	309.28	1	PC(20:0/20:1)+AcO	-80	-10	-50	-15	113.44 (15)	15.04 (19)	51.94 (18)
673	900.67	307.264	1.03	PC(20:0/20:2)+AcO	-80	-10	-50	-15	33.1 (22)	7.66 (29)	#DIV/0!
674	898.654	305.249	1.02	PC(20:0/20:3)+AcO	-80	-10	-50	-15	13.1 (21)	17.25 (24)	88.48 (6)
675	896.639	303.233	1.03	PC(20:0/20:4)+AcO	-80	-10	-50	-15	25.28 (25)	15.64 (21)	44.66 (6)
676	894.623	301.217	1.09	PC(20:0/20:5)+AcO	-80	-10	-50	-15	34.4 (12)	39.87 (14)	#DIV/0!
677	922.654	329.249	1.16	PC(20:0/22:5)+AcO	-80	-10	-50	-15	49.15 (17)	100.75 (12)	#DIV/0!
678	920.639	327.233	1.13	PC(20:0/22:6)+AcO	-80	-10	-50	-15	91.44 (13)	52.91 (18)	#DIV/0!
679	424.247	227.202	2.56	LPE(14:0)-H	-80	-10	-50	-15	#DIV/0!	31.91 (12)	23.88 (14)
680	452.278	255.233	1	LPE(16:0)-H	-80	-10	-50	-15	14.81 (17)	11.22 (15)	11.15 (16)
681	450.263	253.217	1.01	LPE(16:1)-H	-80	-10	-40	-15	27.35 (21)	17.69 (17)	13.25 (19)
682	480.31	283.264	1	LPE(18:0)-H	-80	-10	-40	-15	2.61 (12)	3.52 (13)	2.35 (14)
683	478.293	281.249	1	LPE(18:1)-H	-80	-10	-40	-15	5.17 (13)	3.83 (12)	0.99 (14)
684	476.278	279.233	1	LPE(18:2)-H	-80	-10	-40	-15	4.93 (16)	8.4 (15)	3.82 (17)
685	474.263	277.217	1.92	LPE(18:3)-H	-80	-10	-40	-15	15.67 (19)	21.85 (20)	7.69 (19)
686	508.341	311.3	1.06	LPE(20:0)-H	-80	-10	-40	-15	29.38 (19)	8.58 (15)	7.39 (17)
687	506.325	309.28	1.03	LPE(20:1)-H	-80	-10	-40	-15	8.34 (20)	10.69 (15)	5.36 (18)
688	504.31	307.264	1.2	LPE(20:2)-H	-80	-10	-40	-15	23.45 (17)	9.69 (16)	6.4 (19)

689	502.294	305.249	1.17	LPE(20:3)-H	-80	-10	-40	-15	6.94 (18)	6.49 (17)	5.54 (19)
690	500.278	303.233	1.11	LPE(20:4)-H	-80	-10	-40	-15	13.78 (18)	7.03 (19)	6.79 (17)
691	498.263	301.217	4.4	LPE(20:5)-H	-80	-10	-40	-15	34.02 (16)	30.77 (20)	34.95 (19)
692	528.31	331.264	1.2	LPE(22:4)-H	-80	-10	-40	-15	6.81 (17)	13.32 (19)	13.51 (20)
693	526.294	329.249	1.57	LPE(22:5)-H	-80	-10	-40	-15	18.67 (18)	5.03 (18)	9.67 (19)
694	524.278	327.233	2.98	LPE(22:6)-H	-80	-10	-40	-15	18.28 (18)	14.18 (19)	12.33 (19)
695	634.445	227.202	1.15	PE(14:0/14:0)-H	-80	-10	-50	-15	44.22 (13)	43.53 (18)	35.85 (22)
696	660.461	253.217	1.04	PE(14:0/16:1)-H	-80	-10	-43	-15	28.91 (13)	30.02 (18)	22.45 (14)
697	688.492	281.249	1	PE(14:0/18:1)-H	-80	-10	-43	-15	25.04 (15)	35.57 (12)	70.85 (9)
698	686.477	279.233	1.04	PE(14:0/18:2)-H	-80	-10	-43	-15	22.81 (24)	17.89 (24)	64.48 (5)
699	714.508	307.264	1.58	PE(14:0/20:2)-H	-80	-10	-50	-15	104.49 (11)	31.1 (12)	68.14 (14)
700	712.492	305.249	1.44	PE(14:0/20:3)-H	-80	-10	-50	-15	66.42 (17)	38.6 (12)	47.07 (7)
701	710.477	303.233	1.6	PE(14:0/20:4)-H	-80	-10	-50	-15	25.23 (23)	42.39 (23)	67.04 (6)
702	736.492	329.249	1.3	PE(14:0/22:5)-H	-80	-10	-50	-15	28.85 (20)	32.55 (21)	99.26 (10)
703	734.477	327.233	1.16	PE(14:0/22:6)-H	-80	-10	-50	-15	24.17 (22)	19.89 (24)	#DIV/0!
704	662.477	255.233	1	PE(16:0/14:0)-H	-80	-10	-50	-15	15.63 (28)	4.71 (44)	18.71 (38)
705	690.508	255.233	1	PE(16:0/16:0)-H	-80	-10	-50	-15	10.23 (18)	12.19 (22)	4.55 (23)
706	688.492	253.217	1	PE(16:0/16:1)-H	-80	-10	-50	-15	3.9 (15)	7.15 (17)	8.78 (19)

707	716.524	281.249	1	PE(16:0/18:1)-H	-80	-10	-50	-15	6.54 (18)	3.34 (17)	3.71 (21)
708	714.508	279.233	1	PE(16:0/18:2)-H	-80	-10	-50	-15	6.51 (30)	4.31 (32)	3.05 (31)
709	712.492	277.217	1.08	PE(16:0/18:3)-H	-80	-10	-50	-15	10.98 (25)	10.14 (28)	6.47 (30)
710	744.555	309.28	1.01	PE(16:0/20:1)-H	-80	-10	-50	-15	13.14 (18)	8.11 (19)	4.36 (23)
711	742.539	307.264	1.01	PE(16:0/20:2)-H	-80	-10	-50	-15	16.63 (22)	7.33 (29)	7.56 (27)
712	740.524	305.249	1.01	PE(16:0/20:3)-H	-80	-10	-50	-15	5.26 (29)	5.29 (31)	1.58 (33)
713	738.508	303.233	1	PE(16:0/20:4)-H	-80	-10	-50	-15	4.14 (21)	2.38 (21)	2.29 (23)
714	736.492	301.217	1.55	PE(16:0/20:5)-H	-80	-10	-50	-15	7.24 (26)	10.7 (24)	6.94 (34)
715	766.539	331.264	1.03	PE(16:0/22:4)-H	-80	-10	-50	-15	9.47 (17)	3.38 (21)	4.15 (23)
716	764.524	329.249	1.04	PE(16:0/22:5)-H	-80	-10	-50	-15	3.62 (28)	1.2 (30)	3.61 (32)
717	762.508	327.233	1.05	PE(16:0/22:6)-H	-80	-10	-50	-15	1.49 (21)	5.5 (21)	4.57 (24)
718	690.508	283.264	1	PE(18:0/14:0)-H	-80	-10	-50	-15	14.23 (19)	7.28 (34)	14.24 (49)
719	718.539	283.264	1	PE(18:0/16:0)-H	-80	-10	-50	-15	8.04 (32)	10.24 (31)	21.24 (17)
720	716.524	283.264	1	PE(18:0/16:1)-H	-80	-10	-50	-15	23.89 (26)	14.28 (33)	12.55 (17)
721	746.57	283.264	1	PE(18:0/18:0)-H	-80	-10	-50	-15	1.17 (30)	4.69 (33)	2.76 (29)
722	744.555	281.249	1	PE(18:0/18:1)-H	-80	-10	-50	-15	4.07 (30)	3.26 (30)	3.19 (31)
723	742.539	279.233	1	PE(18:0/18:2)-H	-80	-10	-50	-15	1.1 (31)	2.61 (26)	1.87 (26)
724	740.524	277.217	1	PE(18:0/18:3)-H	-80	-10	-50	-15	3.25 (27)	4.28 (26)	3.82 (27)

725	772.586	309.28	1	PE(18:0/20:1)-H	-80	-10	-50	-15	6.18 (30)	0.65 (34)	3.65 (31)
726	770.57	307.264	1	PE(18:0/20:2)-H	-80	-10	-50	-15	3.48 (31)	3.45 (33)	1.18 (34)
727	768.555	305.249	1	PE(18:0/20:3)-H	-80	-10	-50	-15	4.07 (29)	2.45 (28)	2.49 (30)
728	766.539	303.233	1	PE(18:0/20:4)-H	-80	-10	-50	-15	1.64 (30)	1.92 (31)	2.86 (30)
729	764.524	301.217	1.02	PE(18:0/20:5)-H	-80	-10	-50	-15	4.17 (25)	3.61 (25)	4.79 (26)
730	794.57	331.264	1	PE(18:0/22:4)-H	-80	-10	-50	-15	3.47 (25)	4.98 (27)	5.62 (26)
731	792.555	329.249	1	PE(18:0/22:5)-H	-80	-10	-50	-15	1.68 (27)	5.37 (27)	4.84 (29)
732	790.539	327.233	1	PE(18:0/22:6)-H	-80	-10	-50	-15	2.29 (36)	1.73 (33)	2.14 (36)
733	714.508	281.249	1	PE(18:1/16:1)-H	-80	-10	-50	-15	26.4 (13)	18.08 (18)	9.03 (16)
734	742.539	281.249	1	PE(18:1/18:1)-H	-80	-10	-50	-15	4.42 (33)	1.51 (33)	3.24 (32)
735	740.524	279.233	1	PE(18:1/18:2)-H	-80	-10	-50	-15	3.15 (35)	1.37 (32)	2.27 (32)
736	738.508	277.217	1.14	PE(18:1/18:3)-H	-80	-10	-50	-15	16.67 (29)	15.2 (29)	10.15 (29)
737	770.57	309.28	1.01	PE(18:1/20:1)-H	-80	-10	-50	-15	7.29 (23)	8.02 (28)	2.43 (35)
738	768.555	307.264	1.01	PE(18:1/20:2)-H	-80	-10	-50	-15	12.98 (25)	8.63 (27)	5.18 (30)
739	766.539	305.249	1.01	PE(18:1/20:3)-H	-80	-10	-50	-15	3.3 (26)	4.77 (28)	3.83 (29)
740	764.524	303.233	1.01	PE(18:1/20:4)-H	-80	-10	-50	-15	1.59 (32)	7.01 (34)	3.39 (35)
741	762.508	301.217	1.7	PE(18:1/20:5)-H	-80	-10	-50	-15	8.12 (13)	8.41 (14)	33.82 (11)
742	792.555	331.264	1.08	PE(18:1/22:4)-H	-80	-10	-50	-15	6.56 (27)	14.12 (28)	7.81 (31)

743	790.539	329.249	1.11	PE(18:1/22:5)-H	-80	-10	-50	-15	6.73 (26)	12.75 (23)	13.09 (26)
744	788.524	327.233	1.06	PE(18:1/22:6)-H	-80	-10	-50	-15	3.53 (31)	8.35 (35)	6.5 (35)
745	712.492	279.233	1	PE(18:2/16:1)-H	-80	-10	-50	-15	2.78 (24)	6.02 (29)	3.13 (30)
746	738.508	279.233	1.01	PE(18:2/18:2)-H	-80	-10	-50	-15	3.64 (21)	4.37 (23)	5.36 (26)
747	736.492	277.217	1.24	PE(18:2/18:3)-H	-80	-10	-50	-15	14.24 (22)	15.45 (22)	23.15 (24)
748	768.555	309.28	1.09	PE(18:2/20:1)-H	-80	-10	-50	-15	14.12 (20)	3.31 (24)	10.43 (23)
749	766.539	307.264	1.26	PE(18:2/20:2)-H	-80	-10	-50	-15	21.06 (21)	27.06 (24)	18.71 (24)
750	764.524	305.249	1.52	PE(18:2/20:3)-H	-80	-10	-50	-15	15.7 (28)	7.18 (32)	9.23 (35)
751	762.508	303.233	1.23	PE(18:2/20:4)-H	-80	-10	-50	-15	6.76 (29)	7.68 (31)	6.58 (35)
752	760.492	301.217	1.53	PE(18:2/20:5)-H	-80	-10	-50	-15	13.45 (27)	52.43 (23)	16.87 (27)
753	790.539	331.264	1.44	PE(18:2/22:4)-H	-80	-10	-50	-15	9.88 (27)	22.77 (21)	14.87 (33)
754	788.524	329.249	1.28	PE(18:2/22:5)-H	-80	-10	-50	-15	13.74 (25)	24.93 (31)	10.47 (26)
755	786.508	327.233	1.98	PE(18:2/22:6)-H	-80	-10	-50	-15	17.08 (25)	13.2 (24)	18.57 (24)
756	676.529	255.233	1	PE(O-16:0/16:0)-H	-80	-10	-50	-15	21.37 (26)	2.3 (44)	6.16 (45)
757	674.513	253.217	1.04	PE(O-16:0/16:1)-H	-80	-10	-50	-15	16.82 (27)	14.76 (28)	23.52 (24)
758	704.56	283.264	1	PE(O-16:0/18:0)-H	-80	-10	-50	-15	35.6 (22)	7.07 (47)	19.25 (45)
759	702.544	281.249	1	PE(O-16:0/18:1)-H	-80	-10	-50	-15	8.88 (17)	5.81 (19)	14.8 (19)
760	700.529	279.233	1.01	PE(O-16:0/18:2)-H	-80	-10	-50	-15	8.05 (16)	27.04 (16)	10.82 (14)

761	698.513	277.217	1.38	PE(O-16:0/18:3)-H	-80	-10	-50	-15	18.43 (19)	12.3 (24)	10.69 (26)
762	730.576	309.28	1.04	PE(O-16:0/20:1)-H	-80	-10	-50	-15	12.01 (19)	11.95 (21)	16.83 (25)
763	728.56	307.264	1.12	PE(O-16:0/20:2)-H	-80	-10	-50	-15	16.57 (19)	24.51 (25)	31.05 (29)
764	726.544	305.249	1.06	PE(O-16:0/20:3)-H	-80	-10	-50	-15	11.97 (28)	7.67 (30)	5.45 (29)
765	724.529	303.233	1.03	PE(O-16:0/20:4)-H	-80	-10	-50	-15	2.15 (22)	5.83 (21)	6.22 (22)
766	722.513	301.217	1.38	PE(O-16:0/20:5)-H	-80	-10	-50	-15	11.55 (28)	15.43 (26)	23.22 (28)
767	752.56	331.264	1.08	PE(O-16:0/22:4)-H	-80	-10	-50	-15	5.74 (19)	8.03 (20)	8.9 (21)
768	750.544	329.249	1.08	PE(O-16:0/22:5)-H	-80	-10	-50	-15	6.32 (21)	4.12 (24)	4.18 (24)
769	748.529	327.233	1.26	PE(O-16:0/22:6)-H	-80	-10	-50	-15	2.1 (24)	10.36 (26)	3.73 (26)
770	704.56	255.233	1	PE(O-18:0/16:0)-H	-80	-10	-50	-15	6.72 (24)	2.74 (30)	4.23 (27)
771	702.544	253.217	1	PE(O-18:0/16:1)-H	-80	-10	-50	-15	7.71 (22)	10.81 (28)	9.66 (27)
772	732.591	283.264	1	PE(O-18:0/18:0)-H	-80	-10	-50	-15	15.12 (30)	8.98 (45)	23.38 (30)
773	730.576	281.249	1	PE(O-18:0/18:1)-H	-80	-10	-50	-15	5.61 (28)	3.77 (29)	1.09 (30)
774	728.56	279.233	1	PE(O-18:0/18:2)-H	-80	-10	-50	-15	6.94 (26)	1.71 (27)	2.66 (29)
775	726.544	277.217	1.07	PE(O-18:0/18:3)-H	-80	-10	-50	-15	12.49 (25)	11.92 (29)	13.25 (31)
776	758.607	309.28	1	PE(O-18:0/20:1)-H	-80	-10	-50	-15	12.17 (24)	10.9 (25)	3.7 (25)
777	756.591	307.264	1.01	PE(O-18:0/20:2)-H	-80	-10	-50	-15	14.78 (29)	9.33 (33)	3.84 (31)
778	754.576	305.249	1	PE(O-18:0/20:3)-H	-80	-10	-50	-15	4.74 (27)	5.74 (31)	3.32 (28)

779	752.56	303.233	1	PE(O-18:0/20:4)-H	-80	-10	-50	-15	4.06 (24)	3.06 (26)	2.57 (29)
780	750.544	301.217	1.16	PE(O-18:0/20:5)-H	-80	-10	-50	-15	14.53 (31)	7.45 (27)	8.74 (33)
781	780.591	331.264	1.01	PE(O-18:0/22:4)-H	-80	-10	-50	-15	7.84 (21)	8.15 (24)	7.79 (23)
782	778.576	329.249	1.03	PE(O-18:0/22:5)-H	-80	-10	-50	-15	4.33 (23)	2.24 (24)	6.23 (26)
783	776.56	327.233	1.11	PE(O-18:0/22:6)-H	-80	-10	-50	-15	5.9 (28)	8.68 (30)	5.86 (32)
784	674.5	283.264	1	PE(P-14:0/18:0)-H	-80	-10	-50	-15	42.83 (17)	11.93 (31)	10.89 (31)
785	672.5	281.249	1.01	PE(P-14:0/18:1)-H	-80	-10	-50	-15	33.93 (19)	2.9 (38)	12.51 (34)
786	674.5	255.233	1	PE(P-16:0/16:0)-H	-80	-10	-50	-15	12.31 (29)	9.01 (46)	7.24 (34)
787	672.5	253.217	1	PE(P-16:0/16:1)-H	-80	-10	-50	-15	10.22 (19)	8.05 (31)	6.03 (31)
788	702.5	283.264	1	PE(P-16:0/18:0)-H	-80	-10	-50	-15	35.67 (18)	12.15 (33)	13.34 (19)
789	700.5	281.249	1	PE(P-16:0/18:1)-H	-80	-10	-50	-15	6.62 (29)	4.65 (30)	3.88 (32)
790	698.5	279.233	1	PE(P-16:0/18:2)-H	-80	-10	-50	-15	3.51 (29)	4.95 (31)	3.79 (33)
791	696.5	277.217	1.16	PE(P-16:0/18:3)-H	-80	-10	-50	-15	10.78 (18)	5.12 (20)	3.56 (28)
792	728.6	309.28	1.01	PE(P-16:0/20:1)-H	-80	-10	-50	-15	5.33 (23)	11.8 (23)	4.77 (25)
793	726.5	307.264	1.03	PE(P-16:0/20:2)-H	-80	-10	-50	-15	14.2 (24)	8.4 (24)	4.99 (30)
794	724.5	305.249	1	PE(P-16:0/20:3)-H	-80	-10	-50	-15	1.84 (25)	1.37 (32)	3.76 (33)
795	722.5	303.233	1	PE(P-16:0/20:4)-H	-80	-10	-50	-15	3.09 (20)	4.31 (21)	3.2 (23)
796	720.5	301.217	1.3	PE(P-16:0/20:5)-H	-80	-10	-50	-15	10.31 (18)	6.51 (22)	5.73 (21)

797	750.5	331.264	1.01	PE(P-16:0/22:4)-H	-80	-10	-50	-15	3.2 (22)	3.22 (21)	2.98 (23)
798	748.5	329.249	1.02	PE(P-16:0/22:5)-H	-80	-10	-50	-15	4.05 (22)	6.37 (21)	4.45 (21)
799	746.5	327.233	1.1	PE(P-16:0/22:6)-H	-80	-10	-50	-15	4.82 (21)	5 (21)	3.2 (22)
800	698.5	281.249	1.02	PE(P-16:1/18:1)-H	-80	-10	-50	-15	32.78 (28)	20.73 (41)	30.66 (27)
801	702.5	255.233	1	PE(P-18:0/16:0)-H	-80	-10	-50	-15	5.36 (33)	3.16 (41)	6.52 (36)
802	700.5	253.217	1	PE(P-18:0/16:1)-H	-80	-10	-50	-15	18.61 (30)	4.3 (32)	2.81 (32)
803	730.6	283.264	1	PE(P-18:0/18:0)-H	-80	-10	-50	-15	7.91 (25)	10.62 (48)	10.36 (34)
804	728.6	281.249	1	PE(P-18:0/18:1)-H	-80	-10	-50	-15	4.66 (30)	3.6 (35)	1.51 (32)
805	726.5	279.233	1	PE(P-18:0/18:2)-H	-80	-10	-50	-15	4.94 (31)	3.11 (36)	2.39 (34)
806	724.5	277.217	1.05	PE(P-18:0/18:3)-H	-80	-10	-50	-15	7.41 (32)	9.76 (29)	12.68 (30)
807	756.6	309.28	1	PE(P-18:0/20:1)-H	-80	-10	-50	-15	6.63 (29)	5.7 (30)	8.68 (33)
808	754.6	307.264	1.01	PE(P-18:0/20:2)-H	-80	-10	-50	-15	12.27 (26)	9.11 (30)	5.9 (34)
809	752.6	305.249	1	PE(P-18:0/20:3)-H	-80	-10	-50	-15	2.29 (32)	1.47 (33)	3.5 (36)
810	750.5	303.233	1	PE(P-18:0/20:4)-H	-80	-10	-50	-15	3.67 (34)	3.33 (35)	3.36 (37)
811	748.5	301.217	1.14	PE(P-18:0/20:5)-H	-80	-10	-50	-15	4.22 (21)	6.41 (22)	1.02 (22)
812	778.6	331.264	1.01	PE(P-18:0/22:4)-H	-80	-10	-50	-15	8.62 (29)	4.06 (30)	4.9 (32)
813	776.6	329.249	1.02	PE(P-18:0/22:5)-H	-80	-10	-50	-15	3.95 (30)	3.12 (30)	4.29 (34)
814	774.5	327.233	1.07	PE(P-18:0/22:6)-H	-80	-10	-50	-15	3.27 (25)	6.73 (23)	3.85 (25)

815	700.5	255.233	1	PE(P-18:1/16:0)-H	-80	-10	-50	-15	10.96 (28)	5.94 (34)	3.01 (31)
816	698.5	253.217	1.04	PE(P-18:1/16:1)-H	-80	-10	-50	-15	10.98 (24)	15.66 (29)	10.14 (27)
817	726.5	281.249	1	PE(P-18:1/18:1)-H	-80	-10	-50	-15	2.55 (23)	5.85 (25)	2.75 (27)
818	724.5	279.233	1	PE(P-18:1/18:2)-H	-80	-10	-50	-15	2.89 (24)	1.64 (25)	1.89 (25)
819	722.5	277.217	1.25	PE(P-18:1/18:3)-H	-80	-10	-50	-15	14.94 (24)	11.1 (26)	8.52 (28)
820	754.6	309.28	1.03	PE(P-18:1/20:1)-H	-80	-10	-50	-15	7.11 (21)	4.47 (21)	13.78 (23)
821	752.6	307.264	1.16	PE(P-18:1/20:2)-H	-80	-10	-50	-15	7.19 (22)	6.43 (28)	6.05 (31)
822	750.5	305.249	1.05	PE(P-18:1/20:3)-H	-80	-10	-50	-15	5.05 (24)	4.35 (27)	1.46 (29)
823	748.5	303.233	1.02	PE(P-18:1/20:4)-H	-80	-10	-50	-15	5.12 (23)	3.1 (23)	3.58 (24)
824	746.5	301.217	1.61	PE(P-18:1/20:5)-H	-80	-10	-50	-15	4.25 (25)	4.73 (33)	5.38 (27)
825	776.6	331.264	1.08	PE(P-18:1/22:4)-H	-80	-10	-50	-15	4.76 (24)	8.53 (23)	7.12 (23)
826	774.5	329.249	1.17	PE(P-18:1/22:5)-H	-80	-10	-50	-15	3.2 (25)	8.98 (28)	4.9 (31)
827	772.5	327.233	1.25	PE(P-18:1/22:6)-H	-80	-10	-50	-15	4.96 (28)	6.85 (29)	5.93 (31)
828	722.5	279.233	1.05	PE(P-18:2/18:2)-H	-80	-10	-50	-15	5.65 (24)	6.95 (23)	6.62 (24)
829	746.5	303.233	1.31	PE(P-18:2/20:4)-H	-80	-10	-50	-15	2.92 (25)	4.48 (24)	6.5 (25)
830	770.5	327.233	1.21	PE(P-18:2/22:6)-H	-80	-10	-50	-15	36.71 (13)	75.08 (9)	60.41 (7)
831	483.273	255.233	1	LPG(16:0)-H	-80	-10	-50	-15	4.27 (10)	37.21 (4)	14.5 (14)
832	481.257	253.217	1.01	LPG(16:1)-H	-80	-10	-50	-15	58.63 (12)	38.8 (11)	47.53 (14)

833	511.304	283.264	1.01	LPG(18:0)-H	-80	-10	-50	-15	16.89 (16)	9.02 (13)	12.08 (20)
834	509.289	281.249	1	LPG(18:1)-H	-80	-10	-50	-15	32.84 (17)	19.12 (15)	14.63 (19)
835	507.273	279.233	1.05	LPG(18:2)-H	-80	-10	-50	-15	#DIV/0!	24.46 (12)	16.48 (14)
836	537.32	309.28	1.29	LPG(20:1)-H	-80	-10	-50	-15	60.01 (13)	35.64 (15)	80.65 (11)
837	533.289	305.249	1.81	LPG(20:3)-H	-80	-10	-50	-15	#DIV/0!	62.89 (13)	34.79 (12)
838	531.273	303.233	6.14	LPG(20:4)-H	-80	-10	-50	-15	46.17 (11)	40.76 (13)	52.56 (17)
839	665.44	227.202	1.06	PG(14:0/14:0)-H	-80	-10	-50	-15	#DIV/0!	33.87 (25)	14.22 (32)
840	661.4	225.2	2	PG(14:1/14:1)-H	-80	-10	-50	-15	#DIV/0!	33.82 (12)	28.14 (11)
841	719.487	281.249	1	PG(14:0/18:1)-H	-80	-10	-50	-15	24.38 (29)	10.57 (33)	15.89 (31)
842	717.471	279.233	1.14	PG(14:0/18:2)-H	-80	-10	-50	-15	#DIV/0!	22.02 (21)	23.4 (21)
843	747.518	309.28	1.28	PG(14:0/20:1)-H	-80	-10	-50	-15	#DIV/0!	43.83 (13)	57.03 (11)
844	739.456	301.217	3.18	PG(14:0/20:5)-H	-80	-10	-50	-15	#DIV/0!	56.72 (29)	38.34 (26)
845	769.503	331.264	2.61	PG(14:0/22:4)-H	-80	-10	-50	-15	#DIV/0!	40.1 (18)	#DIV/0!
846	693.471	227.202	1.01	PG(16:0/14:0)-H	-80	-10	-50	-15	7.96 (42)	5.4 (42)	6.51 (37)
847	721.503	255.233	1	PG(16:0/16:0)-H	-80	-10	-50	-15	8.03 (35)	4.53 (43)	7.69 (42)
848	719.487	253.217	1	PG(16:0/16:1)-H	-80	-10	-50	-15	11.9 (40)	4.67 (47)	4.07 (47)
849	749.534	283.264	1	PG(16:0/18:0)-H	-80	-10	-50	-15	17.88 (37)	6.31 (34)	6.21 (42)
850	747.518	281.249	1	PG(16:0/18:1)-H	-80	-10	-50	-15	3.64 (45)	2.37 (53)	1.66 (52)

851	745.503	279.233	1	PG(16:0/18:2)-H	-80	-10	-50	-15	5.3 (45)	4.27 (43)	1.87 (47)
852	743.487	277.217	1.08	PG(16:0/18:3)-H	-80	-10	-50	-15	20.89 (28)	16.99 (30)	27.5 (30)
853	775.549	309.28	1.02	PG(16:0/20:1)-H	-80	-10	-50	-15	17.67 (37)	5.53 (40)	16.72 (35)
854	773.534	307.264	1.01	PG(16:0/20:2)-H	-80	-10	-50	-15	7.41 (38)	8.58 (42)	7.52 (40)
855	771.518	305.249	1.07	PG(16:0/20:3)-H	-80	-10	-50	-15	16.39 (39)	8.66 (43)	10.82 (40)
856	769.503	303.233	1.08	PG(16:0/20:4)-H	-80	-10	-50	-15	9.78 (39)	4.59 (45)	9.12 (30)
857	767.487	301.217	1.45	PG(16:0/20:5)-H	-80	-10	-50	-15	#DIV/0!	37.12 (18)	70.62 (15)
858	795.518	329.249	1.2	PG(16:0/22:5)-H	-80	-10	-50	-15	27.12 (19)	23.39 (29)	33.41 (16)
859	721.503	227.202	1.04	PG(18:0/14:0)-H	-80	-10	-50	-15	29.65 (30)	29.63 (25)	19.1 (31)
860	747.518	253.217	1	PG(18:0/16:1)-H	-80	-10	-50	-15	20.79 (36)	6.84 (43)	10.55 (41)
861	777.565	283.264	1.01	PG(18:0/18:0)-H	-80	-10	-50	-15	8.4 (40)	6.29 (42)	22.67 (37)
862	775.549	281.249	1	PG(18:0/18:1)-H	-80	-10	-50	-15	3.01 (48)	0.98 (47)	2.22 (50)
863	773.534	279.233	1	PG(18:0/18:2)-H	-80	-10	-50	-15	2.83 (49)	3.77 (52)	2.28 (49)
864	771.518	277.217	1.31	PG(18:0/18:3)-H	-80	-10	-50	-15	23.06 (32)	14.1 (38)	16.54 (42)
865	805.596	283.264	1.08	PG(18:0/20:0)-H	-80	-10	-50	-15	#DIV/0!	24.49 (50)	26.56 (51)
866	803.581	309.28	1.03	PG(18:0/20:1)-H	-80	-10	-50	-15	8.52 (40)	5.58 (42)	7.04 (37)
867	801.565	307.264	1.01	PG(18:0/20:2)-H	-80	-10	-50	-15	4.05 (42)	2.83 (46)	8.81 (46)
868	799.549	305.249	1.06	PG(18:0/20:3)-H	-80	-10	-50	-15	8.47 (41)	5.88 (47)	14.73 (36)

869	797.534	303.233	1.05	PG(18:0/20:4)-H	-80	-10	-50	-15	12.86 (39)	7.57 (41)	9.36 (39)
870	795.518	301.217	1.55	PG(18:0/20:5)-H	-80	-10	-50	-15	20.13 (14)	46.73 (19)	#DIV/0!
871	825.565	331.264	1.39	PG(18:0/22:4)-H	-80	-10	-50	-15	#DIV/0!	48.33 (21)	21.39 (21)
872	823.549	329.249	1.47	PG(18:0/22:5)-H	-80	-10	-50	-15	30.36 (20)	53.11 (29)	39.09 (24)
873	745.503	281.249	1	PG(18:1/16:1)-H	-80	-10	-50	-15	10.89 (31)	2.74 (41)	21.4 (31)
874	773.534	281.249	1	PG(18:1/18:1)-H	-80	-10	-50	-15	6.73 (49)	3.68 (46)	5.82 (41)
875	771.518	279.233	1	PG(18:1/18:2)-H	-80	-10	-50	-15	9 (44)	10.04 (42)	16.78 (34)
876	769.503	277.217	1.13	PG(18:1/18:3)-H	-80	-10	-50	-15	#DIV/0!	22.52 (28)	38.86 (31)
877	799.549	307.264	1.2	PG(18:1/20:2)-H	-80	-10	-50	-15	29.57 (22)	22.02 (33)	30.45 (28)
878	797.534	305.249	1.02	PG(18:1/20:3)-H	-80	-10	-50	-15	31.8 (23)	24.99 (28)	34.41 (27)
879	795.518	303.233	1.01	PG(18:1/20:4)-H	-80	-10	-50	-15	28.2 (28)	12.74 (41)	10.68 (28)
880	823.549	331.264	1.18	PG(18:1/22:4)-H	-80	-10	-50	-15	#DIV/0!	17.87 (10)	44.59 (13)
881	821.534	329.249	1.06	PG(18:1/22:5)-H	-80	-10	-50	-15	#DIV/0!	37.93 (19)	64.78 (13)
882	769.503	279.233	1.06	PG(18:2/18:2)-H	-80	-10	-50	-15	18.21 (35)	14.78 (29)	23.95 (21)
883	799.549	309.28	1.07	PG(18:2/20:1)-H	-80	-10	-50	-15	#DIV/0!	33.86 (14)	19.52 (11)
884	797.534	307.264	1.2	PG(18:2/20:2)-H	-80	-10	-50	-15	#DIV/0!	14.7 (15)	62.22 (18)
885	795.518	305.249	2.55	PG(18:2/20:3)-H	-80	-10	-50	-15	54.01 (12)	40.89 (9)	28.05 (8)
886	793.503	303.233	1.04	PG(18:2/20:4)-H	-80	-10	-50	-15	15.83 (20)	25.64 (11)	48.1 (13)

887	803.581	281.249	1	PG(20:0/18:1)-H	-80	-10	- 50	-15	#DIV/0!	54.02 (25)	12.33 (14)
888	801.565	279.233	1.04	PG(20:0/18:2)-H	-80	-10	- 50	-15	#DIV/0!	35.16 (26)	45.79 (10)
889	599.32	283.264	1	LPI(18:0)-H	-80	-10	- 50	-15	15.82 (8)	15.71 (7)	40.41 (7)
890	807.503	281.249	1	PI(14:0/18:1)-H	-80	-10	- 60	-15	15.7 (27)	23.14 (25)	24.66 (7)
891	805.487	279.233	1	PI(14:0/18:2)-H	-80	-10	- 60	-15	26.19 (28)	18.66 (28)	#DIV/0!
892	835.534	309.28	1.11	PI(14:0/20:1)-H	-80	-10	- 60	-15	58.78 (18)	51.33 (19)	121.86 (15)
893	833.518	307.264	1.09	PI(14:0/20:2)-H	-80	-10	- 60	-15	25.83 (16)	60 (17)	16.84 (10)
894	831.503	305.249	1.06	PI(14:0/20:3)-H	-80	-10	- 60	-15	28.45 (26)	26.02 (22)	#DIV/0!
895	829.487	303.233	1.06	PI(14:0/20:4)-H	-80	-10	- 60	-15	9.53 (27)	14.82 (30)	17.17 (7)
896	827.472	301.217	1.18	PI(14:0/20:5)-H	-80	-10	- 60	-15	#DIV/0!	44.4 (20)	33.25 (12)
897	855.503	329.249	1.16	PI(14:0/22:5)-H	-80	-10	- 60	-15	41.5 (22)	#DIV/0!	#DIV/0!
898	809.518	255.233	1	PI(16:0/16:0)-H	-80	-10	- 60	-15	20.95 (21)	16.49 (13)	30.24 (22)
899	807.503	253.217	1	PI(16:0/16:1)-H	-80	-10	- 60	-15	14.11 (11)	18.43 (15)	5.97 (15)
900	837.55	283.264	1	PI(16:0/18:0)-H	-80	-10	- 60	-15	19.8 (20)	9.22 (11)	10.83 (13)
901	835.534	281.249	1	PI(16:0/18:1)-H	-80	-10	- 60	-15	6.06 (13)	2.2 (15)	5.82 (15)
902	833.518	279.233	1	PI(16:0/18:2)-H	-80	-10	- 60	-15	5.47 (13)	5.59 (14)	2.72 (13)
903	814.561	277.218	1	PI(16:0/18:3)-H	-80	-10	- 60	-15	11.9 (25)	4.68 (25)	10.03 (21)
904	863.565	309.28	1.07	PI(16:0/20:1)-H	-80	-10	- 60	-15	11.59 (12)	17.57 (16)	20.71 (16)

905	861.55	307.264	1.03	PI(16:0/20:2)-H	-80	-10	-60	-15	19.6 (12)	5.74 (14)	10.22 (15)
906	859.534	305.249	1	PI(16:0/20:3)-H	-80	-10	-60	-15	8.37 (14)	8.37 (16)	1.17 (19)
907	857.518	303.233	1	PI(16:0/20:4)-H	-80	-10	-60	-15	5.14 (15)	7.39 (17)	5.31 (20)
908	855.503	301.217	1.21	PI(16:0/20:5)-H	-80	-10	-60	-15	48.93 (16)	30.28 (14)	28.61 (17)
909	885.55	331.264	1.11	PI(16:0/22:4)-H	-80	-10	-60	-15	15.52 (13)	27.45 (17)	31.44 (18)
910	883.534	329.249	1.09	PI(16:0/22:5)-H	-80	-10	-60	-15	18.32 (15)	4.36 (19)	10.82 (17)
911	881.518	327.233	1.29	PI(16:0/22:6)-H	-80	-10	-60	-15	25.92 (16)	16.99 (17)	15.77 (18)
912	809.518	227.202	1.26	PI(18:0/14:0)-H	-80	-10	-60	-15	#DIV/0!	39.96 (12)	59.35 (13)
913	835.534	253.217	1	PI(18:0/16:1)-H	-80	-10	-60	-15	7.61 (14)	6.58 (15)	5.04 (19)
914	865.581	283.264	1	PI(18:0/18:0)-H	-80	-10	-60	-15	9.96 (12)	9.06 (12)	14.36 (15)
915	863.565	281.249	1	PI(18:0/18:1)-H	-80	-10	-60	-15	4.44 (14)	2.2 (13)	9.64 (15)
916	861.55	279.233	1	PI(18:0/18:2)-H	-80	-10	-60	-15	1.06 (12)	7.36 (14)	4.85 (16)
917	893.612	283.264	1	PI(18:0/20:0)-H	-80	-10	-60	-15	#DIV/0!	20.03 (16)	13.06 (17)
918	891.597	309.28	1.03	PI(18:0/20:1)-H	-80	-10	-60	-15	31.27 (15)	21.13 (16)	13.39 (16)
919	889.581	307.264	1	PI(18:0/20:2)-H	-80	-10	-60	-15	4.22 (17)	7.5 (18)	7.34 (20)
920	887.565	305.249	1	PI(18:0/20:3)-H	-80	-10	-60	-15	4.47 (20)	6.31 (18)	4.95 (22)
921	885.55	283.3	1	PI(18:0/20:4)-H	-80	-10	-60	-15	3.96 (16)	1.08 (15)	2.8 (17)
922	883.534	301.217	1.13	PI(18:0/20:5)-H	-80	-10	-60	-15	12.74 (15)	14.41 (17)	5.71 (15)

923	913.581	331.264	1.02	PI(18:0/22:4)-H	-80	-10	-60	-15	32.47 (15)	21.91 (15)	9.77 (14)
924	911.565	329.249	1.01	PI(18:0/22:5)-H	-80	-10	-60	-15	4.72 (14)	6.05 (15)	12.22 (16)
925	909.55	327.233	1.05	PI(18:0/22:6)-H	-80	-10	-60	-15	22.49 (15)	7.63 (17)	10.15 (18)
926	833.518	281.249	1	PI(18:1/16:1)-H	-80	-10	-60	-15	16.8 (21)	12.72 (22)	11.43 (22)
927	861.55	281.249	1	PI(18:1/18:1)-H	-80	-10	-60	-15	7.72 (12)	7.71 (12)	8.17 (14)
928	859.534	279.233	1	PI(18:1/18:2)-H	-80	-10	-60	-15	13.04 (14)	14.14 (13)	4.91 (15)
929	889.581	309.28	1.05	PI(18:1/20:1)-H	-80	-10	-60	-15	15.55 (16)	18.68 (15)	19.58 (17)
930	887.565	307.264	1.03	PI(18:1/20:2)-H	-80	-10	-60	-15	31.3 (17)	41.05 (17)	18.8 (18)
931	885.55	305.249	1.02	PI(18:1/20:3)-H	-80	-10	-60	-15	4.29 (18)	4.34 (14)	12.06 (13)
932	883.534	303.233	1	PI(18:1/20:4)-H	-80	-10	-60	-15	9.38 (14)	10.2 (14)	2.81 (15)
933	881.518	301.217	1.17	PI(18:1/20:5)-H	-80	-10	-60	-15	64.3 (13)	45.44 (15)	35.73 (14)
934	909.55	329.249	1.12	PI(18:1/22:5)-H	-80	-10	-60	-15	26.8 (15)	33.4 (17)	24.35 (20)
935	907.534	327.233	1.16	PI(18:1/22:6)-H	-80	-10	-60	-15	43.76 (14)	34.74 (10)	23.18 (14)
936	831.503	279.233	1	PI(18:2/16:1)-H	-80	-10	-60	-15	18.39 (25)	14.02 (22)	5.9 (22)
937	857.518	279.233	1	PI(18:2/18:2)-H	-80	-10	-60	-15	35.43 (19)	14.16 (11)	18.15 (14)
938	855.503	277.217	1.08	PI(18:2/18:3)-H	-80	-10	-60	-15	31.12 (15)	83.56 (13)	37.63 (13)
939	887.565	309.28	1.04	PI(18:2/20:1)-H	-80	-10	-60	-15	9.05 (15)	30.38 (14)	16.34 (19)
940	883.534	305.249	1.05	PI(18:2/20:3)-H	-80	-10	-60	-15	36.71 (22)	34.2 (22)	20.02 (18)

941	881.518	303.233	1.07	PI(18:2/20:4)-H	-80	-10	- 60	-15	27.65 (26)	22.26 (34)	26.87 (14)
942	907.534	329.249	1.13	PI(18:2/22:5)-H	-80	-10	- 60	-15	37.21 (18)	63.78 (16)	36 (9)
943	905.518	327.233	1.14	PI(18:2/22:6)-H	-80	-10	- 60	-15	82.19 (13)	47.04 (12)	74.19 (11)
944	863.565	253.217	1.03	PI(20:0/16:1)-H	-80	-10	- 60	-15	21.36 (13)	35.1 (17)	32.25 (17)
945	891.597	281.249	1	PI(20:0/18:1)-H	-80	-10	- 60	-15	14.66 (16)	17.9 (16)	24.5 (18)
946	889.581	279.233	1.02	PI(20:0/18:2)-H	-80	-10	- 60	-15	29.64 (28)	20.13 (30)	31.45 (13)
947	887.565	277.217	1.09	PI(20:0/18:3)-H	-80	-10	- 60	-15	47.96 (11)	66.77 (15)	19.68 (16)
948	919.628	309.28	1.06	PI(20:0/20:1)-H	-80	-10	- 60	-15	37.41 (20)	32.92 (19)	25.17 (10)
949	917.612	307.264	1.12	PI(20:0/20:2)-H	-80	-10	- 60	-15	23.8 (14)	56.22 (14)	64 (11)
950	915.597	305.249	1.24	PI(20:0/20:3)-H	-80	-10	- 60	-15	24.4 (15)	23.08 (17)	27.98 (14)
951	913.581	303.233	1.06	PI(20:0/20:4)-H	-80	-10	- 60	-15	26.37 (15)	13.77 (16)	9.6 (12)
952	496.268	255.233	1.08	LPS(16:0)-H	-80	-10	- 50	-15	18.42 (18)	24.02 (13)	26.68 (18)
953	522.284	281.249	1.32	LPS(18:1)-H	-80	-10	- 50	-15	73.89 (11)	29.95 (16)	41.16 (15)
954	520.268	279.233	1.65	LPS(18:2)-H	-80	-10	- 50	-15	45.81 (12)	18.63 (21)	71.98 (13)
955	544.268	303.233	4.95	LPS(20:4)-H	-80	-10	- 50	-15	112.13 (17)	52.63 (13)	30.38 (10)
956	542.252	301.217	5.32	LPS(20:5)-H	-80	-10	- 50	-15	30.09 (18)	34.52 (18)	66.31 (10)
957	678.435	227.202	1.12	PS(14:0/14:0)-H	-80	-10	- 51	-15	4.22 (15)	29.15 (12)	79.07 (14)
958	730.466	279.233	1.01	PS(14:0/18:2)-H	-80	-10	- 50	-15	11.69 (20)	16.15 (23)	16.43 (20)

959	760.513	309.28	1.06	PS(14:0/20:1)-H	-80	-10	-50	-15	65.68 (19)	46.05 (26)	45.06 (25)
960	758.498	307.264	1.17	PS(14:0/20:2)-H	-80	-10	-50	-15	24.14 (20)	54.47 (23)	54.73 (15)
961	756.482	305.249	1.1	PS(14:0/20:3)-H	-80	-10	-50	-15	12.42 (20)	30.54 (23)	18.07 (28)
962	754.466	303.233	1.08	PS(14:0/20:4)-H	-80	-10	-50	-15	9.04 (24)	13.95 (25)	6.6 (25)
963	782.498	331.264	1.2	PS(14:0/22:4)-H	-80	-10	-50	-15	26.77 (21)	30.08 (24)	22.66 (22)
964	780.482	329.249	1.34	PS(14:0/22:5)-H	-80	-10	-50	-15	25.89 (27)	15.15 (29)	10.97 (30)
965	778.466	327.233	1.23	PS(14:0/22:6)-H	-80	-10	-50	-15	24.34 (24)	35.74 (24)	27.19 (22)
966	706.466	227.202	1.08	PS(16:0/14:0)-H	-80	-10	-50	-15	16.25 (17)	31.97 (18)	41.89 (20)
967	734.498	255.233	1	PS(16:0/16:0)-H	-80	-10	-50	-15	19.76 (26)	18.98 (24)	9.67 (27)
968	732.482	253.217	1.02	PS(16:0/16:1)-H	-80	-10	-50	-15	22.22 (27)	24.55 (27)	24.79 (20)
969	760.513	283.264	1.04	PS(16:0/18:0)-H	-80	-10	-50	-15	#DIV/0!	73.66 (7)	38.23 (8)
970	760.513	281.249	1	PS(16:0/18:1)-H	-80	-10	-50	-15	11.72 (33)	10.69 (36)	6.29 (33)
971	758.498	279.233	1	PS(16:0/18:2)-H	-80	-10	-50	-15	3.27 (30)	8.33 (29)	11.06 (20)
972	756.482	277.217	1.04	PS(16:0/18:3)-H	-80	-10	-50	-15	31.24 (18)	13.31 (21)	34.8 (9)
973	788.545	309.28	1.03	PS(16:0/20:1)-H	-80	-10	-50	-15	25.91 (18)	20.35 (30)	29.41 (18)
974	786.529	307.264	1.04	PS(16:0/20:2)-H	-80	-10	-50	-15	39.51 (18)	21.6 (39)	31.9 (27)
975	784.513	305.249	1.03	PS(16:0/20:3)-H	-80	-10	-50	-15	8.46 (26)	14.8 (20)	15.56 (20)
976	782.498	303.233	1.04	PS(16:0/20:4)-H	-80	-10	-50	-15	15.72 (31)	17.69 (26)	19.52 (25)

977	780.482	301.217	1.25	PS(16:0/20:5)-H	-80	-10	-50	-15	47.34 (25)	73.07 (13)	63.42 (13)
978	810.529	331.264	1.28	PS(16:0/22:4)-H	-80	-10	-50	-15	40.06 (24)	65.96 (23)	98.65 (8)
979	808.513	329.249	1.33	PS(16:0/22:5)-H	-80	-10	-50	-15	30.14 (30)	29.71 (24)	34.63 (22)
980	734.498	227.202	1.07	PS(18:0/14:0)-H	-80	-10	-50	-15	12.82 (18)	49.57 (21)	28.62 (23)
981	760.513	253.217	1.02	PS(18:0/16:1)-H	-80	-10	-50	-15	11.46 (24)	40.5 (27)	11.54 (24)
982	790.56	283.264	1	PS(18:0/18:0)-H	-80	-10	-50	-15	0.8 (31)	4.8 (33)	2.5 (30)
983	788.545	281.249	1	PS(18:0/18:1)-H	-80	-10	-50	-15	6.65 (28)	5.25 (31)	6.36 (33)
984	786.529	279.233	1	PS(18:0/18:2)-H	-80	-10	-50	-15	11.13 (25)	11.99 (25)	15.12 (19)
985	784.513	277.217	1	PS(18:0/18:3)-H	-80	-10	-50	-15	13.26 (9)	45.94 (11)	30.1 (7)
986	818.592	283.264	1	PS(18:0/20:0)-H	-80	-10	-50	-15	3.64 (33)	5.01 (31)	9.8 (30)
987	816.576	309.28	1.01	PS(18:0/20:1)-H	-80	-10	-50	-15	19.94 (23)	11.95 (32)	26.07 (27)
988	814.56	307.264	1.01	PS(18:0/20:2)-H	-80	-10	-50	-15	35.95 (20)	35.6 (29)	29.92 (26)
989	812.545	305.249	1.01	PS(18:0/20:3)-H	-80	-10	-50	-15	24.01 (22)	38.15 (25)	4.86 (29)
990	810.529	303.233	1.02	PS(18:0/20:4)-H	-80	-10	-50	-15	4.79 (29)	6.51 (27)	10.98 (25)
991	838.56	331.264	1.06	PS(18:0/22:4)-H	-80	-10	-50	-15	31.17 (16)	50.54 (27)	37.05 (16)
992	834.529	327.233	1.14	PS(18:0/22:6)-H	-80	-10	-50	-15	64.37 (13)	68.76 (10)	70.02 (8)
993	758.498	281.249	1	PS(18:1/16:1)-H	-80	-10	-50	-15	3.65 (25)	5.87 (25)	3.55 (23)
994	786.529	281.249	1	PS(18:1/18:1)-H	-80	-10	-50	-15	8.93 (27)	4.91 (32)	3.56 (33)

995	784.513	279.233	1	PS(18:1/18:2)-H	-80	-10	-50	-15	8.17 (23)	3 (28)	8.06 (25)
996	782.498	277.217	1.05	PS(18:1/18:3)-H	-80	-10	-50	-15	38.51 (20)	57.54 (19)	30.11 (13)
997	814.56	309.28	1.01	PS(18:1/20:1)-H	-80	-10	-50	-15	21.65 (21)	25.25 (30)	24.21 (29)
998	812.545	307.264	1.01	PS(18:1/20:2)-H	-80	-10	-50	-15	63.45 (19)	23.66 (26)	24.95 (28)
999	810.529	305.249	1.01	PS(18:1/20:3)-H	-80	-10	-50	-15	10.22 (23)	14.47 (24)	19.02 (24)
1000	808.513	303.233	1.01	PS(18:1/20:4)-H	-80	-10	-50	-15	2.16 (28)	9.87 (36)	11.39 (34)
1001	806.498	301.217	1.21	PS(18:1/20:5)-H	-80	-10	-50	-15	25.76 (23)	45.23 (29)	9.94 (31)
1002	836.545	331.264	1.11	PS(18:1/22:4)-H	-80	-10	-50	-15	28.84 (26)	30.08 (23)	37.78 (27)
1003	834.529	329.249	1.09	PS(18:1/22:5)-H	-80	-10	-50	-15	13.84 (30)	20.16 (30)	13.04 (24)
1004	832.513	327.233	1.1	PS(18:1/22:6)-H	-80	-10	-50	-15	24.52 (23)	61.4 (20)	20.22 (30)
1005	756.482	279.233	1	PS(18:2/16:1)-H	-80	-10	-50	-15	4.04 (22)	3.84 (23)	5.86 (24)
1006	782.498	279.233	1	PS(18:2/18:2)-H	-80	-10	-50	-15	5.79 (32)	4.39 (33)	2.61 (32)
1007	780.482	277.217	1.11	PS(18:2/18:3)-H	-80	-10	-50	-15	24.64 (22)	26.85 (25)	24.31 (29)
1008	812.545	309.28	1.01	PS(18:2/20:1)-H	-80	-10	-50	-15	34.79 (23)	22.23 (30)	34.5 (24)
1009	810.529	307.264	1.02	PS(18:2/20:2)-H	-80	-10	-50	-15	16.24 (23)	32.95 (25)	24.58 (20)
1010	808.513	305.249	1.04	PS(18:2/20:3)-H	-80	-10	-50	-15	5.95 (29)	11.99 (30)	7.51 (33)
1011	806.498	303.233	1.02	PS(18:2/20:4)-H	-80	-10	-50	-15	6.75 (33)	4.76 (33)	3.75 (32)
1012	804.482	301.217	1.56	PS(18:2/20:5)-H	-80	-10	-50	-15	26.67 (22)	24.71 (32)	25.85 (27)

1013	834.529	331.264	1.14	PS(18:2/22:4)-H	-80	-10	-50	-15	25.83 (23)	11.9 (28)	21.19 (26)
1014	832.513	329.249	1.15	PS(18:2/22:5)-H	-80	-10	-50	-15	5.47 (28)	11.08 (32)	11.28 (31)
1015	830.498	327.233	1.21	PS(18:2/22:6)-H	-80	-10	-50	-15	33.29 (18)	43.52 (14)	44.36 (9)
1016	788.545	253.217	1	PS(20:0/16:1)-H	-80	-10	-50	-15	21.4 (22)	20.25 (18)	47.28 (12)
1017	814.56	281.249	1	PS(20:0/18:1)-H	-80	-10	-50	-15	15.64 (17)	19.92 (20)	42.26 (18)
1018	814.561	279.234	1	PS(20:0/18:2)-H	-80	-10	-50	-15	3.64 (26)	4.75 (27)	10.27 (23)
1019	812.545	277.217	1.02	PS(20:0/18:3)-H	-80	-10	-50	-15	38.88 (21)	52.85 (19)	9.94 (26)
1020	844.607	309.28	1.01	PS(20:0/20:1)-H	-80	-10	-50	-15	27.49 (32)	12.57 (30)	12.03 (34)
1021	842.592	307.264	1.01	PS(20:0/20:2)-H	-80	-10	-50	-15	30.49 (19)	24.25 (23)	13.52 (22)
1022	840.576	305.249	1	PS(20:0/20:3)-H	-80	-10	-50	-15	12.83 (25)	14.03 (24)	13.52 (24)
1023	838.56	303.233	1.01	PS(20:0/20:4)-H	-80	-10	-50	-15	12.87 (33)	7.35 (32)	7.93 (35)
1024	836.545	301.217	1.06	PS(20:0/20:5)-H	-80	-10	-50	-15	63.05 (20)	62.8 (18)	30.22 (21)
1025	866.592	331.264	1.17	PS(20:0/22:4)-H	-80	-10	-50	-15	50.14 (26)	29.6 (26)	30.89 (26)
1026	864.576	329.249	9	PS(20:0/22:5)-H	-80	-10	-50	-15	3.99 (35)	6.75 (34)	5.06 (35)
1027	862.56	327.233	1	PS(20:0/22:6)-H	-80	-10	-50	-15	44 (24)	27.59 (30)	30.99 (26)
1028	645.45	281.249	1.07	PA(14:0/18:1)-H	-80	-10	-50	-15	#DIV/0!	55.7 (24)	66.54 (16)
1029	619.434	227.202	1.04	PA(16:0/14:0)-H	-80	-10	-50	-15	#DIV/0!	53.7 (14)	41.39 (13)
1030	647.466	255.233	1	PA(16:0/16:0)-H	-80	-10	-50	-15	7.89 (38)	10.47 (34)	14.02 (33)

1031	675.497	283.264	1	PA(16:0/18:0)-H	-80	-10	-50	-15	56.78 (20)	15.39 (36)	8.17 (35)
1032	673.481	281.249	1	PA(16:0/18:1)-H	-80	-10	-50	-15	9.32 (19)	15.27 (44)	13.77 (49)
1033	671.466	279.233	1	PA(16:0/18:2)-H	-80	-10	-50	-15	17.33 (19)	12.51 (19)	11.35 (20)
1034	701.513	309.28	1	PA(16:0/20:1)-H	-80	-10	-50	-15	#DIV/0!	42.05 (17)	58.05 (20)
1035	699.497	307.264	1	PA(16:0/20:2)-H	-80	-10	-50	-15	#DIV/0!	50.32 (22)	46.44 (17)
1036	697.481	305.249	1.04	PA(16:0/20:3)-H	-80	-10	-50	-15	48.37 (16)	49.62 (14)	59.53 (17)
1037	695.466	303.233	1.47	PA(16:0/20:4)-H	-80	-10	-50	-15	35.06 (19)	32.92 (12)	67.01 (11)
1038	723.497	331.264	1.03	PA(16:0/22:4)-H	-80	-10	-50	-15	#DIV/0!	53.66 (18)	88.68 (16)
1039	721.481	329.249	1	PA(16:0/22:5)-H	-80	-10	-50	-15	58.48 (16)	26.92 (23)	28.15 (20)
1040	719.466	327.233	1	PA(16:0/22:6)-H	-80	-10	-50	-15	8.66 (10)	94.51 (15)	58.99 (10)
1041	703.528	283.264	1	PA(18:0/18:0)-H	-80	-10	-50	-15	24.04 (17)	15 (31)	26.6 (25)
1042	701.513	281.249	1	PA(18:0/18:1)-H	-80	-10	-50	-15	4.27 (24)	8.16 (27)	4.19 (23)
1043	699.497	279.233	1	PA(18:0/18:2)-H	-80	-10	-50	-15	4.63 (26)	4.02 (32)	3.24 (31)
1044	697.481	277.217	1.06	PA(18:0/18:3)-H	-80	-10	-50	-15	28.79 (19)	13.14 (21)	13.95 (25)
1045	731.56	283.264	1	PA(18:0/20:0)-H	-80	-10	-50	-15	29.65 (22)	13.43 (29)	36.46 (24)
1046	729.544	309.28	1	PA(18:0/20:1)-H	-80	-10	-50	-15	20.78 (21)	7.5 (29)	15.88 (31)
1047	727.528	307.264	1	PA(18:0/20:2)-H	-80	-10	-50	-15	26.93 (23)	30.22 (30)	8.94 (27)
1048	725.513	305.249	1	PA(18:0/20:3)-H	-80	-10	-50	-15	5.12 (25)	6.92 (30)	3.65 (29)

1049	723.497	303.233	1	PA(18:0/20:4)-H	-80	-10	- 50	-15	3.32 (23)	3.6 (22)	56.27 (5)
1050	721.481	301.217	2.44	PA(18:0/20:5)-H	-80	-10	- 50	-15	5.06 (25)	9.32 (28)	#DIV/0!
1051	751.528	331.264	1	PA(18:0/22:4)-H	-80	-10	- 50	-15	6.3 (20)	5.09 (22)	#DIV/0!
1052	749.513	329.249	1.11	PA(18:0/22:5)-H	-80	-10	- 50	-15	50.47 (16)	63.28 (10)	68.88 (14)
1053	747.497	327.233	1.14	PA(18:0/22:6)-H	-80	-10	- 50	-15	5.69 (26)	10.48 (33)	59.09 (7)
1054	671.466	281.249	1	PA(18:1/16:1)-H	-80	-10	- 50	-15	19.85 (20)	16.74 (31)	16.57 (7)
1055	699.497	281.249	1	PA(18:1/18:1)-H	-80	-10	- 50	-15	11.3 (22)	5.92 (23)	24.14 (12)
1056	697.481	279.233	1	PA(18:1/18:2)-H	-80	-10	- 50	-15	14.4 (25)	6.2 (21)	83.33 (5)
1057	695.466	277.217	1	PA(18:1/18:3)-H	-80	-10	- 50	-15	26.5 (16)	25.72 (21)	#DIV/0!
1058	727.528	309.28	1	PA(18:1/20:1)-H	-80	-10	- 50	-15	20.33 (20)	23.18 (32)	27.94 (29)
1059	725.513	307.264	1	PA(18:1/20:2)-H	-80	-10	- 50	-15	18.46 (20)	19.96 (20)	17.8 (7)
1060	723.497	305.249	1	PA(18:1/20:3)-H	-80	-10	- 50	-15	18.06 (24)	8.79 (23)	36.2 (6)
1061	721.481	303.233	1	PA(18:1/20:4)-H	-80	-10	- 50	-15	13.11 (21)	7.1 (21)	33.86 (5)
1062	719.466	301.217	1.95	PA(18:1/20:5)-H	-80	-10	- 50	-15	48.63 (15)	29.04 (24)	65.14 (7)
1063	749.513	331.264	1.02	PA(18:1/22:4)-H	-80	-10	- 50	-15	15.81 (22)	30.94 (21)	97.92 (8)
1064	747.497	329.249	1.08	PA(18:1/22:5)-H	-80	-10	- 50	-15	33.56 (23)	10.85 (20)	22.96 (6)
1065	745.481	327.233	1	PA(18:1/22:6)-H	-80	-10	- 50	-15	40.24 (22)	19.58 (20)	26.35 (9)
1066	669.45	279.233	1	PA(18:2/16:1)-H	-80	-10	- 50	-15	37.58 (17)	29.16 (20)	44.86 (6)

1067	695.466	279.233	1	PA(18:2/18:2)-H	-80	-10	-50	-15	23.23 (22)	15.52 (48)	#DIV/0!
1068	725.513	309.28	1.17	PA(18:2/20:1)-H	-80	-10	-50	-15	81.91 (13)	56.66 (15)	34.54 (24)
1069	721.481	305.249	1.36	PA(18:2/20:3)-H	-80	-10	-50	-15	88.9 (15)	20.8 (17)	35.47 (13)
1070	719.466	303.233	1.88	PA(18:2/20:4)-H	-80	-10	-50	-15	49.28 (18)	69.32 (18)	15.89 (16)
1071	743.466	327.233	3.06	PA(18:2/22:6)-H	-80	-10	-50	-15	11.99 (21)	32.31 (10)	35.22 (12)
1072	701.513	253.217	1	PA(20:0/16:1)-H	-80	-10	-50	-15	17.09 (24)	9.05 (31)	7.38 (30)
1073	729.544	281.249	1	PA(20:0/18:1)-H	-80	-10	-50	-15	4.2 (31)	6.76 (32)	1.48 (34)
1074	727.528	279.233	1	PA(20:0/18:2)-H	-80	-10	-50	-15	4.17 (30)	3.5 (31)	1.18 (36)
1075	725.513	277.217	1.28	PA(20:0/18:3)-H	-80	-10	-50	-15	20.48 (24)	6.16 (30)	12.72 (29)
1076	757.575	309.28	1	PA(20:0/20:1)-H	-80	-10	-50	-15	19.21 (26)	10.51 (33)	10.11 (34)
1077	755.56	307.264	1	PA(20:0/20:2)-H	-80	-10	-50	-15	15.87 (22)	21.6 (27)	15.47 (33)
1078	753.544	305.249	1	PA(20:0/20:3)-H	-80	-10	-50	-15	4.96 (30)	9.08 (28)	5.47 (35)
1079	751.528	303.233	1	PA(20:0/20:4)-H	-80	-10	-50	-15	3.47 (29)	3.33 (33)	1.1 (34)
1080	749.513	301.217	1.55	PA(20:0/20:5)-H	-80	-10	-50	-15	6.66 (25)	12.29 (24)	3.76 (29)
1081	779.56	331.264	1	PA(20:0/22:4)-H	-80	-10	-50	-15	7.61 (23)	9.17 (22)	2 (20)
1082	777.544	329.249	1	PA(20:0/22:5)-H	-80	-10	-50	-15	5.73 (25)	6.75 (27)	4.27 (31)
1083	775.528	327.233	1.26	PA(20:0/22:6)-H	-80	-10	-50	-15	4.59 (23)	8.38 (22)	3.43 (22)

Supplementary- Table S2: The reproducibility of retention time and % CV for RT (n=5, technical replicates) table for 3 different days.

S. No.	Lipid species	Day 1 rep1	Day 1 rep2	Day 1 rep3	Day 1 rep4	Day 1 rep5	Day 1 CV	Day 2 rep1	Day 2 rep2	Day 2 rep3	Day 2 rep4	Day 2 rep5	Day 2 CV	Day 3 rep1	Day 3 rep2	Day 3 rep3	Day 3 rep4	Day 3 rep5	Day 3 CV
1	SM(14:0)+H	12.31	12.28	12.31	12.30	12.30	0.09	12.20	12.22	12.19	12.21	12.19	0.09	12.05	12.09	12.08	12.07	12.04	0.15
2	SM(16:0)+H	12.22	12.20	12.22	12.21	12.21	0.07	12.11	12.14	12.10	12.11	12.11	0.14	11.96	11.98	11.97	11.97	11.94	0.14
3	SM(18:0)+H	12.13	12.13	12.15	12.14	12.14	0.08	12.01	12.04	11.99	12.00	12.01	0.16	11.86	11.89	11.87	11.86	11.85	0.12
4	SM(18:1)+H	12.13	12.12	12.15	12.14	12.14	0.08	12.02	12.05	12.01	12.02	12.02	0.12	11.86	11.89	11.88	11.87	11.85	0.14
5	SM(20:0)+H	12.11	12.10	12.13	12.11	12.11	0.10	11.94	11.97	11.92	11.93	11.96	0.17	11.80	11.83	11.82	11.80	11.78	0.15
6	SM(20:1)+H	12.10	12.08	12.10	12.10	12.09	0.08	11.94	11.98	11.92	11.94	11.95	0.19	11.79	11.81	11.81	11.78	11.78	0.15
7	SM(22:0)+H	12.05	12.03	12.05	12.06	12.05	0.07	11.92	11.94	11.91	11.93	11.93	0.08	11.81	11.82	11.83	11.81	11.81	0.08
8	SM(22:1)+H	12.04	12.02	12.04	12.05	12.04	0.10	11.91	11.93	11.90	11.91	11.92	0.08	11.79	11.81	11.81	11.80	11.80	0.08
9	SM(24:0)+H	11.98	11.96	11.97	11.97	11.97	0.08	11.86	11.87	11.84	11.86	11.86	0.08	11.74	11.76	11.76	11.75	11.74	0.08
10	SM(24:1)+H	11.99	11.97	11.98	11.99	11.98	0.06	11.86	11.88	11.85	11.87	11.87	0.09	11.74	11.76	11.76	11.75	11.73	0.12
11	SM(26:0)+H	12.36	12.37	12.41	12.38	12.40	0.18	12.05	12.08	12.04	12.08	12.06	0.13	11.93	11.96	11.94	11.93	11.95	0.12
12	SM(26:1)+H	11.97	11.93	11.96	11.98	11.96	0.17	11.87	11.89	11.86	11.86	11.87	0.12	11.73	11.77	11.74	11.74	11.73	0.13
13	CE(24:0)+NH4	2.44	2.38	2.41	2.37	2.42	1.13	2.43	2.39	2.37	2.52	2.38	2.54	2.36	2.43	2.36	2.35	2.35	1.39
14	CE(22:6)+NH4	2.39	2.37	2.42	2.41	2.41	0.93	2.38	2.38	2.43	2.45	2.40	1.28	2.36	2.45	2.41	2.37	2.36	1.68
15	CE(20:0)+NH4	2.35	2.35	2.41	2.41	2.33	1.68	2.46	2.37	2.43	2.48	2.42	1.62	2.37	2.42	2.37	2.35	2.35	1.23
16	CE(20:1)+NH4	2.32	2.29	2.34	2.34	2.29	1.08	2.47	2.29	2.35	2.40	2.37	2.83	2.34	2.28	2.32	2.32	2.33	0.92
17	CE(22:5)+NH4	2.62	2.69	2.58	2.66	2.64	1.57	2.61	2.55	2.56	2.55	2.57	1.06	2.42	2.53	2.52	2.51	2.48	1.80
18	CE(14:0)+NH4	2.41	2.50	2.52	2.55	2.51	2.18	2.53	2.52	2.49	2.53	2.50	0.71	2.49	2.55	2.53	2.46	2.52	1.40
19	CE(16:0)+NH4	2.44	2.50	2.54	2.49	2.40	2.28	2.52	2.52	2.43	2.61	2.53	2.49	2.46	2.47	2.51	2.53	2.47	1.23
20	CE(16:1)+NH4	2.41	2.33	2.87	2.43	2.53	8.32	2.44	2.61	2.50	2.52	2.56	2.42	2.52	2.70	2.50	2.33	2.63	5.56
21	CE(18:0)+NH4	2.36	2.41	2.38	2.48	2.36	2.05	2.43	2.43	2.44	2.43	2.43	0.31	2.44	2.45	2.36	2.39	2.45	1.75
22	CE(18:1)+NH4	2.50	2.50	2.49	2.50	2.44	1.04	2.61	2.64	2.55	2.52	2.51	2.25	2.50	2.51	2.56	2.50	2.46	1.55
23	CE(18:2)+NH4	2.41	2.43	2.49	2.47	2.44	1.36	2.45	2.50	2.47	2.47	2.53	1.26	2.42	2.53	2.40	2.47	2.43	1.98

24	CE(18:3)+NH4	2.39	2.43	2.44	2.41	2.41	0.77	2.37	2.39	2.42	2.44	2.41	1.13	2.39	2.37	2.45	2.40	2.38	1.36
25	CE(20:2)+NH4	2.41	2.38	2.45	2.43	2.36	1.44	2.41	2.43	2.44	2.45	2.46	0.75	2.41	2.46	2.42	2.43	2.44	0.82
26	CE(20:3)+NH4	2.39	2.45	2.42	2.48	2.38	1.76	2.56	2.55	2.54	2.61	2.57	0.96	2.53	2.50	2.55	2.52	2.52	0.63
27	CE(20:4)+NH4	2.40	2.39	2.43	2.45	2.38	1.25	2.43	2.47	2.50	2.44	2.46	1.17	2.46	2.47	2.41	2.45	2.44	0.88
28	CE(20:5)+NH4	2.30	2.39	2.40	2.44	2.33	2.39	2.42	2.48	2.43	2.37	2.43	1.66	2.42	2.48	2.41	2.37	2.33	2.38
29	CE(22:0)+NH4	2.42	2.46	2.34	2.46	2.43	2.07	2.43	2.43	2.39	2.40	2.40	0.80	2.38	2.48	2.39	2.42	2.42	1.58
30	CE(22:1)+NH4	2.40	2.36	2.43	2.45	2.44	1.61	2.51	2.47	2.45	2.49	2.48	0.94	2.35	2.51	2.46	2.32	2.45	3.31
31	CE(22:2)+NH4	2.39	2.43	2.43	2.45	2.44	0.99	2.40	2.45	2.47	2.50	2.36	2.33	2.49	2.41	2.45	2.38	2.44	1.75
32	CE(22:4)+NH4	2.34	2.46	2.43	2.57	2.42	3.31	2.51	2.54	2.52	2.56	2.55	0.82	2.51	2.47	2.50	2.52	2.53	0.95
33	CE(24:1)+NH4	2.96	2.86	2.92	2.99	2.99	1.92	2.72	2.71	2.74	2.66	2.59	2.25	2.65	2.62	2.62	2.60	2.64	0.76
34	CER(14:0)+H	2.61	2.59	2.59	2.61	2.55	0.86	2.62	2.67	2.61	2.60	2.72	1.92	2.57	2.65	2.58	2.49	2.57	2.24
35	CER(16:0)+H	2.60	2.59	2.60	2.59	2.59	0.29	2.58	2.55	2.54	2.55	2.57	0.57	2.54	2.53	2.49	2.53	2.57	1.14
36	CER(18:0)+H	2.58	2.56	2.59	2.55	2.62	0.98	2.58	2.51	2.52	2.51	2.50	1.28	2.52	2.53	2.51	2.52	2.58	1.15
37	CER(18:1)+H	2.70	2.68	2.72	2.73	2.73	0.77	2.67	2.63	2.67	2.70	2.71	1.14	2.69	2.66	2.65	2.67	2.67	0.62
38	CER(20:0)+H	2.60	2.51	2.57	2.55	2.63	1.86	2.50	2.46	2.51	2.55	2.50	1.33	2.53	2.48	2.54	2.52	2.54	1.02
39	CER(20:1)+H	2.68	2.68	2.67	2.69	2.64	0.72	2.64	2.67	2.67	2.67	2.68	0.63	2.63	2.64	2.59	2.64	2.64	0.80
40	CER(22:0)+H	2.57	2.51	2.54	2.53	2.54	0.94	2.54	2.54	2.52	2.49	2.53	0.75	2.48	2.48	2.51	2.55	2.51	1.22
41	CER(22:1)+H	2.56	2.60	2.60	2.57	2.55	0.84	2.57	2.57	2.56	2.60	2.58	0.60	2.59	2.59	2.57	2.59	2.56	0.48
42	CER(24:0)+H	2.52	2.54	2.51	2.53	2.49	0.79	2.54	2.53	2.53	2.49	2.53	0.76	2.54	2.54	2.50	2.54	2.52	0.73
43	CER(24:1)+H	2.56	2.57	2.58	2.56	2.54	0.61	2.54	2.56	2.56	2.56	2.53	0.54	2.56	2.54	2.58	2.58	2.57	0.57
44	CER(26:0)+H	2.61	2.63	2.61	2.61	2.59	0.42	2.59	2.57	2.64	2.61	2.60	0.96	2.60	2.60	2.61	2.63	2.56	1.00
45	CER(26:1)+H	2.67	2.67	2.66	2.66	2.67	0.27	2.66	2.65	2.65	2.66	2.65	0.20	2.64	2.64	2.61	2.62	2.64	0.59
46	DCER(14:0)+H	2.60	2.60	2.79	2.69	2.62	3.12	2.55	2.63	2.58	2.49	3.02	7.87	2.52	2.53	2.74	2.58	N/A	3.90
47	DCER(16:0)+H	2.52	2.55	2.56	2.55	2.52	0.64	2.52	2.52	2.53	2.52	2.53	0.22	2.54	2.53	2.51	2.53	2.51	0.52
48	DCER(18:0)+H	2.52	2.52	2.53	2.56	2.53	0.59	2.48	2.52	2.54	2.49	2.55	1.16	2.52	2.50	2.52	2.52	2.50	0.53
49	DCER(18:1)+H	2.71	2.48	2.63	2.47	2.40	5.02	2.55	2.54	2.68	2.55	2.60	2.29	2.63	2.66	2.67	2.55	2.60	1.82
50	DCER(20:0)+H	2.54	2.51	2.54	2.53	2.55	0.64	2.49	2.49	2.50	2.50	2.50	0.22	2.54	2.51	2.50	2.53	2.49	0.83
51	DCER(20:1)+H	2.44	2.48	2.44	2.59	2.62	3.45	2.55	2.73	2.61	2.52	2.63	3.25	2.64	2.57	2.83	2.66	2.60	3.80
52	DCER(22:0)+H	2.53	2.55	2.51	2.51	2.51	0.69	2.49	2.53	2.51	2.50	2.52	0.68	2.47	2.47	2.51	2.51	2.46	0.99

53	DCER(22:1)+H	2.51	2.54	2.53	2.52	2.52	0.51	2.52	2.46	2.49	2.53	2.52	1.13	2.51	2.48	2.50	2.53	2.48	0.83
54	DCER(24:0)+H	2.51	2.54	2.52	2.51	2.52	0.54	2.51	2.49	2.53	2.54	2.52	0.80	2.51	2.49	2.51	2.50	2.49	0.26
55	DCER(24:1)+H	2.50	2.52	2.50	2.51	2.50	0.38	2.48	2.48	2.51	2.52	2.52	0.84	2.47	2.49	2.49	2.50	2.46	0.67
56	DCER(26:0)+H	2.53	2.61	2.62	2.56	2.62	1.61	2.58	2.57	2.61	2.72	2.65	2.39	2.67	2.64	2.55	2.65	2.57	2.02
57	HCER(14:0)+H	6.61	6.67	6.66	6.71	6.63	0.55	6.49	6.53	6.42	6.51	6.45	0.64	5.66	5.62	5.61	5.62	5.60	0.44
58	HCER(16:0)+H	6.47	6.49	6.51	6.50	6.50	0.22	6.37	6.30	6.31	6.30	6.30	0.52	6.03	6.00	5.97	5.95	5.92	0.73
59	HCER(18:0)+H	6.32	6.36	6.28	6.41	6.35	0.78	6.14	6.19	6.18	6.21	6.15	0.44	5.85	5.81	5.82	5.87	5.79	0.55
60	HCER(18:1)+H	6.65	6.73	6.67	6.76	6.74	0.69	6.61	6.61	6.64	6.57	6.63	0.36	6.11	6.05	6.06	6.02	6.05	0.50
61	HCER(20:0)+H	6.22	6.24	6.25	6.33	6.24	0.67	6.05	6.05	6.05	6.10	6.05	0.39	5.76	5.68	5.71	5.73	5.73	0.50
62	HCER(20:1)+H	6.30	6.53	6.38	6.38	6.39	1.31	6.36	6.22	6.36	6.29	6.22	1.12	5.98	5.92	5.90	5.89	5.89	0.64
63	HCER(22:0)+H	6.08	6.15	6.07	6.22	6.14	0.97	5.96	5.97	6.00	5.92	5.94	0.52	5.71	5.68	5.67	5.66	5.66	0.41
64	HCER(22:1)+H	6.17	6.21	6.18	6.25	6.19	0.52	6.04	6.02	5.99	6.05	5.96	0.59	5.84	5.78	5.77	5.76	5.77	0.55
65	HCER(24:0)+H	6.06	6.01	6.04	6.07	6.05	0.36	5.89	5.86	5.86	5.82	5.84	0.45	5.62	5.51	5.58	5.54	5.54	0.82
66	HCER(24:1)+H	6.04	6.11	6.10	6.19	6.07	0.90	5.92	5.88	5.94	5.88	5.93	0.45	5.73	5.67	5.66	5.66	5.66	0.53
67	HCER(26:0)+H	N/A	N/A	N/A	N/A	N/A	#####	N/A	N/A	N/A	N/A	N/A	#####	5.61	5.52	5.50	5.51	5.50	0.84
68	HCER(26:1)+H	6.01	6.01	6.02	6.07	6.02	0.40	5.98	5.90	6.06	5.95	5.87	1.30	5.64	5.56	5.54	5.55	5.54	0.79
69	HCER(d18:0/18:0)+H	6.36	6.35	6.41	6.39	6.41	0.44	6.13	6.25	6.06	6.14	6.12	1.13	5.85	5.79	5.81	5.80	5.80	0.38
70	HCER(d18:0/20:0)+H	6.22	6.30	6.14	6.24	6.17	1.00	6.08	6.00	6.00	6.07	5.98	0.74	5.82	5.78	5.73	5.76	5.75	0.62
71	HCER(d18:0/22:0)+H	6.07	6.13	6.11	6.12	6.16	0.55	5.98	5.90	5.95	5.96	5.93	0.54	5.66	5.60	5.66	5.66	5.63	0.51
72	HCER(d18:0/24:0)+H	5.99	6.02	6.00	6.07	6.02	0.48	5.89	5.89	5.77	5.87	5.88	0.85	5.57	5.61	5.60	5.63	5.53	0.71
73	HCER(d18:0/24:1)+H	6.07	6.05	6.10	6.12	6.09	0.47	5.90	5.87	5.87	5.95	5.92	0.60	5.74	5.66	5.66	5.66	5.66	0.60
74	HCER(d18:0/26:0)+H	N/A	N/A	N/A	N/A	N/A	#####	N/A	N/A	N/A	N/A	N/A	#####	5.50	5.45	5.48	5.46	5.49	0.37
75	HCER(d18:0/26:1)+H	N/A	N/A	N/A	N/A	N/A	#####	N/A	N/A	N/A	N/A	N/A	#####	5.62	5.57	5.55	5.57	5.54	0.52
76	LCER(14:0)+H	2.36	2.34	2.38	2.31	2.34	1.02	2.43	2.39	2.41	2.32	2.38	1.78	2.78	2.79	2.67	2.51	2.79	4.51
77	LCER(16:0)+H	2.40	2.33	2.39	2.31	2.36	1.60	2.53	2.41	2.37	2.42	2.41	2.43	2.38	2.49	2.37	2.40	2.46	2.10
78	LCER(18:0)+H	2.40	2.49	2.42	2.42	2.42	1.41	2.43	2.56	2.42	2.50	2.49	2.37	2.49	2.48	2.53	2.51	2.35	2.83
79	LCER(18:1)+H	2.42	2.45	2.48	2.40	2.45	1.26	2.51	2.51	2.45	2.46	2.51	1.21	2.43	2.46	2.51	2.43	2.46	1.31
80	LCER(20:0)+H	2.35	2.36	2.35	2.40	2.35	1.04	2.42	2.39	2.39	2.42	2.37	0.79	2.42	2.45	2.36	2.40	2.35	1.59
81	LCER(20:1)+H	2.43	2.42	2.43	2.43	2.47	0.72	2.52	2.48	2.53	2.56	2.56	1.20	2.49	2.50	2.49	2.48	2.44	0.89

82	LCER(22:0)+H	2.46	2.50	2.48	2.46	2.45	0.78	2.67	2.49	2.32	2.60	2.63	5.58	2.38	2.56	2.75	2.47	2.50	5.53
83	LCER(22:1)+H	2.61	2.49	2.45	2.44	2.53	2.71	2.73	2.54	2.52	2.47	2.72	4.61	2.45	2.44	2.53	2.41	2.49	1.90
84	LCER(24:0)+H	2.59	2.64	2.59	2.67	2.65	1.38	2.45	2.50	2.62	2.74	2.64	4.45	2.67	2.38	2.55	2.69	2.49	5.08
85	LCER(24:1)+H	2.53	2.44	2.52	2.47	2.47	1.64	2.49	2.51	2.51	2.57	2.49	1.33	2.53	2.37	2.46	2.37	2.46	2.73
86	LCER(26:0)+H	2.50	2.51	2.51	2.55	2.47	1.22	2.46	2.48	2.47	2.47	2.53	1.16	2.58	2.53	2.44	2.50	2.53	2.01
87	LCER(26:1)+H	2.47	2.52	2.54	2.48	2.47	1.27	2.55	2.47	2.46	2.49	2.53	1.48	2.43	2.50	2.49	2.47	2.45	1.06
88	LCER(d18:0/18:0)+H	2.38	2.40	2.41	2.42	2.49	1.76	2.37	2.43	2.50	2.51	2.39	2.66	2.37	2.43	2.43	2.51	2.41	2.03
89	LCER(d18:0/20:0)+H	2.37	2.31	2.54	2.36	2.41	3.54	2.41	2.29	2.37	2.49	2.45	3.24	2.44	2.43	2.32	2.32	2.48	3.17
90	LCER(d18:0/22:0)+H	2.32	2.25	2.45	2.37	2.67	6.70	2.23	2.68	2.50	2.42	2.52	6.62	2.49	2.45	2.32	2.62	2.45	4.34
91	LCER(d18:0/24:0)+H	2.47	2.45	2.20	2.29	2.90	10.99	2.64	2.44	2.60	2.58	2.47	3.35	2.61	2.50	2.38	2.57	2.54	3.50
92	LCER(d18:0/24:1)+H	2.34	2.68	2.38	2.37	2.65	6.74	2.29	2.56	2.53	2.65	2.44	5.55	2.48	2.32	2.46	2.55	2.53	3.69
93	LCER(d18:0/26:0)+H	2.47	2.77	2.33	2.55	2.41	6.71	2.51	2.47	2.64	2.61	2.46	3.33	2.51	2.54	2.61	2.64	2.48	2.67
94	LCER(d18:0/26:1)+H	2.30	2.77	2.40	2.04	2.42	11.01	2.46	2.38	2.46	2.46	2.47	1.47	2.40	2.84	2.42	2.45	2.54	7.15
95	TAG(40:0/FA14:0)+NH4	2.42	2.26	2.43	2.47	2.45	3.59	2.46	2.49	2.39	2.58	2.51	2.85	2.30	2.38	2.41	2.46	2.49	3.10
96	TAG(40:0/FA16:0)+NH4	2.29	2.43	2.45	2.46	2.47	2.98	2.44	2.44	2.42	2.44	2.45	0.42	2.42	2.40	2.42	2.44	2.46	0.89
97	TAG(42:0/FA14:0)+NH4	2.26	2.43	2.37	2.40	2.44	2.98	2.42	2.49	2.39	2.38	2.46	2.01	2.45	2.53	2.37	2.51	2.35	3.32
98	TAG(42:0/FA16:0)+NH4	2.37	2.40	2.42	2.43	2.42	0.85	2.41	2.41	2.40	2.43	2.38	0.78	2.39	2.41	2.46	2.44	2.42	1.05
99	TAG(42:1/FA14:0)+NH4	2.22	2.33	2.41	2.41	2.42	3.64	2.32	2.36	2.39	2.34	2.37	1.17	2.35	2.38	2.39	2.41	2.36	1.10
100	TAG(42:1/FA16:0)+NH4	2.41	2.34	2.34	2.43	2.40	1.75	2.42	2.37	2.37	2.40	2.37	1.03	2.39	2.34	2.38	2.37	2.39	0.91
101	TAG(42:1/FA18:1)+NH4	2.33	2.42	2.45	2.37	2.37	1.97	2.43	2.42	2.39	2.40	2.44	0.81	2.43	2.45	2.40	2.45	2.38	1.28
102	TAG(42:2/FA18:2)+NH4	2.33	2.27	2.38	2.35	2.43	2.45	2.43	2.35	2.40	2.43	2.47	1.75	2.39	2.49	2.46	2.47	2.37	2.14
103	TAG(44:0/FA14:0)+NH4	2.39	2.41	2.41	2.41	2.44	0.68	2.39	2.40	2.43	2.48	2.43	1.38	2.46	2.40	2.45	2.44	2.40	1.20
104	TAG(44:0/FA16:0)+NH4	2.40	2.45	2.40	2.46	2.42	1.09	2.47	2.46	2.45	2.47	2.45	0.50	2.45	2.46	2.46	2.46	2.43	0.49
105	TAG(44:0/FA18:0)+NH4	2.32	2.40	2.44	2.38	2.36	1.96	2.41	2.41	2.43	2.45	2.59	3.07	2.40	2.46	2.47	2.44	2.45	1.07
106	TAG(44:1/FA14:0)+NH4	2.35	2.34	2.36	2.33	2.36	0.59	2.36	2.37	2.41	2.51	2.44	2.60	2.45	2.36	2.43	2.47	2.36	2.16
107	TAG(44:1/FA16:0)+NH4	2.35	2.40	2.37	2.37	2.35	0.83	2.40	2.39	2.41	2.39	2.41	0.43	2.34	2.39	2.41	2.41	2.38	1.23
108	TAG(44:1/FA16:1)+NH4	2.34	2.40	2.32	2.41	2.43	1.88	2.40	2.44	2.42	2.43	2.38	1.10	2.34	2.36	2.44	2.45	2.33	2.38
109	TAG(44:1/FA18:1)+NH4	2.31	2.39	2.34	2.32	2.35	1.33	2.40	2.36	2.41	2.42	2.42	1.06	2.39	2.42	2.42	2.41	2.45	0.89
110	TAG(44:2/FA14:0)+NH4	2.37	2.34	2.28	2.39	2.41	2.19	2.39	2.43	2.35	2.50	2.46	2.48	2.32	2.41	2.39	2.41	2.46	1.99

111	TAG(44:2/FA16:0)+NH4	2.36	2.36	2.33	2.35	2.26	1.86	2.33	2.37	2.36	2.39	2.39	1.02	2.39	2.39	2.36	2.40	2.32	1.44
112	TAG(44:2/FA16:1)+NH4	2.41	2.38	2.32	2.40	2.32	1.88	2.36	2.41	2.46	2.53	2.38	2.83	2.36	2.37	2.64	2.80	2.38	8.00
113	TAG(44:2/FA18:1)+NH4	2.36	2.32	2.38	2.35	2.40	1.25	2.36	2.41	2.38	2.41	2.38	0.97	2.29	2.37	2.36	2.42	2.36	1.88
114	TAG(44:2/FA18:2)+NH4	2.28	2.33	2.35	2.32	2.32	1.07	2.38	2.38	2.35	2.41	2.37	0.91	2.34	2.40	2.41	2.39	2.36	1.25
115	TAG(44:3/FA18:2)+NH4	2.46	2.34	2.27	2.25	2.34	3.48	2.29	2.39	2.45	2.40	2.36	2.41	2.35	2.38	2.46	2.40	2.47	2.08
116	TAG(45:0/FA14:0)+NH4	2.41	2.40	2.45	2.46	2.41	1.16	2.48	2.44	2.47	2.42	2.50	1.26	2.43	2.48	2.47	2.42	2.45	0.98
117	TAG(45:0/FA16:0)+NH4	2.42	2.44	2.44	2.46	2.43	0.67	2.51	2.56	2.46	2.48	2.51	1.61	2.45	2.45	2.44	2.41	2.47	0.99
118	TAG(45:1/FA16:0)+NH4	2.33	2.37	2.37	2.39	2.38	0.98	2.41	2.44	2.47	2.39	2.43	1.25	2.38	2.45	2.35	2.40	2.39	1.55
119	TAG(45:1/FA18:1)+NH4	2.37	2.36	2.31	2.39	2.45	2.12	2.44	2.45	2.45	2.40	2.47	1.14	2.38	2.45	2.43	2.42	2.42	1.10
120	TAG(46:0/FA14:0)+NH4	2.41	2.45	2.44	2.44	2.44	0.70	2.46	2.50	2.46	2.46	2.49	0.76	2.44	2.44	2.48	2.45	2.47	0.73
121	TAG(46:0/FA16:0)+NH4	2.40	2.43	2.44	2.45	2.46	0.85	2.47	2.48	2.49	2.46	2.49	0.58	2.42	2.48	2.47	2.44	2.49	1.13
122	TAG(46:0/FA18:0)+NH4	2.39	2.40	2.42	2.40	2.41	0.56	2.45	2.44	2.45	2.46	2.43	0.52	2.51	2.47	2.47	2.48	2.49	0.61
123	TAG(46:1/FA14:0)+NH4	2.35	2.35	2.37	2.38	2.39	0.75	2.41	2.41	2.47	2.41	2.42	1.04	2.38	2.41	2.45	2.39	2.43	1.19
124	TAG(46:1/FA16:0)+NH4	2.34	2.37	2.38	2.39	2.38	0.87	2.37	2.39	2.41	2.41	2.40	0.54	2.36	2.40	2.42	2.40	2.41	1.00
125	TAG(46:1/FA16:1)+NH4	2.34	2.37	2.35	2.40	2.38	1.04	2.40	2.41	2.42	2.43	2.43	0.57	2.39	2.40	2.41	2.40	2.41	0.32
126	TAG(46:1/FA18:0)+NH4	2.33	2.40	2.35	2.39	2.38	1.22	2.38	2.38	2.44	2.40	2.40	0.98	2.34	2.36	2.37	2.41	2.39	1.09
127	TAG(46:1/FA18:1)+NH4	2.35	2.36	2.38	2.40	2.38	0.75	2.42	2.42	2.43	2.45	2.38	0.98	2.41	2.42	2.42	2.40	2.44	0.52
128	TAG(46:2/FA14:0)+NH4	2.32	2.32	2.37	2.35	2.39	1.26	2.32	2.40	2.41	2.45	2.45	2.14	2.36	2.37	2.39	2.40	2.40	0.76
129	TAG(46:2/FA16:0)+NH4	2.29	2.34	2.36	2.40	2.36	1.65	2.30	2.34	2.36	2.40	2.40	1.82	2.32	2.36	2.38	2.41	2.40	1.46
130	TAG(46:2/FA16:1)+NH4	2.32	2.34	2.35	2.36	2.37	0.94	2.34	2.37	2.38	2.39	2.39	0.85	2.37	2.39	2.38	2.44	2.40	1.13
131	TAG(46:2/FA18:1)+NH4	2.29	2.32	2.36	2.41	2.37	2.01	2.37	2.39	2.37	2.42	2.38	0.94	2.36	2.36	2.40	2.38	2.37	0.63
132	TAG(46:2/FA18:2)+NH4	2.33	2.35	2.35	2.38	2.37	0.89	2.34	2.35	2.38	2.41	2.40	1.25	2.32	2.35	2.36	2.36	2.35	0.69
133	TAG(46:3/FA14:0)+NH4	2.34	2.38	2.35	2.35	2.32	0.92	2.38	2.33	2.42	2.42	2.45	2.00	2.39	2.42	2.41	2.46	2.38	1.18
134	TAG(46:3/FA16:0)+NH4	2.31	2.38	2.30	2.30	2.35	1.58	2.32	2.43	2.37	2.39	2.38	1.70	2.37	2.35	2.34	2.34	2.36	0.61
135	TAG(46:3/FA16:1)+NH4	2.25	2.31	2.33	2.33	2.37	1.89	2.37	2.44	2.37	2.41	2.42	1.19	2.34	2.36	2.40	2.42	2.37	1.36
136	TAG(46:3/FA18:1)+NH4	2.29	2.35	2.33	2.31	2.31	0.94	2.37	2.38	2.37	2.36	2.39	0.46	2.33	2.38	2.34	2.40	2.38	1.26
137	TAG(46:3/FA18:2)+NH4	2.30	2.36	2.30	2.35	2.34	1.27	2.37	2.34	2.37	2.41	2.36	1.12	2.32	2.33	2.41	2.41	2.34	1.85
138	TAG(46:3/FA18:3)+NH4	2.27	2.39	2.26	2.31	2.38	2.66	2.32	2.31	2.35	2.34	2.49	3.17	2.31	2.33	2.41	2.38	2.37	1.61
139	TAG(46:4/FA18:2)+NH4	2.35	2.38	2.39	2.35	2.41	1.12	2.41	2.47	2.39	2.52	2.40	2.20	2.36	2.41	2.50	2.39	2.39	2.09

140	TAG(47:0/FA14:0)+NH4	2.39	2.42	2.40	2.34	2.43	1.52	2.43	2.38	2.43	2.45	2.45	1.23	2.44	2.37	2.44	2.45	2.48	1.61
141	TAG(47:0/FA16:0)+NH4	2.44	2.45	2.47	2.45	2.48	0.58	2.47	2.48	2.48	2.50	2.48	0.44	2.46	2.46	2.46	2.46	2.49	0.58
142	TAG(47:0/FA17:0)+NH4	2.34	2.37	2.36	2.39	2.38	0.92	2.42	2.44	2.43	2.42	2.39	0.82	2.38	2.41	2.43	2.46	2.43	1.23
143	TAG(47:1/FA14:0)+NH4	2.38	2.37	2.40	2.39	2.41	0.67	2.42	2.37	2.42	2.46	2.42	1.19	2.41	2.45	2.43	2.43	2.45	0.71
144	TAG(47:1/FA16:0)+NH4	2.35	2.35	2.37	2.41	2.40	1.10	2.37	2.40	2.37	2.39	2.38	0.59	2.40	2.41	2.41	2.42	2.38	0.72
145	TAG(47:1/FA16:1)+NH4	2.39	2.37	2.39	2.36	2.40	0.68	2.42	2.43	2.43	2.44	2.46	0.68	2.39	2.43	2.43	2.45	2.47	1.14
146	TAG(47:1/FA17:0)+NH4	2.30	2.35	2.38	2.31	2.36	1.39	2.40	2.33	2.40	2.47	2.36	2.22	2.35	2.38	2.37	2.40	2.34	1.02
147	TAG(47:1/FA18:1)+NH4	2.41	2.42	2.41	2.38	2.44	0.95	2.47	2.45	2.49	2.46	2.46	0.54	2.43	2.45	2.40	2.46	2.45	0.86
148	TAG(47:2/FA14:0)+NH4	2.38	2.35	2.37	2.37	2.39	0.69	2.38	2.33	2.42	2.38	2.49	2.43	2.34	2.38	2.45	2.34	2.42	1.96
149	TAG(47:2/FA16:1)+NH4	2.33	2.36	2.29	2.37	2.33	1.35	2.36	2.37	2.41	2.39	2.39	0.85	2.33	2.19	2.36	2.37	2.44	4.01
150	TAG(47:2/FA18:1)+NH4	2.33	2.30	2.37	2.40	2.34	1.66	2.37	2.39	2.42	2.42	2.39	0.92	2.37	2.35	2.37	2.38	2.36	0.49
151	TAG(47:2/FA18:2)+NH4	2.35	2.42	2.39	2.41	2.45	1.52	2.43	2.45	2.41	2.43	2.48	1.23	2.38	2.41	2.41	2.41	2.43	0.79
152	TAG(48:0/FA14:0)+NH4	2.40	2.46	2.44	2.45	2.47	1.01	2.49	2.50	2.49	2.51	2.44	1.03	2.49	2.49	2.46	2.45	2.46	0.71
153	TAG(48:0/FA16:0)+NH4	2.49	2.48	2.51	2.51	2.54	0.97	2.58	2.56	2.52	2.57	2.53	0.97	2.50	2.51	2.50	2.50	2.50	0.27
154	TAG(48:0/FA18:0)+NH4	2.47	2.49	2.49	2.47	2.49	0.47	2.56	2.52	2.50	2.50	2.51	0.99	2.49	2.45	2.50	2.50	2.49	0.81
155	TAG(48:1/FA14:0)+NH4	2.41	2.41	2.42	2.40	2.40	0.39	2.46	2.45	2.45	2.45	2.46	0.20	2.40	2.44	2.43	2.44	2.44	0.74
156	TAG(48:1/FA16:0)+NH4	2.40	2.37	2.42	2.42	2.41	0.88	2.46	2.45	2.46	2.48	2.44	0.65	2.41	2.43	2.44	2.44	2.43	0.53
157	TAG(48:1/FA16:1)+NH4	2.40	2.40	2.40	2.43	2.41	0.54	2.45	2.46	2.49	2.45	2.43	0.81	2.41	2.43	2.43	2.46	2.43	0.63
158	TAG(48:1/FA18:0)+NH4	2.38	2.37	2.41	2.42	2.41	0.82	2.37	2.40	2.48	2.46	2.44	1.83	2.41	2.41	2.40	2.43	2.42	0.39
159	TAG(48:1/FA18:1)+NH4	2.39	2.39	2.40	2.40	2.43	0.63	2.43	2.45	2.45	2.46	2.47	0.62	2.42	2.44	2.47	2.42	2.44	0.78
160	TAG(48:2/FA14:0)+NH4	2.34	2.34	2.35	2.37	2.39	0.81	2.36	2.38	2.41	2.39	2.39	0.69	2.37	2.41	2.42	2.38	2.39	0.86
161	TAG(48:2/FA16:0)+NH4	2.35	2.34	2.37	2.37	2.37	0.64	2.40	2.39	2.41	2.38	2.39	0.55	2.39	2.37	2.43	2.42	2.39	1.02
162	TAG(48:2/FA16:1)+NH4	2.34	2.34	2.33	2.34	2.38	0.82	2.38	2.38	2.41	2.41	2.41	0.74	2.38	2.36	2.41	2.39	2.39	0.66
163	TAG(48:2/FA18:0)+NH4	2.33	2.37	2.40	2.34	2.36	1.21	2.33	2.36	2.39	2.47	2.42	2.28	2.40	2.41	2.41	2.35	2.34	1.43
164	TAG(48:2/FA18:1)+NH4	2.34	2.35	2.37	2.38	2.36	0.76	2.40	2.40	2.40	2.36	2.38	0.74	2.37	2.41	2.40	2.40	2.38	0.64
165	TAG(48:2/FA18:2)+NH4	2.34	2.34	2.37	2.39	2.36	0.93	2.35	2.40	2.40	2.44	2.40	1.20	2.36	2.39	2.40	2.43	2.42	1.14
166	TAG(48:3/FA14:0)+NH4	2.31	2.33	2.33	2.32	2.32	0.38	2.37	2.31	2.35	2.33	2.37	1.15	2.30	2.34	2.37	2.40	2.34	1.45
167	TAG(48:3/FA16:0)+NH4	2.31	2.34	2.32	2.35	2.35	0.79	2.33	2.35	2.35	2.37	2.37	0.69	2.34	2.37	2.36	2.35	2.36	0.44
168	TAG(48:3/FA16:1)+NH4	2.33	2.32	2.34	2.36	2.34	0.57	2.35	2.37	2.38	2.39	2.38	0.58	2.33	2.36	2.39	2.36	2.37	0.85

169	TAG(48:3/FA18:1)+NH4	2.32	2.32	2.33	2.35	2.37	0.92	2.33	2.33	2.36	2.36	2.36	0.71	2.35	2.35	2.36	2.34	2.37	0.58
170	TAG(48:3/FA18:2)+NH4	2.33	2.33	2.34	2.28	2.36	1.22	2.33	2.35	2.39	2.41	2.36	1.40	2.33	2.36	2.35	2.35	2.38	0.73
171	TAG(48:3/FA18:3)+NH4	2.31	2.35	2.31	2.34	2.35	0.85	2.34	2.32	2.37	2.37	2.36	0.94	2.37	2.36	2.44	2.38	2.35	1.47
172	TAG(48:4/FA14:0)+NH4	2.33	2.33	2.28	2.33	2.34	1.11	2.35	2.23	2.30	2.23	2.30	2.27	2.36	2.35	2.38	2.38	2.38	0.63
173	TAG(48:4/FA16:0)+NH4	2.27	2.30	2.27	2.38	2.42	2.90	2.30	2.33	2.45	2.36	2.35	2.41	2.38	2.35	2.32	2.33	2.40	1.54
174	TAG(48:4/FA16:1)+NH4	2.27	2.33	2.32	2.25	2.32	1.53	2.38	2.34	2.39	2.32	2.36	1.24	2.29	2.34	2.44	2.36	2.37	2.29
175	TAG(48:4/FA18:1)+NH4	2.30	2.34	2.32	2.32	2.34	0.81	2.36	2.35	2.37	2.34	2.38	0.64	2.33	2.31	2.39	2.38	2.31	1.77
176	TAG(48:4/FA18:2)+NH4	2.34	2.36	2.37	2.29	2.32	1.27	2.32	2.29	2.37	2.36	2.40	1.82	2.26	2.36	2.31	2.36	2.27	2.10
177	TAG(48:4/FA18:3)+NH4	2.27	2.32	2.32	2.32	2.32	0.88	2.33	2.39	2.36	2.34	2.34	1.08	2.32	2.32	2.39	2.40	2.35	1.63
178	TAG(48:4/FA20:4)+NH4	2.32	2.38	2.31	2.31	2.31	1.32	2.36	2.42	2.38	2.37	2.35	1.08	2.34	2.37	2.33	2.35	2.39	1.11
179	TAG(48:5/FA18:2)+NH4	2.32	2.26	2.29	2.30	2.21	1.87	2.35	2.44	2.47	2.44	2.53	2.65	2.31	2.37	2.46	2.32	2.38	2.52
180	TAG(48:5/FA18:3)+NH4	2.32	2.34	2.24	2.26	2.41	2.90	2.24	2.43	2.42	2.31	2.33	3.34	2.38	2.37	2.40	2.33	2.29	1.93
181	TAG(49:0/FA16:0)+NH4	2.38	2.42	2.42	2.43	2.45	0.94	2.47	2.47	2.47	2.48	2.47	0.17	2.40	2.44	2.48	2.47	2.46	1.23
182	TAG(49:0/FA17:0)+NH4	2.36	2.41	2.42	2.40	2.40	1.01	2.43	2.44	2.42	2.49	2.45	1.06	2.41	2.46	2.42	2.42	2.48	1.29
183	TAG(49:0/FA18:0)+NH4	2.48	2.50	2.49	2.49	2.46	0.53	2.51	2.52	2.48	2.54	2.49	0.92	2.49	2.49	2.47	2.49	2.50	0.44
184	TAG(49:1/FA14:0)+NH4	2.35	2.34	2.36	2.34	2.41	1.22	2.39	2.39	2.39	2.36	2.37	0.66	2.41	2.41	2.40	2.36	2.41	0.88
185	TAG(49:1/FA16:0)+NH4	2.41	2.41	2.41	2.44	2.43	0.54	2.45	2.47	2.44	2.45	2.46	0.49	2.43	2.44	2.46	2.46	2.46	0.60
186	TAG(49:1/FA16:1)+NH4	2.36	2.37	2.39	2.39	2.43	1.16	2.37	2.44	2.41	2.47	2.43	1.56	2.39	2.41	2.42	2.42	2.40	0.57
187	TAG(49:1/FA17:0)+NH4	2.34	2.36	2.38	2.37	2.39	0.78	2.35	2.41	2.42	2.37	2.41	1.18	2.39	2.39	2.39	2.39	2.40	0.16
188	TAG(49:1/FA18:1)+NH4	2.42	2.45	2.45	2.44	2.45	0.55	2.48	2.48	2.45	2.46	2.46	0.50	2.43	2.47	2.46	2.45	2.48	0.81
189	TAG(49:2/FA14:0)+NH4	2.33	2.38	2.34	2.34	2.38	0.96	2.31	2.33	2.38	2.36	2.33	1.12	2.35	2.33	2.40	2.35	2.40	1.42
190	TAG(49:2/FA16:0)+NH4	2.34	2.37	2.35	2.35	2.40	0.92	2.38	2.38	2.40	2.43	2.41	0.87	2.37	2.40	2.40	2.40	2.41	0.64
191	TAG(49:2/FA16:1)+NH4	2.36	2.35	2.37	2.34	2.37	0.61	2.44	2.37	2.38	2.44	2.40	1.37	2.39	2.35	2.42	2.42	2.36	1.37
192	TAG(49:2/FA17:0)+NH4	2.32	2.36	2.36	2.40	2.32	1.52	2.42	2.36	2.33	2.36	2.32	1.73	2.32	2.36	2.35	2.39	2.34	1.06
193	TAG(49:2/FA18:1)+NH4	2.32	2.36	2.34	2.40	2.38	1.37	2.36	2.38	2.36	2.39	2.37	0.58	2.36	2.38	2.38	2.39	2.38	0.55
194	TAG(49:2/FA18:2)+NH4	2.36	2.36	2.36	2.40	2.39	0.84	2.42	2.36	2.38	2.42	2.37	1.13	2.37	2.39	2.42	2.42	2.38	0.82
195	TAG(49:3/FA16:0)+NH4	2.30	2.35	2.33	2.35	2.38	1.18	2.29	2.38	2.36	2.38	2.35	1.56	2.33	2.36	2.35	2.33	2.33	0.64
196	TAG(49:3/FA16:1)+NH4	2.32	2.35	2.34	2.33	2.37	0.86	2.37	2.37	2.36	2.41	2.42	1.19	2.37	2.40	2.40	2.34	2.38	1.00
197	TAG(49:3/FA18:2)+NH4	2.33	2.33	2.30	2.33	2.34	0.64	2.36	2.32	2.38	2.38	2.37	0.95	2.32	2.29	2.35	2.38	2.33	1.46

198	TAG(49:3/FA18:3)+NH4	2.29	2.31	2.31	2.34	2.33	0.83	2.36	2.32	2.37	2.42	2.33	1.67	2.31	2.39	2.39	2.40	2.41	1.72
199	TAG(50:0/FA14:0)+NH4	2.46	2.42	2.45	2.49	2.52	1.44	2.64	2.55	2.54	2.50	2.45	2.79	2.46	2.50	2.50	2.48	2.57	1.66
200	TAG(50:0/FA16:0)+NH4	2.50	2.48	2.56	2.54	2.51	1.24	2.72	2.69	2.59	2.65	2.59	2.24	2.53	2.54	2.53	2.52	2.50	0.64
201	TAG(50:0/FA18:0)+NH4	2.54	2.54	2.60	2.61	2.58	1.25	2.78	2.78	2.72	2.74	2.70	1.39	2.66	2.59	2.65	2.60	2.62	1.16
202	TAG(50:1/FA14:0)+NH4	2.42	2.41	2.45	2.46	2.45	0.87	2.50	2.49	2.45	2.47	2.46	0.85	2.44	2.43	2.46	2.46	2.47	0.67
203	TAG(50:1/FA16:0)+NH4	2.44	2.44	2.47	2.48	2.47	0.71	2.54	2.50	2.49	2.50	2.51	0.71	2.46	2.51	2.48	2.48	2.50	0.79
204	TAG(50:1/FA16:1)+NH4	2.41	2.42	2.40	2.45	2.45	0.88	2.51	2.48	2.46	2.48	2.47	0.64	2.46	2.46	2.47	2.40	2.47	1.21
205	TAG(50:1/FA18:0)+NH4	2.42	2.42	2.45	2.46	2.44	0.74	2.54	2.50	2.49	2.51	2.50	0.84	2.55	2.48	2.58	2.56	2.45	2.17
206	TAG(50:1/FA18:1)+NH4	2.46	2.48	2.47	2.48	2.46	0.33	2.51	2.53	2.53	2.52	2.51	0.43	2.52	2.50	2.50	2.48	2.46	0.86
207	TAG(50:1/FA20:1)+NH4	2.37	2.42	2.38	2.41	2.44	1.03	2.38	2.40	2.36	2.36	2.40	0.83	2.42	2.46	2.42	2.44	2.44	0.65
208	TAG(50:2/FA14:0)+NH4	2.35	2.36	2.40	2.40	2.41	1.18	2.41	2.40	2.40	2.42	2.43	0.55	2.40	2.46	2.51	2.50	2.43	1.90
209	TAG(50:2/FA16:0)+NH4	2.37	2.37	2.39	2.42	2.40	0.94	2.44	2.43	2.41	2.46	2.45	0.79	2.42	2.41	2.44	2.44	2.44	0.69
210	TAG(50:2/FA16:1)+NH4	2.38	2.37	2.40	2.42	2.41	0.80	2.42	2.43	2.38	2.43	2.42	0.76	2.41	2.40	2.43	2.41	2.41	0.46
211	TAG(50:2/FA18:0)+NH4	2.38	2.36	2.39	2.38	2.36	0.51	2.40	2.38	2.39	2.41	2.42	0.70	2.42	2.42	2.41	2.37	2.41	0.83
212	TAG(50:2/FA18:1)+NH4	2.36	2.41	2.40	2.39	2.43	1.01	2.43	2.42	2.41	2.43	2.43	0.40	2.40	2.43	2.41	2.43	2.41	0.59
213	TAG(50:2/FA18:2)+NH4	2.35	2.38	2.37	2.42	2.42	1.22	2.45	2.48	2.44	2.45	2.43	0.69	2.41	2.42	2.43	2.42	2.40	0.57
214	TAG(50:2/FA20:2)+NH4	2.36	2.39	2.40	2.45	2.41	1.26	2.46	2.40	2.37	2.37	2.45	1.71	2.43	2.42	2.36	2.36	2.34	1.67
215	TAG(50:3/FA14:0)+NH4	2.31	2.33	2.35	2.37	2.36	1.10	2.37	2.38	2.37	2.36	2.39	0.45	2.35	2.39	2.38	2.35	2.38	0.68
216	TAG(50:3/FA16:0)+NH4	2.31	2.34	2.32	2.35	2.35	0.76	2.37	2.36	2.37	2.38	2.37	0.34	2.35	2.39	2.39	2.39	2.38	0.76
217	TAG(50:3/FA16:1)+NH4	2.32	2.35	2.32	2.34	2.34	0.60	2.37	2.35	2.39	2.37	2.38	0.57	2.36	2.37	2.37	2.39	2.38	0.49
218	TAG(50:3/FA18:0)+NH4	2.29	2.37	2.31	2.34	2.37	1.58	2.40	2.34	2.35	2.33	2.40	1.35	2.40	2.39	2.40	2.38	2.38	0.36
219	TAG(50:3/FA18:1)+NH4	2.31	2.34	2.33	2.37	2.36	0.88	2.35	2.36	2.37	2.38	2.39	0.67	2.36	2.38	2.38	2.36	2.37	0.48
220	TAG(50:3/FA18:2)+NH4	2.30	2.36	2.33	2.33	2.34	0.88	2.36	2.35	2.37	2.40	2.37	0.73	2.35	2.36	2.39	2.38	2.41	0.98
221	TAG(50:3/FA18:3)+NH4	2.32	2.36	2.35	2.37	2.36	0.72	2.37	2.36	2.35	2.40	2.40	0.89	2.34	2.37	2.39	2.37	2.36	0.79
222	TAG(50:3/FA20:3)+NH4	2.33	2.35	2.33	2.33	2.35	0.58	2.40	2.41	2.39	2.43	2.38	0.87	2.39	2.38	2.36	2.41	2.45	1.48
223	TAG(50:4/FA14:0)+NH4	2.31	2.32	2.32	2.33	2.33	0.44	2.34	2.34	2.36	2.34	2.40	1.07	2.33	2.35	2.38	2.36	2.36	0.79
224	TAG(50:4/FA16:0)+NH4	2.29	2.32	2.33	2.34	2.36	1.10	2.36	2.36	2.36	2.40	2.37	0.70	2.36	2.39	2.42	2.33	2.39	1.40
225	TAG(50:4/FA16:1)+NH4	2.32	2.33	2.30	2.33	2.35	0.74	2.37	2.35	2.36	2.37	2.39	0.63	2.31	2.35	2.36	2.40	2.36	1.29
226	TAG(50:4/FA18:1)+NH4	2.30	2.30	2.32	2.34	2.35	1.02	2.32	2.37	2.32	2.37	2.47	2.56	2.33	2.40	2.29	2.37	2.36	1.71

227	TAG(50:4/FA18:2)+NH4	2.31	2.31	2.31	2.33	2.33	0.50	2.33	2.37	2.35	2.35	2.35	0.63	2.32	2.37	2.37	2.34	2.37	0.89
228	TAG(50:4/FA18:3)+NH4	2.28	2.31	2.31	2.33	2.31	0.80	2.30	2.35	2.32	2.36	2.31	1.05	2.36	2.35	2.37	2.31	2.37	1.00
229	TAG(50:4/FA20:3)+NH4	2.29	2.35	2.26	2.31	2.31	1.49	2.37	2.42	2.42	2.55	2.45	2.72	2.36	2.42	2.39	2.40	2.46	1.46
230	TAG(50:4/FA20:4)+NH4	2.29	2.32	2.34	2.29	2.34	1.11	2.34	2.37	2.37	2.38	2.33	0.99	2.34	2.34	2.39	2.38	2.39	1.04
231	TAG(50:5/FA14:0)+NH4	2.27	2.28	2.31	2.35	2.29	1.35	2.41	2.37	2.36	2.48	2.39	2.04	2.30	2.37	2.34	2.21	2.33	2.63
232	TAG(50:5/FA16:0)+NH4	2.32	2.28	2.29	2.34	2.33	1.11	2.34	2.35	2.42	2.49	2.38	2.52	2.29	2.35	2.38	2.34	2.39	1.73
233	TAG(50:5/FA16:1)+NH4	2.31	2.31	2.26	2.33	2.32	1.18	2.31	2.37	2.38	2.37	2.38	1.25	2.33	2.27	2.41	2.31	2.37	2.44
234	TAG(50:5/FA18:1)+NH4	2.24	2.32	2.32	2.36	2.32	1.98	2.46	2.28	2.33	2.40	2.42	3.11	2.40	2.33	2.36	2.36	2.34	1.17
235	TAG(50:5/FA18:2)+NH4	2.31	2.29	2.30	2.32	2.30	0.45	2.31	2.34	2.29	2.34	2.35	0.94	2.30	2.34	2.35	2.35	2.30	1.09
236	TAG(50:5/FA18:3)+NH4	2.28	2.31	2.28	2.31	2.29	0.60	2.35	2.34	2.32	2.29	2.37	1.35	2.30	2.35	2.32	2.37	2.33	1.31
237	TAG(50:5/FA20:4)+NH4	2.32	2.32	2.32	2.30	2.30	0.54	2.32	2.34	2.35	2.29	2.35	0.98	2.27	2.35	2.29	2.41	2.37	2.50
238	TAG(50:5/FA20:5)+NH4	2.28	2.27	2.29	2.31	2.30	0.65	2.37	2.40	2.37	2.37	2.32	1.19	2.37	2.41	2.32	2.34	2.39	1.64
239	TAG(50:6/FA20:4)+NH4	2.31	2.34	2.34	2.31	2.36	0.92	2.27	2.30	2.37	2.43	2.45	3.34	2.33	2.35	2.36	2.45	2.40	2.07
240	TAG(51:0/FA16:0)+NH4	2.28	2.38	2.29	2.35	2.31	1.77	2.32	2.36	2.33	2.36	2.35	0.74	2.35	2.43	2.42	2.39	2.34	1.64
241	TAG(51:0/FA17:0)+NH4	2.37	2.44	2.43	2.45	2.41	1.34	2.44	2.53	2.44	2.52	2.48	1.67	2.52	2.57	2.50	2.49	2.52	1.22
242	TAG(51:0/FA18:0)+NH4	2.44	2.45	2.46	2.48	2.44	0.63	2.47	2.53	2.50	2.50	2.49	0.85	2.50	2.49	2.52	2.46	2.53	0.96
243	TAG(51:1/FA16:0)+NH4	2.36	2.40	2.33	2.39	2.36	1.13	2.36	2.41	2.41	2.42	2.42	1.08	2.35	2.42	2.42	2.41	2.42	1.26
244	TAG(51:1/FA17:0)+NH4	2.39	2.41	2.40	2.41	2.38	0.56	2.45	2.46	2.44	2.47	2.43	0.61	2.44	2.46	2.46	2.42	2.45	0.71
245	TAG(51:1/FA18:0)+NH4	2.44	2.44	2.44	2.45	2.48	0.69	2.51	2.49	2.48	2.49	2.47	0.56	2.49	2.50	2.49	2.47	2.50	0.51
246	TAG(51:1/FA18:1)+NH4	2.41	2.43	2.40	2.45	2.41	0.79	2.46	2.48	2.45	2.45	2.45	0.43	2.45	2.48	2.44	2.39	2.48	1.52
247	TAG(51:2/FA16:0)+NH4	2.37	2.38	2.35	2.40	2.38	0.84	2.38	2.37	2.42	2.36	2.40	0.99	2.40	2.41	2.41	2.38	2.41	0.48
248	TAG(51:2/FA16:1)+NH4	2.32	2.38	2.35	2.34	2.39	1.19	2.33	2.35	2.38	2.37	2.37	0.77	2.34	2.36	2.37	2.37	2.39	0.69
249	TAG(51:2/FA17:0)+NH4	2.34	2.33	2.35	2.36	2.38	0.73	2.38	2.40	2.41	2.42	2.41	0.62	2.41	2.41	2.44	2.41	2.41	0.61
250	TAG(51:2/FA18:1)+NH4	2.38	2.39	2.39	2.40	2.41	0.54	2.45	2.46	2.43	2.45	2.44	0.36	2.40	2.43	2.44	2.44	2.52	1.83
251	TAG(51:2/FA18:2)+NH4	2.35	2.35	2.37	2.38	2.37	0.53	2.40	2.44	2.44	2.42	2.41	0.79	2.40	2.43	2.41	2.40	2.45	0.97
252	TAG(51:3/FA16:1)+NH4	2.30	2.31	2.35	2.36	2.36	1.27	2.31	2.34	2.37	2.38	2.34	1.17	2.33	2.38	2.33	2.34	2.38	1.14
253	TAG(51:3/FA17:0)+NH4	2.33	2.31	2.32	2.32	2.29	0.60	2.36	2.33	2.42	2.36	2.34	1.39	2.38	2.37	2.39	2.35	2.37	0.54
254	TAG(51:3/FA18:2)+NH4	2.33	2.34	2.35	2.33	2.36	0.60	2.37	2.38	2.38	2.37	2.40	0.42	2.35	2.37	2.40	2.37	2.40	0.96
255	TAG(51:3/FA18:3)+NH4	2.33	2.32	2.31	2.32	2.39	1.43	2.33	2.36	2.36	2.35	2.34	0.50	2.30	2.45	2.32	2.34	2.38	2.51

256	TAG(51:4/FA16:1)+NH4	2.30	2.31	2.31	2.32	2.34	0.55	2.30	2.36	2.39	2.29	2.29	1.95	2.30	2.36	2.35	2.31	2.38	1.44
257	TAG(51:4/FA18:2)+NH4	2.31	2.32	2.31	2.30	2.33	0.40	2.38	2.37	2.36	2.32	2.33	1.15	2.31	2.31	2.34	2.34	2.35	0.78
258	TAG(51:4/FA18:3)+NH4	2.31	2.29	2.30	2.32	2.36	1.17	2.32	2.31	2.39	2.38	2.36	1.54	2.29	2.33	2.36	2.34	2.34	1.08
259	TAG(51:4/FA20:4)+NH4	2.30	2.33	2.31	2.33	2.35	0.73	2.36	2.38	2.41	2.43	2.45	1.52	2.35	2.30	2.39	2.38	2.33	1.55
260	TAG(51:5/FA18:2)+NH4	2.30	2.31	2.25	2.27	2.30	1.13	2.36	2.31	2.37	2.33	2.46	2.35	2.36	2.39	2.48	2.41	2.27	3.22
261	TAG(51:5/FA18:3)+NH4	2.30	2.32	2.28	2.34	2.29	1.02	2.27	2.25	2.32	2.26	2.30	1.27	2.33	2.30	2.35	2.31	2.36	1.15
262	TAG(52:0/FA16:0)+NH4	2.56	2.57	2.56	2.58	2.54	0.65	2.65	2.77	2.60	2.58	2.56	3.28	2.76	2.61	2.57	2.57	2.56	3.23
263	TAG(52:0/FA18:0)+NH4	2.56	2.56	2.75	2.58	2.59	3.08	2.69	2.62	2.63	2.57	2.61	1.53	2.43	2.62	2.58	2.57	2.57	2.79
264	TAG(52:0/FA20:0)+NH4	2.32	2.30	2.33	2.34	2.33	0.56	2.27	2.28	2.29	2.33	2.36	1.66	2.30	2.33	2.31	2.30	2.32	0.64
265	TAG(52:1/FA16:0)+NH4	2.50	2.50	2.49	2.50	2.51	0.24	2.63	2.58	2.55	2.55	2.54	1.48	2.54	2.57	2.55	2.51	2.54	0.92
266	TAG(52:1/FA16:1)+NH4	2.45	2.46	2.48	2.45	2.51	0.95	2.53	2.56	2.50	2.52	2.50	0.99	2.48	2.50	2.52	2.48	2.49	0.75
267	TAG(52:1/FA18:0)+NH4	2.53	2.52	2.58	2.56	2.52	1.09	2.71	2.68	2.59	2.66	2.64	1.67	2.57	2.55	2.51	2.60	2.57	1.36
268	TAG(52:1/FA18:1)+NH4	2.45	2.48	2.48	2.51	2.46	0.98	2.60	2.60	2.51	2.56	2.54	1.48	2.51	2.50	2.51	2.51	2.52	0.23
269	TAG(52:1/FA20:0)+NH4	2.30	2.33	2.32	2.33	2.34	0.71	2.32	2.29	2.29	2.33	2.35	1.11	2.27	2.32	2.30	2.28	2.31	0.98
270	TAG(52:1/FA20:1)+NH4	2.44	2.44	2.49	2.47	2.43	1.02	2.39	2.37	2.42	2.45	2.45	1.38	2.45	2.49	2.42	2.48	2.46	1.10
271	TAG(52:2/FA14:0)+NH4	2.35	2.38	2.37	2.36	2.36	0.51	2.32	2.36	2.35	2.34	2.34	0.65	2.35	2.36	2.38	2.36	2.35	0.56
272	TAG(52:2/FA16:0)+NH4	2.40	2.44	2.44	2.45	2.43	0.78	2.48	2.49	2.48	2.49	2.49	0.23	2.46	2.47	2.48	2.47	2.46	0.40
273	TAG(52:2/FA16:1)+NH4	2.37	2.39	2.44	2.42	2.40	1.11	2.46	2.43	2.46	2.44	2.46	0.63	2.52	2.44	2.46	2.46	2.43	1.44
274	TAG(52:2/FA18:0)+NH4	2.41	2.43	2.44	2.43	2.44	0.44	2.50	2.47	2.48	2.49	2.48	0.46	2.44	2.50	2.48	2.44	2.44	1.22
275	TAG(52:2/FA18:1)+NH4	2.42	2.43	2.45	2.46	2.43	0.60	2.49	2.48	2.49	2.46	2.48	0.50	2.44	2.48	2.47	2.47	2.46	0.67
276	TAG(52:2/FA18:2)+NH4	2.39	2.40	2.41	2.43	2.40	0.62	2.46	2.46	2.46	2.45	2.47	0.34	2.43	2.45	2.46	2.42	2.45	0.71
277	TAG(52:2/FA20:0)+NH4	2.28	2.29	2.35	2.40	2.36	2.22	2.31	2.28	2.33	2.33	2.29	0.96	2.31	2.33	2.30	2.31	2.33	0.62
278	TAG(52:2/FA20:1)+NH4	2.34	2.38	2.37	2.39	2.40	0.90	2.32	2.30	2.32	2.36	2.34	0.87	2.35	2.37	2.39	2.35	2.38	0.76
279	TAG(52:2/FA20:2)+NH4	2.41	2.46	2.44	2.46	2.40	1.16	2.48	2.48	2.47	2.48	2.45	0.45	2.44	2.46	2.47	2.47	2.47	0.51
280	TAG(52:3/FA14:0)+NH4	2.33	2.36	2.31	2.38	2.34	1.09	2.34	2.33	2.37	2.31	2.35	0.86	2.37	2.37	2.40	2.37	2.38	0.56
281	TAG(52:3/FA16:0)+NH4	2.34	2.38	2.36	2.39	2.39	0.89	2.43	2.42	2.42	2.40	2.40	0.50	2.36	2.39	2.43	2.39	2.41	1.08
282	TAG(52:3/FA16:1)+NH4	2.33	2.38	2.36	2.37	2.38	0.81	2.40	2.40	2.40	2.38	2.41	0.47	2.37	2.41	2.39	2.39	2.39	0.59
283	TAG(52:3/FA18:0)+NH4	2.34	2.37	2.35	2.37	2.40	1.05	2.40	2.38	2.42	2.42	2.44	0.95	2.38	2.41	2.42	2.39	2.42	0.74
284	TAG(52:3/FA18:1)+NH4	2.35	2.39	2.36	2.38	2.36	0.69	2.39	2.40	2.40	2.42	2.41	0.49	2.40	2.42	2.40	2.40	2.40	0.33

285	TAG(52:3/FA18:2)+NH4	2.35	2.37	2.38	2.38	2.38	0.58	2.43	2.40	2.41	2.42	2.42	0.50	2.39	2.40	2.43	2.40	2.41	0.65
286	TAG(52:3/FA18:3)+NH4	2.32	2.35	2.36	2.36	2.35	0.83	2.39	2.35	2.39	2.36	2.33	1.02	2.35	2.37	2.40	2.35	2.38	0.92
287	TAG(52:3/FA20:0)+NH4	2.32	2.37	2.34	2.33	2.38	1.03	2.39	2.32	2.42	2.39	2.37	1.45	2.38	2.40	2.34	2.39	2.38	0.92
288	TAG(52:3/FA20:1)+NH4	2.32	2.32	2.31	2.36	2.33	0.79	2.32	2.30	2.33	2.35	2.35	0.91	2.27	2.32	2.33	2.36	2.36	1.48
289	TAG(52:3/FA20:2)+NH4	2.35	2.35	2.36	2.35	2.35	0.19	2.36	2.41	2.38	2.38	2.38	0.66	2.33	2.38	2.40	2.41	2.41	1.34
290	TAG(52:3/FA20:3)+NH4	2.36	2.39	2.38	2.40	2.38	0.66	2.42	2.44	2.44	2.46	2.42	0.71	2.40	2.38	2.41	2.42	2.41	0.63
291	TAG(52:4/FA14:0)+NH4	2.33	2.35	2.35	2.35	2.32	0.60	2.34	2.31	2.35	2.33	2.40	1.45	2.33	2.34	2.38	2.37	2.34	0.93
292	TAG(52:4/FA16:0)+NH4	2.30	2.31	2.32	2.32	2.34	0.69	2.35	2.34	2.35	2.38	2.34	0.64	2.34	2.36	2.35	2.34	2.39	0.87
293	TAG(52:4/FA16:1)+NH4	2.29	2.30	2.32	2.33	2.35	1.06	2.31	2.35	2.37	2.35	2.33	0.97	2.34	2.36	2.37	2.34	2.36	0.58
294	TAG(52:4/FA18:0)+NH4	2.29	2.29	2.33	2.31	2.31	0.81	2.35	2.35	2.39	2.26	2.32	2.09	2.31	2.41	2.30	2.29	2.34	2.09
295	TAG(52:4/FA18:1)+NH4	2.28	2.31	2.32	2.33	2.34	0.99	2.33	2.37	2.35	2.35	2.34	0.59	2.34	2.36	2.39	2.33	2.34	1.07
296	TAG(52:4/FA18:2)+NH4	2.31	2.31	2.30	2.34	2.34	0.82	2.34	2.36	2.33	2.36	2.36	0.57	2.33	2.36	2.36	2.35	2.37	0.68
297	TAG(52:4/FA18:3)+NH4	2.30	2.32	2.30	2.34	2.35	0.95	2.33	2.35	2.34	2.34	2.36	0.42	2.34	2.36	2.36	2.37	2.35	0.53
298	TAG(52:4/FA20:0)+NH4	2.30	2.30	2.32	2.30	2.30	0.34	2.35	2.32	2.31	2.30	2.31	0.83	2.37	2.29	2.40	2.29	2.30	2.14
299	TAG(52:4/FA20:2)+NH4	2.33	2.28	2.30	2.35	2.32	1.18	2.38	2.34	2.36	2.29	2.38	1.56	2.33	2.33	2.37	2.31	2.36	1.12
300	TAG(52:4/FA20:3)+NH4	2.28	2.32	2.29	2.33	2.32	0.91	2.37	2.36	2.36	2.34	2.37	0.59	2.32	2.35	2.39	2.37	2.36	1.09
301	TAG(52:4/FA20:4)+NH4	2.31	2.30	2.33	2.34	2.31	0.68	2.37	2.37	2.37	2.37	2.39	0.39	2.35	2.35	2.39	2.35	2.38	0.75
302	TAG(52:4/FA22:4)+NH4	2.28	2.30	2.33	2.33	2.35	1.16	2.38	2.34	2.38	2.40	2.40	1.07	2.37	2.40	2.35	2.41	2.40	1.11
303	TAG(52:5/FA14:0)+NH4	2.29	2.31	2.30	2.31	2.33	0.77	2.36	2.34	2.32	2.38	2.37	1.02	2.24	2.30	2.34	2.31	2.34	1.81
304	TAG(52:5/FA16:0)+NH4	2.27	2.28	2.30	2.32	2.32	0.99	2.32	2.32	2.30	2.30	2.32	0.38	2.31	2.33	2.33	2.33	2.33	0.42
305	TAG(52:5/FA16:1)+NH4	2.29	2.26	2.28	2.30	2.34	1.22	2.33	2.34	2.33	2.28	2.30	1.10	2.32	2.32	2.35	2.30	2.34	0.88
306	TAG(52:5/FA18:1)+NH4	2.31	2.28	2.30	2.30	2.32	0.58	2.32	2.34	2.30	2.28	2.34	1.07	2.34	2.34	2.32	2.31	2.32	0.57
307	TAG(52:5/FA18:2)+NH4	2.26	2.28	2.30	2.29	2.30	0.65	2.32	2.33	2.32	2.33	2.31	0.38	2.31	2.31	2.31	2.31	2.33	0.48
308	TAG(52:5/FA18:3)+NH4	2.28	2.28	2.29	2.30	2.32	0.70	2.31	2.34	2.29	2.32	2.31	0.81	2.31	2.32	2.31	2.30	2.33	0.47
309	TAG(52:5/FA20:3)+NH4	2.28	2.28	2.26	2.30	2.30	0.76	2.34	2.36	2.33	2.36	2.33	0.63	2.27	2.31	2.32	2.38	2.33	1.66
310	TAG(52:5/FA20:4)+NH4	2.30	2.28	2.29	2.32	2.32	0.74	2.35	2.35	2.32	2.31	2.34	0.76	2.32	2.32	2.35	2.30	2.33	0.75
311	TAG(52:5/FA20:5)+NH4	2.29	2.30	2.32	2.34	2.32	0.87	2.34	2.32	2.34	2.32	2.36	0.70	2.38	2.34	2.36	2.36	2.39	0.77
312	TAG(52:5/FA22:5)+NH4	2.29	2.29	2.33	2.33	2.32	0.86	2.31	2.34	2.37	2.31	2.37	1.40	2.29	2.32	2.35	2.40	2.34	1.86
313	TAG(52:6/FA14:0)+NH4	2.26	2.30	2.28	2.24	2.31	1.22	2.34	2.26	2.32	2.34	2.33	1.42	2.30	2.44	2.34	2.34	2.40	2.27

314	TAG(52:6/FA16:0)+NH4	2.28	2.26	2.28	2.30	2.31	0.79	2.34	2.33	2.27	2.33	2.30	1.32	2.31	2.32	2.31	2.30	2.37	1.20
315	TAG(52:6/FA16:1)+NH4	2.25	2.25	2.30	2.29	2.28	0.95	2.31	2.30	2.28	2.32	2.25	1.22	2.27	2.30	2.31	2.30	2.33	0.93
316	TAG(52:6/FA18:1)+NH4	2.26	2.26	2.21	2.31	2.25	1.50	2.37	2.38	2.40	2.35	2.42	1.10	2.29	2.36	2.38	2.34	2.37	1.52
317	TAG(52:6/FA18:2)+NH4	2.24	2.24	2.26	2.27	2.27	0.68	2.30	2.32	2.28	2.29	2.28	0.66	2.30	2.30	2.28	2.26	2.29	0.88
318	TAG(52:6/FA18:3)+NH4	2.25	2.25	2.26	2.29	2.28	0.75	2.26	2.32	2.24	2.28	2.25	1.38	2.22	2.30	2.26	2.26	2.30	1.59
319	TAG(52:6/FA20:4)+NH4	2.27	2.22	2.29	2.30	2.28	1.43	2.31	2.27	2.26	2.32	2.32	1.15	2.31	2.28	2.26	2.27	2.32	1.15
320	TAG(52:6/FA20:5)+NH4	2.27	2.30	2.25	2.27	2.29	0.86	2.30	2.29	2.28	2.29	2.25	0.82	2.31	2.30	2.32	2.27	2.30	0.88
321	TAG(52:6/FA22:6)+NH4	2.28	2.25	2.26	2.33	2.26	1.44	2.34	2.29	2.32	2.33	2.34	0.96	2.28	2.35	2.36	2.31	2.32	1.26
322	TAG(52:7/FA16:0)+NH4	2.31	2.31	2.31	2.34	2.30	0.59	2.34	2.36	2.31	2.32	2.43	1.95	2.34	2.37	2.37	2.34	2.41	1.14
323	TAG(52:7/FA18:1)+NH4	2.39	2.43	2.40	2.39	2.37	0.83	2.48	2.43	2.44	2.45	2.44	0.78	2.42	2.46	2.49	2.43	2.45	1.06
324	TAG(52:7/FA20:5)+NH4	2.28	2.23	2.31	2.31	2.31	1.54	2.35	2.37	2.39	2.32	2.39	1.28	2.41	2.35	2.37	2.32	2.34	1.45
325	TAG(52:7/FA22:6)+NH4	2.29	2.31	2.29	2.32	2.33	0.84	2.31	2.30	2.29	2.37	2.34	1.32	2.30	2.31	2.30	2.34	2.32	0.60
326	TAG(52:8/FA16:1)+NH4	2.33	2.36	2.34	2.34	2.30	0.96	2.34	2.32	2.34	2.35	2.29	1.07	2.20	2.37	2.33	2.37	2.33	3.09
327	TAG(52:8/FA18:2)+NH4	2.39	2.33	2.43	2.41	2.40	1.66	2.46	2.50	2.42	2.37	2.37	2.34	2.44	2.36	2.41	2.40	2.38	1.24
328	TAG(53:0/FA16:0)+NH4	2.28	2.27	2.28	2.30	2.30	0.58	2.34	2.34	2.34	2.27	2.30	1.31	2.30	2.32	2.32	2.32	2.31	0.42
329	TAG(53:1/FA16:0)+NH4	2.31	2.31	2.27	2.33	2.32	0.94	2.29	2.33	2.32	2.28	2.31	0.96	2.31	2.36	2.33	2.34	2.30	0.99
330	TAG(53:1/FA17:0)+NH4	2.41	2.45	2.44	2.49	2.45	1.21	2.51	2.50	2.50	2.51	2.44	1.18	2.51	2.52	2.54	2.51	2.52	0.52
331	TAG(53:1/FA18:0)+NH4	2.42	2.44	2.43	2.42	2.44	0.35	2.43	2.46	2.42	2.44	2.40	0.88	2.46	2.52	2.44	2.42	2.50	1.58
332	TAG(53:1/FA18:1)+NH4	2.31	2.34	2.35	2.35	2.36	0.68	2.35	2.33	2.38	2.35	2.40	1.07	2.36	2.35	2.39	2.39	2.41	1.09
333	TAG(53:2/FA16:0)+NH4	2.26	2.23	2.32	2.32	2.29	1.66	2.31	2.29	2.28	2.27	2.28	0.70	2.30	2.31	2.30	2.26	2.33	1.15
334	TAG(53:2/FA17:0)+NH4	2.39	2.43	2.39	2.39	2.45	1.13	2.47	2.45	2.45	2.46	2.45	0.29	2.44	2.43	2.44	2.42	2.44	0.36
335	TAG(53:2/FA18:1)+NH4	2.36	2.39	2.36	2.39	2.41	0.98	2.36	2.38	2.36	2.37	2.38	0.35	2.39	2.38	2.40	2.44	2.40	1.01
336	TAG(53:2/FA18:2)+NH4	2.34	2.34	2.32	2.34	2.37	0.82	2.33	2.33	2.32	2.36	2.31	0.82	2.32	2.34	2.39	2.29	2.35	1.61
337	TAG(53:3/FA16:0)+NH4	2.27	2.29	2.27	2.30	2.35	1.33	2.27	2.28	2.29	2.28	2.29	0.32	2.28	2.32	2.28	2.30	2.33	0.84
338	TAG(53:3/FA17:0)+NH4	2.34	2.37	2.34	2.38	2.36	0.68	2.40	2.38	2.41	2.39	2.41	0.47	2.40	2.44	2.41	2.40	2.39	0.82
339	TAG(53:3/FA18:2)+NH4	2.32	2.34	2.33	2.34	2.34	0.29	2.31	2.36	2.35	2.36	2.37	1.04	2.35	2.37	2.36	2.36	2.34	0.51
340	TAG(53:4/FA16:0)+NH4	2.27	2.29	2.27	2.32	2.30	0.99	2.25	2.25	2.32	2.31	2.26	1.47	2.28	2.27	2.29	2.32	2.27	0.88
341	TAG(53:4/FA17:0)+NH4	2.30	2.31	2.32	2.35	2.33	0.96	2.37	2.33	2.33	2.36	2.36	0.82	2.34	2.33	2.36	2.38	2.37	0.95
342	TAG(53:4/FA18:2)+NH4	2.32	2.31	2.34	2.30	2.31	0.66	2.32	2.30	2.32	2.31	2.32	0.32	2.33	2.34	2.34	2.31	2.37	0.95

343	TAG(53:4/FA18:3)+NH4	2.31	2.32	2.31	2.32	2.33	0.40	2.32	2.30	2.31	2.37	2.29	1.39	2.33	2.35	2.31	2.35	2.37	0.87
344	TAG(53:4/FA20:4)+NH4	2.33	2.29	2.36	2.35	2.31	1.21	2.37	2.41	2.38	2.33	2.42	1.50	2.36	2.41	2.38	2.40	2.37	0.89
345	TAG(53:5/FA20:4)+NH4	2.35	2.32	2.32	2.32	2.35	0.62	2.35	2.41	2.34	2.44	2.34	1.96	2.36	2.37	2.38	2.38	2.40	0.61
346	TAG(53:6/FA20:4)+NH4	2.28	2.31	2.26	2.29	2.27	0.85	2.46	2.42	2.27	2.33	2.30	3.45	2.35	2.34	2.31	2.33	2.25	1.73
347	TAG(54:0/FA16:0)+NH4	2.52	2.52	2.55	2.55	2.56	0.74	3.20	3.17	3.13	3.15	3.17	0.83	2.49	3.24	2.48	2.55	3.15	13.57
348	TAG(54:0/FA18:0)+NH4	2.56	2.57	2.61	2.57	2.63	1.13	3.22	3.22	3.20	3.23	3.24	0.46	2.60	3.24	2.52	2.57	3.23	13.11
349	TAG(54:1/FA16:0)+NH4	2.56	2.56	2.58	2.57	2.60	0.63	3.11	3.06	2.98	3.07	2.95	2.20	2.48	2.48	2.53	2.58	2.53	1.65
350	TAG(54:1/FA18:0)+NH4	2.53	2.55	2.59	2.59	2.53	1.13	2.60	2.63	2.58	2.57	2.61	0.94	2.60	2.50	2.54	2.60	2.57	1.61
351	TAG(54:1/FA18:1)+NH4	2.50	2.51	2.56	2.56	2.55	1.13	2.62	2.57	2.59	2.59	2.64	1.03	2.82	2.70	2.53	2.48	2.56	5.30
352	TAG(54:1/FA20:0)+NH4	2.25	2.32	2.36	2.32	2.37	2.06	2.31	2.26	2.31	2.28	2.29	0.99	2.31	2.23	2.35	2.35	2.27	2.22
353	TAG(54:1/FA20:1)+NH4	2.41	2.45	2.46	2.42	2.54	2.17	2.44	2.37	2.39	2.39	2.32	1.94	2.40	2.54	2.49	2.46	2.47	2.17
354	TAG(54:2/FA16:0)+NH4	2.38	2.46	2.49	2.43	2.44	1.61	2.39	2.39	2.41	2.37	2.39	0.53	2.42	2.44	2.43	2.47	2.47	1.05
355	TAG(54:2/FA18:0)+NH4	2.47	2.52	2.53	2.52	2.55	1.19	2.61	2.67	2.60	2.59	2.55	1.63	2.55	2.54	2.59	2.61	2.53	1.25
356	TAG(54:2/FA18:1)+NH4	2.46	2.49	2.53	2.52	2.51	1.11	2.57	2.67	2.60	2.58	2.54	1.83	2.53	2.52	2.47	2.51	2.50	1.00
357	TAG(54:2/FA18:2)+NH4	2.46	2.49	2.47	2.41	2.48	1.19	2.51	2.55	2.52	2.51	2.49	0.87	2.49	2.50	2.48	2.52	2.48	0.59
358	TAG(54:2/FA20:0)+NH4	2.35	2.35	2.35	2.36	2.32	0.58	2.29	2.27	2.25	2.29	2.31	0.99	2.32	2.28	2.31	2.32	2.31	0.76
359	TAG(54:2/FA20:1)+NH4	2.43	2.43	2.44	2.44	2.46	0.42	2.38	2.36	2.35	2.35	2.34	0.67	2.43	2.42	2.40	2.46	2.45	1.00
360	TAG(54:2/FA20:2)+NH4	2.41	2.49	2.46	2.45	2.44	1.29	2.44	2.49	2.44	2.48	2.42	1.12	2.45	2.47	2.48	2.53	2.45	1.26
361	TAG(54:3/FA16:0)+NH4	2.37	2.41	2.40	2.42	2.39	0.89	2.38	2.42	2.38	2.40	2.41	0.73	2.42	2.46	2.42	2.46	2.45	0.78
362	TAG(54:3/FA16:1)+NH4	2.37	2.37	2.41	2.36	2.37	0.71	2.31	2.31	2.27	2.25	2.25	1.27	2.30	2.37	2.34	2.30	2.37	1.39
363	TAG(54:3/FA18:0)+NH4	2.39	2.42	2.44	2.42	2.45	0.88	2.48	2.50	2.49	2.48	2.49	0.42	2.45	2.45	2.45	2.47	2.46	0.30
364	TAG(54:3/FA18:1)+NH4	2.40	2.40	2.42	2.46	2.43	0.97	2.44	2.49	2.46	2.47	2.50	0.92	2.46	2.47	2.45	2.47	2.44	0.45
365	TAG(54:3/FA18:2)+NH4	2.37	2.42	2.41	2.43	2.39	0.92	2.44	2.45	2.46	2.48	2.46	0.62	2.44	2.43	2.46	2.48	2.43	0.91
366	TAG(54:3/FA18:3)+NH4	2.36	2.38	2.39	2.39	2.35	0.72	2.37	2.37	2.37	2.40	2.36	0.73	2.41	2.44	2.43	2.35	2.45	1.72
367	TAG(54:3/FA20:1)+NH4	2.37	2.38	2.38	2.39	2.38	0.35	2.34	2.36	2.33	2.35	2.35	0.39	2.37	2.37	2.37	2.38	2.38	0.10
368	TAG(54:3/FA20:2)+NH4	2.40	2.40	2.40	2.41	2.41	0.19	2.41	2.42	2.45	2.42	2.42	0.66	2.43	2.44	2.43	2.44	2.42	0.22
369	TAG(54:3/FA20:3)+NH4	2.40	2.40	2.42	2.39	2.38	0.59	2.45	2.44	2.46	2.46	2.43	0.58	2.34	2.43	2.46	2.40	2.41	1.77
370	TAG(54:4/FA16:0)+NH4	2.33	2.34	2.37	2.37	2.38	0.94	2.39	2.39	2.40	2.37	2.41	0.53	2.38	2.43	2.41	2.41	2.43	0.78
371	TAG(54:4/FA16:1)+NH4	2.35	2.32	2.34	2.36	2.34	0.56	2.29	2.33	2.33	2.34	2.29	1.13	2.34	2.33	2.32	2.30	2.38	1.26

372	TAG(54:4/FA18:0)+NH4	2.35	2.33	2.36	2.37	2.36	0.59	2.37	2.41	2.39	2.41	2.42	0.89	2.36	2.41	2.49	2.48	2.40	2.27
373	TAG(54:4/FA18:1)+NH4	2.33	2.35	2.33	2.36	2.36	0.67	2.38	2.41	2.39	2.43	2.40	0.71	2.39	2.38	2.38	2.39	2.39	0.14
374	TAG(54:4/FA18:2)+NH4	2.32	2.33	2.33	2.35	2.36	0.66	2.38	2.40	2.37	2.38	2.39	0.41	2.38	2.37	2.37	2.41	2.38	0.59
375	TAG(54:4/FA18:3)+NH4	2.34	2.33	2.33	2.36	2.37	0.73	2.34	2.35	2.35	2.32	2.34	0.51	2.35	2.35	2.37	2.36	2.37	0.39
376	TAG(54:4/FA20:1)+NH4	2.33	2.33	2.34	2.35	2.32	0.41	2.35	2.37	2.32	2.25	2.32	1.91	2.34	2.34	2.32	2.31	2.34	0.56
377	TAG(54:4/FA20:2)+NH4	2.33	2.33	2.34	2.37	2.35	0.66	2.34	2.35	2.35	2.36	2.35	0.22	2.37	2.36	2.35	2.38	2.39	0.57
378	TAG(54:4/FA20:3)+NH4	2.35	2.35	2.35	2.36	2.37	0.41	2.41	2.41	2.40	2.42	2.39	0.52	2.39	2.40	2.40	2.39	2.40	0.19
379	TAG(54:4/FA20:4)+NH4	2.35	2.37	2.38	2.37	2.35	0.51	2.44	2.42	2.41	2.41	2.41	0.54	2.40	2.40	2.41	2.39	2.41	0.28
380	TAG(54:4/FA22:4)+NH4	2.32	2.37	2.39	2.36	2.38	1.22	2.39	2.37	2.39	2.38	2.34	0.86	2.39	2.41	2.43	2.40	2.41	0.71
381	TAG(54:5/FA16:0)+NH4	2.29	2.31	2.32	2.34	2.32	0.65	2.36	2.36	2.35	2.37	2.36	0.34	2.32	2.34	2.36	2.34	2.34	0.60
382	TAG(54:5/FA16:1)+NH4	2.28	2.27	2.28	2.33	2.31	1.06	2.31	2.30	2.34	2.34	2.33	0.75	2.34	2.34	2.31	2.33	2.35	0.77
383	TAG(54:5/FA18:0)+NH4	2.28	2.30	2.30	2.32	2.29	0.63	2.36	2.34	2.30	2.29	2.32	1.21	2.32	2.35	2.37	2.33	2.36	0.71
384	TAG(54:5/FA18:1)+NH4	2.29	2.28	2.29	2.28	2.30	0.40	2.34	2.35	2.35	2.33	2.35	0.30	2.34	2.35	2.35	2.34	2.34	0.26
385	TAG(54:5/FA18:2)+NH4	2.27	2.27	2.29	2.28	2.32	0.88	2.34	2.34	2.35	2.32	2.30	0.77	2.33	2.32	2.34	2.32	2.34	0.43
386	TAG(54:5/FA18:3)+NH4	2.28	2.29	2.31	2.31	2.31	0.52	2.32	2.32	2.28	2.32	2.28	1.02	2.31	2.32	2.34	2.33	2.34	0.46
387	TAG(54:5/FA20:2)+NH4	2.30	2.29	2.31	2.34	2.30	0.82	2.32	2.35	2.32	2.29	2.24	1.90	2.27	2.31	2.38	2.32	2.37	1.94
388	TAG(54:5/FA20:3)+NH4	2.31	2.29	2.29	2.29	2.31	0.47	2.32	2.35	2.32	2.34	2.30	0.86	2.31	2.36	2.34	2.33	2.33	0.65
389	TAG(54:5/FA20:4)+NH4	2.31	2.32	2.33	2.31	2.35	0.65	2.34	2.35	2.35	2.37	2.34	0.46	2.32	2.36	2.38	2.36	2.34	1.01
390	TAG(54:5/FA20:5)+NH4	2.30	2.29	2.33	2.30	2.33	0.75	2.36	2.37	2.34	2.29	2.32	1.44	2.37	2.34	2.30	2.41	2.36	1.66
391	TAG(54:5/FA22:4)+NH4	2.28	2.31	2.29	2.31	2.33	0.90	2.32	2.40	2.34	2.31	2.31	1.54	2.32	2.35	2.34	2.29	2.34	1.04
392	TAG(54:5/FA22:5)+NH4	2.33	2.31	2.31	2.33	2.37	0.98	2.33	2.36	2.32	2.32	2.39	1.39	2.34	2.34	2.37	2.37	2.35	0.80
393	TAG(54:6/FA16:0)+NH4	2.26	2.27	2.29	2.29	2.29	0.69	2.31	2.33	2.29	2.30	2.31	0.62	2.32	2.33	2.29	2.30	2.32	0.62
394	TAG(54:6/FA16:1)+NH4	2.25	2.27	2.27	2.30	2.32	1.12	2.31	2.32	2.26	2.31	2.35	1.50	2.28	2.34	2.33	2.31	2.34	1.13
395	TAG(54:6/FA18:1)+NH4	2.25	2.26	2.28	2.27	2.30	0.94	2.32	2.32	2.27	2.26	2.31	1.19	2.31	2.33	2.31	2.33	2.33	0.44
396	TAG(54:6/FA18:2)+NH4	2.25	2.27	2.28	2.29	2.30	0.95	2.26	2.30	2.29	2.27	2.30	0.91	2.28	2.33	2.29	2.28	2.31	0.92
397	TAG(54:6/FA18:3)+NH4	2.25	2.25	2.31	2.29	2.30	1.28	2.29	2.32	2.28	2.26	2.31	1.07	2.29	2.31	2.27	2.29	2.30	0.57
398	TAG(54:6/FA20:3)+NH4	2.29	2.24	2.29	2.30	2.28	1.03	2.30	2.31	2.26	2.32	2.33	1.15	2.33	2.35	2.34	2.32	2.35	0.42
399	TAG(54:6/FA20:4)+NH4	2.28	2.27	2.30	2.30	2.31	0.68	2.32	2.34	2.31	2.29	2.31	0.74	2.31	2.33	2.32	2.30	2.34	0.69
400	TAG(54:6/FA20:5)+NH4	2.27	2.27	2.31	2.31	2.30	0.87	2.31	2.31	2.30	2.30	2.31	0.27	2.30	2.34	2.30	2.33	2.33	0.73

401	TAG(54:6/FA22:5)+NH4	2.27	2.24	2.29	2.31	2.30	1.09	2.34	2.32	2.34	2.30	2.33	0.80	2.28	2.28	2.41	2.43	2.31	3.05
402	TAG(54:6/FA22:6)+NH4	2.27	2.28	2.30	2.30	2.33	0.94	2.28	2.31	2.32	2.28	2.29	0.81	2.31	2.31	2.31	2.31	2.31	0.10
403	TAG(54:7/FA16:1)+NH4	2.23	2.22	2.29	2.27	2.28	1.33	2.30	2.33	2.32	2.31	2.24	1.54	2.32	2.34	2.31	2.33	2.38	1.12
404	TAG(54:7/FA18:1)+NH4	2.24	2.28	2.31	2.31	2.31	1.33	2.35	2.31	2.33	2.31	2.29	0.92	2.30	2.32	2.33	2.31	2.33	0.56
405	TAG(54:7/FA18:2)+NH4	2.24	2.24	2.27	2.25	2.28	0.74	2.23	2.28	2.26	2.22	2.24	1.07	2.26	2.28	2.25	2.30	2.28	0.89
406	TAG(54:7/FA18:3)+NH4	2.24	2.19	2.26	2.25	2.27	1.42	2.25	2.28	2.22	2.17	2.23	1.92	2.24	2.26	2.18	2.25	2.28	1.78
407	TAG(54:7/FA20:4)+NH4	2.26	2.27	2.27	2.26	2.29	0.61	2.26	2.29	2.29	2.29	2.31	0.75	2.28	2.29	2.25	2.27	2.29	0.71
408	TAG(54:7/FA20:5)+NH4	2.24	2.26	2.29	2.28	2.28	0.97	2.29	2.29	2.25	2.26	2.31	1.14	2.30	2.26	2.31	2.28	2.29	0.83
409	TAG(54:7/FA22:5)+NH4	2.26	2.25	2.29	2.25	2.29	0.97	2.26	2.32	2.27	2.31	2.31	1.31	2.24	2.33	2.32	2.25	2.36	2.22
410	TAG(54:7/FA22:6)+NH4	2.26	2.25	2.27	2.29	2.29	0.74	2.28	2.31	2.26	2.27	2.29	0.91	2.26	2.26	2.27	2.28	2.26	0.43
411	TAG(54:8/FA18:2)+NH4	2.26	2.24	2.27	2.28	2.26	0.57	2.33	2.31	2.26	2.36	2.28	1.81	2.30	2.36	2.24	2.33	2.27	2.11
412	TAG(54:8/FA18:3)+NH4	2.22	2.24	2.28	2.24	2.25	0.91	2.23	2.26	2.17	2.26	2.25	1.71	2.24	2.22	2.20	2.22	2.31	1.77
413	TAG(54:8/FA20:4)+NH4	2.25	2.23	2.26	2.19	2.29	1.64	2.34	2.27	2.21	2.18	2.31	2.83	2.19	2.24	2.27	2.28	2.23	1.57
414	TAG(54:8/FA20:5)+NH4	2.23	2.24	2.26	2.27	2.28	0.98	2.33	2.32	2.27	2.24	2.32	1.64	2.27	2.25	2.24	2.28	2.30	0.98
415	TAG(54:8/FA22:6)+NH4	2.25	2.23	2.25	2.29	2.28	1.12	2.32	2.31	2.29	2.31	2.30	0.52	2.28	2.28	2.29	2.31	2.31	0.58
416	TAG(55:1/FA16:0)+NH4	2.25	2.23	2.28	2.27	2.29	1.01	2.30	2.29	2.28	2.25	2.29	0.77	2.30	2.32	2.27	2.28	2.35	1.29
417	TAG(55:1/FA18:1)+NH4	2.25	2.27	2.28	2.31	2.30	1.02	2.28	2.28	2.30	2.29	2.30	0.38	2.28	2.33	2.32	2.36	2.32	1.18
418	TAG(55:2/FA18:1)+NH4	2.27	2.26	2.26	2.35	2.32	1.66	2.30	2.27	2.23	2.26	2.30	1.31	2.29	2.33	2.26	2.25	2.32	1.61
419	TAG(55:2/FA18:2)+NH4	2.25	2.28	2.26	2.27	2.29	0.75	2.30	2.36	2.32	2.33	2.32	0.88	2.26	2.29	2.28	2.34	2.36	1.82
420	TAG(55:3/FA18:1)+NH4	2.30	2.26	2.33	2.32	2.35	1.56	2.26	2.29	2.27	2.26	2.27	0.51	2.27	2.30	2.27	2.27	2.32	1.04
421	TAG(55:3/FA18:2)+NH4	2.29	2.29	2.29	2.28	2.34	1.14	2.34	2.30	2.26	2.32	2.26	1.57	2.35	2.34	2.36	2.34	2.37	0.50
422	TAG(55:4/FA18:1)+NH4	2.28	2.31	2.32	2.27	2.34	1.14	2.30	2.29	2.25	2.27	2.28	0.80	2.32	2.32	2.28	2.31	2.29	0.75
423	TAG(55:4/FA18:2)+NH4	2.27	2.27	2.27	2.28	2.30	0.57	2.29	2.28	2.24	2.22	2.29	1.40	2.28	2.32	2.24	2.28	2.28	1.24
424	TAG(55:5/FA18:1)+NH4	2.28	2.24	2.26	2.31	2.29	1.17	2.30	2.31	2.28	2.27	2.27	0.73	2.28	2.33	2.32	2.31	2.32	0.86
425	TAG(55:5/FA18:2)+NH4	2.24	2.22	2.26	2.27	2.29	1.11	2.31	2.34	2.24	2.26	2.29	1.69	2.28	2.32	2.27	2.24	2.31	1.44
426	TAG(55:5/FA20:4)+NH4	2.31	2.34	2.33	2.31	2.35	0.74	2.29	2.32	2.36	2.41	2.35	1.85	2.32	2.37	2.33	2.33	2.36	0.81
427	TAG(55:7/FA22:6)+NH4	2.27	2.25	2.31	2.28	2.23	1.42	2.26	2.33	2.28	2.29	2.33	1.38	2.28	2.34	2.26	2.27	2.30	1.42
428	TAG(56:10/FA18:2)+NH4	2.30	2.33	2.31	2.31	2.34	0.73	2.24	2.30	2.29	2.23	2.28	1.41	2.29	2.37	2.34	2.27	2.32	1.72

429	TAG(56:1/FA16:0)+NH4	2.60	2.59	2.61	2.62	2.59	0.42	2.68	2.61	2.62	2.62	2.65	1.10	3.26	3.27	3.25	3.22	3.23	0.53
430	TAG(56:1/FA18:1)+NH4	2.58	2.60	2.59	2.53	2.56	0.96	2.58	2.56	2.60	2.57	2.62	0.87	3.21	3.18	3.23	3.18	3.14	1.09
431	TAG(56:2/FA16:0)+NH4	2.57	2.55	2.59	2.59	2.55	0.78	2.63	2.72	2.65	2.62	2.67	1.51	3.01	3.12	3.09	3.05	3.13	1.69
432	TAG(56:2/FA18:0)+NH4	2.45	2.50	2.51	2.47	2.41	1.58	2.35	2.20	2.30	2.33	2.30	2.51	2.35	2.51	2.30	2.59	2.40	4.86
433	TAG(56:2/FA20:0)+NH4	2.30	2.32	2.30	2.33	2.32	0.54	2.33	2.28	2.29	2.26	2.28	1.05	2.26	2.29	2.31	2.30	2.27	0.86
434	TAG(56:2/FA20:1)+NH4	2.44	2.38	2.44	2.44	2.44	1.01	2.32	2.26	2.28	2.24	2.23	1.59	2.31	2.37	2.34	2.32	2.39	1.56
435	TAG(56:3/FA16:0)+NH4	2.48	2.50	2.49	2.50	2.49	0.33	2.52	2.54	2.62	2.55	2.57	1.54	2.59	2.54	2.68	2.58	2.58	1.98
436	TAG(56:3/FA18:0)+NH4	2.44	2.48	2.46	2.52	2.53	1.48	2.48	2.49	2.47	2.47	2.42	1.04	2.47	2.49	2.58	2.49	2.49	1.82
437	TAG(56:3/FA18:1)+NH4	2.42	2.42	2.44	2.45	2.44	0.56	2.31	2.30	2.20	2.19	2.30	2.69	2.36	2.39	2.39	2.38	2.42	0.86
438	TAG(56:3/FA18:2)+NH4	2.48	2.51	2.51	2.51	2.50	0.51	2.53	2.57	2.58	2.55	2.57	0.77	2.60	2.56	2.59	2.63	2.56	1.23
439	TAG(56:3/FA20:0)+NH4	2.34	2.37	2.37	2.38	2.34	0.79	2.31	2.31	2.29	2.29	2.29	0.43	2.26	2.30	2.29	2.27	2.33	1.29
440	TAG(56:3/FA20:1)+NH4	2.38	2.40	2.40	2.45	2.43	1.20	2.34	2.34	2.29	2.28	2.34	1.19	2.38	2.37	2.17	2.38	2.40	4.13
441	TAG(56:3/FA20:2)+NH4	2.43	2.45	2.39	2.43	2.42	0.93	2.39	2.40	2.33	2.33	2.39	1.44	2.39	2.58	2.51	2.42	2.43	3.08
442	TAG(56:4/FA16:0)+NH4	2.39	2.42	2.38	2.41	2.41	0.70	2.49	2.45	2.42	2.46	2.48	1.14	2.44	2.45	2.46	2.47	2.49	0.80
443	TAG(56:4/FA18:0)+NH4	2.38	2.40	2.40	2.42	2.45	1.13	2.47	2.43	2.48	2.45	2.48	0.95	2.48	2.46	2.46	2.46	2.45	0.43
444	TAG(56:4/FA18:1)+NH4	2.35	2.39	2.37	2.39	2.41	1.00	2.39	2.34	2.32	2.36	2.35	0.94	2.40	2.39	2.38	2.37	2.40	0.56
445	TAG(56:4/FA18:2)+NH4	2.36	2.37	2.36	2.37	2.39	0.47	2.37	2.37	2.31	2.32	2.37	1.28	2.38	2.42	2.38	2.40	2.38	0.71
446	TAG(56:4/FA20:1)+NH4	2.33	2.38	2.37	2.40	2.38	1.02	2.36	2.37	2.32	2.37	2.35	0.92	2.36	2.36	2.31	2.35	2.37	0.97
447	TAG(56:4/FA20:2)+NH4	2.36	2.38	2.38	2.37	2.37	0.36	2.38	2.38	2.34	2.36	2.36	0.72	2.37	2.37	2.41	2.38	2.40	0.81
448	TAG(56:4/FA20:3)+NH4	2.36	2.39	2.40	2.39	2.41	0.83	2.46	2.48	2.39	2.49	2.47	1.56	2.44	2.43	2.46	2.47	2.48	0.76
449	TAG(56:4/FA20:4)+NH4	2.38	2.37	2.35	2.41	2.42	1.21	2.47	2.48	2.46	2.51	2.47	0.85	2.45	2.49	2.48	2.44	2.40	1.53
450	TAG(56:4/FA22:4)+NH4	2.38	2.35	2.35	2.38	2.41	1.09	2.40	2.46	2.46	2.38	2.42	1.55	2.40	2.43	2.39	2.40	2.43	0.61
451	TAG(56:5/FA16:0)+NH4	2.33	2.32	2.35	2.34	2.35	0.50	2.35	2.35	2.38	2.34	2.41	1.19	2.35	2.39	2.38	2.37	2.35	0.75
452	TAG(56:5/FA18:0)+NH4	2.34	2.35	2.32	2.35	2.37	0.84	2.39	2.40	2.40	2.40	2.41	0.42	2.40	2.39	2.46	2.42	2.44	1.17
453	TAG(56:5/FA18:1)+NH4	2.34	2.33	2.32	2.36	2.33	0.72	2.39	2.33	2.37	2.42	2.38	1.38	2.35	2.37	2.36	2.39	2.37	0.51
454	TAG(56:5/FA18:2)+NH4	2.29	2.30	2.32	2.33	2.35	0.90	2.35	2.37	2.34	2.31	2.33	0.98	2.33	2.33	2.35	2.35	2.35	0.46
455	TAG(56:5/FA20:1)+NH4	2.31	2.28	2.34	2.33	2.32	1.02	2.32	2.35	2.34	2.33	2.34	0.56	2.31	2.35	2.31	2.31	2.37	1.27
456	TAG(56:5/FA20:2)+NH4	2.31	2.31	2.33	2.32	2.34	0.50	2.38	2.33	2.28	2.33	2.33	1.45	2.33	2.34	2.37	2.38	2.35	0.87
457	TAG(56:5/FA20:3)+NH4	2.32	2.35	2.32	2.34	2.36	0.76	2.39	2.40	2.39	2.36	2.38	0.71	2.36	2.36	2.41	2.40	2.37	0.93

458	TAG(56:5/FA20:4)+NH4	2.33	2.36	2.35	2.36	2.38	0.70	2.41	2.43	2.40	2.40	2.42	0.52	2.38	2.40	2.43	2.40	2.39	0.81
459	TAG(56:5/FA22:4)+NH4	2.33	2.32	2.35	2.34	2.34	0.51	2.40	2.39	2.39	2.39	2.36	0.60	2.37	2.37	2.38	2.38	2.38	0.17
460	TAG(56:5/FA22:5)+NH4	2.31	2.30	2.30	2.36	2.36	1.36	2.37	2.39	2.44	2.40	2.36	1.30	2.36	2.39	2.39	2.40	2.38	0.67
461	TAG(56:6/FA16:0)+NH4	2.28	2.29	2.29	2.30	2.33	0.80	2.30	2.35	2.31	2.35	2.39	1.50	2.34	2.37	2.33	2.36	2.35	0.78
462	TAG(56:6/FA18:0)+NH4	2.25	2.32	2.32	2.33	2.31	1.34	2.36	2.39	2.40	2.27	2.37	2.26	2.38	2.36	2.40	2.36	2.35	0.86
463	TAG(56:6/FA18:1)+NH4	2.28	2.31	2.32	2.31	2.33	0.85	2.35	2.36	2.31	2.34	2.34	0.72	2.33	2.32	2.34	2.36	2.34	0.58
464	TAG(56:6/FA18:2)+NH4	2.29	2.28	2.27	2.30	2.28	0.50	2.33	2.32	2.32	2.34	2.35	0.62	2.33	2.33	2.33	2.32	2.35	0.39
465	TAG(56:6/FA18:3)+NH4	2.24	2.26	2.28	2.27	2.32	1.36	2.34	2.35	2.28	2.38	2.27	2.11	2.30	2.27	2.33	2.28	2.34	1.30
466	TAG(56:6/FA20:2)+NH4	2.24	2.28	2.29	2.27	2.31	1.08	2.28	2.35	2.26	2.33	2.31	1.50	2.29	2.36	2.30	2.36	2.27	1.73
467	TAG(56:6/FA20:3)+NH4	2.25	2.28	2.31	2.30	2.29	0.99	2.33	2.36	2.35	2.33	2.33	0.57	2.32	2.36	2.34	2.33	2.34	0.53
468	TAG(56:6/FA20:4)+NH4	2.29	2.31	2.32	2.31	2.33	0.71	2.35	2.35	2.33	2.34	2.30	0.94	2.33	2.34	2.34	2.35	2.35	0.46
469	TAG(56:6/FA20:5)+NH4	2.30	2.30	2.32	2.31	2.34	0.69	2.29	2.32	2.34	2.34	2.32	0.90	2.34	2.29	2.37	2.33	2.37	1.43
470	TAG(56:6/FA22:4)+NH4	2.27	2.28	2.28	2.29	2.31	0.62	2.33	2.34	2.34	2.33	2.33	0.20	2.35	2.33	2.35	2.32	2.33	0.56
471	TAG(56:6/FA22:5)+NH4	2.28	2.33	2.30	2.31	2.34	1.13	2.36	2.36	2.35	2.34	2.35	0.27	2.35	2.36	2.35	2.34	2.37	0.57
472	TAG(56:6/FA22:6)+NH4	2.28	2.32	2.30	2.29	2.29	0.69	2.31	2.32	2.27	2.26	2.31	1.31	2.28	2.34	2.29	2.27	2.35	1.57
473	TAG(56:7/FA16:0)+NH4	2.26	2.26	2.26	2.28	2.27	0.42	2.30	2.30	2.29	2.28	2.30	0.44	2.30	2.32	2.28	2.31	2.31	0.60
474	TAG(56:7/FA16:1)+NH4	2.24	2.25	2.28	2.26	2.27	0.77	2.32	2.26	2.33	2.28	2.30	1.20	2.31	2.33	2.31	2.29	2.25	1.38
475	TAG(56:7/FA18:0)+NH4	2.26	2.28	2.30	2.33	2.27	1.14	2.22	2.32	2.35	2.30	2.37	2.52	2.28	2.35	2.33	2.33	2.33	1.05
476	TAG(56:7/FA18:1)+NH4	2.25	2.26	2.27	2.27	2.28	0.52	2.31	2.32	2.31	2.31	2.30	0.31	2.29	2.31	2.30	2.30	2.31	0.36
477	TAG(56:7/FA18:2)+NH4	2.24	2.27	2.24	2.26	2.26	0.59	2.29	2.29	2.26	2.27	2.29	0.63	2.29	2.30	2.25	2.30	2.32	1.05
478	TAG(56:7/FA18:3)+NH4	2.24	2.28	2.28	2.25	2.28	0.94	2.31	2.26	2.30	2.32	2.29	1.01	2.30	2.34	2.29	2.31	2.29	0.82
479	TAG(56:7/FA20:3)+NH4	2.25	2.25	2.26	2.28	2.28	0.70	2.30	2.28	2.28	2.31	2.35	1.21	2.31	2.31	2.27	2.32	2.24	1.43
480	TAG(56:7/FA20:4)+NH4	2.24	2.25	2.26	2.28	2.28	0.80	2.27	2.28	2.31	2.32	2.33	1.01	2.30	2.31	2.31	2.29	2.31	0.46
481	TAG(56:7/FA20:5)+NH4	2.24	2.24	2.26	2.29	2.30	1.18	2.28	2.32	2.27	2.26	2.27	0.96	2.29	2.27	2.35	2.29	2.32	1.33
482	TAG(56:7/FA22:4)+NH4	2.26	2.23	2.22	2.25	2.27	0.93	2.31	2.34	2.31	2.26	2.25	1.65	2.31	2.23	2.29	2.29	2.35	1.86
483	TAG(56:7/FA22:5)+NH4	2.25	2.26	2.28	2.28	2.27	0.63	2.26	2.28	2.29	2.29	2.29	0.66	2.31	2.30	2.32	2.28	2.30	0.77
484	TAG(56:7/FA22:6)+NH4	2.22	2.29	2.27	2.30	2.29	1.35	2.28	2.27	2.25	2.28	2.27	0.45	2.26	2.29	2.28	2.30	2.30	0.70
485	TAG(56:8/FA16:0)+NH4	2.23	2.25	2.26	2.30	2.27	1.25	2.27	2.28	2.25	2.23	2.27	0.84	2.27	2.28	2.30	2.29	2.31	0.69
486	TAG(56:8/FA16:1)+NH4	2.23	2.28	2.24	2.28	2.26	0.89	2.27	2.36	2.32	2.36	2.40	2.06	2.31	2.55	2.26	2.43	2.46	4.80

487	TAG(56:8/FA18:1)+NH4	2.25	2.27	2.27	2.24	2.30	0.98	2.27	2.27	2.28	2.29	2.25	0.58	2.27	2.34	2.28	2.31	2.34	1.48
488	TAG(56:8/FA18:2)+NH4	2.22	2.22	2.25	2.23	2.28	1.17	2.28	2.29	2.22	2.26	2.30	1.38	2.23	2.29	2.27	2.30	2.26	1.14
489	TAG(56:8/FA18:3)+NH4	2.21	2.22	2.27	2.22	2.25	1.08	2.19	2.27	2.22	2.19	2.25	1.55	2.25	2.32	2.27	2.30	2.26	1.24
490	TAG(56:8/FA20:4)+NH4	2.22	2.22	2.23	2.24	2.26	0.73	2.26	2.23	2.27	2.27	2.27	0.83	2.26	2.27	2.25	2.24	2.29	0.97
491	TAG(56:8/FA20:5)+NH4	2.22	2.23	2.21	2.25	2.24	0.75	2.25	2.29	2.27	2.28	2.28	0.55	2.29	2.29	2.27	2.24	2.28	0.86
492	TAG(56:8/FA22:5)+NH4	2.18	2.24	2.24	2.25	2.24	1.33	2.30	2.26	2.26	2.32	2.25	1.32	2.22	2.30	2.27	2.25	2.25	1.20
493	TAG(56:8/FA22:6)+NH4	2.22	2.24	2.22	2.25	2.26	0.68	2.28	2.26	2.27	2.26	2.25	0.57	2.27	2.28	2.26	2.28	2.25	0.60
494	TAG(56:9/FA18:3)+NH4	2.21	2.21	2.25	2.27	2.27	1.35	2.31	2.20	2.27	2.33	2.24	2.22	2.24	2.30	2.22	2.27	2.23	1.52
495	TAG(56:9/FA20:4)+NH4	2.20	2.23	2.23	2.23	2.27	1.10	2.19	2.24	2.26	2.25	2.32	2.07	2.25	2.25	2.19	2.26	2.24	1.23
496	TAG(56:9/FA20:5)+NH4	2.18	2.22	2.24	2.20	2.22	0.96	2.26	2.26	2.29	2.30	2.22	1.30	2.24	2.28	2.29	2.24	2.28	1.03
497	TAG(56:9/FA22:6)+NH4	2.19	2.24	2.23	2.23	2.22	0.75	2.24	2.26	2.21	2.25	2.28	1.03	2.21	2.26	2.27	2.22	2.29	1.52
498	TAG(57:10/FA22:6)+NH4	2.32	2.40	2.27	2.25	2.31	2.47	2.41	2.25	2.48	2.41	2.39	3.53	2.29	2.31	2.34	2.37	2.37	1.46
499	TAG(57:2/FA18:1)+NH4	2.25	2.28	2.27	2.23	2.32	1.45	2.28	2.26	2.24	2.24	2.29	1.02	2.26	2.28	2.29	2.27	2.20	1.61
500	TAG(57:3/FA18:2)+NH4	2.27	2.29	2.31	2.34	2.34	1.38	2.34	2.39	2.31	2.39	2.38	1.51	2.39	2.37	2.45	2.32	2.36	2.04
501	TAG(58:10/FA18:2)+NH4	2.26	2.28	2.26	2.27	2.29	0.46	2.26	2.31	2.32	2.28	2.33	1.29	2.26	2.33	2.35	2.33	2.31	1.46
502	TAG(58:10/FA20:4)+NH4	2.23	2.24	2.22	2.20	2.22	0.70	2.16	2.22	2.26	2.17	2.29	2.50	2.30	2.28	2.32	2.21	2.28	1.89
503	TAG(58:10/FA20:5)+NH4	2.21	2.24	2.21	2.23	2.27	1.12	2.27	2.18	2.24	2.32	2.28	2.41	2.24	2.22	2.17	2.27	2.36	3.25
504	TAG(58:10/FA22:5)+NH4	2.22	2.19	2.18	2.19	2.29	2.04	2.20	2.24	2.30	2.38	2.28	2.97	2.27	2.27	2.27	2.23	2.22	1.02
505	TAG(58:10/FA22:6)+NH4	2.19	2.21	2.24	2.23	2.21	0.89	2.21	2.26	2.24	2.24	2.27	1.02	2.25	2.20	2.24	2.26	2.32	1.95
506	TAG(58:2/FA18:1)+NH4	3.15	3.17	3.18	3.19	3.18	0.43	2.63	2.64	2.63	2.72	2.68	1.40	3.24	3.23	3.26	3.24	3.25	0.39
507	TAG(58:3/FA18:1)+NH4	2.54	2.55	2.56	2.56	2.55	0.37	2.63	2.61	2.67	2.66	2.65	1.01	2.98	3.09	3.12	3.09	3.14	2.07
508	TAG(58:5/FA18:1)+NH4	2.31	2.39	2.41	2.38	2.39	1.59	2.44	2.36	2.38	2.43	2.39	1.44	2.42	2.37	2.42	2.40	2.41	0.82
509	TAG(58:6/FA16:0)+NH4	2.30	2.31	2.31	2.31	2.36	1.08	2.34	2.39	2.35	2.33	2.35	0.89	2.38	2.36	2.39	2.34	2.32	1.19
510	TAG(58:6/FA18:0)+NH4	2.31	2.33	2.38	2.36	2.33	1.27	2.38	2.45	2.42	2.39	2.42	1.13	2.34	2.37	2.38	2.35	2.40	1.02
511	TAG(58:6/FA18:1)+NH4	2.32	2.32	2.33	2.33	2.31	0.29	2.37	2.36	2.38	2.35	2.38	0.61	2.32	2.35	2.34	2.37	2.32	0.96
512	TAG(58:6/FA20:4)+NH4	2.32	2.32	2.38	2.38	2.35	1.35	2.38	2.38	2.36	2.34	2.37	0.77	2.38	2.32	2.39	2.38	2.39	1.21
513	TAG(58:6/FA22:4)+NH4	2.28	2.32	2.30	2.35	2.32	1.18	2.40	2.35	2.37	2.37	2.38	0.75	2.36	2.39	2.36	2.33	2.37	0.92

514	TAG(58:6/FA22:5)+NH4	2.32	2.34	2.32	2.36	2.35	0.79	2.37	2.37	2.36	2.37	2.36	0.37	2.32	2.39	2.33	2.40	2.37	1.47
515	TAG(58:7/FA16:0)+NH4	2.32	2.29	2.29	2.32	2.31	0.61	2.28	2.31	2.32	2.38	2.30	1.62	2.34	2.29	2.33	2.28	2.31	1.05
516	TAG(58:7/FA18:0)+NH4	2.31	2.27	2.30	2.29	2.34	1.11	2.35	2.36	2.28	2.34	2.41	1.97	2.32	2.40	2.29	2.34	2.23	2.72
517	TAG(58:7/FA18:1)+NH4	2.28	2.27	2.30	2.27	2.30	0.63	2.32	2.32	2.27	2.33	2.31	1.06	2.32	2.28	2.31	2.31	2.30	0.66
518	TAG(58:7/FA18:2)+NH4	2.23	2.26	2.28	2.29	2.28	1.01	2.31	2.34	2.34	2.37	2.28	1.48	2.32	2.30	2.33	2.30	2.34	0.69
519	TAG(58:7/FA20:4)+NH4	2.25	2.31	2.32	2.31	2.33	1.37	2.39	2.45	2.37	2.37	2.34	1.82	2.37	2.28	2.33	2.34	2.35	1.49
520	TAG(58:7/FA22:4)+NH4	2.26	2.27	2.26	2.25	2.33	1.39	2.30	2.29	2.29	2.29	2.29	0.16	2.28	2.28	2.29	2.29	2.31	0.57
521	TAG(58:7/FA22:5)+NH4	2.27	2.27	2.29	2.30	2.29	0.60	2.34	2.30	2.32	2.33	2.36	1.02	2.30	2.31	2.32	2.31	2.30	0.31
522	TAG(58:7/FA22:6)+NH4	2.28	2.28	2.30	2.28	2.31	0.69	2.25	2.28	2.28	2.29	2.24	1.01	2.28	2.30	2.26	2.26	2.30	0.84
523	TAG(58:8/FA18:1)+NH4	2.22	2.25	2.23	2.28	2.24	0.96	2.25	2.28	2.24	2.29	2.31	1.28	2.30	2.35	2.24	2.25	2.35	2.26
524	TAG(58:8/FA18:2)+NH4	2.24	2.24	2.22	2.25	2.27	0.82	2.24	2.17	2.28	2.28	2.27	2.12	2.26	2.28	2.29	2.32	2.25	1.25
525	TAG(58:8/FA20:3)+NH4	2.23	2.24	2.29	2.26	2.25	1.02	2.21	2.39	2.30	2.19	2.36	3.87	2.26	2.31	2.26	2.38	2.28	2.10
526	TAG(58:8/FA20:4)+NH4	2.23	2.24	2.27	2.27	2.25	0.81	2.33	2.29	2.29	2.22	2.32	1.88	2.33	2.18	2.33	2.34	2.29	2.84
527	TAG(58:8/FA22:5)+NH4	2.21	2.27	2.25	2.24	2.26	1.07	2.30	2.29	2.25	2.28	2.25	0.99	2.27	2.27	2.32	2.27	2.28	1.01
528	TAG(58:8/FA22:6)+NH4	2.26	2.27	2.23	2.25	2.28	0.81	2.27	2.27	2.24	2.26	2.27	0.59	2.26	2.26	2.23	2.25	2.27	0.59
529	TAG(58:9/FA18:1)+NH4	2.22	2.27	2.24	2.26	2.28	1.13	2.26	2.30	2.27	2.28	2.29	0.71	2.31	2.33	2.37	2.30	2.34	1.28
530	TAG(58:9/FA18:2)+NH4	2.21	2.27	2.25	2.24	2.23	0.96	2.32	2.27	2.25	2.30	2.35	1.67	2.29	2.27	2.32	2.29	2.34	1.18
531	TAG(58:9/FA20:4)+NH4	2.23	2.21	2.23	2.21	2.26	0.92	2.26	2.22	2.29	2.26	2.28	1.20	2.31	2.30	2.29	2.24	2.24	1.51
532	TAG(58:9/FA22:5)+NH4	2.17	2.23	2.21	2.24	2.23	1.14	2.19	2.26	2.22	2.30	2.28	2.07	2.29	2.20	2.22	2.27	2.24	1.54
533	TAG(58:9/FA22:6)+NH4	2.23	2.22	2.20	2.21	2.21	0.38	2.23	2.26	2.26	2.23	2.27	0.86	2.25	2.23	2.30	2.25	2.30	1.46
534	TAG(60:10/FA22:5)+NH4	2.22	2.20	2.21	2.26	2.26	1.39	2.26	2.21	2.26	2.29	2.33	1.95	2.39	2.15	2.16	2.34	2.27	4.69
535	TAG(60:10/FA22:6)+NH4	2.21	2.21	2.18	2.20	2.23	0.75	2.29	2.29	2.26	2.35	2.26	1.61	2.31	2.32	2.22	2.20	2.30	2.36
536	TAG(60:11/FA22:5)+NH4	2.23	2.30	2.23	2.19	2.24	1.73	2.22	2.25	2.33	2.32	2.34	2.33	2.33	2.25	2.21	2.30	2.15	3.23
537	TAG(60:11/FA22:6)+NH4	2.21	2.20	2.17	2.22	2.22	0.84	2.30	2.28	2.32	2.30	2.38	1.53	2.22	2.16	2.23	2.28	2.25	1.99
538	TAG(60:12/FA22:6)+NH4	2.19	2.21	2.16	2.21	2.18	0.98	2.24	2.20	2.34	2.30	2.26	2.30	2.20	2.28	2.21	2.31	2.20	2.30
539	DAG(14:0/14:0)+NH4	2.93	2.79	2.82	2.82	2.87	1.86	2.84	2.81	2.84	2.67	2.52	5.10	2.78	2.79	2.92	2.85	2.79	2.03
540	DAG(14:0/16:1)+NH4	2.79	2.48	2.33	2.53	2.47	6.72	2.61	2.60	2.39	2.38	2.49	4.37	2.75	2.75	2.70	2.49	2.61	4.21

541	DAG(16:0/16:0)+NH4	2.39	2.35	2.31	2.35	2.36	1.12	2.44	2.33	2.54	2.37	2.37	3.47	2.58	2.36	2.28	2.56	2.61	6.01
542	DAG(14:0/18:1)+NH4	2.62	2.64	2.67	2.67	2.72	1.44	2.72	2.62	2.67	2.67	2.68	1.34	2.63	2.71	2.66	2.67	2.70	1.12
543	DAG(16:1/16:1)+NH4	2.53	2.38	2.34	2.34	2.34	3.45	2.72	2.37	2.61	2.37	2.34	6.91	2.34	2.36	2.41	2.45	2.60	4.20
544	DAG(14:0/18:3)+NH4	2.30	2.35	2.50	2.29	2.49	4.23	2.32	2.37	2.41	2.38	2.35	1.40	2.58	2.41	2.58	2.38	2.38	4.17
545	DAG(14:0/20:0)+NH4	2.43	2.43	2.51	2.27	2.40	3.70	2.48	2.37	2.30	2.27	2.43	3.61	2.35	2.42	2.41	2.50	2.38	2.43
546	DAG(16:1/18:0)+NH4	2.30	2.37	2.35	2.39	2.35	1.46	2.70	2.59	2.85	2.63	2.92	5.18	2.78	2.79	2.80	2.85	2.79	0.97
547	DAG(16:0/18:1)+NH4	2.32	2.33	2.36	2.47	2.39	2.46	2.48	2.48	2.52	2.67	2.58	3.13	2.45	2.47	2.50	2.44	2.45	1.01
548	DAG(16:1/18:1)+NH4	2.57	2.63	2.55	2.56	2.57	1.20	2.58	2.55	2.58	2.58	2.59	0.65	2.55	2.54	2.56	2.56	2.54	0.41
549	DAG(16:0/18:2)+NH4	2.35	2.36	2.38	2.38	2.37	0.58	2.26	2.40	2.33	2.43	2.35	2.79	2.35	2.33	2.34	2.56	2.28	4.59
550	DAG(16:0/18:3)+NH4	2.45	2.38	2.38	2.49	2.29	3.15	2.31	2.35	2.36	2.45	2.31	2.47	2.42	2.38	2.40	2.41	2.45	1.09
551	DAG(16:1/18:3)+NH4	2.29	2.27	2.33	2.42	2.75	8.09	2.30	2.37	2.39	2.41	2.25	2.83	2.34	2.51	2.47	2.36	2.32	3.52
552	DAG(14:0/20:4)+NH4	2.37	2.33	2.41	2.91	2.49	9.39	2.42	3.30	2.44	2.26	2.42	16.15	2.50	2.50	2.56	2.66	2.51	2.72
553	DAG(18:0/18:1)+NH4	2.65	2.68	2.65	2.64	2.66	0.62	2.64	2.63	2.67	2.67	2.67	0.71	2.61	2.64	2.59	2.67	2.61	1.11
554	DAG(18:1/18:1)+NH4	2.37	2.37	2.29	2.34	2.36	1.42	2.31	2.30	2.35	2.42	2.38	2.12	2.29	2.35	2.36	2.27	2.35	1.72
555	DAG(18:0/18:2)+NH4	N/A	N/A	N/A	N/A	2.53	#####	N/A	2.66	2.57	2.68	N/A	2.17	2.70	2.79	2.76	2.67	2.92	3.62
556	DAG(18:1/18:2)+NH4	2.30	2.38	2.33	2.33	2.37	1.37	2.38	2.33	2.34	2.32	2.34	1.07	2.30	2.36	2.32	2.35	2.42	1.91
557	DAG(18:0/18:3)+NH4	2.37	2.42	2.28	2.42	2.29	2.87	2.43	2.53	2.41	2.42	2.36	2.57	2.45	2.45	2.40	2.46	2.19	4.85
558	DAG(16:1/20:2)+NH4	2.62	2.49	2.29	2.41	2.80	7.88	2.59	2.38	2.30	2.29	2.33	5.22	2.45	2.36	2.68	2.38	2.37	5.48
559	DAG(16:0/20:3)+NH4	2.47	2.45	2.49	2.40	2.21	4.79	2.35	2.42	2.34	2.38	2.37	1.40	2.27	2.40	2.35	2.41	2.49	3.45
560	DAG(16:0/20:4)+NH4	2.39	2.32	2.26	2.40	2.55	4.50	2.55	2.52	2.30	2.55	2.42	4.35	2.34	2.53	2.34	2.37	2.21	4.93
561	DAG(18:2/18:3)+NH4	2.31	2.34	2.33	2.27	2.20	2.64	2.38	2.37	2.45	2.44	2.49	2.08	2.35	2.25	2.36	2.50	2.41	3.89
562	DAG(16:1/20:4)+NH4	2.19	2.50	2.40	2.33	2.29	4.99	2.26	2.36	2.42	2.30	2.25	3.05	2.43	2.45	2.32	2.46	2.30	3.21
563	DAG(16:0/20:5)+NH4	2.37	2.52	2.29	2.39	2.40	3.43	2.65	2.44	2.41	2.45	2.39	4.23	2.65	2.52	2.31	2.27	2.43	6.27
564	DAG(14:0/22:6)+NH4	2.39	2.33	2.55	2.47	2.53	3.73	2.47	2.55	2.52	2.47	2.58	1.99	2.52	2.38	2.46	2.53	2.39	2.87
565	DAG(18:1/20:1)+NH4	2.31	2.33	2.35	2.32	2.32	0.65	2.33	2.33	2.32	2.29	2.26	1.27	2.35	2.27	2.29	2.38	2.34	2.05
566	DAG(18:1/20:2)+NH4	2.31	2.32	2.55	2.35	2.26	4.64	2.39	2.48	2.42	2.33	2.36	2.58	2.27	2.42	2.24	2.34	2.32	2.96
567	DAG(18:1/20:3)+NH4	2.38	2.38	2.38	2.40	2.53	2.76	2.42	2.52	2.39	2.43	2.31	3.18	2.38	2.49	2.27	2.31	2.53	4.58
568	DAG(18:2/20:3)+NH4	2.46	2.53	2.54	2.43	2.49	1.84	2.34	2.36	2.32	2.40	2.54	3.64	2.37	2.27	2.32	2.42	2.42	2.79
569	DAG(18:1/20:4)+NH4	2.29	2.46	2.34	2.43	2.40	2.95	2.42	2.29	2.38	2.47	2.39	2.73	2.36	2.30	2.55	2.38	2.32	4.14

570	DAG(16:0/22:5)+NH4	2.43	2.46	2.49	2.49	2.43	1.25	2.53	2.46	2.42	2.56	2.54	2.40	2.39	2.39	2.64	2.35	2.35	4.96
571	DAG(18:2/20:4)+NH4	2.34	2.59	2.41	2.47	2.55	4.12	2.29	2.41	2.43	2.49	2.47	3.24	2.39	2.37	2.26	2.30	2.41	2.66
572	DAG(18:1/20:5)+NH4	2.49	2.25	2.37	2.29	2.35	3.97	2.37	2.29	2.29	2.48	2.42	3.60	2.46	2.35	2.37	2.28	2.41	2.89
573	DAG(16:0/22:6)+NH4	2.43	2.38	2.42	2.41	2.33	1.70	2.45	2.47	2.42	2.47	2.37	1.65	2.30	2.36	2.46	2.36	2.28	2.93
574	DAG(18:2/20:5)+NH4	2.30	2.45	2.31	2.41	2.26	3.47	2.35	2.60	2.41	2.45	2.60	4.46	2.35	2.26	2.40	2.25	2.44	3.67
575	DAG(16:1/22:6)+NH4	2.68	2.11	2.73	2.37	2.64	10.50	2.50	2.38	2.75	2.45	2.29	6.95	2.50	2.47	2.50	2.32	2.27	4.50
576	DAG(20:0/20:0)+NH4	2.33	2.35	2.34	2.39	2.35	1.04	2.33	2.35	2.37	2.36	2.34	0.76	2.35	2.35	2.30	2.34	2.34	0.74
577	DAG(18:1/22:4)+NH4	2.35	2.31	2.33	2.41	2.37	1.61	2.38	2.36	2.33	2.40	2.40	1.39	2.31	2.36	2.43	2.35	2.37	1.90
578	DAG(18:2/22:4)+NH4	2.51	2.43	2.20	2.82	2.29	9.68	2.30	2.54	2.48	2.57	2.45	4.27	2.33	2.44	2.19	2.52	2.61	6.77
579	DAG(18:1/22:5)+NH4	2.36	2.51	2.72	2.47	2.46	5.33	2.30	2.53	2.51	2.45	2.56	4.23	2.38	2.38	2.30	2.42	2.32	2.06
580	DAG(18:2/22:5)+NH4	2.48	2.43	2.50	2.42	2.43	1.50	2.35	2.51	2.41	2.36	2.53	3.48	2.26	2.32	2.34	2.37	2.31	1.76
581	DAG(18:1/22:6)+NH4	2.36	2.65	2.41	2.44	2.34	5.14	2.29	2.37	2.33	2.32	2.35	1.34	2.38	2.40	2.32	2.37	2.32	1.50
582	DAG(18:2/22:6)+NH4	2.39	2.51	2.35	2.21	2.38	4.53	2.48	2.48	2.30	2.45	2.37	3.24	2.37	2.34	2.52	2.38	2.45	2.99
583	MAG(20:1)+NH4	2.39	2.41	2.49	2.49	2.43	1.88	2.62	2.58	2.59	2.60	2.64	0.88	2.43	2.45	2.44	2.35	2.40	1.62
584	MAG(20:3)+NH4	2.72	2.48	2.49	2.44	2.40	4.95	2.63	2.49	2.44	2.42	2.49	3.31	2.45	2.43	2.44	2.40	2.42	0.84
585	MAG(22:1)+NH4	2.47	2.58	2.43	2.50	2.52	2.37	2.55	2.52	2.53	2.85	2.57	5.39	2.50	2.63	2.46	2.60	2.45	3.30
586	MAG(22:3)+NH4	2.44	2.46	2.50	2.47	2.41	1.35	2.43	2.32	2.45	2.52	2.47	3.03	2.59	2.40	2.45	2.42	2.41	3.20
587	MAG(22:5)+NH4	2.34	2.48	2.37	2.53	2.28	4.28	2.49	2.35	2.48	2.42	2.53	2.93	2.65	2.67	2.42	2.52	2.82	5.88
588	LPC(14:0)+AcO	13.09	12.99	12.99	13.01	12.99	0.33	12.92	12.91	12.90	12.91	12.87	0.15	12.82	12.81	12.84	12.82	12.81	0.09
589	LPC(16:0)+AcO	12.87	12.86	12.86	12.89	12.86	0.10	12.73	12.77	12.70	12.76	12.73	0.21	12.63	12.64	12.64	12.65	12.61	0.14
590	LPC(16:1)+AcO	12.97	12.90	12.93	12.94	12.92	0.21	12.80	12.80	12.78	12.77	12.74	0.18	12.69	12.68	12.69	12.69	12.66	0.12
591	LPC(18:0)+AcO	12.72	12.69	12.69	12.70	12.70	0.10	12.58	12.57	12.54	12.58	12.56	0.13	12.45	12.46	12.45	12.47	12.43	0.13
592	LPC(18:1)+AcO	12.74	12.72	12.74	12.75	12.75	0.09	12.59	12.63	12.59	12.62	12.60	0.17	12.47	12.52	12.49	12.51	12.46	0.20
593	LPC(18:2)+AcO	12.84	12.81	12.83	12.82	12.83	0.08	12.67	12.69	12.66	12.66	12.66	0.09	12.54	12.60	12.59	12.56	12.49	0.36
594	LPC(18:3)+AcO	12.87	12.93	12.94	12.88	12.91	0.21	12.77	12.75	12.73	12.76	12.75	0.10	12.66	12.70	12.66	12.65	12.61	0.23
595	LPC(20:0)+AcO	12.59	12.60	12.62	12.57	12.60	0.15	12.46	12.44	12.42	12.45	12.45	0.11	12.34	12.33	12.34	12.37	12.33	0.12
596	LPC(20:1)+AcO	12.64	12.61	12.62	12.59	12.59	0.17	12.48	12.48	12.47	12.50	12.46	0.12	12.34	12.39	12.38	12.37	12.35	0.18
597	LPC(20:2)+AcO	12.71	12.67	12.66	12.64	12.69	0.23	12.50	12.49	12.55	12.49	12.49	0.21	12.43	12.46	12.43	12.41	12.40	0.19
598	LPC(20:3)+AcO	12.69	12.67	12.71	12.69	12.71	0.14	12.56	12.59	12.54	12.55	12.55	0.15	12.43	12.44	12.45	12.46	12.43	0.09

599	LPC(20:4)+AcO	12.67	12.64	12.68	12.65	12.67	0.12	12.57	12.51	12.51	12.51	12.51	0.21	12.40	12.44	12.42	12.43	12.37	0.23
600	LPC(20:5)+AcO	12.89	12.81	12.83	12.82	12.75	0.41	12.62	12.53	12.78	12.50	12.57	0.89	12.53	12.58	12.52	12.39	12.54	0.58
601	LPC(22:4)+AcO	12.56	12.55	12.57	12.60	12.62	0.23	12.37	12.50	12.41	12.47	12.45	0.41	12.30	12.37	12.35	12.28	12.32	0.31
602	LPC(22:5)+AcO	12.65	12.62	12.64	12.62	12.63	0.09	12.51	12.46	12.44	12.49	12.50	0.23	12.37	12.37	12.35	12.39	12.34	0.15
603	LPC(22:6)+AcO	12.68	12.57	12.59	12.59	12.58	0.37	12.39	12.49	12.41	12.52	12.51	0.48	12.36	12.34	12.49	12.42	12.30	0.59
604	PC(14:0/14:0)+AcO	11.01	11.03	10.96	10.82	10.82	0.92	10.65	10.75	10.76	10.81	10.81	0.59	10.19	10.42	10.49	10.40	10.29	1.13
605	PC(14:0/18:1)+AcO	10.65	10.71	10.68	10.64	10.62	0.33	10.40	10.43	10.43	10.37	10.46	0.34	10.01	9.93	9.98	9.92	9.94	0.39
606	PC(14:0/18:2)+AcO	10.75	10.74	10.69	10.66	10.69	0.37	10.45	10.54	10.44	10.42	10.56	0.60	10.08	10.03	10.00	9.99	10.02	0.36
607	PC(14:0/18:3)+AcO	10.70	10.89	10.99	10.85	10.82	0.97	10.68	10.81	10.80	10.76	10.85	0.60	10.44	10.19	10.39	10.34	10.28	0.92
608	PC(14:0/20:1)+AcO	10.50	10.40	10.85	10.31	10.33	2.12	10.70	10.75	10.87	10.74	10.87	0.74	10.31	10.31	10.34	10.33	10.34	0.18
609	PC(14:0/20:2)+AcO	10.68	10.60	10.63	10.66	10.51	0.62	10.58	10.57	10.51	10.65	10.77	0.93	10.30	10.35	9.83	10.20	10.06	2.05
610	PC(14:0/20:3)+AcO	10.68	10.57	10.71	10.64	10.57	0.58	10.30	10.41	10.46	10.32	10.45	0.74	9.94	9.84	9.81	9.89	9.92	0.56
611	PC(14:0/20:4)+AcO	10.30	10.38	10.42	10.36	10.35	0.43	10.04	9.99	10.03	9.96	10.00	0.29	9.54	9.57	9.62	9.56	9.61	0.35
612	PC(14:0/20:5)+AcO	10.59	10.29	10.67	10.32	10.41	1.61	10.61	10.87	10.51	10.38	10.65	1.69	9.42	9.84	9.48	9.49	9.72	1.89
613	PC(14:0/22:5)+AcO	10.49	10.54	10.52	10.37	10.43	0.64	11.43	10.21	10.59	11.15	10.98	4.42	10.60	10.73	10.93	10.89	10.61	1.44
614	PC(14:1/14:1)+AcO	10.84	10.40	10.33	10.56	10.73	2.04	10.45	10.30	10.20	10.53	11.00	2.94	9.80	9.78	9.52	9.93	9.61	1.67
615	PC(16:0/14:0)+AcO	10.82	10.90	10.86	10.85	10.87	0.25	10.63	10.64	10.68	10.61	10.64	0.21	10.28	10.29	10.24	10.19	10.21	0.40
616	PC(16:0/16:0)+AcO	10.69	10.68	10.65	10.68	10.68	0.15	10.46	10.48	10.50	10.46	10.52	0.24	10.11	10.05	10.06	10.04	10.06	0.28
617	PC(16:0/16:1)+AcO	10.76	10.78	10.76	10.74	10.72	0.22	10.41	10.46	10.46	10.41	10.45	0.25	10.09	9.98	10.00	9.95	10.00	0.52
618	PC(16:0/18:0)+AcO	10.42	10.43	10.41	10.40	10.42	0.11	10.16	10.21	10.27	10.16	10.25	0.47	9.82	9.76	9.77	9.76	9.78	0.25
619	PC(16:0/18:1)+AcO	10.60	10.56	10.56	10.59	10.53	0.24	10.20	10.24	10.27	10.22	10.28	0.34	9.82	9.76	9.80	9.75	9.77	0.31
620	PC(16:0/18:2)+AcO	10.68	10.60	10.60	10.62	10.59	0.32	10.22	10.28	10.31	10.25	10.34	0.43	9.86	9.80	9.82	9.80	9.78	0.31
621	PC(16:0/18:3)+AcO	10.68	10.64	10.64	10.65	10.60	0.25	10.29	10.33	10.36	10.27	10.35	0.39	9.89	9.90	9.86	9.88	9.83	0.28
622	PC(16:0/20:1)+AcO	10.52	10.45	10.47	10.47	10.48	0.26	10.15	10.18	10.22	10.13	10.21	0.38	9.72	9.66	9.72	9.62	9.66	0.45
623	PC(16:0/20:2)+AcO	10.50	10.49	10.50	10.47	10.46	0.18	10.12	10.16	10.18	10.13	10.18	0.29	9.67	9.66	9.70	9.62	9.63	0.31
624	PC(16:0/20:3)+AcO	10.38	10.39	10.37	10.38	10.36	0.10	9.96	9.98	10.04	9.95	10.02	0.37	9.51	9.48	9.53	9.42	9.51	0.44
625	PC(16:0/20:4)+AcO	10.20	10.21	10.15	10.16	10.13	0.32	9.76	9.78	9.79	9.74	9.81	0.30	9.31	9.28	9.30	9.25	9.27	0.26
626	PC(16:0/20:5)+AcO	10.27	10.21	10.23	10.23	10.18	0.32	9.82	9.85	9.81	9.77	9.85	0.35	9.40	9.37	9.35	9.28	9.34	0.49
627	PC(16:0/22:4)+AcO	10.19	10.20	10.12	10.14	10.10	0.44	9.76	9.77	9.79	9.71	9.78	0.33	9.28	9.28	9.30	9.26	9.26	0.21

628	PC(16:0/22:5)+AcO	10.19	10.21	10.15	10.12	10.13	0.37	9.78	9.79	9.81	9.75	9.84	0.33	9.33	9.27	9.29	9.24	9.28	0.35
629	PC(16:1/18:1)+AcO	10.52	10.47	10.48	10.47	10.46	0.22	10.14	10.18	10.19	10.18	10.23	0.31	9.71	9.70	9.72	9.62	9.65	0.45
630	PC(16:1/18:2)+AcO	10.58	10.54	10.62	10.63	10.63	0.35	10.10	10.29	10.25	10.32	10.32	0.91	9.78	9.82	9.80	9.82	9.68	0.58
631	PC(16:0/22:6)+AcO	10.13	10.11	10.05	10.04	10.07	0.39	9.71	9.72	9.76	9.67	9.74	0.34	9.25	9.16	9.22	9.12	9.18	0.55
632	PC(18:0/14:0)+AcO	10.81	10.79	10.74	10.72	10.72	0.39	10.50	10.58	10.53	10.34	10.42	0.91	10.11	10.05	10.02	9.99	10.11	0.55
633	PC(18:0/16:1)+AcO	10.58	10.60	10.62	10.56	10.63	0.25	10.18	10.26	10.25	10.23	10.31	0.46	9.78	9.79	9.79	9.77	9.77	0.10
634	PC(18:0/18:0)+AcO	10.37	10.36	10.34	10.33	10.36	0.18	10.07	10.12	10.15	10.08	10.12	0.31	9.68	9.68	9.67	9.60	9.65	0.36
635	PC(18:0/18:1)+AcO	10.42	10.42	10.40	10.39	10.39	0.14	9.99	10.05	10.06	9.99	10.06	0.36	9.57	9.52	9.58	9.42	9.54	0.66
636	PC(18:0/18:2)+AcO	10.41	10.39	10.41	10.39	10.40	0.08	10.00	10.05	10.09	10.02	10.01	0.36	9.59	9.56	9.59	9.49	9.55	0.42
637	PC(18:0/18:3)+AcO	10.49	10.41	10.42	10.47	10.45	0.31	10.01	10.15	10.07	10.01	10.17	0.75	9.57	9.58	9.57	9.50	9.58	0.36
638	PC(18:0/20:0)+AcO	N/A	N/A	N/A	N/A	N/A	#####	10.66	10.67	10.72	10.60	10.67	0.43	10.28	10.22	10.23	10.24	10.23	0.23
639	PC(18:0/20:1)+AcO	10.36	10.40	10.22	10.35	10.38	0.69	9.93	10.01	10.04	10.00	9.92	0.54	9.48	9.52	9.50	9.39	9.41	0.62
640	PC(18:0/20:2)+AcO	10.29	10.31	10.29	10.30	10.28	0.08	9.88	9.93	9.96	9.91	9.88	0.35	9.47	9.42	9.48	9.29	9.40	0.83
641	PC(18:0/20:3)+AcO	10.20	10.20	10.19	10.15	10.17	0.22	9.80	9.80	9.85	9.76	9.81	0.32	9.32	9.31	9.31	9.26	9.28	0.24
642	PC(18:0/20:4)+AcO	10.06	10.06	10.02	10.01	10.02	0.25	9.64	9.69	9.69	9.61	9.71	0.43	9.22	9.16	9.18	9.12	9.16	0.39
643	PC(18:0/20:5)+AcO	10.08	10.09	10.02	10.06	10.02	0.33	9.68	9.71	9.73	9.64	9.74	0.44	9.23	9.15	9.21	9.13	9.15	0.48
644	PC(18:0/22:4)+AcO	10.07	10.11	10.01	10.04	10.07	0.39	9.71	9.71	9.76	9.63	9.70	0.48	9.21	9.15	9.20	9.14	9.17	0.33
645	PC(18:0/22:5)+AcO	10.08	10.10	10.04	10.02	10.01	0.36	9.68	9.69	9.73	9.63	9.70	0.37	9.21	9.17	9.16	9.13	9.15	0.32
646	PC(18:0/22:6)+AcO	10.01	10.01	9.97	9.98	9.99	0.21	9.60	9.64	9.68	9.56	9.64	0.49	9.14	9.09	9.09	9.04	9.07	0.40
647	PC(18:1/16:1)+AcO	10.54	10.53	10.49	10.45	10.50	0.32	10.11	10.14	10.22	10.19	10.19	0.43	9.72	9.67	9.76	9.69	9.70	0.35
648	PC(18:1/18:1)+AcO	10.32	10.35	10.31	10.27	10.33	0.29	9.91	9.97	10.01	9.90	9.97	0.46	9.45	9.45	9.49	9.37	9.46	0.49
649	PC(18:1/18:2)+AcO	10.38	10.39	10.38	10.37	10.35	0.17	9.96	10.00	10.08	10.00	10.00	0.41	9.53	9.50	9.53	9.45	9.53	0.35
650	PC(18:1/18:3)+AcO	10.50	10.37	10.50	10.37	10.52	0.69	10.07	10.04	10.22	10.08	10.03	0.76	9.63	9.49	9.56	9.60	9.69	0.77
651	PC(18:1/20:1)+AcO	10.27	10.20	10.22	10.21	10.25	0.27	9.87	9.91	9.84	9.90	9.87	0.28	9.35	9.30	9.42	9.31	9.38	0.52
652	PC(18:1/20:2)+AcO	10.32	10.28	10.28	10.22	10.15	0.65	9.90	9.94	9.90	9.81	9.84	0.52	9.44	9.40	9.46	9.31	9.41	0.60
653	PC(18:1/20:3)+AcO	10.19	10.23	10.14	10.11	10.14	0.49	9.81	9.78	9.81	9.75	9.84	0.37	9.28	9.28	9.27	9.21	9.23	0.34
654	PC(18:1/20:4)+AcO	10.05	10.04	9.99	10.03	9.97	0.34	9.62	9.70	9.68	9.61	9.70	0.47	9.23	9.14	9.17	9.10	9.12	0.54
655	PC(18:1/20:5)+AcO	10.05	10.08	10.08	10.07	9.91	0.70	9.72	9.79	9.73	9.58	9.72	0.77	9.23	9.13	9.23	9.18	9.17	0.48
656	PC(18:1/22:4)+AcO	10.20	10.06	10.05	10.05	9.98	0.80	9.57	9.59	9.68	9.65	9.58	0.50	9.24	9.18	9.16	9.13	9.17	0.44

657	PC(18:1/22:5)+AcO	10.05	10.07	10.01	10.00	10.05	0.30	9.66	9.74	9.78	9.59	9.72	0.77	9.13	9.17	9.17	9.12	9.14	0.26
658	PC(18:1/22:6)+AcO	10.02	10.00	9.87	9.98	9.99	0.57	9.56	9.51	9.65	9.52	9.69	0.81	9.16	9.11	9.07	9.03	9.07	0.52
659	PC(18:2/16:1)+AcO	10.59	10.53	10.56	10.54	10.51	0.26	10.14	10.27	10.23	10.24	10.28	0.52	9.78	9.80	9.77	9.77	9.74	0.20
660	PC(18:2/18:2)+AcO	10.41	10.41	10.44	10.43	10.42	0.10	10.00	10.10	10.10	10.00	10.12	0.58	9.58	9.62	9.61	9.52	9.57	0.41
661	PC(18:2/18:3)+AcO	10.53	10.48	10.61	10.46	10.46	0.60	10.27	10.26	10.20	10.22	10.18	0.38	9.79	9.66	9.82	9.73	9.63	0.83
662	PC(18:2/20:1)+AcO	10.26	10.29	10.23	10.22	10.11	0.68	9.78	9.80	9.89	9.88	9.85	0.48	9.39	9.36	9.40	9.28	9.30	0.59
663	PC(18:2/20:2)+AcO	10.27	10.31	10.31	10.35	10.28	0.30	9.89	9.86	9.99	9.88	9.97	0.59	9.46	9.30	9.41	9.36	9.37	0.63
664	PC(18:2/20:3)+AcO	10.22	10.29	10.18	10.16	10.18	0.52	9.81	9.83	9.80	9.82	9.86	0.23	9.31	9.26	9.32	9.25	9.30	0.32
665	PC(18:2/20:4)+AcO	10.11	10.09	10.05	10.05	10.05	0.29	9.74	9.73	9.69	9.63	9.72	0.46	9.21	9.12	9.16	9.12	9.14	0.40
666	PC(18:2/20:5)+AcO	10.11	10.08	10.25	10.33	10.24	1.01	9.69	10.06	9.72	9.59	9.86	1.88	9.26	9.14	9.24	9.26	9.14	0.67
667	PC(18:2/22:5)+AcO	10.03	10.06	9.98	10.25	10.02	1.07	9.72	9.66	9.72	9.63	9.75	0.51	9.32	9.18	9.21	9.15	9.13	0.83
668	PC(18:2/22:6)+AcO	10.01	9.96	9.93	10.11	10.14	0.90	9.51	9.65	9.70	9.73	9.65	0.87	9.23	9.37	9.28	9.04	9.04	1.61
669	PC(20:0/16:1)+AcO	N/A	N/A	N/A	N/A	N/A	#####	10.67	10.80	10.60	10.74	10.93	1.17	10.34	N/A	N/A	N/A	N/A	#####
670	PC(20:0/18:1)+AcO	10.30	10.40	10.34	11.03	10.47	2.84	10.68	10.66	10.64	10.61	10.68	0.28	11.33	10.96	11.17	11.06	11.10	1.24
671	PC(20:0/18:3)+AcO	N/A	N/A	N/A	N/A	N/A	#####	10.55	10.88	10.98	10.77	10.64	1.61	11.60	N/A	N/A	N/A	11.58	0.12
672	PC(20:0/20:1)+AcO	10.09	10.96	10.43	10.31	10.65	3.16	10.65	10.60	10.49	10.52	10.48	0.72	10.04	10.18	10.18	10.19	11.00	3.73
673	PC(20:0/20:2)+AcO	10.16	10.37	10.06	10.03	10.11	1.34	10.69	10.19	10.44	10.51	11.18	3.51	N/A	10.07	N/A	N/A	N/A	#####
674	PC(20:0/20:3)+AcO	10.26	10.21	10.10	10.08	10.06	0.86	9.74	9.71	9.77	9.70	9.76	0.32	10.42	10.34	10.24	9.96	N/A	1.96
675	PC(20:0/20:4)+AcO	10.12	10.02	10.01	10.02	9.94	0.63	9.73	9.70	9.64	9.58	9.68	0.61	10.00	10.38	N/A	10.21	10.29	1.58
676	PC(20:0/20:5)+AcO	10.16	9.91	10.16	9.99	10.04	1.07	9.59	9.59	9.68	9.56	9.97	1.77	N/A	10.02	10.53	10.64	10.02	3.19
677	PC(20:0/22:5)+AcO	10.00	10.12	10.09	9.93	10.05	0.75	10.24	11.01	9.30	9.56	9.79	6.71	11.40	10.94	10.96	11.06	N/A	1.92
678	PC(20:0/22:6)+AcO	10.21	9.93	10.18	10.24	10.52	2.04	9.46	9.75	9.81	9.32	9.63	2.13	N/A	10.23	10.49	10.21	N/A	1.50
679	LPE(14:0)-H	13.09	12.97	12.12	12.79	13.08	3.18	13.16	13.10	13.10	13.18	13.13	0.26	12.93	13.14	13.13	12.92	12.81	1.11
680	LPE(16:0)-H	13.12	13.07	13.09	13.11	13.08	0.14	13.13	13.01	13.02	13.01	13.08	0.41	12.94	12.95	13.07	12.94	12.93	0.44
681	LPE(16:1)-H	13.12	12.97	12.93	13.06	13.14	0.69	13.13	13.05	13.04	13.06	13.09	0.28	12.92	13.03	13.13	12.98	12.98	0.61
682	LPE(18:0)-H	13.06	13.00	13.00	13.04	12.98	0.26	12.95	12.90	12.87	12.89	12.90	0.24	12.79	12.81	12.80	12.80	12.78	0.09
683	LPE(18:1)-H	13.07	13.01	13.03	13.03	13.02	0.16	13.00	12.92	12.91	12.92	12.91	0.28	12.82	12.85	12.86	12.82	12.79	0.23
684	LPE(18:2)-H	13.10	13.05	13.07	13.07	13.06	0.12	13.09	12.98	12.97	12.97	13.02	0.39	12.88	12.90	12.95	12.90	12.85	0.28
685	LPE(18:3)-H	13.12	13.07	13.09	13.09	13.09	0.13	13.12	13.00	12.98	13.08	13.13	0.51	12.94	12.94	13.02	12.96	12.97	0.24

686	LPE(20:0)-H	12.97	13.06	12.94	12.97	12.94	0.40	13.13	12.80	12.77	12.87	12.98	1.12	12.69	12.71	13.13	12.70	12.70	1.50
687	LPE(20:1)-H	12.96	12.96	12.94	12.94	12.99	0.14	12.83	12.82	12.80	12.85	12.76	0.26	12.72	12.74	12.72	12.73	12.70	0.12
688	LPE(20:2)-H	13.03	12.96	12.94	13.01	13.08	0.42	12.92	12.86	12.88	12.81	12.85	0.31	12.71	12.78	12.80	12.83	12.73	0.42
689	LPE(20:3)-H	13.03	13.00	13.00	13.03	13.01	0.11	12.90	12.89	12.88	12.88	12.82	0.22	12.77	12.81	12.83	12.81	12.79	0.17
690	LPE(20:4)-H	13.01	12.98	13.00	12.99	12.99	0.09	12.86	12.88	12.85	12.88	12.86	0.12	12.76	12.80	12.76	12.77	12.74	0.17
691	LPE(20:5)-H	12.99	12.97	12.98	12.99	13.05	0.25	12.85	12.93	12.85	12.94	12.97	0.42	12.82	12.86	12.84	12.83	12.84	0.11
692	LPE(22:4)-H	12.97	12.92	12.95	12.94	12.97	0.18	12.76	12.79	12.78	12.80	12.78	0.10	12.68	12.66	12.67	12.71	12.71	0.17
693	LPE(22:5)-H	12.99	12.97	12.93	13.04	12.96	0.32	12.82	12.86	12.79	12.80	12.80	0.21	12.72	12.76	12.76	12.77	12.73	0.17
694	LPE(22:6)-H	12.95	12.95	12.91	13.03	12.90	0.40	12.92	12.87	12.80	12.87	12.79	0.41	12.76	12.77	12.79	12.76	12.72	0.19
695	PE(14:0/14:0)-H	10.96	N/A	10.94	11.63	11.05	2.92	11.01	11.04	10.93	10.92	11.10	0.69	10.59	10.69	10.99	10.86	10.76	1.43
696	PE(14:0/16:1)-H	11.05	10.82	10.97	11.01	11.21	1.28	10.92	10.93	10.94	11.15	11.15	1.09	10.67	10.60	10.59	10.59	10.68	0.45
697	PE(14:0/18:1)-H	10.82	10.93	10.81	10.81	10.79	0.53	10.74	10.81	10.81	10.85	10.87	0.46	11.48	11.55	N/A	11.28	11.58	1.18
698	PE(14:0/18:2)-H	10.82	10.72	10.84	10.74	10.80	0.50	10.71	10.69	10.67	10.69	10.65	0.21	11.33	N/A	10.93	N/A	N/A	2.57
699	PE(14:0/20:2)-H	10.55	10.82	10.58	10.94	10.62	1.57	10.84	10.94	10.32	10.93	11.06	2.68	10.50	10.88	10.46	10.27	10.45	2.12
700	PE(14:0/20:3)-H	10.13	10.51	10.55	10.54	10.69	2.00	9.95	9.95	10.05	10.07	10.41	1.87	N/A	10.48	N/A	10.64	N/A	1.03
701	PE(14:0/20:4)-H	10.59	10.60	10.49	10.66	10.64	0.60	10.63	10.44	10.48	10.42	10.39	0.90	10.64	11.02	N/A	11.06	10.85	1.74
702	PE(14:0/22:5)-H	10.49	10.50	10.50	10.62	10.51	0.52	10.26	10.57	10.41	10.65	10.58	1.48	10.46	10.38	N/A	N/A	11.19	4.17
703	PE(14:0/22:6)-H	10.38	10.66	10.47	10.49	10.56	1.00	10.26	10.37	10.47	10.14	10.32	1.18	N/A	N/A	N/A	10.46	11.19	4.76
704	PE(16:0/14:0)-H	10.94	10.97	11.00	11.02	11.04	0.36	11.45	11.53	11.61	11.39	11.48	0.74	11.09	11.10	11.11	11.40	11.30	1.26
705	PE(16:0/16:0)-H	10.90	10.88	10.87	10.90	10.91	0.13	10.73	10.76	10.77	10.74	10.78	0.18	10.38	10.34	10.39	10.32	10.32	0.32
706	PE(16:0/16:1)-H	10.91	10.88	10.89	10.85	10.87	0.20	10.79	10.80	10.83	10.73	10.83	0.38	10.43	10.44	10.43	10.35	10.35	0.44
707	PE(16:0/18:1)-H	10.76	10.77	10.77	10.74	10.75	0.14	10.68	10.71	10.71	10.65	10.70	0.22	10.29	10.25	10.26	10.22	10.23	0.25
708	PE(16:0/18:2)-H	10.77	10.78	10.77	10.77	10.76	0.08	10.70	10.70	10.70	10.67	10.73	0.18	10.28	10.26	10.26	10.20	10.25	0.27
709	PE(16:0/18:3)-H	10.82	10.78	10.76	10.77	10.77	0.21	10.67	10.71	10.70	10.69	10.76	0.33	10.27	10.27	10.29	10.26	10.25	0.14
710	PE(16:0/20:1)-H	10.72	10.73	10.74	10.75	10.77	0.19	10.63	10.69	10.64	10.61	10.67	0.33	10.23	10.20	10.23	10.17	10.18	0.29
711	PE(16:0/20:2)-H	10.74	10.75	10.72	10.72	10.77	0.21	10.65	10.65	10.66	10.55	10.58	0.47	10.23	10.18	10.15	10.15	10.17	0.33
712	PE(16:0/20:3)-H	10.69	10.69	10.68	10.69	10.66	0.11	10.51	10.59	10.59	10.45	10.56	0.55	10.13	10.07	10.07	9.99	10.07	0.48
713	PE(16:0/20:4)-H	10.62	10.60	10.58	10.58	10.57	0.18	10.38	10.41	10.43	10.39	10.42	0.22	9.94	9.88	9.89	9.84	9.86	0.38
714	PE(16:0/20:5)-H	10.64	10.60	10.63	10.60	10.63	0.16	10.42	10.43	10.52	10.44	10.52	0.47	10.00	9.94	9.94	9.87	9.95	0.48

715	PE(16:0/22:4)-H	10.61	10.59	10.58	10.55	10.59	0.19	10.37	10.43	10.44	10.35	10.42	0.37	9.92	9.85	9.91	9.83	9.79	0.56
716	PE(16:0/22:5)-H	10.62	10.62	10.60	10.57	10.58	0.21	10.39	10.41	10.43	10.37	10.45	0.32	9.94	9.90	9.88	9.87	9.87	0.30
717	PE(16:0/22:6)-H	10.58	10.56	10.53	10.53	10.50	0.27	10.29	10.30	10.33	10.29	10.36	0.29	9.83	9.81	9.82	9.79	9.79	0.21
718	PE(18:0/14:0)-H	11.81	10.96	11.34	12.01	11.16	3.86	11.61	11.55	11.45	11.42	11.53	0.67	11.44	11.24	11.65	11.23	11.15	1.81
719	PE(18:0/16:0)-H	10.77	10.83	10.79	10.77	10.78	0.24	10.76	10.72	10.72	10.73	10.80	0.31	11.55	11.60	11.61	11.62	11.61	0.22
720	PE(18:0/16:1)-H	10.77	10.84	10.72	10.71	10.72	0.49	11.19	11.10	11.30	11.16	10.99	1.01	11.41	11.43	11.46	11.52	11.42	0.40
721	PE(18:0/18:0)-H	10.52	10.55	10.53	10.52	10.53	0.12	10.25	10.29	10.29	10.26	10.32	0.30	9.84	9.81	9.89	9.84	9.87	0.34
722	PE(18:0/18:1)-H	10.62	10.62	10.61	10.61	10.64	0.11	10.22	10.28	10.29	10.24	10.31	0.35	9.91	9.82	9.88	9.79	9.82	0.48
723	PE(18:0/18:2)-H	10.67	10.67	10.67	10.64	10.63	0.17	10.29	10.35	10.33	10.31	10.41	0.46	9.95	9.89	9.93	9.89	9.89	0.27
724	PE(18:0/18:3)-H	10.70	10.68	10.69	10.69	10.70	0.06	10.37	10.46	10.45	10.38	10.52	0.60	10.02	9.98	10.05	10.01	9.98	0.29
725	PE(18:0/20:1)-H	10.58	10.60	10.60	10.55	10.62	0.27	10.32	10.40	10.44	10.50	10.48	0.70	10.04	10.05	10.03	9.99	9.95	0.41
726	PE(18:0/20:2)-H	10.52	10.51	10.52	10.47	10.52	0.19	10.29	10.42	10.38	10.39	10.43	0.51	9.85	9.88	9.88	9.79	9.81	0.42
727	PE(18:0/20:3)-H	10.45	10.47	10.43	10.46	10.43	0.18	10.06	10.11	10.14	10.07	10.14	0.37	9.63	9.62	9.66	9.58	9.60	0.31
728	PE(18:0/20:4)-H	10.45	10.45	10.43	10.43	10.41	0.15	10.03	10.06	10.14	10.01	10.15	0.61	9.61	9.59	9.63	9.54	9.57	0.38
729	PE(18:0/20:5)-H	10.43	10.47	10.44	10.43	10.43	0.18	10.15	10.06	10.14	10.06	10.11	0.41	9.68	9.62	9.65	9.51	9.63	0.67
730	PE(18:0/22:4)-H	10.44	10.43	10.42	10.42	10.40	0.13	10.07	10.03	10.16	10.08	10.15	0.55	9.62	9.58	9.64	9.49	9.59	0.58
731	PE(18:0/22:5)-H	10.42	10.43	10.38	10.41	10.37	0.26	10.03	10.04	10.11	10.05	10.10	0.38	9.60	9.60	9.60	9.51	9.50	0.53
732	PE(18:0/22:6)-H	10.38	10.34	10.34	10.34	10.33	0.19	9.92	10.01	10.07	9.97	10.02	0.58	9.57	9.50	9.53	9.47	9.44	0.55
733	PE(18:1/16:1)-H	10.63	10.63	10.72	10.66	10.61	0.41	10.51	10.65	10.62	10.63	10.63	0.54	10.26	10.26	10.24	10.24	10.23	0.13
734	PE(18:1/18:1)-H	10.65	10.67	10.65	10.64	10.66	0.07	10.51	10.51	10.52	10.47	10.52	0.20	10.08	10.03	10.06	10.02	10.04	0.25
735	PE(18:1/18:2)-H	10.66	10.68	10.67	10.66	10.66	0.09	10.51	10.54	10.56	10.50	10.57	0.29	10.08	10.05	10.09	10.03	10.08	0.28
736	PE(18:1/18:3)-H	10.72	10.75	10.71	10.69	10.65	0.34	10.59	10.64	10.66	10.51	10.57	0.54	10.14	10.08	10.09	10.06	10.16	0.40
737	PE(18:1/20:1)-H	10.67	10.65	10.61	10.60	10.59	0.33	10.45	10.44	10.53	10.47	10.57	0.52	10.00	10.00	10.04	10.01	10.00	0.15
738	PE(18:1/20:2)-H	10.65	10.65	10.65	10.61	10.58	0.30	10.36	10.49	10.52	10.50	10.49	0.61	10.01	9.98	9.97	9.91	10.05	0.51
739	PE(18:1/20:3)-H	10.60	10.59	10.57	10.55	10.56	0.19	10.34	10.38	10.41	10.33	10.42	0.39	9.90	9.85	9.92	9.85	9.87	0.34
740	PE(18:1/20:4)-H	10.47	10.48	10.45	10.45	10.40	0.29	10.19	10.25	10.27	10.20	10.29	0.40	9.74	9.67	9.69	9.66	9.68	0.33
741	PE(18:1/20:5)-H	10.52	10.53	10.55	10.48	10.47	0.30	10.28	10.37	10.49	10.24	10.49	1.12	9.75	9.69	9.66	9.82	9.76	0.64
742	PE(18:1/22:4)-H	10.34	10.51	10.41	10.42	10.46	0.60	10.21	10.31	10.26	10.14	10.23	0.62	9.79	9.62	9.68	9.65	9.74	0.68
743	PE(18:1/22:5)-H	10.46	10.45	10.45	10.45	10.42	0.16	10.23	10.28	10.32	10.20	10.28	0.46	9.72	9.67	9.72	9.69	9.68	0.25

744	PE(18:1/22:6)-H	10.38	10.43	10.37	10.36	10.33	0.35	10.13	10.21	10.25	10.10	10.19	0.60	9.66	9.58	9.64	9.55	9.52	0.58
745	PE(18:2/16:1)-H	10.59	10.60	10.56	10.56	10.55	0.23	10.32	10.39	10.39	10.38	10.38	0.31	9.87	9.84	9.92	9.73	9.82	0.73
746	PE(18:2/18:2)-H	10.68	10.70	10.67	10.68	10.67	0.11	10.55	10.56	10.62	10.54	10.63	0.39	10.15	10.10	10.13	10.10	10.12	0.21
747	PE(18:2/18:3)-H	10.68	10.73	10.73	10.68	10.73	0.27	10.60	10.56	10.69	10.55	10.70	0.68	10.19	10.28	10.26	10.06	10.23	0.86
748	PE(18:2/20:1)-H	10.58	10.64	10.62	10.60	10.60	0.23	10.53	10.49	10.45	10.44	10.48	0.34	10.02	9.96	9.95	9.92	10.01	0.42
749	PE(18:2/20:2)-H	10.63	10.68	10.64	10.60	10.64	0.27	10.51	10.49	10.71	10.50	10.50	0.89	9.92	10.10	9.99	9.92	9.95	0.74
750	PE(18:2/20:3)-H	10.64	10.62	10.63	10.63	10.58	0.23	10.32	10.37	10.43	10.45	10.48	0.64	9.91	9.94	9.96	9.85	9.84	0.54
751	PE(18:2/20:4)-H	10.53	10.52	10.53	10.46	10.52	0.29	10.26	10.29	10.35	10.28	10.34	0.40	9.79	9.73	9.78	9.71	9.72	0.38
752	PE(18:2/20:5)-H	10.51	10.54	10.72	10.59	10.68	0.85	10.35	10.57	10.46	10.24	10.37	1.19	9.81	9.92	9.73	9.90	9.79	0.82
753	PE(18:2/22:4)-H	10.58	10.45	10.47	10.52	10.39	0.68	10.28	10.11	10.30	10.35	10.12	1.06	9.99	9.79	9.72	9.73	9.70	1.23
754	PE(18:2/22:5)-H	10.59	10.49	10.55	10.47	10.47	0.51	10.06	10.26	10.25	10.55	10.22	1.73	9.71	9.76	9.72	9.74	9.72	0.22
755	PE(18:2/22:6)-H	10.50	10.49	10.49	10.31	10.39	0.80	9.95	10.27	10.14	10.19	10.41	1.65	9.54	9.59	9.62	9.56	9.29	1.37
756	PE(O-16:0/16:0)-H	10.95	10.87	10.89	10.99	10.93	0.43	11.20	11.19	11.17	11.26	11.27	0.39	10.79	10.61	10.74	10.67	10.66	0.65
757	PE(O-16:0/16:1)-H	10.88	10.93	10.81	10.81	10.91	0.51	10.88	10.75	10.88	10.75	10.77	0.65	10.42	10.59	10.57	10.28	10.51	1.19
758	PE(O-16:0/18:0)-H	10.68	10.86	10.84	10.92	10.62	1.18	11.38	11.35	11.27	11.33	11.43	0.53	11.01	10.95	11.14	11.57	10.84	2.56
759	PE(O-16:0/18:1)-H	10.71	10.69	10.67	10.69	10.66	0.20	10.59	10.62	10.62	10.62	10.59	0.16	10.13	10.13	10.18	10.11	10.12	0.24
760	PE(O-16:0/18:2)-H	10.68	10.69	10.68	10.65	10.65	0.19	10.59	10.64	10.58	10.62	10.61	0.20	10.12	10.12	10.11	10.07	10.03	0.40
761	PE(O-16:0/18:3)-H	10.74	10.76	10.80	10.73	10.71	0.33	10.61	10.54	10.61	10.65	10.73	0.66	10.14	10.18	10.21	10.23	10.24	0.40
762	PE(O-16:0/20:1)-H	10.64	10.65	10.67	10.67	10.65	0.13	10.65	10.64	10.64	10.53	10.63	0.46	10.11	10.11	10.01	10.06	10.09	0.42
763	PE(O-16:0/20:2)-H	10.67	10.70	10.65	10.68	10.60	0.37	10.45	10.52	10.57	10.67	10.64	0.84	10.05	10.08	10.02	10.27	9.87	1.40
764	PE(O-16:0/20:3)-H	10.57	10.56	10.55	10.48	10.54	0.32	10.40	10.39	10.38	10.31	10.43	0.45	9.85	9.79	9.90	9.74	9.93	0.77
765	PE(O-16:0/20:4)-H	10.52	10.50	10.45	10.44	10.46	0.30	10.21	10.27	10.27	10.19	10.30	0.46	9.79	9.68	9.73	9.68	9.67	0.52
766	PE(O-16:0/20:5)-H	10.49	10.55	10.54	10.49	10.50	0.28	10.23	10.36	10.32	10.15	10.27	0.80	9.78	9.64	9.78	9.65	9.76	0.72
767	PE(O-16:0/22:4)-H	10.51	10.50	10.48	10.45	10.45	0.25	10.23	10.24	10.32	10.19	10.26	0.46	9.72	9.75	9.72	9.66	9.71	0.34
768	PE(O-16:0/22:5)-H	10.53	10.54	10.48	10.46	10.46	0.38	10.26	10.25	10.32	10.22	10.34	0.47	9.77	9.76	9.73	9.68	9.72	0.37
769	PE(O-16:0/22:6)-H	10.45	10.40	10.39	10.39	10.33	0.40	10.15	10.13	10.23	10.11	10.20	0.50	9.65	9.60	9.70	9.57	9.56	0.63
770	PE(O-18:0/16:0)-H	10.81	10.83	10.79	10.80	10.82	0.15	10.54	10.57	10.58	10.52	10.62	0.38	10.22	10.16	10.20	10.15	10.11	0.42
771	PE(O-18:0/16:1)-H	10.74	10.75	10.76	10.73	10.72	0.15	10.37	10.49	10.48	10.46	10.54	0.58	10.01	10.04	9.95	9.99	9.98	0.33
772	PE(O-18:0/18:0)-H	10.77	10.72	10.65	10.66	10.64	0.53	11.19	11.14	11.04	11.14	11.05	0.61	10.49	10.46	10.16	10.41	10.16	1.56

773	PE(O-18:0/18:1)-H	10.65	10.67	10.67	10.64	10.64	0.16	10.36	10.41	10.40	10.40	10.47	0.37	10.01	9.95	9.97	9.95	9.94	0.29
774	PE(O-18:0/18:2)-H	10.65	10.68	10.63	10.65	10.62	0.21	10.35	10.42	10.43	10.36	10.43	0.38	9.98	9.95	9.98	9.93	9.95	0.20
775	PE(O-18:0/18:3)-H	10.67	10.60	10.69	10.67	10.64	0.35	10.50	10.47	10.50	10.45	10.51	0.20	10.10	10.02	10.01	9.96	9.94	0.65
776	PE(O-18:0/20:1)-H	10.56	10.68	10.51	10.65	10.52	0.74	10.34	10.36	10.50	10.45	10.44	0.67	9.93	9.96	10.01	9.85	9.79	0.88
777	PE(O-18:0/20:2)-H	10.60	10.58	10.53	10.65	10.51	0.54	10.30	10.34	10.35	10.24	10.36	0.46	9.94	9.64	9.81	9.81	9.74	1.14
778	PE(O-18:0/20:3)-H	10.44	10.41	10.45	10.42	10.40	0.21	10.05	10.04	10.15	10.09	10.12	0.48	9.60	9.56	9.58	9.55	9.55	0.23
779	PE(O-18:0/20:4)-H	10.37	10.36	10.32	10.32	10.33	0.23	9.94	9.97	10.01	9.92	10.00	0.40	9.47	9.38	9.48	9.40	9.45	0.44
780	PE(O-18:0/20:5)-H	10.43	10.38	10.32	10.33	10.37	0.43	10.09	10.03	10.08	10.14	10.12	0.45	9.55	9.50	9.54	9.42	9.47	0.57
781	PE(O-18:0/22:4)-H	10.36	10.34	10.32	10.26	10.23	0.52	9.96	9.92	9.97	9.91	9.97	0.32	9.45	9.44	9.43	9.31	9.45	0.63
782	PE(O-18:0/22:5)-H	10.43	10.38	10.35	10.35	10.37	0.31	10.09	10.11	10.20	10.10	10.16	0.45	9.60	9.54	9.61	9.49	9.60	0.51
783	PE(O-18:0/22:6)-H	10.32	10.40	10.35	10.28	10.26	0.52	9.96	9.96	10.04	9.96	10.09	0.61	9.48	9.45	9.52	9.34	9.40	0.72
784	PE(P-14:0/18:0)-H	11.36	11.57	11.25	11.47	11.48	1.11	11.34	11.27	11.38	11.40	11.46	0.63	11.10	11.23	11.16	11.10	11.04	0.65
785	PE(P-14:0/18:1)-H	10.56	10.65	10.70	10.75	10.72	0.72	11.41	11.19	10.86	11.24	11.49	2.17	11.17	10.04	10.60	10.98	10.61	4.05
786	PE(P-16:0/16:0)-H	10.71	10.69	10.65	10.62	10.69	0.33	11.08	11.01	11.11	11.08	10.82	1.08	10.16	10.13	10.12	10.02	10.09	0.51
787	PE(P-16:0/16:1)-H	10.67	10.65	10.66	10.67	10.65	0.10	10.43	10.46	10.49	10.48	10.58	0.53	10.02	10.04	9.94	10.00	9.90	0.59
788	PE(P-16:0/18:0)-H	10.68	10.54	10.57	10.59	10.48	0.68	11.62	11.37	11.25	11.34	11.44	1.21	11.30	11.30	11.27	11.28	11.23	0.25
789	PE(P-16:0/18:1)-H	10.59	10.58	10.56	10.53	10.54	0.26	10.35	10.41	10.39	10.32	10.38	0.33	9.90	9.83	9.83	9.81	9.82	0.35
790	PE(P-16:0/18:2)-H	10.61	10.59	10.55	10.54	10.55	0.27	10.36	10.42	10.40	10.30	10.40	0.46	9.89	9.84	9.87	9.83	9.81	0.33
791	PE(P-16:0/18:3)-H	10.62	10.58	10.57	10.56	10.57	0.21	10.35	10.48	10.52	10.40	10.46	0.65	9.89	9.93	9.86	9.92	9.87	0.32
792	PE(P-16:0/20:1)-H	10.55	10.54	10.54	10.53	10.52	0.11	10.33	10.40	10.37	10.28	10.41	0.54	9.84	9.78	9.81	9.76	9.70	0.55
793	PE(P-16:0/20:2)-H	10.56	10.52	10.51	10.55	10.48	0.30	10.26	10.36	10.34	10.24	10.30	0.51	9.79	9.75	9.81	9.73	9.73	0.36
794	PE(P-16:0/20:3)-H	10.44	10.44	10.43	10.41	10.42	0.13	10.15	10.20	10.22	10.14	10.22	0.37	9.65	9.64	9.67	9.60	9.63	0.26
795	PE(P-16:0/20:4)-H	10.35	10.33	10.34	10.32	10.30	0.19	9.99	10.03	10.06	9.98	10.06	0.39	9.50	9.48	9.48	9.42	9.45	0.33
796	PE(P-16:0/20:5)-H	10.35	10.37	10.36	10.34	10.33	0.11	10.00	10.02	10.14	9.93	10.10	0.82	9.55	9.50	9.55	9.44	9.46	0.52
797	PE(P-16:0/22:4)-H	10.36	10.35	10.36	10.30	10.30	0.30	9.99	10.00	10.05	9.97	10.04	0.36	9.48	9.45	9.48	9.42	9.45	0.27
798	PE(P-16:0/22:5)-H	10.34	10.36	10.34	10.31	10.28	0.32	10.00	10.06	10.05	9.96	10.03	0.40	9.49	9.47	9.48	9.45	9.45	0.19
799	PE(P-16:0/22:6)-H	10.30	10.29	10.28	10.24	10.23	0.31	9.90	9.93	9.98	9.84	9.94	0.52	9.42	9.37	9.39	9.33	9.33	0.42
800	PE(P-16:1/18:1)-H	10.51	10.62	10.49	10.62	10.45	0.73	10.76	11.01	10.95	10.73	10.84	1.11	9.55	10.22	9.73	9.80	9.88	2.53
801	PE(P-18:0/16:0)-H	10.65	10.63	10.61	10.65	10.65	0.16	10.62	10.59	10.60	10.64	10.66	0.27	10.11	10.02	10.07	10.06	10.00	0.42

802	PE(P-18:0/16:1)-H	10.59	10.55	10.61	10.48	10.51	0.52	10.35	10.33	10.32	10.26	10.29	0.36	9.93	9.83	9.78	9.78	9.79	0.64
803	PE(P-18:0/18:0)-H	10.57	10.58	10.57	10.54	10.47	0.44	11.03	11.11	11.05	11.04	11.00	0.38	10.61	10.56	10.34	10.60	10.36	1.28
804	PE(P-18:0/18:1)-H	10.53	10.50	10.49	10.47	10.46	0.25	10.25	10.27	10.28	10.24	10.27	0.17	9.77	9.70	9.74	9.67	9.70	0.40
805	PE(P-18:0/18:2)-H	10.50	10.50	10.46	10.49	10.47	0.16	10.20	10.25	10.26	10.21	10.26	0.27	9.75	9.66	9.70	9.68	9.69	0.35
806	PE(P-18:0/18:3)-H	10.52	10.53	10.49	10.45	10.51	0.30	10.30	10.40	10.25	10.22	10.28	0.66	9.79	9.69	9.74	9.67	9.71	0.46
807	PE(P-18:0/20:1)-H	10.56	10.47	10.45	10.52	10.48	0.42	10.30	10.42	10.36	10.34	10.41	0.49	9.82	9.75	9.78	9.74	9.72	0.40
808	PE(P-18:0/20:2)-H	10.47	10.41	10.35	10.37	10.40	0.44	10.16	10.10	10.26	10.03	10.23	0.92	9.63	9.60	9.56	9.55	9.63	0.39
809	PE(P-18:0/20:3)-H	10.37	10.34	10.33	10.32	10.31	0.23	10.00	10.06	10.09	9.99	10.06	0.45	9.51	9.47	9.50	9.41	9.44	0.45
810	PE(P-18:0/20:4)-H	10.27	10.26	10.24	10.21	10.23	0.24	9.83	9.87	9.94	9.79	9.89	0.59	9.35	9.29	9.29	9.27	9.26	0.36
811	PE(P-18:0/20:5)-H	10.29	10.28	10.24	10.23	10.21	0.33	9.90	9.95	9.98	9.85	9.95	0.50	9.39	9.30	9.33	9.30	9.29	0.45
812	PE(P-18:0/22:4)-H	10.25	10.28	10.21	10.22	10.23	0.28	9.88	9.89	9.95	9.81	9.94	0.56	9.36	9.30	9.33	9.27	9.30	0.39
813	PE(P-18:0/22:5)-H	10.25	10.26	10.26	10.21	10.24	0.23	9.90	9.93	9.94	9.84	9.95	0.46	9.37	9.31	9.33	9.29	9.33	0.34
814	PE(P-18:0/22:6)-H	10.18	10.19	10.20	10.17	10.19	0.14	9.76	9.82	9.87	9.76	9.88	0.60	9.25	9.21	9.24	9.16	9.22	0.37
815	PE(P-18:1/16:0)-H	10.59	10.59	10.55	10.52	10.55	0.27	10.36	10.49	10.43	10.38	10.39	0.47	9.94	9.88	9.92	9.81	9.86	0.53
816	PE(P-18:1/16:1)-H	10.51	10.57	10.44	10.47	10.58	0.56	10.40	10.37	10.34	10.25	10.37	0.54	9.74	9.73	9.86	9.79	9.75	0.53
817	PE(P-18:1/18:1)-H	10.48	10.45	10.44	10.43	10.42	0.21	10.13	10.19	10.23	10.15	10.22	0.43	9.72	9.65	9.64	9.59	9.64	0.49
818	PE(P-18:1/18:2)-H	10.48	10.46	10.44	10.41	10.42	0.30	10.20	10.22	10.24	10.17	10.25	0.29	9.72	9.66	9.69	9.63	9.64	0.40
819	PE(P-18:1/18:3)-H	10.47	10.51	10.39	10.45	10.43	0.43	10.26	10.19	10.26	10.22	10.19	0.35	9.80	9.66	9.74	9.65	9.64	0.74
820	PE(P-18:1/20:1)-H	10.48	10.41	10.47	10.42	10.42	0.32	10.18	10.20	10.29	10.06	10.25	0.86	9.77	9.59	9.60	9.67	9.60	0.80
821	PE(P-18:1/20:2)-H	10.44	10.36	10.33	10.36	10.36	0.37	10.10	10.16	10.20	10.01	10.24	0.90	9.62	9.51	9.57	9.57	9.67	0.63
822	PE(P-18:1/20:3)-H	10.32	10.34	10.29	10.31	10.31	0.18	9.99	9.99	10.04	9.96	10.08	0.47	9.47	9.43	9.42	9.39	9.42	0.32
823	PE(P-18:1/20:4)-H	10.25	10.25	10.26	10.22	10.20	0.24	9.85	9.86	9.92	9.79	9.90	0.52	9.30	9.28	9.31	9.25	9.27	0.25
824	PE(P-18:1/20:5)-H	10.23	10.27	10.28	10.22	10.19	0.37	9.86	9.85	10.06	9.82	9.96	1.02	9.40	9.37	9.35	9.34	9.35	0.27
825	PE(P-18:1/22:4)-H	10.24	10.27	10.24	10.23	10.20	0.23	9.83	9.88	9.91	9.82	9.90	0.41	9.33	9.28	9.32	9.26	9.29	0.33
826	PE(P-18:1/22:5)-H	10.26	10.25	10.21	10.20	10.22	0.25	9.82	9.89	9.97	9.82	9.91	0.64	9.35	9.28	9.32	9.26	9.27	0.40
827	PE(P-18:1/22:6)-H	10.18	10.19	10.13	10.13	10.16	0.26	9.81	9.75	9.81	9.72	9.83	0.49	9.27	9.22	9.23	9.19	9.20	0.35
828	PE(P-18:2/18:2)-H	10.53	10.47	10.46	10.43	10.43	0.37	10.17	10.28	10.28	10.19	10.27	0.54	9.73	9.66	9.74	9.66	9.70	0.37
829	PE(P-18:2/20:4)-H	10.28	10.31	10.22	10.23	10.23	0.37	9.87	9.94	9.96	9.84	9.97	0.57	9.41	9.35	9.30	9.33	9.25	0.63
830	PE(P-18:2/22:6)-H	10.19	10.19	10.33	10.39	10.19	0.90	10.46	9.94	10.08	10.14	10.06	1.90	9.53	9.35	9.42	9.71	9.49	1.41

831	LPG(16:0)-H	12.92	12.78	12.85	12.87	12.77	0.47	12.78	12.60	12.56	12.58	12.59	0.70	11.64	11.60	11.64	11.65	11.63	0.16
832	LPG(16:1)-H	11.68	12.69	11.98	12.05	12.30	3.10	11.97	11.85	11.81	11.90	11.92	0.54	11.55	11.86	11.64	12.45	11.69	3.07
833	LPG(18:0)-H	12.58	12.57	12.65	12.61	12.60	0.23	11.72	11.66	11.77	11.72	11.68	0.33	11.45	11.61	11.47	11.45	11.55	0.61
834	LPG(18:1)-H	12.79	12.65	12.62	12.80	12.63	0.69	11.73	11.75	11.79	11.79	11.77	0.22	11.55	11.62	11.64	11.63	11.49	0.56
835	LPG(18:2)-H	N/A	N/A	N/A	N/A	N/A	#####	11.76	11.74	11.83	11.71	11.80	0.41	11.54	11.66	11.64	11.63	11.58	0.43
836	LPG(20:1)-H	12.53	12.54	12.47	13.23	12.63	2.48	11.83	12.01	11.61	12.46	11.72	2.79	11.60	12.57	12.55	12.38	N/A	3.73
837	LPG(20:3)-H	N/A	10.88	11.44	N/A	11.12	2.53	11.53	11.58	11.47	11.51	11.49	0.37	11.53	11.08	N/A	11.60	11.51	2.06
838	LPG(20:4)-H	N/A	11.74	11.77	11.91	N/A	0.73	12.20	12.62	11.61	11.58	12.53	4.09	12.37	12.30	11.86	11.76	12.22	2.27
839	PG(14:0/14:0)-H	7.35	7.33	7.50	7.27	7.30	1.23	7.10	7.23	7.32	7.61	7.28	2.59	6.75	6.67	6.67	6.34	6.61	2.41
840	PG(14:1/14:1)-H	7.41	7.12	6.91	7.33	6.83	3.56	7.10	7.20	6.74	7.52	7.31	4.01	N/A	6.75	7.54	7.69	6.90	6.43
841	PG(14:0/18:1)-H	7.02	7.07	6.98	7.05	7.08	0.58	6.86	6.94	6.89	6.86	6.89	0.48	6.38	6.28	6.36	6.31	6.36	0.63
842	PG(14:0/18:2)-H	7.01	6.96	7.11	7.28	7.01	1.83	6.96	6.89	6.96	6.76	6.68	1.85	6.41	6.30	6.45	6.32	6.28	1.15
843	PG(14:0/20:1)-H	7.11	7.42	7.17	7.37	6.79	3.49	6.76	6.97	6.38	6.61	7.25	4.92	N/A	6.94	6.91	7.25	7.09	2.20
844	PG(14:0/20:5)-H	6.82	7.07	7.23	7.43	6.93	3.41	6.69	6.83	7.18	7.03	6.89	2.69	6.14	6.22	6.26	6.36	6.19	1.28
845	PG(14:0/22:4)-H	7.26	6.67	7.38	7.50	7.68	5.23	7.10	7.37	6.71	6.41	6.57	5.77	7.52	5.66	7.21	5.68	7.35	13.91
846	PG(16:0/14:0)-H	7.17	7.29	7.18	7.19	7.20	0.65	7.03	7.03	7.03	6.96	7.11	0.77	6.61	6.53	6.54	6.51	6.49	0.68
847	PG(16:0/16:0)-H	7.05	7.10	7.00	7.11	7.09	0.66	6.88	6.90	6.94	6.82	6.92	0.71	6.45	6.39	6.40	6.37	6.36	0.54
848	PG(16:0/16:1)-H	7.02	6.96	6.97	7.05	7.03	0.54	6.83	6.85	6.89	6.79	6.85	0.51	6.39	6.34	6.35	6.32	6.33	0.46
849	PG(16:0/18:0)-H	6.86	6.92	7.01	6.96	6.88	0.86	6.73	6.73	6.78	6.69	6.74	0.43	6.33	6.27	6.31	6.24	6.22	0.73
850	PG(16:0/18:1)-H	6.84	6.91	6.84	6.91	6.90	0.48	6.66	6.69	6.72	6.63	6.69	0.54	6.27	6.20	6.23	6.18	6.21	0.57
851	PG(16:0/18:2)-H	6.81	6.89	6.83	6.95	6.87	0.80	6.65	6.71	6.75	6.66	6.72	0.63	6.29	6.18	6.22	6.17	6.20	0.80
852	PG(16:0/18:3)-H	6.87	6.87	7.06	7.11	6.87	1.74	6.68	6.71	6.84	6.86	6.75	1.16	6.31	6.32	6.30	6.26	6.18	0.94
853	PG(16:0/20:1)-H	6.73	6.87	6.72	6.88	6.73	1.18	6.57	6.60	6.60	6.58	6.53	0.45	6.15	6.08	6.13	6.08	6.16	0.62
854	PG(16:0/20:2)-H	6.80	6.81	6.73	6.88	6.82	0.80	6.62	6.61	6.64	6.59	6.64	0.32	6.22	6.13	6.11	6.10	6.14	0.78
855	PG(16:0/20:3)-H	6.76	6.96	6.81	6.87	6.80	1.11	6.63	6.71	6.65	6.59	6.64	0.67	6.30	6.14	6.13	6.15	6.22	1.15
856	PG(16:0/20:4)-H	6.45	6.58	6.55	6.53	6.58	0.79	6.41	6.38	6.41	6.32	6.39	0.56	5.95	5.95	5.90	5.88	5.90	0.57
857	PG(16:0/20:5)-H	6.96	7.18	6.78	6.64	6.56	3.65	6.36	6.66	6.51	6.47	6.33	2.09	5.97	5.87	5.97	5.91	5.76	1.47
858	PG(16:0/22:5)-H	6.59	6.58	6.58	6.62	6.73	0.94	6.60	6.57	6.73	6.33	6.86	3.01	6.01	6.05	5.71	5.86	5.96	2.23
859	PG(18:0/14:0)-H	6.94	7.06	7.09	7.11	7.09	0.98	6.98	7.01	6.87	6.92	6.77	1.36	6.44	6.47	6.36	6.47	6.32	1.07

860	PG(18:0/16:1)-H	6.80	6.92	6.85	6.85	6.85	0.65	6.65	6.70	6.76	6.61	6.73	0.89	6.26	6.13	6.17	6.20	6.19	0.79
861	PG(18:0/18:0)-H	6.79	6.80	6.79	6.80	6.89	0.65	6.56	6.61	6.67	6.55	6.59	0.70	6.22	6.06	6.06	6.10	6.16	1.09
862	PG(18:0/18:1)-H	6.72	6.79	6.72	6.80	6.76	0.58	6.56	6.59	6.61	6.53	6.59	0.50	6.15	6.08	6.10	6.07	6.09	0.54
863	PG(18:0/18:2)-H	6.66	6.72	6.68	6.77	6.73	0.63	6.54	6.56	6.58	6.50	6.54	0.46	6.13	6.07	6.06	6.04	6.05	0.54
864	PG(18:0/18:3)-H	6.75	6.79	6.72	6.98	6.86	1.50	6.65	6.58	6.89	6.72	6.55	1.99	6.20	6.35	6.29	6.11	6.11	1.71
865	PG(18:0/20:0)-H	7.19	7.07	6.76	6.62	7.31	4.17	7.18	6.66	7.04	6.93	7.15	3.00	6.89	6.46	6.53	6.98	6.58	3.47
866	PG(18:0/20:1)-H	6.70	6.63	6.67	6.73	6.65	0.57	6.47	6.48	6.52	6.42	6.49	0.58	6.05	6.02	5.97	5.96	5.97	0.67
867	PG(18:0/20:2)-H	6.71	6.69	6.62	6.69	6.68	0.50	6.50	6.51	6.55	6.44	6.50	0.60	6.09	6.04	6.01	5.99	6.01	0.65
868	PG(18:0/20:3)-H	6.64	6.67	6.69	6.74	6.74	0.65	6.48	6.52	6.56	6.46	6.47	0.63	6.07	6.03	6.02	5.97	6.00	0.62
869	PG(18:0/20:4)-H	6.35	6.42	6.37	6.48	6.38	0.82	6.25	6.24	6.29	6.18	6.29	0.76	5.83	5.77	5.78	5.75	5.73	0.62
870	PG(18:0/20:5)-H	6.52	6.67	7.05	6.98	6.74	3.21	6.41	6.76	6.36	6.22	6.32	3.23	6.84	6.66	6.85	6.72	N/A	1.38
871	PG(18:0/22:4)-H	7.18	6.88	6.48	6.44	6.81	4.54	6.73	6.27	6.03	6.79	6.09	5.54	6.02	6.37	6.05	6.32	6.09	2.63
872	PG(18:0/22:5)-H	7.12	6.39	6.81	6.45	6.71	4.43	6.29	6.73	6.26	6.08	6.32	3.77	5.89	5.75	6.10	6.05	5.78	2.69
873	PG(18:1/16:1)-H	6.84	6.91	6.91	6.88	6.86	0.46	6.63	6.67	6.70	6.61	6.66	0.58	6.26	6.17	6.22	6.14	6.17	0.78
874	PG(18:1/18:1)-H	6.65	6.76	6.66	6.76	6.72	0.79	6.53	6.55	6.58	6.51	6.56	0.41	6.13	6.06	6.07	6.04	6.07	0.56
875	PG(18:1/18:2)-H	6.63	6.73	6.66	6.79	6.85	1.35	6.57	6.65	6.61	6.49	6.54	0.94	6.11	6.07	6.08	6.02	6.01	0.71
876	PG(18:1/18:3)-H	7.82	7.87	7.98	7.50	7.20	4.16	6.68	6.63	6.64	6.48	6.65	1.22	6.23	6.22	6.05	6.05	6.09	1.44
877	PG(18:1/20:2)-H	6.89	6.59	6.66	6.62	6.90	2.25	6.41	6.43	6.48	6.42	6.72	2.02	6.08	6.00	6.02	6.10	6.05	0.71
878	PG(18:1/20:3)-H	5.32	5.46	5.51	5.61	5.55	1.99	5.15	5.24	5.18	5.19	5.23	0.73	4.75	4.80	4.81	4.96	4.82	1.61
879	PG(18:1/20:4)-H	5.22	5.49	5.28	5.23	5.32	2.09	5.11	5.11	5.11	5.06	5.13	0.51	4.64	4.65	4.61	4.71	4.64	0.74
880	PG(18:1/22:4)-H	7.37	7.14	7.45	7.15	7.61	2.73	7.82	7.51	7.30	7.97	7.15	4.55	7.48	7.81	8.02	7.93	7.69	2.69
881	PG(18:1/22:5)-H	6.87	7.66	6.92	7.64	6.96	5.60	7.14	7.13	7.24	6.94	7.81	4.58	7.18	7.49	7.52	7.27	7.30	2.02
882	PG(18:2/18:2)-H	6.73	6.78	6.73	6.76	6.74	0.30	6.55	6.55	6.61	6.53	6.65	0.77	6.11	6.04	6.07	6.02	6.15	0.86
883	PG(18:2/20:1)-H	5.42	5.25	5.71	5.45	5.66	3.39	5.56	5.16	5.58	5.52	5.24	3.64	5.16	5.93	5.91	5.89	5.38	6.37
884	PG(18:2/20:2)-H	7.40	7.73	7.67	7.36	7.98	3.37	7.57	7.46	7.86	7.53	7.55	2.04	7.45	7.19	7.38	7.45	7.31	1.49
885	PG(18:2/20:3)-H	7.07	7.21	7.50	7.15	7.74	3.80	7.39	7.48	7.68	7.47	7.08	2.91	7.72	7.74	7.77	7.39	7.31	2.87
886	PG(18:2/20:4)-H	7.11	6.55	6.98	6.56	6.70	3.78	6.74	7.00	7.11	6.87	7.09	2.23	7.04	6.59	6.75	6.81	7.45	4.80
887	PG(20:0/18:1)-H	6.59	6.78	6.46	6.90	6.83	2.75	6.41	6.69	6.28	6.51	6.36	2.45	7.51	7.20	7.25	7.55	7.50	2.24
888	PG(20:0/18:2)-H	6.92	6.74	6.82	6.72	6.49	2.41	6.10	6.39	6.35	6.44	6.22	2.18	6.41	6.23	6.72	6.44	6.38	2.77

889	LPI(18:0)-H	13.12	13.14	13.09	13.11	13.15	0.19	13.13	12.75	13.17	13.15	13.13	1.34	13.14	13.14	13.13	13.16	13.12	0.10
890	PI(14:0/18:1)-H	10.80	10.55	10.49	10.48	10.47	1.34	10.16	10.42	10.17	10.41	10.28	1.22	11.07	10.95	11.10	11.29	10.74	1.83
891	PI(14:0/18:2)-H	10.25	10.62	10.45	10.33	10.31	1.43	10.15	10.38	10.23	10.35	10.30	0.90	10.91	10.95	11.17	10.85	11.15	1.32
892	PI(14:0/20:1)-H	10.49	10.33	10.79	9.90	10.90	3.77	10.61	10.53	10.14	10.81	10.39	2.38	10.36	10.73	10.53	11.23	11.02	3.28
893	PI(14:0/20:2)-H	10.41	10.67	10.04	10.00	10.36	2.69	10.51	10.72	10.57	10.45	10.63	1.00	11.00	10.41	N/A	N/A	10.74	2.75
894	PI(14:0/20:3)-H	10.14	10.34	10.21	10.08	10.19	0.95	9.99	10.05	10.05	10.17	10.22	0.95	10.89	N/A	9.98	N/A	N/A	6.18
895	PI(14:0/20:4)-H	9.93	9.92	9.96	10.01	9.89	0.46	9.76	9.57	9.76	9.64	9.90	1.31	9.92	10.38	10.49	10.31	10.42	2.16
896	PI(14:0/20:5)-H	10.11	10.68	10.08	10.59	10.73	3.02	10.62	10.79	9.99	10.43	10.41	2.88	10.66	10.66	10.67	10.74	9.91	3.30
897	PI(14:0/22:5)-H	9.98	10.00	10.13	9.76	10.00	1.36	10.52	9.82	10.42	N/A	10.46	3.18	10.72	10.23	10.53	10.66	10.13	2.49
898	PI(16:0/16:0)-H	12.97	12.96	13.06	12.99	12.97	0.31	12.94	12.98	12.92	12.93	12.91	0.22	10.95	10.86	10.60	N/A	10.68	1.49
899	PI(16:0/16:1)-H	12.99	12.94	13.03	12.98	12.96	0.25	12.88	12.96	12.91	12.92	12.91	0.22	12.84	12.80	12.82	12.76	12.78	0.22
900	PI(16:0/18:0)-H	12.97	12.95	12.97	12.94	12.93	0.14	12.86	12.95	12.90	12.85	12.86	0.30	12.77	12.74	12.77	12.75	12.75	0.12
901	PI(16:0/18:1)-H	12.93	12.94	12.93	12.94	12.92	0.06	12.85	12.84	12.86	12.88	12.85	0.13	12.75	12.75	12.75	12.72	12.71	0.16
902	PI(16:0/18:2)-H	12.94	12.93	12.94	12.94	12.92	0.06	12.85	12.86	12.88	12.85	12.85	0.10	12.76	12.74	12.74	12.74	12.71	0.14
903	PI(16:0/18:3)-H	10.68	10.64	10.63	10.63	10.60	0.24	10.27	10.27	10.32	10.25	10.32	0.30	9.90	9.80	9.87	9.79	9.81	0.49
904	PI(16:0/20:1)-H	12.87	12.88	12.91	12.92	12.87	0.21	12.86	12.84	12.82	12.78	12.77	0.30	12.72	12.64	12.74	12.74	12.67	0.35
905	PI(16:0/20:2)-H	12.92	12.93	12.84	12.88	12.85	0.31	12.82	12.81	12.83	12.78	12.82	0.16	12.67	12.69	12.73	12.70	12.66	0.22
906	PI(16:0/20:3)-H	12.89	12.88	12.87	12.88	12.82	0.22	12.79	12.78	12.79	12.77	12.78	0.06	12.66	12.70	12.70	12.65	12.65	0.20
907	PI(16:0/20:4)-H	12.85	12.83	12.83	12.83	12.82	0.09	12.74	12.72	12.75	12.76	12.72	0.15	12.62	12.62	12.60	12.63	12.58	0.16
908	PI(16:0/20:5)-H	12.89	12.91	13.03	12.77	12.84	0.75	12.86	12.83	12.87	12.68	12.80	0.58	12.66	12.69	12.64	12.66	12.61	0.25
909	PI(16:0/22:4)-H	12.83	12.80	12.85	12.79	12.79	0.20	12.72	12.74	12.74	12.83	12.74	0.33	12.58	12.62	12.58	12.63	12.55	0.27
910	PI(16:0/22:5)-H	12.87	12.79	12.85	12.84	12.82	0.23	12.78	12.75	12.75	12.74	12.76	0.14	12.65	12.61	12.60	12.62	12.63	0.14
911	PI(16:0/22:6)-H	12.87	12.80	12.83	12.81	12.81	0.22	12.68	12.76	12.75	12.69	12.73	0.28	12.60	12.60	12.54	12.66	12.49	0.52
912	PI(18:0/14:0)-H	13.02	13.04	12.70	13.11	12.95	1.23	13.13	12.89	12.93	12.99	12.94	0.71	12.76	12.80	13.05	12.72	12.83	1.00
913	PI(18:0/16:1)-H	12.91	12.92	12.95	12.91	12.94	0.16	12.82	12.89	12.84	12.84	12.82	0.23	12.72	12.72	12.74	12.72	12.64	0.30
914	PI(18:0/18:0)-H	12.93	12.94	12.89	12.91	12.88	0.19	12.81	12.84	12.82	12.76	12.81	0.22	12.73	12.73	12.66	12.67	12.65	0.32
915	PI(18:0/18:1)-H	12.90	12.89	12.89	12.88	12.86	0.10	12.81	12.80	12.81	12.79	12.79	0.07	12.71	12.71	12.69	12.68	12.67	0.14
916	PI(18:0/18:2)-H	12.91	12.89	12.86	12.91	12.88	0.14	12.82	12.82	12.83	12.77	12.75	0.26	12.69	12.69	12.69	12.68	12.66	0.10
917	PI(18:0/20:0)-H	N/A	N/A	N/A	N/A	N/A	#####	10.71	10.74	10.88	10.79	10.84	0.68	10.43	10.46	10.43	10.36	10.36	0.43

918	PI(18:0/20:1)-H	12.87	12.99	12.92	12.88	12.96	0.39	12.91	12.85	12.89	12.76	12.81	0.48	12.79	12.64	12.62	12.69	12.60	0.59
919	PI(18:0/20:2)-H	12.83	12.80	12.88	12.87	12.84	0.24	12.76	12.79	12.74	12.83	12.76	0.27	12.67	12.65	12.66	12.66	12.61	0.17
920	PI(18:0/20:3)-H	12.86	12.84	12.81	12.82	12.81	0.16	12.75	12.77	12.73	12.75	12.74	0.12	12.64	12.62	12.61	12.60	12.57	0.21
921	PI(18:0/20:4)-H	12.80	12.76	12.77	12.77	12.79	0.12	12.70	12.71	12.70	12.71	12.66	0.15	12.57	12.54	12.56	12.56	12.49	0.26
922	PI(18:0/20:5)-H	12.85	12.79	12.73	12.89	12.86	0.51	12.72	12.69	12.77	12.63	12.71	0.40	12.55	12.56	12.55	12.58	12.50	0.25
923	PI(18:0/22:4)-H	12.85	12.74	12.74	12.79	12.74	0.38	12.66	12.70	12.73	12.71	12.69	0.20	12.56	12.56	12.57	12.50	12.58	0.25
924	PI(18:0/22:5)-H	12.76	12.73	12.76	12.83	12.78	0.27	12.71	12.71	12.70	12.73	12.67	0.15	12.52	12.50	12.58	12.57	12.52	0.27
925	PI(18:0/22:6)-H	12.77	12.80	12.72	12.70	12.72	0.31	12.72	12.73	12.66	12.63	12.65	0.33	12.43	12.55	12.57	12.55	12.46	0.49
926	PI(18:1/16:1)-H	10.44	10.46	10.43	10.44	10.44	0.10	10.11	10.12	10.11	10.05	10.12	0.28	9.63	9.56	9.62	9.60	9.65	0.34
927	PI(18:1/18:1)-H	12.87	12.87	12.89	12.90	12.86	0.13	12.81	12.81	12.80	12.76	12.78	0.17	12.68	12.67	12.68	12.66	12.64	0.13
928	PI(18:1/18:2)-H	12.87	12.86	12.88	12.85	12.86	0.08	12.79	12.81	12.82	12.79	12.78	0.14	12.70	12.70	12.66	12.66	12.68	0.16
929	PI(18:1/20:1)-H	12.79	12.82	12.82	12.81	12.85	0.15	12.79	12.82	12.77	12.77	12.88	0.36	12.63	12.64	12.58	12.71	12.55	0.48
930	PI(18:1/20:2)-H	12.85	12.84	12.75	12.85	12.84	0.32	12.78	12.76	12.75	12.77	12.74	0.13	12.69	12.60	12.58	12.60	12.63	0.35
931	PI(18:1/20:3)-H	12.87	12.81	12.85	12.86	12.77	0.34	12.69	12.77	12.73	12.72	12.70	0.23	12.57	12.63	12.57	12.62	12.59	0.22
932	PI(18:1/20:4)-H	12.80	12.73	12.82	12.79	12.80	0.27	12.71	12.71	12.69	12.67	12.66	0.17	12.55	12.57	12.58	12.57	12.52	0.18
933	PI(18:1/20:5)-H	12.70	12.61	N/A	12.79	12.92	1.04	12.30	12.89	12.91	12.79	12.67	1.96	12.56	12.57	12.52	12.66	12.61	0.42
934	PI(18:1/22:5)-H	12.76	12.71	12.95	12.87	12.93	0.81	12.73	12.79	12.72	12.73	12.71	0.25	12.54	12.72	12.51	12.52	12.57	0.66
935	PI(18:1/22:6)-H	12.86	12.68	12.76	12.88	12.76	0.66	12.73	12.61	12.62	12.80	12.61	0.68	12.50	12.44	12.46	12.49	12.56	0.38
936	PI(18:2/16:1)-H	10.50	10.41	10.42	10.41	10.37	0.45	10.04	10.08	10.10	10.05	10.11	0.32	9.59	9.58	9.59	9.57	9.55	0.21
937	PI(18:2/18:2)-H	12.92	12.81	12.84	12.94	12.92	0.44	12.78	12.78	12.83	12.80	12.77	0.18	12.72	12.72	12.66	12.66	12.63	0.30
938	PI(18:2/18:3)-H	10.56	10.61	N/A	10.31	10.37	1.39	12.91	12.96	12.70	13.37	12.87	1.93	12.89	12.70	12.61	12.75	12.68	0.83
939	PI(18:2/20:1)-H	12.79	12.83	12.99	N/A	N/A	0.79	12.71	12.74	12.75	12.79	12.67	0.36	12.70	12.57	12.68	12.60	12.57	0.51
940	PI(18:2/20:3)-H	10.10	10.39	10.22	9.94	10.03	1.71	10.02	9.81	9.72	9.67	9.66	1.53	12.63	12.70	12.65	12.59	12.61	0.34
941	PI(18:2/20:4)-H	9.91	9.94	9.73	9.87	10.03	1.09	9.57	9.67	9.59	9.56	9.47	0.76	12.58	12.58	12.60	12.58	12.53	0.19
942	PI(18:2/22:5)-H	9.96	10.18	10.18	9.99	9.79	1.66	10.10	9.59	9.66	9.36	9.77	2.78	N/A	10.34	10.31	10.09	10.49	1.57
943	PI(18:2/22:6)-H	10.59	10.96	11.07	9.83	11.34	5.44	10.17	11.45	11.14	10.58	10.59	4.73	10.66	10.88	10.60	11.13	11.24	2.60
944	PI(20:0/16:1)-H	10.77	10.06	10.83	10.29	10.17	3.38	10.81	11.11	10.69	10.41	10.26	3.14	10.87	10.15	10.72	10.70	10.46	2.66
945	PI(20:0/18:1)-H	9.98	9.97	9.92	9.93	9.95	0.23	10.78	10.70	10.79	10.85	10.83	0.54	10.48	10.35	10.23	10.15	10.30	1.22
946	PI(20:0/18:2)-H	9.96	10.05	9.81	9.89	10.23	1.61	10.25	10.12	10.56	10.40	10.91	2.93	12.83	12.60	12.63	12.70	12.65	0.71

947	PI(20:0/18:3)-H	10.27	10.11	9.81	10.29	10.44	2.37	10.88	10.95	10.74	10.56	10.69	1.42	10.26	10.18	10.66	N/A	10.43	2.02
948	PI(20:0/20:1)-H	10.06	10.01	9.88	9.39	10.19	3.13	9.32	11.21	10.69	10.41	9.38	8.13	10.21	10.66	10.47	10.00	10.17	2.55
949	PI(20:0/20:2)-H	10.31	10.54	10.60	9.95	10.45	2.47	10.63	9.97	10.03	10.51	10.69	3.29	10.21	10.70	10.60	10.35	10.63	1.97
950	PI(20:0/20:3)-H	12.80	12.74	12.78	12.75	12.84	0.31	12.79	12.68	12.74	12.71	12.69	0.36	12.55	12.57	12.65	12.62	12.52	0.43
951	PI(20:0/20:4)-H	12.85	12.80	12.72	12.89	12.79	0.50	12.65	12.65	12.61	12.71	12.53	0.52	12.46	12.52	12.54	12.53	12.50	0.26
952	LPS(16:0)-H	12.75	12.84	12.83	12.71	13.02	0.92	12.80	12.72	12.76	12.70	12.75	0.31	12.54	12.57	12.61	12.55	12.53	0.25
953	LPS(18:1)-H	12.79	12.61	12.73	12.65	12.76	0.60	12.66	12.49	12.52	12.64	12.55	0.59	12.39	12.60	12.42	12.39	12.64	0.99
954	LPS(18:2)-H	12.92	12.22	12.59	12.95	12.50	2.42	12.73	12.84	12.53	12.87	12.45	1.49	12.56	12.44	12.45	12.45	12.41	0.47
955	LPS(20:4)-H	12.41	12.90	N/A	N/A	N/A	2.73	13.13	13.15	13.10	13.22	13.19	0.37	12.70	12.50	12.22	12.25	N/A	1.81
956	LPS(20:5)-H	13.18	13.20	13.18	13.19	13.24	0.18	13.21	13.23	13.20	13.19	13.19	0.11	10.36	10.81	10.56	10.50	10.20	2.17
957	PS(14:0/14:0)-H	11.05	10.78	10.50	10.37	10.56	2.49	10.79	10.72	10.59	10.44	N/A	1.45	11.15	10.73	10.46	10.38	10.20	3.48
958	PS(14:0/18:2)-H	10.72	10.60	10.64	10.65	10.70	0.44	10.32	10.44	10.49	10.33	10.38	0.69	10.04	9.98	10.08	9.70	9.99	1.49
959	PS(14:0/20:1)-H	10.54	10.60	10.57	10.41	10.84	1.47	10.19	9.95	10.53	10.20	10.92	3.65	10.26	9.54	9.88	10.13	9.21	4.38
960	PS(14:0/20:2)-H	10.49	10.29	10.32	10.36	10.07	1.51	10.67	11.03	11.60	10.56	11.51	4.28	10.50	10.47	11.20	11.18	N/A	3.76
961	PS(14:0/20:3)-H	10.51	10.56	10.66	10.38	10.49	0.98	10.09	9.88	10.04	10.05	10.46	2.12	9.57	9.57	9.68	9.69	9.43	1.08
962	PS(14:0/20:4)-H	10.34	10.41	10.39	10.33	10.26	0.58	9.77	10.04	10.01	10.09	10.01	1.24	9.51	9.53	9.49	9.45	9.48	0.32
963	PS(14:0/22:4)-H	10.05	10.38	10.17	10.54	10.30	1.84	9.81	9.86	10.21	10.11	9.78	1.92	10.08	9.56	9.42	9.60	9.46	2.75
964	PS(14:0/22:5)-H	10.41	10.57	10.45	10.51	10.26	1.12	10.12	10.54	10.14	10.19	10.29	1.68	9.63	9.46	9.48	9.64	9.71	1.12
965	PS(14:0/22:6)-H	10.41	10.32	10.36	10.43	10.31	0.50	10.04	9.96	9.97	10.25	10.26	1.46	9.44	9.41	9.33	9.64	9.45	1.21
966	PS(16:0/14:0)-H	10.77	10.77	10.89	11.23	10.66	2.05	11.02	11.39	11.76	10.73	10.49	4.58	11.33	10.95	11.30	11.09	11.19	1.41
967	PS(16:0/16:0)-H	10.80	10.92	12.43	11.00	11.04	5.98	11.38	11.26	11.26	11.40	11.26	0.62	10.85	10.97	10.91	10.96	10.83	0.58
968	PS(16:0/16:1)-H	11.17	11.17	10.53	10.90	11.36	2.93	11.39	11.23	11.01	11.11	10.91	1.69	9.87	10.93	10.92	10.99	10.50	4.47
969	PS(16:0/18:0)-H	N/A	13.07	13.02	13.40	N/A	1.56	12.87	13.09	13.10	13.08	12.97	0.79	13.08	13.08	12.79	12.72	12.88	1.29
970	PS(16:0/18:1)-H	11.03	11.09	10.99	11.31	11.13	1.12	10.98	10.70	10.70	10.86	10.70	1.18	10.24	10.54	10.55	10.46	10.36	1.23
971	PS(16:0/18:2)-H	10.58	10.65	10.59	10.61	10.64	0.30	10.41	10.26	10.44	10.31	10.18	1.05	9.83	9.79	9.73	9.74	9.76	0.41
972	PS(16:0/18:3)-H	10.79	11.37	10.89	11.02	10.61	2.60	10.43	10.57	11.08	10.92	10.91	2.50	10.77	10.88	10.96	N/A	10.56	1.61
973	PS(16:0/20:1)-H	10.70	10.57	10.49	10.52	10.43	0.95	11.06	10.68	11.28	11.09	11.00	1.98	10.79	11.24	9.92	10.41	10.20	4.93
974	PS(16:0/20:2)-H	10.95	10.46	10.92	10.31	11.19	3.43	11.15	10.32	10.57	11.05	10.51	3.35	10.75	10.33	9.62	10.40	9.76	4.60
975	PS(16:0/20:3)-H	10.12	10.59	10.56	10.36	10.27	1.91	9.90	10.09	9.93	9.87	10.02	0.91	9.51	9.51	9.50	9.55	9.38	0.68

976	PS(16:0/20:4)-H	10.15	10.19	10.17	10.26	10.17	0.43	9.70	9.72	9.80	9.62	9.79	0.74	9.25	9.31	9.23	9.24	9.29	0.34
977	PS(16:0/20:5)-H	10.55	10.79	10.62	9.91	10.55	3.20	10.95	10.72	11.17	10.35	10.69	2.86	N/A	10.47	N/A	11.06	10.86	2.79
978	PS(16:0/22:4)-H	10.70	10.54	10.61	10.26	10.50	1.57	10.09	10.16	11.79	11.42	10.81	6.94	10.36	10.59	10.96	10.56	10.70	2.07
979	PS(16:0/22:5)-H	10.11	10.26	10.27	10.51	10.41	1.49	10.27	10.05	10.26	11.79	10.55	6.60	9.50	9.40	9.34	9.21	9.28	1.18
980	PS(18:0/14:0)-H	10.55	10.58	10.55	10.59	10.95	1.62	10.45	10.77	10.50	10.33	10.54	1.53	9.90	9.87	10.14	9.95	10.39	2.17
981	PS(18:0/16:1)-H	10.62	10.48	10.58	10.44	10.55	0.72	10.19	10.43	10.48	10.83	10.20	2.50	9.88	9.72	9.56	9.80	9.72	1.22
982	PS(18:0/18:0)-H	10.44	10.42	10.39	10.37	10.35	0.32	10.06	10.14	10.18	10.14	10.17	0.49	9.63	9.56	9.62	9.53	9.57	0.45
983	PS(18:0/18:1)-H	10.40	10.44	10.42	10.40	10.29	0.54	10.20	10.21	10.18	10.09	10.22	0.52	9.67	9.62	9.61	9.56	9.56	0.47
984	PS(18:0/18:2)-H	10.55	10.44	10.45	10.39	10.45	0.55	10.07	10.12	10.10	10.06	10.10	0.25	9.65	9.54	9.63	9.53	9.60	0.57
985	PS(18:0/18:3)-H	10.49	10.50	10.86	10.81	11.19	2.70	10.87	10.85	10.74	10.73	10.68	0.77	10.64	10.59	10.60	10.67	10.67	0.36
986	PS(18:0/20:0)-H	9.93	9.93	9.89	9.88	9.89	0.24	9.55	9.57	9.63	9.52	9.63	0.49	9.11	9.09	9.08	9.01	9.00	0.53
987	PS(18:0/20:1)-H	10.37	10.32	10.29	10.39	10.18	0.80	10.69	10.80	10.60	10.75	10.80	0.78	9.61	9.48	9.58	9.67	9.46	0.92
988	PS(18:0/20:2)-H	10.55	10.32	10.32	10.32	10.20	1.25	10.54	11.22	11.11	10.42	10.39	3.70	9.54	9.56	9.53	9.54	9.95	1.88
989	PS(18:0/20:3)-H	10.13	10.25	10.04	10.04	10.19	0.91	10.08	10.20	9.69	9.61	9.74	2.64	9.37	9.24	9.41	9.47	9.56	1.28
990	PS(18:0/20:4)-H	10.10	10.15	10.00	10.00	10.01	0.69	9.62	9.72	9.71	9.60	9.69	0.53	9.20	9.20	9.12	9.14	9.07	0.61
991	PS(18:0/22:4)-H	12.73	12.68	12.50	12.66	12.63	0.68	10.29	10.82	10.42	10.15	10.99	3.40	9.27	8.86	9.18	8.91	10.53	7.30
992	PS(18:0/22:6)-H	9.86	10.14	10.29	9.85	9.89	2.00	10.13	10.40	10.76	10.02	9.71	3.91	10.36	10.52	10.42	9.63	10.20	3.45
993	PS(18:1/16:1)-H	10.52	10.50	10.49	10.46	10.49	0.18	10.10	10.14	10.21	10.09	10.17	0.48	9.70	9.71	9.72	9.61	9.61	0.59
994	PS(18:1/18:1)-H	10.53	10.71	10.47	10.42	10.41	1.18	10.36	10.01	10.22	10.21	10.28	1.28	9.57	9.82	9.77	9.88	9.87	1.28
995	PS(18:1/18:2)-H	10.41	10.45	10.47	10.41	10.42	0.25	9.96	10.03	10.10	9.98	10.06	0.55	9.51	9.55	9.50	9.34	9.46	0.84
996	PS(18:1/18:3)-H	10.60	10.38	10.35	10.26	10.67	1.69	10.52	10.33	10.86	10.64	10.26	2.29	10.21	10.35	10.67	10.64	10.24	2.10
997	PS(18:1/20:1)-H	10.33	10.76	10.60	11.01	10.42	2.55	11.11	10.66	10.71	10.68	10.83	1.74	9.49	10.25	10.31	10.27	10.66	4.20
998	PS(18:1/20:2)-H	10.00	10.36	10.39	10.09	9.99	1.93	10.26	10.89	10.49	10.68	10.56	2.19	10.32	9.62	9.82	9.40	9.74	3.49
999	PS(18:1/20:3)-H	10.11	10.23	10.13	10.09	10.20	0.59	9.60	9.79	9.77	9.61	9.92	1.36	9.31	9.22	9.22	9.21	9.18	0.54
1000	PS(18:1/20:4)-H	10.05	10.05	9.94	9.96	9.98	0.50	9.59	9.67	9.73	9.53	9.60	0.82	9.12	9.17	9.14	9.07	9.07	0.47
1001	PS(18:1/20:5)-H	10.19	9.86	10.02	9.94	10.19	1.46	9.59	9.93	9.80	9.60	9.72	1.46	9.16	9.11	9.25	9.37	9.45	1.56
1002	PS(18:1/22:4)-H	10.02	9.96	10.10	10.09	10.04	0.57	9.81	9.54	10.17	9.68	9.80	2.40	9.72	9.11	9.65	8.99	9.45	3.44
1003	PS(18:1/22:5)-H	10.12	10.02	9.98	10.04	10.11	0.60	9.78	9.72	9.92	9.78	10.14	1.69	9.38	9.11	9.08	9.10	9.09	1.43
1004	PS(18:1/22:6)-H	9.86	9.89	9.85	10.01	9.90	0.64	10.58	10.39	9.68	9.34	9.45	5.68	9.10	9.30	8.96	8.97	9.21	1.61

1005	PS(18:2/16:1)-H	10.54	10.53	10.48	10.51	10.48	0.27	10.13	10.18	10.19	10.18	10.18	0.25	9.70	9.70	9.70	9.66	9.70	0.20
1006	PS(18:2/18:2)-H	10.37	10.38	10.36	10.34	10.36	0.15	10.13	10.22	10.17	10.15	10.20	0.34	9.55	9.50	9.63	9.47	9.50	0.64
1007	PS(18:2/18:3)-H	10.27	10.40	10.28	10.29	10.47	0.83	10.47	9.74	10.59	9.45	10.22	4.84	9.59	10.27	9.90	9.88	9.58	2.87
1008	PS(18:2/20:1)-H	10.74	10.75	10.60	10.41	10.25	2.05	10.31	10.75	10.48	10.68	10.16	2.37	9.76	10.28	10.21	10.11	10.05	2.01
1009	PS(18:2/20:2)-H	10.65	10.54	10.76	10.42	10.02	2.74	10.96	11.22	10.29	10.83	10.68	3.20	9.71	10.07	10.12	9.50	9.74	2.66
1010	PS(18:2/20:3)-H	10.24	10.17	10.18	10.29	10.13	0.62	9.83	9.97	9.82	9.94	9.97	0.75	9.54	9.26	9.27	9.34	9.36	1.22
1011	PS(18:2/20:4)-H	10.00	10.07	10.06	9.96	9.98	0.48	9.87	9.80	9.83	9.68	10.00	1.15	9.29	9.24	9.10	9.14	9.21	0.84
1012	PS(18:2/20:5)-H	10.20	10.06	9.96	10.16	10.00	1.02	10.04	9.53	9.86	9.70	10.18	2.65	9.13	9.55	9.34	9.19	9.04	2.15
1013	PS(18:2/22:4)-H	10.26	10.23	9.97	10.19	10.14	1.12	9.97	10.23	10.27	10.19	10.26	1.21	9.63	9.62	9.67	9.52	9.28	1.64
1014	PS(18:2/22:5)-H	10.19	10.26	10.13	10.25	10.18	0.52	10.25	10.28	10.24	10.20	10.26	0.27	9.70	9.64	9.70	9.63	9.65	0.35
1015	PS(18:2/22:6)-H	10.01	10.06	9.82	10.06	9.91	1.05	9.96	10.11	10.11	10.03	10.00	0.68	9.43	9.48	9.52	9.74	9.49	1.27
1016	PS(20:0/16:1)-H	10.62	10.24	10.70	10.32	10.75	2.20	10.10	10.47	10.31	10.57	10.13	2.00	10.01	9.62	10.10	9.92	9.62	2.26
1017	PS(20:0/18:1)-H	10.66	10.64	10.52	10.61	10.69	0.61	10.61	10.64	10.60	10.65	10.72	0.43	10.30	10.25	10.16	10.15	10.06	0.93
1018	PS(20:0/18:2)-H	10.55	10.50	10.52	10.51	10.49	0.22	10.14	10.18	10.21	10.18	10.22	0.31	9.78	9.70	9.70	9.69	9.76	0.42
1019	PS(20:0/18:3)-H	10.07	10.27	10.18	10.71	10.27	2.36	10.22	10.09	10.09	10.51	10.49	2.03	10.03	9.54	9.93	10.06	10.37	2.98
1020	PS(20:0/20:1)-H	9.97	10.10	10.14	9.83	10.13	1.32	9.84	10.51	10.33	10.19	10.17	2.41	9.50	9.58	8.99	9.65	9.68	3.00
1021	PS(20:0/20:2)-H	10.43	10.47	10.29	10.16	10.39	1.19	10.05	9.86	10.20	10.23	10.22	1.58	9.47	9.37	9.64	9.77	9.48	1.64
1022	PS(20:0/20:3)-H	10.35	10.28	10.23	10.26	10.37	0.58	9.95	9.96	10.07	9.91	9.96	0.62	9.41	9.34	9.47	9.36	9.46	0.64
1023	PS(20:0/20:4)-H	9.99	9.94	9.95	9.94	9.91	0.29	9.60	9.71	9.64	9.61	9.66	0.43	9.15	9.17	9.25	9.14	9.13	0.53
1024	PS(20:0/20:5)-H	10.31	10.47	10.43	10.04	10.46	1.73	11.04	11.51	10.86	11.04	11.88	3.73	9.58	8.91	9.58	9.12	9.54	3.34
1025	PS(20:0/22:4)-H	10.06	9.98	9.94	10.34	10.03	1.57	9.61	9.47	9.84	9.93	10.36	3.46	9.12	9.11	8.96	9.13	9.21	0.97
1026	PS(20:0/22:5)-H	10.09	10.06	10.06	10.04	10.01	0.26	9.66	9.72	9.73	9.68	9.73	0.32	9.24	9.21	9.24	9.17	9.16	0.40
1027	PS(20:0/22:6)-H	9.66	9.98	10.06	10.13	9.94	1.81	10.77	9.76	9.98	9.64	9.58	4.88	9.26	9.13	9.10	9.21	9.05	0.92
1028	PA(14:0/18:1)-H	11.19	10.89	10.50	10.88	N/A	2.59	11.17	11.07	11.37	11.03	11.06	1.25	11.19	10.63	N/A	11.26	11.32	2.87
1029	PA(16:0/14:0)-H	10.70	10.11	10.65	9.89	10.91	4.11	10.71	10.79	10.80	11.17	10.69	1.79	11.00	10.63	10.01	10.72	10.32	3.60
1030	PA(16:0/16:0)-H	11.18	10.86	10.83	10.98	10.90	1.29	11.38	11.20	11.24	11.13	11.17	0.83	11.35	10.75	10.81	10.85	10.98	2.19
1031	PA(16:0/18:0)-H	10.54	11.61	11.74	11.61	11.44	4.26	11.23	11.20	11.30	11.37	11.35	0.64	11.27	11.23	11.31	11.21	10.88	1.55
1032	PA(16:0/18:1)-H	10.58	10.56	10.53	10.57	10.60	0.24	10.55	10.83	10.37	10.95	10.69	2.13	10.86	10.42	10.38	10.14	10.05	3.03
1033	PA(16:0/18:2)-H	10.61	10.68	10.58	10.49	10.48	0.79	10.32	10.47	10.35	10.38	10.41	0.53	9.92	9.88	9.88	9.93	9.93	0.26

1034	PA(16:0/20:1)-H	11.27	11.24	11.19	N/A	11.48	1.13	10.87	11.22	10.85	10.87	10.98	1.42	10.50	10.43	10.84	10.57	11.12	2.67
1035	PA(16:0/20:2)-H	10.54	10.78	10.56	11.27	10.62	2.84	10.57	10.86	11.06	10.72	10.86	1.69	11.29	9.83	10.28	11.06	10.74	5.55
1036	PA(16:0/20:3)-H	10.37	10.38	10.50	10.47	10.28	0.85	10.01	10.48	10.25	9.89	10.25	2.30	9.48	9.61	9.76	9.63	9.69	1.09
1037	PA(16:0/20:4)-H	10.41	10.33	10.38	10.42	10.13	1.14	10.01	9.83	10.01	9.53	9.68	2.14	9.78	9.23	10.24	9.14	9.32	4.82
1038	PA(16:0/22:4)-H	10.91	10.40	10.92	10.37	10.40	2.70	10.36	10.02	10.79	10.34	10.37	2.67	11.02	10.02	9.52	10.99	10.56	6.22
1039	PA(16:0/22:5)-H	12.34	12.27	12.19	12.39	12.50	0.95	11.74	12.23	12.13	12.05	12.33	1.86	12.14	12.11	11.88	12.29	12.12	1.21
1040	PA(16:0/22:6)-H	12.15	N/A	12.12	12.85	12.76	3.12	12.76	11.05	12.70	12.13	12.74	5.97	12.43	12.02	12.81	12.65	11.92	3.16
1041	PA(18:0/18:0)-H	10.46	10.61	10.62	10.61	10.51	0.68	10.88	11.11	11.14	11.06	11.33	1.43	11.19	11.33	11.21	11.36	11.23	0.67
1042	PA(18:0/18:1)-H	10.54	10.53	10.51	10.50	10.50	0.18	10.30	10.33	10.37	10.27	10.35	0.38	9.88	9.83	9.82	9.79	9.79	0.36
1043	PA(18:0/18:2)-H	10.56	10.51	10.50	10.50	10.51	0.23	10.33	10.37	10.36	10.30	10.34	0.27	9.89	9.80	9.83	9.80	9.77	0.44
1044	PA(18:0/18:3)-H	10.55	10.62	10.51	10.51	10.56	0.40	10.41	10.36	10.44	10.45	10.50	0.47	9.84	9.84	10.05	9.73	9.74	1.34
1045	PA(18:0/20:0)-H	10.56	10.40	10.56	10.53	10.54	0.62	10.83	10.82	10.78	10.78	10.81	0.22	11.40	10.88	10.82	10.24	10.87	3.76
1046	PA(18:0/20:1)-H	10.59	10.48	10.47	10.41	10.38	0.77	10.33	10.32	10.47	10.20	10.39	0.96	9.72	9.84	9.75	9.69	9.71	0.60
1047	PA(18:0/20:2)-H	10.59	10.44	10.47	10.50	10.37	0.77	10.22	10.18	10.43	10.28	10.22	0.96	9.68	9.95	9.66	9.60	9.74	1.37
1048	PA(18:0/20:3)-H	10.43	10.42	10.33	10.35	10.34	0.44	10.12	10.16	10.23	10.16	10.18	0.38	9.68	9.63	9.65	9.53	9.62	0.59
1049	PA(18:0/20:4)-H	10.34	10.33	10.32	10.31	10.28	0.24	9.96	9.96	10.06	9.92	10.04	0.61	10.89	11.08	N/A	10.89	N/A	1.00
1050	PA(18:0/20:5)-H	10.37	10.39	10.28	10.43	10.30	0.62	10.13	10.00	10.17	9.95	10.14	0.95	N/A	N/A	N/A	10.58	11.25	4.37
1051	PA(18:0/22:4)-H	10.33	10.33	10.26	10.25	10.24	0.43	9.95	9.97	10.05	9.94	10.02	0.45	10.93	10.81	10.60	11.00	11.08	1.71
1052	PA(18:0/22:5)-H	12.80	12.38	12.76	12.98	12.33	2.23	12.00	11.88	11.98	12.16	12.33	1.46	12.38	12.15	12.10	12.77	12.09	2.37
1053	PA(18:0/22:6)-H	10.27	10.31	10.28	10.26	10.24	0.26	9.89	9.88	9.96	9.92	9.93	0.31	N/A	10.66	10.88	10.56	10.99	1.83
1054	PA(18:1/16:1)-H	11.23	11.52	11.39	10.96	11.40	1.93	10.45	11.21	10.89	11.54	11.16	3.68	11.04	11.43	11.84	N/A	11.96	3.61
1055	PA(18:1/18:1)-H	10.51	10.54	10.52	10.52	10.54	0.14	10.25	10.28	10.28	10.24	10.21	0.27	11.23	11.13	11.26	11.21	11.11	0.58
1056	PA(18:1/18:2)-H	10.64	10.56	10.52	10.58	10.51	0.48	10.26	10.29	10.30	10.29	10.29	0.14	10.72	11.05	11.32	11.11	10.85	2.13
1057	PA(18:1/18:3)-H	10.78	10.24	10.39	10.40	10.46	1.89	10.42	10.27	10.33	10.29	10.57	1.18	10.72	N/A	10.31	10.85	10.98	2.72
1058	PA(18:1/20:1)-H	10.55	10.36	10.46	10.17	10.69	1.89	10.68	10.41	10.79	10.52	10.21	2.13	10.50	10.21	10.13	10.32	10.67	2.11
1059	PA(18:1/20:2)-H	10.32	10.42	10.43	10.46	10.44	0.53	10.10	9.88	10.06	10.12	10.23	1.26	10.72	10.69	10.75	10.45	11.04	1.96
1060	PA(18:1/20:3)-H	10.41	10.37	10.41	10.37	10.25	0.64	9.95	10.00	9.98	9.93	10.06	0.52	10.85	11.11	N/A	10.87	10.98	1.09
1061	PA(18:1/20:4)-H	10.31	10.19	10.22	10.18	10.14	0.61	9.64	9.67	9.71	9.65	9.67	0.29	10.57	10.45	10.27	10.60	10.64	1.44
1062	PA(18:1/20:5)-H	10.61	10.38	10.17	10.37	10.16	1.80	9.76	9.81	9.68	9.68	9.92	1.03	10.61	N/A	10.89	10.46	10.85	1.90

1063	PA(18:1/22:4)-H	10.34	10.23	10.05	10.16	10.24	1.04	9.85	9.73	9.78	9.67	9.66	0.82	11.09	10.81	10.75	10.92	10.34	2.57
1064	PA(18:1/22:5)-H	10.14	10.04	10.36	10.10	10.32	1.37	9.60	9.81	9.68	9.64	9.77	0.92	10.19	10.67	N/A	10.91	10.20	3.42
1065	PA(18:1/22:6)-H	10.24	10.08	10.08	10.09	9.97	0.94	9.65	9.54	9.60	9.51	9.63	0.63	10.36	10.58	10.82	10.49	10.54	1.59
1066	PA(18:2/16:1)-H	10.73	10.67	10.59	10.37	10.44	1.40	10.33	10.17	10.37	10.47	10.45	1.18	11.20	10.81	N/A	10.56	10.70	2.53
1067	PA(18:2/18:2)-H	10.42	10.50	10.65	10.16	10.20	1.97	9.96	10.23	10.21	9.90	10.23	1.63	N/A	N/A	N/A	N/A	N/A	#####
1068	PA(18:2/20:1)-H	10.06	10.25	10.29	10.13	10.56	1.86	10.24	9.81	9.83	9.78	10.02	1.98	10.38	8.95	9.37	9.49	9.33	5.57
1069	PA(18:2/20:3)-H	10.77	10.32	N/A	N/A	10.41	2.23	10.81	9.91	10.86	10.25	10.44	3.79	9.29	9.50	9.95	10.06	9.40	3.56
1070	PA(18:2/20:4)-H	10.09	10.05	10.01	10.41	10.84	3.40	9.64	10.04	9.99	10.02	9.72	1.91	9.15	9.22	9.14	8.98	9.31	1.32
1071	PA(18:2/22:6)-H	10.09	10.67	10.58	10.39	10.62	2.26	10.45	10.47	10.71	10.65	10.45	1.20	10.86	10.14	10.31	10.17	10.95	3.71
1072	PA(20:0/16:1)-H	10.48	10.41	10.49	10.48	10.49	0.34	10.37	10.38	10.43	10.34	10.36	0.33	9.87	9.57	9.82	9.84	9.73	1.23
1073	PA(20:0/18:1)-H	10.46	10.46	10.45	10.44	10.42	0.16	10.18	10.23	10.24	10.18	10.25	0.34	9.72	9.70	9.69	9.61	9.65	0.45
1074	PA(20:0/18:2)-H	10.46	10.44	10.42	10.38	10.43	0.28	10.12	10.21	10.25	10.14	10.20	0.51	9.70	9.65	9.65	9.61	9.65	0.35
1075	PA(20:0/18:3)-H	10.49	10.52	10.43	10.51	10.45	0.36	10.21	10.21	10.23	10.40	10.41	0.99	9.84	9.75	9.79	9.67	9.84	0.70
1076	PA(20:0/20:1)-H	10.49	10.60	10.56	10.62	10.30	1.21	10.00	10.40	10.34	10.22	10.24	1.47	9.69	9.69	9.76	9.57	9.88	1.18
1077	PA(20:0/20:2)-H	10.37	10.24	10.54	10.32	10.39	1.07	10.10	10.14	10.18	10.05	10.14	0.48	9.64	9.64	9.62	9.51	9.49	0.78
1078	PA(20:0/20:3)-H	10.31	10.30	10.25	10.30	10.27	0.25	10.01	10.03	10.04	9.97	10.04	0.28	9.51	9.41	9.47	9.37	9.39	0.62
1079	PA(20:0/20:4)-H	10.21	10.27	10.20	10.19	10.19	0.32	9.80	9.86	9.90	9.79	9.89	0.49	9.32	9.27	9.29	9.23	9.23	0.39
1080	PA(20:0/20:5)-H	10.28	10.25	10.26	10.24	10.18	0.36	9.84	9.92	9.96	9.78	9.83	0.74	9.38	9.29	9.37	9.28	9.24	0.68
1081	PA(20:0/22:4)-H	10.28	10.22	10.23	10.19	10.18	0.38	9.88	9.92	9.92	9.75	9.89	0.73	9.28	9.27	9.33	9.26	9.24	0.36
1082	PA(20:0/22:5)-H	10.25	10.24	10.17	10.14	10.20	0.44	9.87	9.88	9.94	9.82	9.94	0.53	9.32	9.30	9.31	9.26	9.29	0.28
1083	PA(20:0/22:6)-H	10.17	10.15	10.10	10.11	10.14	0.27	9.77	9.72	9.81	9.71	9.84	0.59	9.24	9.22	9.25	9.19	9.11	0.63

Supplementary- Table S3: TAG's species with their respective isomers.

S.No.	Lipids	FA composition (Total carbon/ un-saturation)	Isomers	% Species abundance	% Isomer abundance
1	TAG(42:2/FA18:2)+NH4	42/2	FA18:2	0.004	100.000
2	TAG(44:2/FA16:0)+NH4	44/2	FA16:0	0.049	58.464
3	TAG(44:2/FA18:2)+NH4	44/2	FA18:2		41.536
4	TAG(44:3/FA18:2)+NH4	44/3	FA18:2	0.006	100.000
5	TAG(46:1/FA18:1)+NH4	46/1	FA18:1	0.087	100.000
6	TAG(46:2/FA16:0)+NH4	46/2	FA16:0	0.089	56.669
7	TAG(46:2/FA18:2)+NH4	46/2	FA18:2		43.331
8	TAG(46:3/FA14:0)+NH4	46/3	FA14:0	0.075	6.830
9	TAG(46:3/FA16:0)+NH4	46/3	FA16:0		19.685
10	TAG(46:3/FA18:1)+NH4	46/3	FA18:1		30.827
11	TAG(46:3/FA18:2)+NH4	46/3	FA18:2		30.297
12	TAG(46:3/FA18:3)+NH4	46/3	FA18:3		12.362
13	TAG(46:4/FA18:2)+NH4	46/4	FA18:2	0.009	100.000
14	TAG(48:0/FA14:0)+NH4	48/0	FA14:0	0.151	47.928
15	TAG(48:0/FA18:0)+NH4	48/0	FA18:0		52.072
16	TAG(48:1/FA14:0)+NH4	48/1	FA14:0	1.558	24.158
17	TAG(48:1/FA16:0)+NH4	48/1	FA16:0		41.308
18	TAG(48:1/FA18:0)+NH4	48/1	FA18:0		2.122
19	TAG(48:1/FA18:1)+NH4	48/1	FA18:1		32.412
20	TAG(48:2/FA14:0)+NH4	48/2	FA14:0	1.084	24.024
21	TAG(48:2/FA16:0)+NH4	48/2	FA16:0		29.366
22	TAG(48:2/FA18:0)+NH4	48/2	FA18:0		0.960
23	TAG(48:2/FA18:1)+NH4	48/2	FA18:1		22.371
24	TAG(48:2/FA18:2)+NH4	48/2	FA18:2		23.279
25	TAG(48:3/FA14:0)+NH4	48/3	FA14:0	0.327	15.966
26	TAG(48:3/FA16:0)+NH4	48/3	FA16:0		19.077
27	TAG(48:3/FA18:1)+NH4	48/3	FA18:1		19.457
28	TAG(48:3/FA18:2)+NH4	48/3	FA18:2		30.395

29	TAG(48:3/FA18:3)+NH4	48/3	FA18:3		15.105
30	TAG(48:4/FA14:0)+NH4	48/4	FA14:0	0.077	6.998
31	TAG(48:4/FA16:0)+NH4	48/4	FA16:0		9.757
32	TAG(48:4/FA18:1)+NH4	48/4	FA18:1		16.322
33	TAG(48:4/FA18:2)+NH4	48/4	FA18:2		31.981
34	TAG(48:4/FA18:3)+NH4	48/4	FA18:3		31.124
35	TAG(48:4/FA20:4)+NH4	48/4	FA20:4		3.817
36	TAG(48:5/FA18:2)+NH4	48/5	FA18:2	0.012	44.637
37	TAG(48:5/FA18:3)+NH4	48/5	FA18:3		55.363
38	TAG(49:3/FA16:0)+NH4	49/3	FA16:0	0.036	61.432
39	TAG(49:3/FA18:3)+NH4	49/3	FA18:3		38.568
40	TAG(50:0/FA16:0)+NH4	50/0	FA16:0	1.161	68.379
41	TAG(50:0/FA18:0)+NH4	50/0	FA18:0		31.621
42	TAG(50:1/FA14:0)+NH4	50/1	FA14:0	5.751	2.434
43	TAG(50:1/FA16:0)+NH4	50/1	FA16:0		57.504
44	TAG(50:1/FA18:0)+NH4	50/1	FA18:0		4.068
45	TAG(50:1/FA18:1)+NH4	50/1	FA18:1		35.994
46	TAG(50:2/FA14:0)+NH4	50/2	FA14:0	6.768	6.507
47	TAG(50:2/FA16:0)+NH4	50/2	FA16:0		35.770
48	TAG(50:2/FA16:1)+NH4	50/2	FA16:1		14.837
49	TAG(50:2/FA18:0)+NH4	50/2	FA18:0		0.936
50	TAG(50:2/FA18:1)+NH4	50/2	FA18:1		26.803
51	TAG(50:2/FA18:2)+NH4	50/2	FA18:2		15.147
52	TAG(50:3/FA14:0)+NH4	50/3	FA14:0	3.167	10.186
53	TAG(50:3/FA16:0)+NH4	50/3	FA16:0		21.371
54	TAG(50:3/FA16:1)+NH4	50/3	FA16:1		20.127
55	TAG(50:3/FA18:0)+NH4	50/3	FA18:0		0.351
56	TAG(50:3/FA18:1)+NH4	50/3	FA18:1		14.394
57	TAG(50:3/FA18:2)+NH4	50/3	FA18:2		26.877
58	TAG(50:3/FA18:3)+NH4	50/3	FA18:3		6.202
59	TAG(50:3/FA20:3)+NH4	50/3	FA20:3		0.491

60	TAG(50:4/FA14:0)+NH4	50/4	FA14:0	0.575	14.302
61	TAG(50:4/FA16:0)+NH4	50/4	FA16:0		9.709
62	TAG(50:4/FA16:1)+NH4	50/4	FA16:1		12.035
63	TAG(50:4/FA18:1)+NH4	50/4	FA18:1		7.695
64	TAG(50:4/FA18:2)+NH4	50/4	FA18:2		27.343
65	TAG(50:4/FA18:3)+NH4	50/4	FA18:3		25.172
66	TAG(50:4/FA20:3)+NH4	50/4	FA20:3		0.941
67	TAG(50:4/FA20:4)+NH4	50/4	FA20:4		2.803
68	TAG(50:5/FA14:0)+NH4	50/5	FA14:0	0.094	11.246
69	TAG(50:5/FA16:0)+NH4	50/5	FA16:0		6.589
70	TAG(50:5/FA16:1)+NH4	50/5	FA16:1		10.187
71	TAG(50:5/FA18:1)+NH4	50/5	FA18:1		7.433
72	TAG(50:5/FA18:2)+NH4	50/5	FA18:2		18.459
73	TAG(50:5/FA18:3)+NH4	50/5	FA18:3		37.754
74	TAG(50:5/FA20:4)+NH4	50/5	FA20:4		8.332
75	TAG(50:6/FA20:4)+NH4	50/6	FA20:4	0.003	100.000
76	TAG(51:2/FA16:0)+NH4	51/2	FA16:0	1.094	30.958
77	TAG(51:2/FA17:0)+NH4	51/2	FA17:0		16.032
78	TAG(51:2/FA18:1)+NH4	51/2	FA18:1		42.191
79	TAG(51:2/FA18:2)+NH4	51/2	FA18:2		10.819
80	TAG(51:3/FA17:0)+NH4	51/3	FA17:0	0.230	12.001
81	TAG(51:3/FA18:2)+NH4	51/3	FA18:2		79.157
82	TAG(51:3/FA18:3)+NH4	51/3	FA18:3		8.842
83	TAG(51:4/FA18:2)+NH4	51/4	FA18:2	0.121	58.062
84	TAG(51:4/FA18:3)+NH4	51/4	FA18:3		35.393
85	TAG(51:4/FA20:4)+NH4	51/4	FA20:4		6.545
86	TAG(51:5/FA18:2)+NH4	51/5	FA18:2	0.034	37.254
87	TAG(51:5/FA18:3)+NH4	51/5	FA18:3		62.746
88	TAG(52:0/FA16:0)+NH4	52/0	FA16:0	0.525	42.391
89	TAG(52:0/FA18:0)+NH4	52/0	FA18:0		57.609
90	TAG(52:1/FA16:0)+NH4	52/1	FA16:0	4.983	33.306

91	TAG(52:1/FA18:0)+NH4	52/1	FA18:0		23.761
92	TAG(52:1/FA18:1)+NH4	52/1	FA18:1		34.004
93	TAG(52:1/FA20:0)+NH4	52/1	FA20:0		4.785
94	TAG(52:1/FA20:1)+NH4	52/1	FA20:1		4.145
95	TAG(52:2/FA14:0)+NH4	52/2	FA14:0	15.578	0.631
96	TAG(52:2/FA16:0)+NH4	52/2	FA16:0		34.141
97	TAG(52:2/FA16:1)+NH4	52/2	FA16:1		1.625
98	TAG(52:2/FA18:0)+NH4	52/2	FA18:0		4.098
99	TAG(52:2/FA18:1)+NH4	52/2	FA18:1		52.369
100	TAG(52:2/FA18:2)+NH4	52/2	FA18:2		4.892
101	TAG(52:2/FA20:0)+NH4	52/2	FA20:0		0.357
102	TAG(52:2/FA20:1)+NH4	52/2	FA20:1		1.486
103	TAG(52:2/FA20:2)+NH4	52/2	FA20:2		0.399
104	TAG(52:3/FA14:0)+NH4	52/3	FA14:0	14.333	0.284
105	TAG(52:3/FA16:0)+NH4	52/3	FA16:0		29.239
106	TAG(52:3/FA16:1)+NH4	52/3	FA16:1		5.285
107	TAG(52:3/FA18:0)+NH4	52/3	FA18:0		0.681
108	TAG(52:3/FA18:1)+NH4	52/3	FA18:1		33.143
109	TAG(52:3/FA18:2)+NH4	52/3	FA18:2		29.074
110	TAG(52:3/FA18:3)+NH4	52/3	FA18:3		0.912
111	TAG(52:3/FA20:0)+NH4	52/3	FA20:0		0.144
112	TAG(52:3/FA20:1)+NH4	52/3	FA20:1		0.393
113	TAG(52:3/FA20:2)+NH4	52/3	FA20:2		0.420
114	TAG(52:3/FA20:3)+NH4	52/3	FA20:3		0.425
115	TAG(52:4/FA14:0)+NH4	52/4	FA14:0	6.340	0.250
116	TAG(52:4/FA16:0)+NH4	52/4	FA16:0		25.170
117	TAG(52:4/FA16:1)+NH4	52/4	FA16:1		7.794
118	TAG(52:4/FA18:0)+NH4	52/4	FA18:0		0.177
119	TAG(52:4/FA18:1)+NH4	52/4	FA18:1		12.061
120	TAG(52:4/FA18:2)+NH4	52/4	FA18:2		38.230
121	TAG(52:4/FA18:3)+NH4	52/4	FA18:3		13.367

122	TAG(52:4/FA20:0)+NH4	52/4	FA20:0		0.240
123	TAG(52:4/FA20:2)+NH4	52/4	FA20:2		0.232
124	TAG(52:4/FA20:3)+NH4	52/4	FA20:3		0.835
125	TAG(52:4/FA20:4)+NH4	52/4	FA20:4		1.496
126	TAG(52:4/FA22:4)+NH4	52/4	FA22:4		0.147
127	TAG(52:5/FA14:0)+NH4	52/5	FA14:0	1.460	0.551
128	TAG(52:5/FA16:0)+NH4	52/5	FA16:0		15.415
129	TAG(52:5/FA16:1)+NH4	52/5	FA16:1		11.889
130	TAG(52:5/FA18:1)+NH4	52/5	FA18:1		5.160
131	TAG(52:5/FA18:2)+NH4	52/5	FA18:2		24.674
132	TAG(52:5/FA18:3)+NH4	52/5	FA18:3		34.101
133	TAG(52:5/FA20:3)+NH4	52/5	FA20:3		0.771
134	TAG(52:5/FA20:4)+NH4	52/5	FA20:4		4.827
135	TAG(52:5/FA20:5)+NH4	52/5	FA20:5		1.860
136	TAG(52:5/FA22:5)+NH4	52/5	FA22:5		0.752
137	TAG(52:6/FA14:0)+NH4	52/6	FA14:0	0.246	1.712
138	TAG(52:6/FA16:0)+NH4	52/6	FA16:0		10.690
139	TAG(52:6/FA16:1)+NH4	52/6	FA16:1		10.855
140	TAG(52:6/FA18:1)+NH4	52/6	FA18:1		3.266
141	TAG(52:6/FA18:2)+NH4	52/6	FA18:2		13.551
142	TAG(52:6/FA18:3)+NH4	52/6	FA18:3		33.574
143	TAG(52:6/FA20:4)+NH4	52/6	FA20:4		6.281
144	TAG(52:6/FA20:5)+NH4	52/6	FA20:5		10.761
145	TAG(52:6/FA22:6)+NH4	52/6	FA22:6		9.309
146	TAG(52:7/FA18:1)+NH4	52/7	FA18:1	0.048	48.967
147	TAG(52:7/FA20:5)+NH4	52/7	FA20:5		20.096
148	TAG(52:7/FA22:6)+NH4	52/7	FA22:6		30.937
149	TAG(52:8/FA18:2)+NH4	52/8	FA18:2	0.010	100.000
150	TAG(53:0/FA16:0)+NH4	53/0	FA16:0	0.040	100.000
151	TAG(53:1/FA16:0)+NH4	53/1	FA16:0	0.499	29.266
152	TAG(53:1/FA17:0)+NH4	53/1	FA17:0		19.462

153	TAG(53:1/FA18:0)+NH4	53/1	FA18:0		20.395
154	TAG(53:1/FA18:1)+NH4	53/1	FA18:1		30.878
155	TAG(53:2/FA16:0)+NH4	53/2	FA16:0	1.329	25.099
156	TAG(53:2/FA17:0)+NH4	53/2	FA17:0		22.206
157	TAG(53:2/FA18:1)+NH4	53/2	FA18:1		48.359
158	TAG(53:2/FA18:2)+NH4	53/2	FA18:2		4.336
159	TAG(53:3/FA16:0)+NH4	53/3	FA16:0	0.701	40.155
160	TAG(53:3/FA17:0)+NH4	53/3	FA17:0		23.791
161	TAG(53:3/FA18:2)+NH4	53/3	FA18:2		36.054
162	TAG(53:4/FA16:0)+NH4	53/4	FA16:0	0.437	26.890
163	TAG(53:4/FA17:0)+NH4	53/4	FA17:0		12.034
164	TAG(53:4/FA18:2)+NH4	53/4	FA18:2		45.001
165	TAG(53:4/FA18:3)+NH4	53/4	FA18:3		13.989
166	TAG(53:4/FA20:4)+NH4	53/4	FA20:4		2.085
167	TAG(53:5/FA20:4)+NH4	53/5	FA20:4	0.015	100.000
168	TAG(53:6/FA20:4)+NH4	53/6	FA20:4	0.008	100.000
169	TAG(54:0/FA16:0)+NH4	54/0	FA16:0	0.170	36.806
170	TAG(54:0/FA18:0)+NH4	54/0	FA18:0		63.194
171	TAG(54:1/FA16:0)+NH4	54/1	FA16:0	2.212	15.871
172	TAG(54:1/FA18:0)+NH4	54/1	FA18:0		16.976
173	TAG(54:1/FA18:1)+NH4	54/1	FA18:1		15.360
174	TAG(54:1/FA20:0)+NH4	54/1	FA20:0		45.293
175	TAG(54:1/FA20:1)+NH4	54/1	FA20:1		6.501
176	TAG(54:2/FA16:0)+NH4	54/2	FA16:0	5.344	18.009
177	TAG(54:2/FA18:0)+NH4	54/2	FA18:0		16.017
178	TAG(54:2/FA18:1)+NH4	54/2	FA18:1		35.864
179	TAG(54:2/FA18:2)+NH4	54/2	FA18:2		2.478
180	TAG(54:2/FA20:0)+NH4	54/2	FA20:0		9.708
181	TAG(54:2/FA20:1)+NH4	54/2	FA20:1		17.288
182	TAG(54:2/FA20:2)+NH4	54/2	FA20:2		0.636
183	TAG(54:3/FA16:0)+NH4	54/3	FA16:0	6.470	8.425

184	TAG(54:3/FA16:1)+NH4	54/3	FA16:1		2.294
185	TAG(54:3/FA18:0)+NH4	54/3	FA18:0		8.866
186	TAG(54:3/FA18:1)+NH4	54/3	FA18:1		55.436
187	TAG(54:3/FA18:2)+NH4	54/3	FA18:2		12.205
188	TAG(54:3/FA18:3)+NH4	54/3	FA18:3		0.320
189	TAG(54:3/FA20:1)+NH4	54/3	FA20:1		8.166
190	TAG(54:3/FA20:2)+NH4	54/3	FA20:2		3.777
191	TAG(54:3/FA20:3)+NH4	54/3	FA20:3		0.512
192	TAG(54:4/FA16:0)+NH4	54/4	FA16:0	4.457	5.354
193	TAG(54:4/FA16:1)+NH4	54/4	FA16:1		1.588
194	TAG(54:4/FA18:0)+NH4	54/4	FA18:0		4.314
195	TAG(54:4/FA18:1)+NH4	54/4	FA18:1		44.390
196	TAG(54:4/FA18:2)+NH4	54/4	FA18:2		29.767
197	TAG(54:4/FA18:3)+NH4	54/4	FA18:3		3.464
198	TAG(54:4/FA20:1)+NH4	54/4	FA20:1		1.680
199	TAG(54:4/FA20:2)+NH4	54/4	FA20:2		3.106
200	TAG(54:4/FA20:3)+NH4	54/4	FA20:3		4.745
201	TAG(54:4/FA20:4)+NH4	54/4	FA20:4		1.135
202	TAG(54:4/FA22:4)+NH4	54/4	FA22:4		0.456
203	TAG(54:5/FA16:0)+NH4	54/5	FA16:0	2.547	5.441
204	TAG(54:5/FA16:1)+NH4	54/5	FA16:1		1.261
205	TAG(54:5/FA18:0)+NH4	54/5	FA18:0		1.137
206	TAG(54:5/FA18:1)+NH4	54/5	FA18:1		26.838
207	TAG(54:5/FA18:2)+NH4	54/5	FA18:2		32.707
208	TAG(54:5/FA18:3)+NH4	54/5	FA18:3		14.277
209	TAG(54:5/FA20:2)+NH4	54/5	FA20:2		0.817
210	TAG(54:5/FA20:3)+NH4	54/5	FA20:3		5.005
211	TAG(54:5/FA20:4)+NH4	54/5	FA20:4		11.266
212	TAG(54:5/FA20:5)+NH4	54/5	FA20:5		0.547
213	TAG(54:5/FA22:4)+NH4	54/5	FA22:4		0.704
214	TAG(54:6/FA16:0)+NH4	54/6	FA16:0	1.151	5.094

215	TAG(54:6/FA16:1)+NH4	54/6	FA16:1		1.674
216	TAG(54:6/FA18:1)+NH4	54/6	FA18:1		12.360
217	TAG(54:6/FA18:2)+NH4	54/6	FA18:2		29.040
218	TAG(54:6/FA18:3)+NH4	54/6	FA18:3		18.680
219	TAG(54:6/FA20:3)+NH4	54/6	FA20:3		1.603
220	TAG(54:6/FA20:4)+NH4	54/6	FA20:4		15.874
221	TAG(54:6/FA20:5)+NH4	54/6	FA20:5		7.641
222	TAG(54:6/FA22:5)+NH4	54/6	FA22:5		1.664
223	TAG(54:6/FA22:6)+NH4	54/6	FA22:6		6.370
224	TAG(54:7/FA16:1)+NH4	54/7	FA16:1	0.377	2.341
225	TAG(54:7/FA18:1)+NH4	54/7	FA18:1		6.150
226	TAG(54:7/FA18:2)+NH4	54/7	FA18:2		17.967
227	TAG(54:7/FA18:3)+NH4	54/7	FA18:3		22.579
228	TAG(54:7/FA20:4)+NH4	54/7	FA20:4		7.980
229	TAG(54:7/FA20:5)+NH4	54/7	FA20:5		18.259
230	TAG(54:7/FA22:5)+NH4	54/7	FA22:5		2.518
231	TAG(54:7/FA22:6)+NH4	54/7	FA22:6		22.204
232	TAG(54:8/FA18:2)+NH4	54/8	FA18:2	0.080	14.076
233	TAG(54:8/FA18:3)+NH4	54/8	FA18:3		27.783
234	TAG(54:8/FA20:4)+NH4	54/8	FA20:4		5.891
235	TAG(54:8/FA20:5)+NH4	54/8	FA20:5		22.280
236	TAG(54:8/FA22:6)+NH4	54/8	FA22:6		29.970
237	TAG(55:1/FA16:0)+NH4	55/1	FA16:0	0.107	52.551
238	TAG(55:1/FA18:1)+NH4	55/1	FA18:1		47.449
239	TAG(55:2/FA18:1)+NH4	55/2	FA18:1	0.182	90.237
240	TAG(55:2/FA18:2)+NH4	55/2	FA18:2		9.763
241	TAG(55:3/FA18:1)+NH4	55/3	FA18:1	0.350	85.569
242	TAG(55:3/FA18:2)+NH4	55/3	FA18:2		14.431
243	TAG(55:4/FA18:1)+NH4	55/4	FA18:1	0.264	69.871
244	TAG(55:4/FA18:2)+NH4	55/4	FA18:2		30.129
245	TAG(55:5/FA18:1)+NH4	55/5	FA18:1	0.159	45.827

246	TAG(55:5/FA18:2)+NH4	55/5	FA18:2		44.558
247	TAG(55:5/FA20:4)+NH4	55/5	FA20:4		9.616
248	TAG(55:7/FA22:6)+NH4	55/7	FA22:6	0.023	100.000
249	TAG(56:10/FA18:2)+NH4	56/10	FA18:2	0.041	100.000
250	TAG(56:1/FA18:1)+NH4	56/1	FA18:1	0.082	100.000
251	TAG(56:2/FA16:0)+NH4	56/2	FA16:0	1.140	29.169
252	TAG(56:2/FA18:0)+NH4	56/2	FA18:0		13.151
253	TAG(56:2/FA20:0)+NH4	56/2	FA20:0		38.695
254	TAG(56:2/FA20:1)+NH4	56/2	FA20:1		18.985
255	TAG(56:3/FA18:0)+NH4	56/3	FA18:0	1.617	5.260
256	TAG(56:3/FA18:1)+NH4	56/3	FA18:1		42.908
257	TAG(56:3/FA18:2)+NH4	56/3	FA18:2		10.239
258	TAG(56:3/FA20:0)+NH4	56/3	FA20:0		12.252
259	TAG(56:3/FA20:1)+NH4	56/3	FA20:1		26.801
260	TAG(56:3/FA20:2)+NH4	56/3	FA20:2		2.540
261	TAG(56:4/FA16:0)+NH4	56/4	FA16:0	1.028	4.765
262	TAG(56:4/FA18:0)+NH4	56/4	FA18:0		3.682
263	TAG(56:4/FA18:1)+NH4	56/4	FA18:1		30.634
264	TAG(56:4/FA18:2)+NH4	56/4	FA18:2		21.888
265	TAG(56:4/FA20:1)+NH4	56/4	FA20:1		23.071
266	TAG(56:4/FA20:2)+NH4	56/4	FA20:2		10.139
267	TAG(56:4/FA20:3)+NH4	56/4	FA20:3		3.680
268	TAG(56:4/FA20:4)+NH4	56/4	FA20:4		1.241
269	TAG(56:4/FA22:4)+NH4	56/4	FA22:4		0.900
270	TAG(56:5/FA16:0)+NH4	56/5	FA16:0	0.733	7.525
271	TAG(56:5/FA18:0)+NH4	56/5	FA18:0		3.837
272	TAG(56:5/FA18:1)+NH4	56/5	FA18:1		20.883
273	TAG(56:5/FA18:2)+NH4	56/5	FA18:2		17.783
274	TAG(56:5/FA20:1)+NH4	56/5	FA20:1		12.286
275	TAG(56:5/FA20:2)+NH4	56/5	FA20:2		8.192
276	TAG(56:5/FA20:3)+NH4	56/5	FA20:3		11.039

277	TAG(56:5/FA20:4)+NH4	56/5	FA20:4		9.743
278	TAG(56:5/FA22:4)+NH4	56/5	FA22:4		8.711
279	TAG(56:6/FA16:0)+NH4	56/6	FA16:0	0.599	10.458
280	TAG(56:6/FA18:0)+NH4	56/6	FA18:0		2.689
281	TAG(56:6/FA18:1)+NH4	56/6	FA18:1		17.356
282	TAG(56:6/FA18:2)+NH4	56/6	FA18:2		11.149
283	TAG(56:6/FA18:3)+NH4	56/6	FA18:3		4.537
284	TAG(56:6/FA20:2)+NH4	56/6	FA20:2		3.604
285	TAG(56:6/FA20:3)+NH4	56/6	FA20:3		8.294
286	TAG(56:6/FA20:4)+NH4	56/6	FA20:4		23.300
287	TAG(56:6/FA20:5)+NH4	56/6	FA20:5		4.324
288	TAG(56:6/FA22:4)+NH4	56/6	FA22:4		6.515
289	TAG(56:6/FA22:6)+NH4	56/6	FA22:6		7.774
290	TAG(56:7/FA16:0)+NH4	56/7	FA16:0	0.647	7.882
291	TAG(56:7/FA18:0)+NH4	56/7	FA18:0		1.683
292	TAG(56:7/FA18:1)+NH4	56/7	FA18:1		7.853
293	TAG(56:7/FA18:2)+NH4	56/7	FA18:2		8.238
294	TAG(56:7/FA18:3)+NH4	56/7	FA18:3		2.810
295	TAG(56:7/FA20:3)+NH4	56/7	FA20:3		2.903
296	TAG(56:7/FA20:4)+NH4	56/7	FA20:4		14.395
297	TAG(56:7/FA20:5)+NH4	56/7	FA20:5		11.614
298	TAG(56:7/FA22:4)+NH4	56/7	FA22:4		0.288
299	TAG(56:7/FA22:5)+NH4	56/7	FA22:5		3.770
300	TAG(56:7/FA22:6)+NH4	56/7	FA22:6		38.563
301	TAG(56:8/FA16:0)+NH4	56/8	FA16:0	0.375	13.199
302	TAG(56:8/FA18:1)+NH4	56/8	FA18:1		12.287
303	TAG(56:8/FA18:2)+NH4	56/8	FA18:2		4.233
304	TAG(56:8/FA18:3)+NH4	56/8	FA18:3		3.573
305	TAG(56:8/FA20:4)+NH4	56/8	FA20:4		9.625
306	TAG(56:8/FA20:5)+NH4	56/8	FA20:5		13.171
307	TAG(56:8/FA22:5)+NH4	56/8	FA22:5		4.939

308	TAG(56:8/FA22:6)+NH4	56/8	FA22:6		38.973
309	TAG(56:9/FA18:3)+NH4	56/9	FA18:3	0.063	8.561
310	TAG(56:9/FA20:4)+NH4	56/9	FA20:4		11.336
311	TAG(56:9/FA20:5)+NH4	56/9	FA20:5		31.104
312	TAG(56:9/FA22:6)+NH4	56/9	FA22:6		48.999
313	TAG(57:2/FA18:1)+NH4	57/2	FA18:1	0.031	100.000
314	TAG(57:3/FA18:2)+NH4	57/3	FA18:2	0.004	100.000
315	TAG(58:10/FA20:5)+NH4	58/10	FA20:5	0.029	21.679
316	TAG(58:10/FA22:6)+NH4	58/10	FA22:6		78.321
317	TAG(58:2/FA18:1)+NH4	58/2	FA18:1	0.169	100.000
318	TAG(58:6/FA16:0)+NH4	58/6	FA16:0	0.073	16.956
319	TAG(58:6/FA18:1)+NH4	58/6	FA18:1		27.596
320	TAG(58:6/FA20:4)+NH4	58/6	FA20:4		16.690
321	TAG(58:6/FA22:4)+NH4	58/6	FA22:4		24.426
322	TAG(58:6/FA22:5)+NH4	58/6	FA22:5		14.332
323	TAG(58:7/FA16:0)+NH4	58/7	FA16:0	0.090	14.393
324	TAG(58:7/FA18:1)+NH4	58/7	FA18:1		14.705
325	TAG(58:7/FA18:2)+NH4	58/7	FA18:2		12.049
326	TAG(58:7/FA22:4)+NH4	58/7	FA22:4		13.986
327	TAG(58:7/FA22:6)+NH4	58/7	FA22:6		44.868
328	TAG(58:8/FA18:1)+NH4	58/8	FA18:1	0.134	16.031
329	TAG(58:8/FA18:2)+NH4	58/8	FA18:2		7.163
330	TAG(58:8/FA22:6)+NH4	58/8	FA22:6		76.806
331	TAG(58:9/FA18:1)+NH4	58/9	FA18:1	0.122	30.259
332	TAG(58:9/FA18:2)+NH4	58/9	FA18:2		9.306
333	TAG(58:9/FA20:4)+NH4	58/9	FA20:4		6.794
334	TAG(58:9/FA22:5)+NH4	58/9	FA22:5		13.123
335	TAG(58:9/FA22:6)+NH4	58/9	FA22:6		40.518
336	TAG(60:10/FA22:6)+NH4	60/10	FA22:6	0.006	100.000
337	TAG(60:11/FA22:5)+NH4	60/11	FA22:5	0.006	30.576
338	TAG(60:11/FA22:6)+NH4	60/11	FA22:6		69.424

339	TAG(60:12/FA22:6)+NH4	60/12	FA22:6	0.002	100.000
	Total			100.000	

Supplementary- Table S4: PL's species with their respective isomers.

S.No.	Lipids	FA composition (Total carbon/ un-saturation)	Isomers	% Species abundance	% Isomer abundance
1	PC(14:0/14:0)+AcO	28/0	FA14:0/FA14:0	0.014	42.311
2	PE(14:0/14:0)-H	28/0	FA14:0/FA14:0		57.689
3	PC(16:0/14:0)+AcO	30/0	FA16:0/FA14:0	0.192	100.000
4	PC(16:0/16:0)+AcO	32/0	FA16:0/FA16:0	3.009	88.527
5	PC(18:0/14:0)+AcO	32/0	FA18:0/FA14:0		0.827
6	PE(16:0/16:0)-H	32/0	FA16:0/FA16:0		6.486
7	PE(18:0/14:0)-H	32/0	FA18:0/FA14:0		0.338
8	PE(P-16:0/16:0)-H	32/0	FA16:0/FA16:0		1.354
9	PG(16:0/16:0)-H	32/0	FA16:0/FA16:0		0.950
10	PI(16:0/16:0)-H	32/0	FA16:0/FA16:0		1.319
11	PS(16:0/16:0)-H	32/0	FA16:0/FA16:0		0.199
12	PC(14:0/18:1)+AcO	32/1	FA14:0/FA18:1	0.838	15.111
13	PC(16:0/16:1)+AcO	32/1	FA16:0/FA16:1		67.340
14	PE(P-14:0/18:1)-H	32/1	FA14:0/FA18:1		0.613
15	PE(P-16:0/16:1)-H	32/1	FA16:0/FA16:1		3.186
16	PI(16:0/16:1)-H	32/1	FA16:0/FA16:1		1.674
17	PS(14:0/18:1)-H	32/1	FA14:0/FA18:1		12.076
18	PC(14:0/18:2)+AcO	32/2	FA14:0/FA18:2	0.210	62.248
19	PE(14:0/18:2)-H	32/2	FA14:0/FA18:2		6.335
20	PG(14:0/18:2)-H	32/2	FA14:0/FA18:2		2.846
21	PI(14:0/18:2)-H	32/2	FA14:0/FA18:2		10.714
22	PS(14:0/18:2)-H	32/2	FA14:0/FA18:2		17.857
23	PC(14:0/18:3)+AcO	32/3	FA14:0/FA18:3	0.014	100.000

24	PC(16:0/18:0)+AcO	34/0	FA16:0/FA18:0	1.757	56.019
25	PE(18:0/16:0)-H	34/0	FA18:0/FA16:0		5.373
26	PE(O-16:0/18:0)-H	34/0	FA16:0/FA18:0		0.957
27	PE(O-18:0/16:0)-H	34/0	FA18:0/FA16:0		16.845
28	PE(P-16:0/18:0)-H	34/0	FA16:0/FA18:0		1.507
29	PE(P-18:0/16:0)-H	34/0	FA18:0/FA16:0		14.758
30	PG(16:0/18:0)-H	34/0	FA16:0/FA18:0		2.112
31	PI(16:0/18:0)-H	34/0	FA16:0/FA18:0		1.856
32	PS(16:0/18:0)-H	34/0	FA16:0/FA18:0		0.573
33	PC(16:0/18:1)+AcO	34/1	FA16:0/FA18:1	15.393	42.674
34	PC(18:0/16:1)+AcO	34/1	FA18:0/FA16:1		0.392
35	PE(16:0/18:1)-H	34/1	FA16:0/FA18:1		10.772
36	PE(O-16:0/18:1)-H	34/1	FA16:0/FA18:1		0.888
37	PE(O-18:0/16:1)-H	34/1	FA18:0/FA16:1		0.854
38	PE(P-16:0/18:1)-H	34/1	FA16:0/FA18:1		5.644
39	PE(P-18:0/16:1)-H	34/1	FA18:0/FA16:1		0.596
40	PE(P-18:1/16:0)-H	34/1	FA18:1/FA16:0		1.241
41	PG(16:0/18:1)-H	34/1	FA16:0/FA18:1		36.156
42	PI(16:0/18:1)-H	34/1	FA16:0/FA18:1		0.561
43	PI(18:0/16:1)-H	34/1	FA18:0/FA16:1		0.089
44	PS(14:0/20:1)-H	34/1	FA14:0/FA20:1		0.019
45	PS(16:0/18:1)-H	34/1	FA16:0/FA18:1		0.027
46	PA(18:0/16:1)-H	34/1	FA18:0/FA16:1		0.087
47	PC(14:0/20:2)+AcO	34/2	FA14:0/FA20:2	13.372	0.044
48	PC(16:0/18:2)+AcO	34/2	FA16:0/FA18:2		66.423
49	PC(16:1/18:1)+AcO	34/2	FA16:1/FA18:1		1.191
50	PC(18:1/16:1)+AcO	34/2	FA18:1/FA16:1		1.289
51	PE(16:0/18:2)-H	34/2	FA16:0/FA18:2		5.239
52	PE(O-16:0/18:2)-H	34/2	FA16:0/FA18:2		1.130
53	PE(P-16:0/18:2)-H	34/2	FA16:0/FA18:2		11.708
54	PE(P-16:1/18:1)-H	34/2	FA16:1/FA18:1		0.111

55	PE(P-18:1/16:1)-H	34/2	FA18:1/FA16:1		0.188
56	PG(16:0/18:2)-H	34/2	FA16:0/FA18:2		0.511
57	PG(18:1/16:1)-H	34/2	FA18:1/FA16:1		11.251
58	PI(16:0/18:2)-H	34/2	FA16:0/FA18:2		0.752
59	PS(16:0/18:2)-H	34/2	FA16:0/FA18:2		0.017
60	PA(16:0/18:2)-H	34/2	FA16:0/FA18:2		0.146
61	PC(14:0/20:3)+AcO	34/3	FA14:0/FA20:3		0.443
62	PC(16:0/18:3)+AcO	34/3	FA16:0/FA18:3		7.214
63	PC(16:1/18:2)+AcO	34/3	FA16:1/FA18:2		2.014
64	PC(18:2/16:1)+AcO	34/3	FA18:2/FA16:1		7.491
65	PE(14:0/20:3)-H	34/3	FA14:0/FA20:3		0.053
66	PE(16:0/18:3)-H	34/3	FA16:0/FA18:3		0.736
67	PE(18:2/16:1)-H	34/3	FA18:2/FA16:1		7.890
68	PE(O-16:0/18:3)-H	34/3	FA16:0/FA18:3	3.270	0.142
69	PE(P-16:0/18:3)-H	34/3	FA16:0/FA18:3		0.480
70	PG(16:0/18:3)-H	34/3	FA16:0/FA18:3		0.673
71	PG(18:2/16:1)-H	34/3	FA18:2/FA16:1		69.844
72	PI(14:0/20:3)-H	34/3	FA14:0/FA20:3		0.165
73	PI(16:0/18:3)-H	34/3	FA16:0/FA18:3		1.131
74	PS(16:0/18:3)-H	34/3	FA16:0/FA18:3		0.183
75	PS(18:2/16:1)-H	34/3	FA18:2/FA16:1		1.542
76	PC(14:0/20:4)+AcO	34/4	FA14:0/FA20:4		16.576
77	PE(14:0/20:4)-H	34/4	FA14:0/FA20:4		9.641
78	PG(14:0/20:4)-H	34/4	FA14:0/FA20:4	0.037	8.849
79	PI(14:0/20:4)-H	34/4	FA14:0/FA20:4		23.224
80	PS(14:0/20:4)-H	34/4	FA14:0/FA20:4		41.710
81	PC(14:0/20:5)+AcO	34/5	FA14:0/FA20:5	0.005	100.000
82	PC(18:0/18:0)+AcO	36/0	FA18:0/FA18:0		43.648
83	PE(18:0/18:0)-H	36/0	FA18:0/FA18:0	0.514	32.131
84	PE(O-18:0/18:0)-H	36/0	FA18:0/FA18:0		5.367
85	PE(P-18:0/18:0)-H	36/0	FA18:0/FA18:0		8.183

86	PG(18:0/18:0)-H	36/0	FA18:0/FA18:0		1.003
87	PI(18:0/18:0)-H	36/0	FA18:0/FA18:0		3.508
88	PS(18:0/18:0)-H	36/0	FA18:0/FA18:0		6.160
89	PC(16:0/20:1)+AcO	36/1	FA16:0/FA20:1		1.343
90	PC(18:0/18:1)+AcO	36/1	FA18:0/FA18:1		20.049
91	PE(16:0/20:1)-H	36/1	FA16:0/FA20:1		0.326
92	PE(18:0/18:1)-H	36/1	FA18:0/FA18:1		53.674
93	PE(O-16:0/20:1)-H	36/1	FA16:0/FA20:1		0.177
94	PE(P-16:0/20:1)-H	36/1	FA16:0/FA20:1		0.699
95	PE(P-18:0/18:1)-H	36/1	FA18:0/FA18:1	9.909	13.664
96	PE(P-18:1/18:0)-H	36/1	FA18:1/FA18:0		0.155
97	PG(18:0/18:1)-H	36/1	FA18:0/FA18:1		5.843
98	PI(18:0/18:1)-H	36/1	FA18:0/FA18:1		1.155
99	PI(20:0/16:1)-H	36/1	FA20:0/FA16:1		0.013
100	PA(18:0/18:1)-H	36/1	FA18:0/FA18:1		2.617
101	PA(20:0/16:1)-H	36/1	FA20:0/FA16:1		0.283
102	PC(16:0/20:2)+AcO	36/2	FA16:0/FA20:2		1.520
103	PC(18:0/18:2)+AcO	36/2	FA18:0/FA18:2		27.316
104	PC(18:1/18:1)+AcO	36/2	FA18:1/FA18:1		4.974
105	PE(16:0/20:2)-H	36/2	FA16:0/FA20:2		0.095
106	PE(18:0/18:2)-H	36/2	FA18:0/FA18:2		35.266
107	PE(18:1/18:1)-H	36/2	FA18:1/FA18:1		7.726
108	PE(O-16:0/20:2)-H	36/2	FA16:0/FA20:2		0.025
109	PE(O-18:0/18:2)-H	36/2	FA18:0/FA18:2	21.481	3.552
110	PE(P-16:0/20:2)-H	36/2	FA16:0/FA20:2		0.161
111	PE(P-18:0/18:2)-H	36/2	FA18:0/FA18:2		10.953
112	PE(P-18:1/18:1)-H	36/2	FA18:1/FA18:1		1.921
113	PG(16:0/20:2)-H	36/2	FA16:0/FA20:2		0.058
114	PG(18:0/18:2)-H	36/2	FA18:0/FA18:2		0.405
115	PG(18:1/18:1)-H	36/2	FA18:1/FA18:1		0.883
116	PI(16:0/20:2)-H	36/2	FA16:0/FA20:2		0.028

117	PI(18:0/18:2)-H	36/2	FA18:0/FA18:2		1.322
118	PI(18:1/18:1)-H	36/2	FA18:1/FA18:1		0.479
119	PS(16:0/20:2)-H	36/2	FA16:0/FA20:2		0.005
120	PS(18:0/18:2)-H	36/2	FA18:0/FA18:2		0.198
121	PS(18:1/18:1)-H	36/2	FA18:1/FA18:1		0.227
122	PA(18:0/18:2)-H	36/2	FA18:0/FA18:2		2.054
123	PA(18:1/18:1)-H	36/2	FA18:1/FA18:1		0.833
124	PC(16:0/20:3)+AcO	36/3	FA16:0/FA20:3		29.438
125	PC(18:0/18:3)+AcO	36/3	FA18:0/FA18:3		1.808
126	PC(18:1/18:2)+AcO	36/3	FA18:1/FA18:2		27.214
127	PE(16:0/20:3)-H	36/3	FA16:0/FA20:3		1.436
128	PE(18:0/18:3)-H	36/3	FA18:0/FA18:3		2.956
129	PE(18:1/18:2)-H	36/3	FA18:1/FA18:2		7.462
130	PE(O-16:0/20:3)-H	36/3	FA16:0/FA20:3		0.506
131	PE(O-18:0/18:3)-H	36/3	FA18:0/FA18:3		0.516
132	PE(P-16:0/20:3)-H	36/3	FA16:0/FA20:3		4.564
133	PE(P-18:0/18:3)-H	36/3	FA18:0/FA18:3	6.321	0.581
134	PE(P-18:1/18:2)-H	36/3	FA18:1/FA18:2		12.147
135	PG(16:0/20:3)-H	36/3	FA16:0/FA20:3		0.109
136	PG(18:0/18:3)-H	36/3	FA18:0/FA18:3		0.029
137	PG(18:1/18:2)-H	36/3	FA18:1/FA18:2		9.902
138	PI(18:1/18:2)-H	36/3	FA18:1/FA18:2		0.684
139	PS(16:0/20:3)-H	36/3	FA16:0/FA20:3		0.034
140	PS(18:0/18:3)-H	36/3	FA18:0/FA18:3		0.167
141	PS(18:1/18:2)-H	36/3	FA18:1/FA18:2		0.243
142	PA(18:0/18:3)-H	36/3	FA18:0/FA18:3		0.117
143	PA(18:1/18:2)-H	36/3	FA18:1/FA18:2		0.087
144	PC(14:0/22:4)+AcO	36/4	FA14:0/FA22:4		0.044
145	PC(16:0/20:4)+AcO	36/4	FA16:0/FA20:4	4.787	51.704
146	PC(18:1/18:3)+AcO	36/4	FA18:1/FA18:3		0.663
147	PC(18:2/18:2)+AcO	36/4	FA18:2/FA18:2		13.853

148	PE(14:0/22:4)-H	36/4	FA14:0/FA22:4		0.026
149	PE(16:0/20:4)-H	36/4	FA16:0/FA20:4		8.667
150	PE(18:1/18:3)-H	36/4	FA18:1/FA18:3		0.263
151	PE(18:2/18:2)-H	36/4	FA18:2/FA18:2		1.865
152	PE(O-16:0/20:4)-H	36/4	FA16:0/FA20:4		2.909
153	PE(P-16:0/20:4)-H	36/4	FA16:0/FA20:4		9.119
154	PE(P-18:2/18:2)-H	36/4	FA18:2/FA18:2		1.459
155	PG(16:0/20:4)-H	36/4	FA16:0/FA20:4		0.463
156	PG(18:1/18:3)-H	36/4	FA18:1/FA18:3		0.234
157	PG(18:2/18:2)-H	36/4	FA18:2/FA18:2		5.067
158	PI(14:0/22:4)-H	36/4	FA14:0/FA22:4		0.050
159	PI(16:0/20:4)-H	36/4	FA16:0/FA20:4		1.416
160	PI(18:2/18:2)-H	36/4	FA18:2/FA18:2		1.046
161	PS(16:0/20:4)-H	36/4	FA16:0/FA20:4		0.115
162	PS(18:1/18:3)-H	36/4	FA18:1/FA18:3		0.100
163	PS(18:2/18:2)-H	36/4	FA18:2/FA18:2		0.857
164	PA(16:0/20:4)-H	36/4	FA16:0/FA20:4		0.080
165	PC(14:0/22:5)+AcO	36/5	FA14:0/FA22:5		1.609
166	PC(16:0/20:5)+AcO	36/5	FA16:0/FA20:5		69.004
167	PC(18:2/18:3)+AcO	36/5	FA18:2/FA18:3		6.771
168	PE(14:0/22:5)-H	36/5	FA14:0/FA22:5		1.145
169	PE(16:0/20:5)-H	36/5	FA16:0/FA20:5		7.928
170	PE(O-16:0/20:5)-H	36/5	FA16:0/FA20:5	0.169	1.788
171	PG(14:0/22:5)-H	36/5	FA14:0/FA22:5		1.754
172	PG(16:0/20:5)-H	36/5	FA16:0/FA20:5		3.326
173	PG(18:2/18:3)-H	36/5	FA18:2/FA18:3		3.063
174	PS(16:0/20:5)-H	36/5	FA16:0/FA20:5		2.578
175	PS(18:2/18:3)-H	36/5	FA18:2/FA18:3		1.035
176	PC(14:0/22:6)+AcO	36/6	FA14:0/FA22:6	0.003	57.330
177	PE(14:0/22:6)-H	36/6	FA14:0/FA22:6		42.670
178	PC(18:0/20:0)+AcO	38/0	FA18:0/FA20:0	0.294	56.148

179	PG(18:0/20:0)-H	38/0	FA18:0/FA20:0		4.588
180	PI(18:0/20:0)-H	38/0	FA18:0/FA20:0		23.216
181	PS(18:0/20:0)-H	38/0	FA18:0/FA20:0		9.042
182	PA(18:0/20:0)-H	38/0	FA18:0/FA20:0		7.007
183	PC(18:0/20:1)+AcO	38/1	FA18:0/FA20:1	0.773	6.352
184	PC(20:0/18:1)+AcO	38/1	FA20:0/FA18:1		11.342
185	PE(18:0/20:1)-H	38/1	FA18:0/FA20:1		16.977
186	PE(O-18:0/20:1)-H	38/1	FA18:0/FA20:1		7.249
187	PE(P-18:0/20:1)-H	38/1	FA18:0/FA20:1		7.095
188	PG(18:0/20:1)-H	38/1	FA18:0/FA20:1		0.380
189	PG(20:0/18:1)-H	38/1	FA20:0/FA18:1		7.727
190	PI(20:0/18:1)-H	38/1	FA20:0/FA18:1		1.531
191	PS(18:0/20:1)-H	38/1	FA18:0/FA20:1		0.152
192	PA(18:0/20:1)-H	38/1	FA18:0/FA20:1		2.318
193	PA(20:0/18:1)-H	38/1	FA20:0/FA18:1		38.875
194	PC(18:0/20:2)+AcO	38/2	FA18:0/FA20:2	1.446	15.510
195	PC(18:1/20:1)+AcO	38/2	FA18:1/FA20:1		3.491
196	PE(18:0/20:2)-H	38/2	FA18:0/FA20:2		7.110
197	PE(18:1/20:1)-H	38/2	FA18:1/FA20:1		3.572
198	PE(O-18:0/20:2)-H	38/2	FA18:0/FA20:2		3.861
199	PE(P-18:0/20:2)-H	38/2	FA18:0/FA20:2		3.223
200	PE(P-18:1/20:1)-H	38/2	FA18:1/FA20:1		0.286
201	PG(18:0/20:2)-H	38/2	FA18:0/FA20:2		0.721
202	PG(18:1/20:1)-H	38/2	FA18:1/FA20:1		0.550
203	PG(20:0/18:2)-H	38/2	FA20:0/FA18:2		5.763
204	PI(18:0/20:2)-H	38/2	FA18:0/FA20:2		1.214
205	PI(20:0/18:2)-H	38/2	FA20:0/FA18:2		0.186
206	PS(20:0/18:2)-H	38/2	FA20:0/FA18:2		3.029
207	PA(20:0/18:2)-H	38/2	FA20:0/FA18:2		51.483
208	PC(18:0/20:3)+AcO	38/3	FA18:0/FA20:3	3.167	46.006
209	PC(18:1/20:2)+AcO	38/3	FA18:1/FA20:2		2.136

210	PC(18:2/20:1)+AcO	38/3	FA18:2/FA20:1		2.482
211	PC(20:0/18:3)+AcO	38/3	FA20:0/FA18:3		0.080
212	PE(18:0/20:3)-H	38/3	FA18:0/FA20:3		22.782
213	PE(18:1/20:2)-H	38/3	FA18:1/FA20:2		0.824
214	PE(18:2/20:1)-H	38/3	FA18:2/FA20:1		0.595
215	PE(O-18:0/20:3)-H	38/3	FA18:0/FA20:3		7.321
216	PE(P-18:0/20:3)-H	38/3	FA18:0/FA20:3		9.893
217	PE(P-18:1/20:2)-H	38/3	FA18:1/FA20:2		0.394
218	PG(18:0/20:3)-H	38/3	FA18:0/FA20:3		0.138
219	PG(18:1/20:2)-H	38/3	FA18:1/FA20:2		0.946
220	PG(18:2/20:1)-H	38/3	FA18:2/FA20:1		0.194
221	PI(18:0/20:3)-H	38/3	FA18:0/FA20:3		4.771
222	PI(18:1/20:2)-H	38/3	FA18:1/FA20:2		0.063
223	PS(18:0/20:3)-H	38/3	FA18:0/FA20:3		0.250
224	PS(18:1/20:2)-H	38/3	FA18:1/FA20:2		0.090
225	PS(18:2/20:1)-H	38/3	FA18:2/FA20:1		0.131
226	PS(20:0/18:3)-H	38/3	FA20:0/FA18:3		0.117
227	PA(18:0/20:3)-H	38/3	FA18:0/FA20:3		0.397
228	PA(20:0/18:3)-H	38/3	FA20:0/FA18:3		0.390
229	PC(16:0/22:4)+AcO	38/4	FA16:0/FA22:4	7.963	2.054
230	PC(18:0/20:4)+AcO	38/4	FA18:0/FA20:4		27.722
231	PC(18:1/20:3)+AcO	38/4	FA18:1/FA20:3		3.424
232	PC(18:2/20:2)+AcO	38/4	FA18:2/FA20:2		0.398
233	PE(16:0/22:4)-H	38/4	FA16:0/FA22:4		0.895
234	PE(18:0/20:4)-H	38/4	FA18:0/FA20:4		32.138
235	PE(18:1/20:3)-H	38/4	FA18:1/FA20:3		1.263
236	PE(18:2/20:2)-H	38/4	FA18:2/FA20:2		0.070
237	PE(O-16:0/22:4)-H	38/4	FA16:0/FA22:4		0.736
238	PE(O-18:0/20:4)-H	38/4	FA18:0/FA20:4		4.534
239	PE(P-16:0/22:4)-H	38/4	FA16:0/FA22:4		3.226
240	PE(P-18:0/20:4)-H	38/4	FA18:0/FA20:4		11.362

241	PE(P-18:1/20:3)-H	38/4	FA18:1/FA20:3		1.313
242	PG(18:0/20:4)-H	38/4	FA18:0/FA20:4		0.077
243	PG(18:1/20:3)-H	38/4	FA18:1/FA20:3		1.216
244	PG(18:2/20:2)-H	38/4	FA18:2/FA20:2		0.091
245	PI(16:0/22:4)-H	38/4	FA16:0/FA22:4		0.043
246	PI(18:0/20:4)-H	38/4	FA18:0/FA20:4		8.182
247	PI(18:1/20:3)-H	38/4	FA18:1/FA20:3		0.107
248	PS(18:0/20:4)-H	38/4	FA18:0/FA20:4		0.079
249	PS(18:1/20:3)-H	38/4	FA18:1/FA20:3		0.086
250	PS(18:2/20:2)-H	38/4	FA18:2/FA20:2		0.020
251	PA(18:0/20:4)-H	38/4	FA18:0/FA20:4		0.502
252	PA(18:1/20:3)-H	38/4	FA18:1/FA20:3		0.459
253	PC(16:0/22:5)+AcO	38/5	FA16:0/FA22:5	2.089	16.386
254	PC(18:0/20:5)+AcO	38/5	FA18:0/FA20:5		5.332
255	PC(18:1/20:4)+AcO	38/5	FA18:1/FA20:4		18.291
256	PC(18:2/20:3)+AcO	38/5	FA18:2/FA20:3		2.772
257	PE(16:0/22:5)-H	38/5	FA16:0/FA22:5		5.107
258	PE(18:0/20:5)-H	38/5	FA18:0/FA20:5		6.102
259	PE(18:1/20:4)-H	38/5	FA18:1/FA20:4		9.624
260	PE(18:2/20:3)-H	38/5	FA18:2/FA20:3		0.434
261	PE(O-18:0/20:5)-H	38/5	FA18:0/FA20:5		1.836
262	PE(P-16:0/22:5)-H	38/5	FA16:0/FA22:5		8.001
263	PE(P-18:0/20:5)-H	38/5	FA18:0/FA20:5		0.596
264	PE(P-18:1/20:4)-H	38/5	FA18:1/FA20:4		14.435
265	PG(16:0/22:5)-H	38/5	FA16:0/FA22:5		0.207
266	PG(18:1/20:4)-H	38/5	FA18:1/FA20:4		6.104
267	PG(18:2/20:3)-H	38/5	FA18:2/FA20:3		0.790
268	PI(16:0/22:5)-H	38/5	FA16:0/FA22:5		0.283
269	PI(18:2/20:3)-H	38/5	FA18:2/FA20:3		0.165
270	PS(18:1/20:4)-H	38/5	FA18:1/FA20:4		0.697
271	PS(18:2/20:3)-H	38/5	FA18:2/FA20:3		0.681

272	PA(18:0/20:5)-H	38/5	FA18:0/FA20:5		0.320
273	PA(18:1/20:4)-H	38/5	FA18:1/FA20:4		1.838
274	PC(16:0/22:6)+AcO	38/6	FA16:0/FA22:6		38.638
275	PC(18:1/20:5)+AcO	38/6	FA18:1/FA20:5		3.707
276	PC(18:2/20:4)+AcO	38/6	FA18:2/FA20:4		17.085
277	PE(16:0/22:6)-H	38/6	FA16:0/FA22:6		8.762
278	PE(18:1/20:5)-H	38/6	FA18:1/FA20:5		3.908
279	PE(18:2/20:4)-H	38/6	FA18:2/FA20:4		0.893
280	PE(O-16:0/22:6)-H	38/6	FA16:0/FA22:6		2.774
281	PE(P-16:0/22:6)-H	38/6	FA16:0/FA22:6	0.528	3.114
282	PE(P-18:1/20:5)-H	38/6	FA18:1/FA20:5		1.719
283	PE(P-18:2/20:4)-H	38/6	FA18:2/FA20:4		3.495
284	PG(18:1/20:5)-H	38/6	FA18:1/FA20:5		2.288
285	PG(18:2/20:4)-H	38/6	FA18:2/FA20:4		8.084
286	PI(18:2/20:4)-H	38/6	FA18:2/FA20:4		0.701
287	PS(18:2/20:4)-H	38/6	FA18:2/FA20:4		4.622
288	PA(18:2/20:4)-H	38/6	FA18:2/FA20:4		0.210
289	PC(18:2/20:5)+AcO	38/7	FA18:2/FA20:5	0.007	75.280
290	PE(18:2/20:5)-H	38/7	FA18:2/FA20:5		24.720
291	PC(20:0/20:1)+AcO	40/1	FA20:0/FA20:1		14.321
292	PS(20:0/20:1)-H	40/1	FA20:0/FA20:1	0.021	14.924
293	PA(20:0/20:1)-H	40/1	FA20:0/FA20:1		70.754
294	PG(20:0/20:2)-H	40/2	FA20:0/FA20:2		18.391
295	PS(20:0/20:2)-H	40/2	FA20:0/FA20:2	0.022	20.580
296	PA(20:0/20:2)-H	40/2	FA20:0/FA20:2		61.029
297	PC(20:0/20:3)+AcO	40/3	FA20:0/FA20:3		8.830
298	PG(20:0/20:3)-H	40/3	FA20:0/FA20:3	0.156	18.965
299	PS(20:0/20:3)-H	40/3	FA20:0/FA20:3		4.362
300	PA(20:0/20:3)-H	40/3	FA20:0/FA20:3		67.843
301	PC(18:0/22:4)+AcO	40/4	FA18:0/FA22:4	0.896	13.390
302	PC(20:0/20:4)+AcO	40/4	FA20:0/FA20:4		4.062

303	PE(18:0/22:4)-H	40/4	FA18:0/FA22:4		7.394
304	PE(O-18:0/22:4)-H	40/4	FA18:0/FA22:4		6.549
305	PE(P-18:0/22:4)-H	40/4	FA18:0/FA22:4		20.628
306	PG(20:0/20:4)-H	40/4	FA20:0/FA20:4		3.630
307	PI(18:0/22:4)-H	40/4	FA18:0/FA22:4		0.624
308	PI(20:0/20:4)-H	40/4	FA20:0/FA20:4		1.328
309	PS(18:0/22:4)-H	40/4	FA18:0/FA22:4		0.105
310	PS(20:0/20:4)-H	40/4	FA20:0/FA20:4		6.094
311	PA(18:0/22:4)-H	40/4	FA18:0/FA22:4		0.901
312	PA(20:0/20:4)-H	40/4	FA20:0/FA20:4		35.295
313	PC(18:0/22:5)+AcO	40/5	FA18:0/FA22:5		30.249
314	PC(18:1/22:4)+AcO	40/5	FA18:1/FA22:4		2.051
315	PC(20:0/20:5)+AcO	40/5	FA20:0/FA20:5		0.355
316	PE(18:0/22:5)-H	40/5	FA18:0/FA22:5		31.745
317	PE(18:1/22:4)-H	40/5	FA18:1/FA22:4		1.901
318	PE(O-18:0/22:5)-H	40/5	FA18:0/FA22:5		5.575
319	PE(P-18:0/22:5)-H	40/5	FA18:0/FA22:5		16.925
320	PE(P-18:1/22:4)-H	40/5	FA18:1/FA22:4	0.756	5.021
321	PG(18:1/22:4)-H	40/5	FA18:1/FA22:4		2.107
322	PG(20:0/20:5)-H	40/5	FA20:0/FA20:5		0.414
323	PI(18:0/22:5)-H	40/5	FA18:0/FA22:5		1.125
324	PS(18:0/22:5)-H	40/5	FA18:0/FA22:5		0.187
325	PS(18:1/22:4)-H	40/5	FA18:1/FA22:4		0.228
326	PA(18:0/22:5)-H	40/5	FA18:0/FA22:5		0.135
327	PA(18:1/22:4)-H	40/5	FA18:1/FA22:4		0.519
328	PA(20:0/20:5)-H	40/5	FA20:0/FA20:5		1.463
329	PC(18:0/22:6)+AcO	40/6	FA18:0/FA22:6		34.868
330	PC(18:1/22:5)+AcO	40/6	FA18:1/FA22:5		7.726
331	PC(18:2/22:4)+AcO	40/6	FA18:2/FA22:4	0.377	0.981
332	PE(18:0/22:6)-H	40/6	FA18:0/FA22:6		25.019
333	PE(18:1/22:5)-H	40/6	FA18:1/FA22:5		4.788

334	PE(18:2/22:4)-H	40/6	FA18:2/FA22:4		0.882
335	PE(O-18:0/22:6)-H	40/6	FA18:0/FA22:6		2.125
336	PE(P-18:0/22:6)-H	40/6	FA18:0/FA22:6		1.300
337	PE(P-18:1/22:5)-H	40/6	FA18:1/FA22:5		7.090
338	PG(18:1/22:5)-H	40/6	FA18:1/FA22:5		6.288
339	PI(18:0/22:6)-H	40/6	FA18:0/FA22:6		0.706
340	PS(18:1/22:5)-H	40/6	FA18:1/FA22:5		1.842
341	PS(18:2/22:4)-H	40/6	FA18:2/FA22:4		1.556
342	PA(18:0/22:6)-H	40/6	FA18:0/FA22:6		3.324
343	PA(18:1/22:5)-H	40/6	FA18:1/FA22:5		1.504
344	PC(18:1/22:6)+AcO	40/7	FA18:1/FA22:6	0.056	22.103
345	PC(18:2/22:5)+AcO	40/7	FA18:2/FA22:5		9.360
346	PE(18:1/22:6)-H	40/7	FA18:1/FA22:6		10.489
347	PE(P-18:1/22:6)-H	40/7	FA18:1/FA22:6		18.266
348	PG(18:1/22:6)-H	40/7	FA18:1/FA22:6		14.437
349	PG(18:2/22:5)-H	40/7	FA18:2/FA22:5		6.533
350	PS(18:1/22:6)-H	40/7	FA18:1/FA22:6		5.980
351	PS(18:2/22:5)-H	40/7	FA18:2/FA22:5		12.831
352	PC(18:2/22:6)+AcO	40/8	FA18:2/FA22:6	0.007	27.017
353	PE(P-18:2/22:6)-H	40/8	FA18:2/FA22:6		24.248
354	PS(18:2/22:6)-H	40/8	FA18:2/FA22:6		48.735
355	PC(20:0/22:4)+AcO	42/4	FA20:0/FA22:4	0.068	2.819
356	PG(20:0/22:4)-H	42/4	FA20:0/FA22:4		7.547
357	PS(20:0/22:4)-H	42/4	FA20:0/FA22:4		5.433
358	PA(20:0/22:4)-H	42/4	FA20:0/FA22:4		84.200
359	PG(20:0/22:5)-H	42/5	FA20:0/FA22:5	0.061	10.398
360	PS(20:0/22:5)-H	42/5	FA20:0/FA22:5		11.319
361	PA(20:0/22:5)-H	42/5	FA20:0/FA22:5		78.283
362	PC(20:0/22:6)+AcO	42/6	FA20:0/FA22:6	0.016	10.721
363	PG(20:0/22:6)-H	42/6	FA20:0/FA22:6		14.591
364	PS(20:0/22:6)-H	42/6	FA20:0/FA22:6		19.310

365	PA(20:0/22:6)-H	42/6	FA20:0/FA22:6		55.377
	Total			100	

Supplementary-Table S5: The list of the samples with their vitamin B12 values, age and sex.

S.No.	Samples	Vitamin B12 (pg/ml)	Age	Sex	S.No.	Samples	Vitamin B12 (pg/ml)	Age	Sex
1	B12 deficient_1	47.1544715	40	F	48	Control_1	400.2	18	M
2	B12 deficient_2	58.7262873	34	F	49	Control_2	404.336043	38	M
3	B12 deficient_3	59.3224932	54	F	50	Control_3	404.471545	50	F
4	B12 deficient_4	65.4065041	28	F	51	Control_4	404.471545	54	M
5	B12 deficient_5	71.5176152	40	F	52	Control_5	405.284553	37	M
6	B12 deficient_6	75.0542005	34	F	53	Control_6	405.826558	39	M
7	B12 deficient_7	81.802168	52	M	54	Control_7	406.097561	37	M
8	B12 deficient_8	83.9837398	49	F	55	Control_8	406.5	21	F
9	B12 deficient_9	88.02	19	F	56	Control_9	407.2	58	F
10	B12 deficient_10	88.3468835	50	F	57	Control_10	410	50	M
11	B12 deficient_11	90.8943089	60	F	58	Control_11	411.382114	56	F
12	B12 deficient_12	95	51	M	59	Control_12	424.4	44	M
13	B12 deficient_13	95.27	20	F	60	Control_13	427.506775	54	F
14	B12 deficient_14	97.2764228	40	F	61	Control_14	433.333333	40	F
15	B12 deficient_15	98.495935	58	F	62	Control_15	434.7	36	F
16	B12 deficient_16	99.0108401	37	M	63	Control_16	437.127371	61	F
17	B12 deficient_17	99.7154472	55	M	64	Control_17	444.03794	51	M
18	B12 deficient_18	104	50	M	65	Control_18	447.831978	36	F
19	B12 deficient_19	106.5	20	M	66	Control_19	454.7	20	M
20	B12 deficient_20	109.701897	21	F	67	Control_20	458.672087	40	M
21	B12 deficient_21	110.447155	49	F	68	Control_21	462.872629	50	F
22	B12 deficient_22	111.7	54	M	69	Control_22	465.582656	58	F

23	B12 deficient_23	112.059621	50	M	70	Control_23	476	58	M
24	B12 deficient_24	115.460705	35	F	71	Control_24	481.5	23	M
25	B12 deficient_25	118.279133	23	M	72	Control_25	482.249323	30	F
26	B12 deficient_26	122.669377	44	M	73	Control_26	503.9	20	M
27	B12 deficient_27	123.98374	41	M	74	Control_27	504.5	18	F
28	B12 deficient_28	127.2	20	F	75	Control_28	513.4	30	F
29	B12 deficient_29	127.7	20	F	76	Control_29	526.96477	52	M
30	B12 deficient_30	128.401084	26	F	77	Control_30	530.352304	36	F
31	B12 deficient_31	128.468835	37	F	78	Control_31	532.8	18	M
32	B12 deficient_32	130.9	21	M	79	Control_32	553.252033	60	M
33	B12 deficient_33	132.777778	30	F	80	Control_33	554.336043	41	F
34	B12 deficient_34	134.6	37	M	81	Control_34	562.330623	53	F
35	B12 deficient_35	137.127371	32	F	82	Control_35	575.338753	51	F
36	B12 deficient_36	139.159892	51	F	83	Control_36	587.3	57	M
37	B12 deficient_37	140.1	21	F	84	Control_37	589	51	M
38	B12 deficient_38	141.192412	56	F	85	Control_38	591.056911	52	F
39	B12 deficient_39	142.411924	53	F	86	Control_39	614.227642	60	M
40	B12 deficient_40	143.224932	54	F	87	Control_40	637.9	22	F
41	B12 deficient_41	144.4	39	M	88	Control_41	643	37	F
42	B12 deficient_42	145.663957	42	F	89	Control_42	719.3	49	F
43	B12 deficient_43	145.934959	58	M	90	Control_43	741.4	55	M
44	B12 deficient_44	146.7	18	M	91	Control_44	750	35	F
45	B12 deficient_45	147.154472	38	M	92	Control_45	758.4	20	F
46	B12 deficient_46	147.6	52	F	93	Control_46	789.295393	25	F
47	B12 deficient_47	147.9	18	M					

Supplementary-Table S6: Spike and recovery

Sample Name _(n=3)	% RECOVERY	RSD	REFERENCE	RSD
SM	105.26	5.90	100.00	3.82
CER	100.32	25.68	100.00	23.61
TAG	113.19	4.15	100.00	27.42
DAG	137.25	5.73	100.00	17.81
LPC	97.03	8.77	100.00	1.98
PC	75.04	28.03	100.00	20.85
LPE	76.28	34.75	100.00	31.83
PE	80.90	5.90	100.00	8.80
PG	105.52	7.58	100.00	2.99
PI	69.75	19.67	100.00	15.36
PS	74.38	24.59	100.00	36.81
PA	74.96	31.19	100.00	25.19

Supplementary- Table S7: Fold change and p-value of different lipid classes in vitamin B12 deficient study.

S.No.	Sample Name	Fold Change (Low B12/ Normal B12	p-value
1	SM	0.979	0.819
2	CE	1.048	0.657
3	CER	1.014	0.921
4	TAG	1.064	0.454
5	DAG	1.023	0.818
6	LPC	1.016	0.779

7	PC	0.975	0.557
8	LPE	0.864	0.456
9	PE	0.858	0.192
10	LPG	0.720	0.262
11	PG	0.842	0.337
12	PI	0.978	0.834
13	PS	0.691	0.103
14	PA	0.812	0.233

Supplementary- Table S8: DE selected lipids.

S.No.	Lipid Name	Fold change	P-value
1	TAG(54:7/FA18:2)	0.5814	0.0037
2	TAG(58:10/FA22)	0.6081	0.0026
3	PA(18:0/22:4)	0.6209	0.0039
4	TAG(54:6/FA18:2)	0.6304	0.0009
5	TAG(50:4/FA14:0)	0.6602	0.0018
6	PE(18:2/22:4)	0.6660	0.0025
7	TAG(54:7/FA18:3)	0.6693	0.0058
8	PE(P-18:2/18:2)	0.6885	0.0071
9	TAG(50:5/FA18:2)	0.6927	0.0030
10	TAG(48:5/FA18:2)	0.6961	0.0059
11	PG(18:1/20:3)	0.7001	0.0327
12	TAG(46:4/FA18:2)	0.7026	0.0123
13	TAG(54:7/FA22:5)	0.7066	0.0003
14	TAG(56:5/FA20:1)	0.7067	0.0058
15	TAG(52:4/FA20:0)	0.7102	0.0005
16	TAG(56:5/FA18:2)	0.7110	0.0055
17	TAG(60:10/FA22:6)	0.7112	0.0070
18	TAG(58:9/FA22:6)	0.7138	0.0296
19	TAG(54:4/FA18:0)	0.7156	0.0004
20	TAG(50:4/FA18:2)	0.7165	0.0025
21	TAG(52:5/FA18:2)	0.7173	0.0005

22	TAG(56:8/FA18:2)	0.7173	0.0077
23	TAG(54:5/FA18:2)	0.7173	0.0009
24	TAG(48:4/FA18:2)	0.7207	0.0144
25	PE(P-18:0/22:4)	0.7232	0.0067
26	TAG(56:7/FA18:3)	0.7242	0.0065
27	PG(18:0/20:2)	0.7290	0.0035
28	TAG(56:6/FA18:2)	0.7296	0.0021
29	TAG(53:4/FA17:0)	0.7314	0.0006
30	TAG(54:6/FA18:3)	0.7317	0.0075
31	TAG(52:6/FA18:2)	0.7320	0.0110
32	TAG(52:4/FA18:2)	0.7407	0.0003
33	TAG(55:5/FA18:2)	0.7426	0.0056
34	TAG(56:3/FA20:0)	0.7430	0.0086
35	TAG(56:6/FA18:3)	0.7472	0.0360
36	TAG(52:5/FA16:1)	0.7482	0.0025
37	TAG(46:3/FA18:2)	0.7537	0.0020
38	LPE(22:4)	0.7542	0.0096
39	TAG(52:4/FA16:0)	0.7558	0.0004
40	TAG(54:5/FA18:1)	0.7568	0.0019
41	TAG(54:6/FA18:1)	0.7604	0.0178
42	TAG(52:2/FA20:0)	0.7609	0.0037
43	TAG(51:2/FA18:1)	1.3127	0.0008
44	TAG(53:1/FA17:0)	1.3412	0.0389
45	PC(16:0/20:5)	1.3445	0.0039
46	PE(18:0/20:5)	1.3531	0.0167
47	TAG(50:0/FA18:0)	1.3566	0.0308
48	TAG(50:1/FA18:0)	1.3575	0.0080
49	TAG(52:6/FA20:5)	1.3651	0.0092
50	PE(18:1/18:3)	1.3844	0.0055
51	HCER(20:0)	1.4028	0.0347
52	PA(20:0/20:5)	1.4080	0.0153
53	PS(18:2/22:6)	1.5529	0.0080
54	LPC(20:5)	1.5642	0.0012
55	PG(14:0/14:0)	2.0954	0.0404
